# An analysis of the global, regional, and national epidemiology and trends of Alzheimer's disease and other dementias linked to smoking from 1990 to 2021 and projections to 2050

**DOI:** 10.18332/tid/207127

**Published:** 2025-06-29

**Authors:** Hongdou Xu, Liang Yang, Shibin Hu, Xuran Xu, Yuan Yang

**Affiliations:** 1 Department of Interventional Radiology, Gaochun Peoples Hospital, Jiangsu University, Nanjing, China; 2 Department of Ultrasound, Gaochun Peoples Hospital, Jiangsu University, Nanjing, China

**Keywords:** Alzheimer's disease, dementia, smoking, disease burden, epidemiological projections

## Abstract

**INTRODUCTION:**

This research assesses the smoking-related impact on Alzheimer's disease and other dementias (ADOD), analyzing variables such as sex, age, sociodemographic index (SDI), region, and country from 1990 to 2021, with forecasts to 2050.

**METHODS:**

Using data from the Global Burden of Disease Study 2021, we examined smoking-related ADOD trends from 1990 to 2021, focusing on deaths, disability-adjusted life years (DALYs), years of life lost (YLLs), and years lived with disability (YLDs) by age, sex, SDI, location, and country. We quantified trends with estimated annual percentage changes and used decomposition analysis to evaluate the effects of population growth, aging, and epidemiological shifts. A frontier analysis identified improvement areas and disparities among countries by development status. Time series prediction models were used to predict smoking-attributable ADOD trends from 2022 to 2050, considering population profiles.

**RESULTS:**

Between 1990 and 2021, there was an observable upward trend in deaths, DALYs, YLLs, and YLDs. In 2021, the burden of smoking-attributable age-related diseases predominantly impacted males across all age groups. Females, however, experienced a more pronounced reduction in age-standardized rates (ASR) of deaths, DALYs, YLLs, and YLDs compared to their male counterparts. The data from 2021 reveal that ASR of deaths, DALYs, and YLLs increased with age, reaching a peak among individuals aged ≥95 years. These ASR trends were consistent across genders, although higher rates were observed in males than in females. In 2021, the high-middle SDI region recorded the highest ASR of deaths, DALYs, YLLs, and YLDs. All five SDI regions experienced declines in ASR of deaths, DALYs, YLLs, and YLDs, with the high-SDI region demonstrating the most significant reductions in the estimated annual percentage change (EAPC). Decomposition analyses suggested that population growth was the primary factor contributing to the increase in overall deaths.

**CONCLUSIONS:**

From 1990 to 2021, there was an increase in deaths, DALYs, YLLs, and YLDs attributable to smoking-related ADOD, with projections indicating a continued rise globally until 2050. The burden of disease is mainly caused by males and middle-aged and elderly people, which should be given sufficient attention. Understanding epidemiological factors is crucial for designing effective, tailored interventions to mitigate the global burden.

## INTRODUCTION

Alzheimer’s disease and other dementias (ADOD) affect memory, language, and behavior, with >55 million individuals currently diagnosed worldwide. This number is projected to triple by 2050^1^.These disorders impose substantial strain on healthcare infrastructures and socioeconomic frameworks globally. In 2019, dementia’s global economic impact was US$1.3 trillion, with each prevented Alzheimer’s disease case saving about $387000 in societal costs^2^. Although symptomatic therapies are available for dementias, current interventions fail to modify disease progression, and no pharmacological agents target underlying neuropathology^3^.

Recent evidence indicates that up to 45% of dementia cases associated with 14 modifiable risk factors, including metabolic disorders (obesity, diabetes), behavioral factors (smoking, excessive alcohol use), and limited education level^1-3^. Among these factors, smoking has emerged as a prominent contributor, implicating mechanisms such as cerebral oxidative stress in disrupting neurobiological pathways^4^.

Numerous studies have investigated the burden of ADOD at global, regional, and national levels. Several studies have examined the impact of modifiable risk factors, such as smoking, on dementia^1,5^. However, the previous study only characterized the relationship between ADOD death and smoking in 195 countries from 1990 to 2017 and did not reveal the elements in the change of smoking-related ADOD and future trends on a global scale^6^. Comprehensive analysis of the burden of smoking-related dementia using statistical measures such as disability-adjusted life years (DALY), years of life lost (YLLs), and years lived with disability (YLDs) by sex, age, and sociodemographic index (SDI) levels is still limited.

This study utilized the Global Burden of Disease Study (GBD) 2021 data to comprehensively analyze the 2021 burden and evolving trends of ADOD associated with smoking risks, considering age, sex, global, GBD subcontinental region, socioeconomic, and national levels from 1990 to 2021. We also aim to forecast future epidemiological trends up to 2050.

## METHODS

### Study population and data collection

The GBD 2021 database encompasses 369 diseases and injuries across 204 countries and territories from 1990 to 2021, employing rigorous methodologies and diverse data sources to ensure accurate and comprehensive estimates. ADOD diagnoses were determined using ICD-10 codes F00, F01, F02, F03, G30, and G31, as outlined by the GBD^7,8^.

### Estimation of disease burden

The study utilized four metrics – death rates, DALYs, YLLs, and YLDs – along with their quantified forms, including case numbers and age-standardized rates (ASR), to illustrate the disease burden associated with smoking-attributable ADOD. This study identifies eight indicators of disease burden: the number of deaths, the age-standardized death rate (ASDR), the number of DALYs, age-standardized DALYs rate (ASDAR), number of YLLs, age-standardized YLLs rate, number of YLDs, and age-standardized YLDs rate. DALYs, calculated by summing YLLs and YLDs, provide a comprehensive measure of health burden by reflecting both fatal and non-fatal health losses. YLLs are calculated by multiplying disease-specific death counts by the standard expected remaining lifespan, whereas YLDs represent the years lived with disability caused by the disease^9^. Estimates are presented as case numbers and ASR per 100000 population, each with 95% uncertainty intervals (UIs).

### Sociodemographic index

The sociodemographic index (SDI) serves as a comprehensive measure of economic and developmental status, closely linked to health outcomes. The measure incorporates per capita income, average schooling, and birth rate figures to present a comprehensive evaluation of development levels in all regions covered by the GBD study. The SDI ranges from 0 to 1, classifying the world into five regions: low, low-middle, middle, high-middle, and high SDI^10^.

### Decomposition and frontier analysis

We employed decomposition analysis to disentangle and quantify the relative contributions of three principal drivers to the observed changes in the absolute number of deaths, DALYs, YLLs, and YLDs attributable to smoking-related ADOD from 1990 to 2021. These drivers include: 1) Population growth, which pertains to changes solely attributable to increases in total population size; 2) Population aging, which involves changes resulting from shifts in the age distribution of the population, specifically an increase in the proportion of older individuals who are at higher risk; and 3) Epidemiological change, which encompasses changes in the underlying age-specific risk or disease rates, reflecting the cumulative impact of factors such as variations in smoking prevalence, exposure intensity, diagnostic practices, and treatment effectiveness over time. This analytical approach enables us to attribute the total observed increase or decrease in burden to each of these distinct demographic and epidemiological factors^11^. Furthermore, we utilized frontier analysis to assess the efficiency of various countries in managing the smoking-related ASR of deaths, DALYs, YLLs, and YLDs in relation to their sociodemographic development, as indicated by the SDI. This method establishes a statistical ‘frontier’ that represents the optimal (lowest) ASR achievable for a given SDI level, derived from global data spanning 1990 to 2021. The frontier effectively delineates the minimum disease burden theoretically attainable for a country at its specific stage of development. Subsequently, we computed the effective difference, or distance to the frontier, for each country in 2021, defined as the absolute difference between the observed ASR and the corresponding frontier value at its SDI. A greater effective difference suggests a higher potential for improvement in mitigating the smoking-related burden of disease compared to the best-performing countries at similar development levels. This analysis facilitates the identification of countries that are underperforming relative to their SDI counterparts and highlights areas where targeted interventions could achieve the most substantial improvements^12^.

### Forecasting the worldwide impact of smoking-related ADOD

Autoregressive integrated moving average (ARIMA) and exponential smoothing (ES) were utilized in R software to forecast the number and ASR of deaths, DALYs, YLLs, and YLDs from 2020 to 2050. The time series of smoking-attributable ADOD from 1990 to 2021 was analyzed using R software, utilizing data on deaths, DALYs, YLLs, and YLDs to investigate trend changes. The ARIMA model, which incorporates auto-regressive, integrated, and moving average components, was employed to examine and forecast the time-series data. The optimal model was selected using the standardized Bayesian Information Criterion (BIC), and a white noise test was performed to evaluate the model at a 0.05 significance level^13^. The ES model utilizes an exponentially weighted average method for time series forecasting, estimating future values by calculating the weighted average of previous observations^14^.

### Statistical analysis

We initially analyzed the burden and temporal trends of smoking-related ADOD, considering variables such as sex, age, SDI, region, and country. Pearson correlation and linear regression analyses were employed to examine the relationship between ASDR, ASDAR, and SDI. The trend in disease burden from 1990 to 2021 was assessed using the estimated average percentage change (EAPC) of age-standardized rates, with a 95% confidence interval (CI) derived from a linear regression model. The assessment of driving factors and potential improvements was performed using decomposition and frontier analysis. Ultimately, predictions of the trend in smoking-related ADOD burden up to 2050 were made using time-series forecasting models such as the ARIMA and ES models, utilizing RStudio (version 4.1.1).

## RESULTS

### The global smoking-attributable burden of ADOD

**Figure 1:**
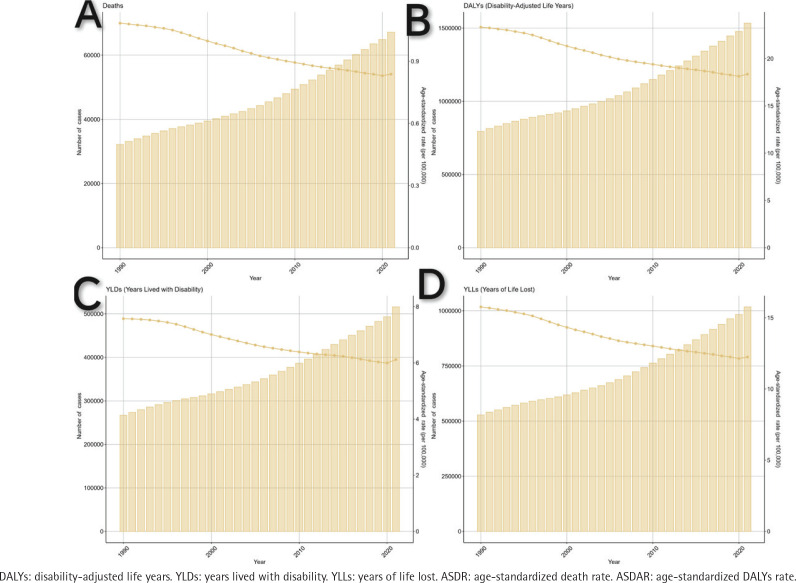
Analysis of global burden indicators for smoking-related Alzheimer’s disease and other dementias from 1990 to 2021. Trends are depicted using column bar graphs and line charts illustrate the age-standardized rates per 1000000 population: A) deaths; B) DALYs; C) YLDs; and D) YLLs

The global burden of smoking-attributable ADOD in 1990 and 2021 is shown in Supplementary file Tables S1–S4. Between 1990 and 2021, the number of deaths related to ADOD increased from 32165 (95% UI: 7446–8932) to 67176 (95% UI: 15695–184665), and DALYs rose from 794915 (95% UI: 344378–1839709) to 1533214 (95% UI: 662723–349642), with overall increases of 108.84% and 92.88%, respectively. The number of YLLs increased from 528022 (95% UI: 122674–1511230) to 1017331 (95% UI: 241832–2876261), and YLDs increased from 266894 (95% UI: 162663–391752) to 515882 (95% UI: 313106–759417), representing overall increases of 93.29% and 92.67%. The ASR of deaths declined from 1.08 (95% UI: 0.25–3.03) to 0.84 (95% UI: 0.19–2.29), and DALYs decreased from 23.33 (95% UI: 9.99–54.46) to 18.36 (95% UI: 7.90–42.07), reflecting overall reductions of 22.22% and 21.30%, respectively. The ASR of YLLs fell from 15.76 (95% UI: 3.62–44.13) to 12.24 (95% UI: 2.90–34.28), and YLDs fell from 7.57 (95% UI: 4.64–11.22) to 6.12 (95% UI: 3.72–9.03), with overall reductions of 22.33% and 19.15% (Figures 1A–1D).

### Global smoking-attributable ADOD burden by sex

There was a marked difference between sexes in smoking-attributable ADOD burden, with males experiencing approximately 2–3 times the burden of females. In 2021, the age-standardized death rate (ASDR) for males was 1.43 (95% UI: 0.34–4.05) and the age-standardized DALY rate (ASDAR) was 30.56 (95% UI: 12.72–71.5), significantly higher than females (ASDR=0.43; 95% UI: 0.10–1.14; ASDAR=9.01; 95% UI: 3.94–20.16). Deaths and DALYs were 46755 (95% UI: 11061–132714) and 1110023 (95% UI: 474038–2558503) for males, versus 20420 (95% UI: 4946–53998) and 423191 (95% UI: 185085–947536) for females. A comparable sex disparity existed for YLLs and YLDs (Supplementary file Tables S3 and S4). Both ASDR and ASDAR declined in both sexes, with estimated annual percentage changes (EAPCs) of -0.95 (95% CI: -0.99 – -0.91) and -0.88 (95% CI: -0.90 – -0.83), respectively. Declines were more pronounced in females (ASDR EAPC= -1.60; 95% CI: -1.68 – -1.52; ASDAR EAPC= -1.50; 95% CI: -1.56 – -1.45) than males (ASDR EAPC= -0.79; 95% CI: -0.85 – -0.73; ASDAR EAPC= -0.73; 95% CI: -0.80 – -0.60). By 2021, despite decreased age-standardized rates (ASR) of years of life lost (YLLs) and years lived with disability (YLDs) in both sexes, males had higher absolute numbers: YLLs were 737183 (95% UI: 177427–2115307) for males versus 280148 (95% UI: 65583–771699) for females, and YLDs were 372840 (95% UI: 228453–545265) versus 143042 (95% UI: 84582–215146). Epidemiological patterns of ASR for YLLs and YLDs showed similar trends (Supplementary file: Tables S1–S4, Figure S1).

### Smoking-attributable ADOD burden by age

Among individuals aged 70–89 years, smoking exposure was the primary contributor to the ADOD burden. As age increased, there was an upward trend in deaths, DALYs, YLLs, and YLDs. From 1990 to 2021, both ASDR and ASDAR exhibited a downward trend across all age groups, with the most pronounced decline in the above 95 years group, reflected by an EAPC of -1.56 (95% CI: -1.63 – -1.49) for ASDR and -1.62 (95% CI: -1.69 – -1.55) for ASDAR. Subgroup analysis by sex revealed a higher smoking-attributable burden in males compared to females. For both males and females, the number of deaths and YLDs due to ADOD from smoking increased with age, peaking at 80–84 and 70–74 years for males, and at 85–89 and 80–84 years for females, before declining annually. Furthermore, the rates of deaths, DALYs, and YLLs for ADOD increased with age in both males and females, peaking in individuals aged >95 years. For both males and females, the YLD rate increased with age, reaching its highest point among individuals aged 90–94 years before declining (Supplementary file: Tables S1–S4, Figure S2).

### Smoking-attributable ADOD burden by SDI

In 2021, the ASR of deaths, DALYs, YLLs, and YLDs were highest in high-middle SDI regions, with ASDR at 0.96 (95% UI: 0.22–2.6), ASDAR at 21.96 (95% UI: 9.6–49.15), ASR of YLLs at 14.41 (95% UI: 3.4–40), and ASR of YLDs at 7.55 (95% UI: 4.5–11.14). Conversely, these rates were lowest in low SDI regions. The middle SDI regions exhibited the highest DALYs, YLLs, and YLDs among the five groups, while the high SDI regions recorded the highest number of deaths. Between 1990 and 2021, the ASR of deaths, DALYs, YLLs, and YLDs declined across the five SDI regions, with the high SDI regions experiencing the most notable decrease. Conversely, the absolute numbers of deaths, DALYs, YLLs, and YLDs increased, especially in the middle SDI region (Supplementary file: Tables S1–S4, Figure S3).

This research analyzed the decomposition of original deaths, YLLs, YLDs, and DALYs for ADOD to investigate the effects of ageing, population growth, and epidemiological transitions on the ADOD burden. Globally, the DALYs and YLDs of both sexes for ADOD demonstrated an increasing trend. The decrease in deaths and YLLs was primarily attributed to the negative impact of epidemiological changes in females. Globally, the middle SDI region experienced the highest increase in DALYs and YLDs, primarily due to population growth, followed by ageing. Population growth contributed 120.62% and ageing 73.43% to the rise in disease burden. There was a negative contribution from epidemiological changes. The impact of epidemiological change differed across SDI regions, showing a notable increase of 142.84% in high SDI regions, while low, low-middle, middle, and high-middle SDI regions experienced decreases of 24.74%, 39.62%, 54.59%, and 89.65%, respectively. The adverse effect of epidemiological change on disease burden increases with higher SDI levels. Afterwards, we conducted a stratification analysis based on sex. Both DALYs and YLDs exhibited similar traits in males and females. Nevertheless, there was an increasing trend in deaths and YLLs among males. In the central SDI region, where the disease burden increased most significantly, the impact of male population growth was most notable (Supplementary file: Figure S4).

We conducted a frontier analysis using ASR of deaths, YLLs, YLDs, and DALYs alongside SDI levels from 1990 to 2021 to better understand potential improvements in the burden of ADOD relative to national development levels. Considering national development in the context of ASDR and ASDAR, the countries with the greatest potential for improvement include Egypt, Rwanda, Tunisia, Paraguay, Lebanon, Kiribati, Jordan, Iraq, Greece, Cambodia, Algeria, Albania, Denmark, and China. Countries with a low SDI (<0.5) include Somalia, Burkina Faso, Guinea-Bissau, and Benin. Despite their high SDI (>0.85), countries such as the Republic of Korea, Sweden, the United States, the Netherlands, and Denmark still possess significant potential for further progress relative to their development level. ASR of YLLs and YLDs, showed similar characteristics (Supplementary file: Figure S5).

### Smoking-attributable ADOD burden by regional and national location

The 2021 disease burden of smoking-attributable ADOD and its trends were analyzed by geographical distribution, including GBD subcontinental regions and countries, with results visualized (Supplementary file Tables S1–S4). East Asia exhibited the highest ASR for deaths, DALYs, YLLs, and YLDs at 1.38 (95% UI: 0.32–3.87), 29.95 (95% UI: 12.67–68.1), 10.09 (95% UI: 6.08–15.1), and 19.86 (95% UI: 4.66–56.37), respectively. In contrast, Western Africa and Western Sub-Saharan Africa recorded the lowest age-standardized death rates (ASDR) at 0.14 (95% UI: 0.03–0.41), age-standardized DALYs at 2.89 (95% UI: 1.12–7.07), YLLs at 2.08 (95% UI: 0.49–6.14), and YLDs at 0.81 (95% UI: 0.49–1.2). Asia reported the highest figures for deaths, DALYs, YLLs, and YLDs, with values of 300.55 (95% UI 226.03–384.96), 958325 (95% UI: 412336–2196528), 635231 (95% UI: 151073–1802835), and 323094 (95% UI: 196254–476426), respectively (Supplementary file: Tables S1–S4, Figure S6).

From 1990 to 2021, most regions exhibited a slight upward trend in ASDR and ASDAR. Eastern Europe experienced the most significant rise in ASR of deaths and YLLs, with EAPCs of 1.23 (95% CI: 0.77–1.69) and 1.09 (95% CI: 0.57–1.6), respectively. East Asia experienced the highest rise in ASR of DALYs and YLDs, with EAPCs of 1.11 (95% CI: 0.62–1.6) and 1.46 (95% CI: 1.01–1.91), respectively. Southern Sub-Saharan Africa experienced the most significant reductions in ASR of deaths, DALYs, YLLs, and YLDs, with EAPCs of -2.15 (95% CI: -3.69 – -0.58), -2.07 (95% CI: -3.03 – -1.11), -2.2 (95% CI: -3.1 – -1.29), and -2.01 (95% CI: -3 – -1.01), respectively. The extensive evaluation of the EAPC for all indicators, followed by cluster analysis, indicated a notable decrease in the ADOD disease burden associated with smoking in five regions, a significant increase in two regions, a slight increase in 31 regions, and stability or a slight decrease in 10 regions ( Supplementary file: Figure S7).

In 2021, Lebanon recorded the highest ASR for deaths, DALYs, YLLs, and YLDs at 1.59 (95% UI: 0.39–4.44), 35.63 (95% UI: 15.39–80.46), 13.37 (95% UI: 8.11–20.29), and 22.26 (95% UI: 5.41–63.5), respectively (Supplementary file: Figure S8). From 1990 to 2021, the fastest increase in ASDAR was observed in Albania (EAPC=2.51; 95% CI: 1.99–3.03), and the fastest decrease was in Madagascar (EAPC= -3.08; 95% CI: -4.93–1.19). Between 1990 and 2021, Georgia experienced the most rapid increase in ASDR, with an EAPC of 2.82 (95% CI: 2.17–3.48), while Madagascar saw the steepest decline, with an EAPC of -3.34 (95% CI: -5.56 – -1.06). Similar results can also be found in the ASR of YLLs, and YLDs (Supplementary file: Tables S3 and S4, Figure S9).

### Predictions of the smoking-attributable ADOD burden

The ARIMA and ES model predictions suggest a global increase in the number and ASR of deaths, DALYs, YLLs, and YLDs due to ADOD from 1990 to 2021. By 2050, projections indicate an upward trend in global ASDR, ASDAR, YLLs, and YLD, with the most notable rise among males, with an EAPC of 2.06 (95% CI: 1.34–2.79), 46.19 (95% CI: 29.76–62.62), 24.16 (95% CI: 17.94–30.38), and 23.15 (95% CI: 12.73–33.58), respectively, according to the ARIMA model (Supplementary file: Figure S10). The ARIMA model predicted that the ASDR and ASDAR for females would remain relatively stable, with a slight variation. This trend was similarly observed in the ES model (Supplementary file: Figure S11).

## DISCUSSION

This study thoroughly examined the impact of smoking-related ADOD across various sexes, age groups, SDI quintiles, GBD regions, and countries from 1990 to 2021, and projected burdens up to 2025. Our findings indicate an increase in global smoking-related deaths, DALYs, YLLs, and YLDs of ADOD, which is projected to persist until 2050. However, the corresponding ASR for both sexes globally decreased between 1990 and 2021. The findings may be due to two factors: the population’s growth and ageing, which have intensified smoking and increased disease burden, and global anti-smoking initiatives over recent decades, such as price and tax controls, public smoking bans, public awareness campaigns about tobacco risks, and enhanced global cooperation, as outlined in the WHO Framework Convention on Tobacco Control (FCTC)^8-11^. At the same time, it also implies actions to control tobacco use are starting to be effective and calls for ongoing implementation of targeted policies and programs to lower the smoking-attributable burden of dementias.

Most studies indicate that Alzheimer’s disease dementia is more prevalent and incident in females than in males. Possible explanations include a lower level of literacy, carrying the ApoE4 genotype, and a longer life span in females^12-15^. Our study indicates that smoking-related ADOD affects males approximately 2 to 3 times more than females across all age groups, aligning with findings by Jiang et al.^6^. The explanation could be that more males smoke than females, and females are less likely to accept smoking. A previous study indicated significant gender disparities in global tobacco consumption patterns. Among male populations, the age-adjusted prevalence rate for daily tobacco use stood at 25.0% (95% UI: 24.2–25.7), while female prevalence rates were markedly lower at 5.4% (95% UI: 5.1–5.7). In addition, male smoking rates demonstrated a 28.4% reduction (95% UI: 25.8–31.1) and female rates showed an even more pronounced decrease of 34.4% (95% UI: 29.4–38.6) when accounting for population aging effects through standardized measurement methodologies^16^. In addition, despite a more marked decrease in smoking prevalence among males compared to females over the past decades^17-19^, the decreasing extent of their ASR of smoking-related ADOD was more significant in females in this study. This could be due to the protective effects of estrogen in cardiovascular function and females also show greater concern for healthcare and disease prevention compared to males^20,21^. The gender disparity in smoking-related ADOD implies a need for targeted smoking prevention strategies to be effectively implemented for males. This study analyzed the mortality and ARS of deaths, DALYs, YLLs, and YLDs due to smoking-related ADOD across all age groups, identifying a declining trend in ARS for most age categories from 1990 to 2021.This decline might be linked to global anti-smoking policies and economic development. The burden of smoking-related ADOD is low in young adulthood. It increases with age, peaking in people aged >90 years in both males and females, mainly due to the cumulative effect of prolonged exposure to the risk factor of smoking. Prevention efforts should target middle-aged and older individuals.

The distribution of smoking-related ADOD burden varied significantly across regions and countries. The high-middle SDI region exhibited the highest ASR for deaths, DALYs, YLLs, and YLDs related to smoking-induced ADOD, with trends remaining stable or slightly declining. This implies that the high-middle SDI region will play a vital role in smoking-related ADOD prevention and education strategies. The high-SDI regions experienced the greatest EAPC in ASR of deaths, likely due to the adoption of extensive tobacco control strategies, such as legislative actions (e.g. tax policies, smoke-free regulations) and population-wide initiatives (mass media campaigns, cessation services) as advised by WHO FCTC guidelines^22^. Decomposition modelling identifies epidemiological change as the predominant driver of burden reconfiguration in high-SDI regions. The observed disparity may result from the combined effects of superior health system performance in high-SDI countries, marked by integrated care networks covering 92.3% of the population compared to 48.7% in low-SDI areas, and advanced medical infrastructure with 8.6 physicians per 1000 people versus 1.4^23-25^. Additionally, our decomposition analysis revealed that population growth is the main factor influencing the transition of smoking-related ADOD burden in low-middle SDI regions. While higher SDI generally correlated with reduced effective distance, select low-SDI nations (Somalia, Burkina Faso, Guinea-Bissau, Benin) demonstrated exceptional efficiency, achieving neoplasm burdens comparable to optimally-resourced systems despite constraints.

The study revealed that in 2021, Asia, particularly East Asia, had the highest counts and ASR of deaths, DALYs, YLLs, and YLDs. China, home to over 300 million adult smokers, faces an increased smoking-related ADOD burden due to its rapidly ageing population, with more than 18% aged ≥60 years. Age is the primary risk factor for dementia, with smoking contributing to faster cognitive decline^26,27^. The intersection of widespread smoking and a massive ageing cohort creates a ‘double burden’, unlike younger populations in many low- and middle-income countries^28^. Our trend analysis indicated a slight increase in the age-standardized rates of deaths, DALYs, YLLs, and YLDs associated with smoking-related ADOD burden across most geographical regions. Notable increases were observed in certain regions, including Eastern Europe and Central Asia. The remarkable increase is largely due to smoking prevalence high rates, weak policy implementation, and historical drivers. For example, in Albania, WHO (2021) reports adult male smoking rates at 45% and female rates at 15%, exceeding global averages. Albania joined the WHO Framework Convention on Tobacco Control (FCTC) in 2007; however, the enforcement has been ineffective, as evidenced by the limited impact of the 2014 public smoking ban and restrictions on tobacco advertising^29-35^. Using ARIMA and ES models to forecast the number and ASR of deaths, DALYs, YLLs, and YLDs related to smoking-induced ADOD burden from 2022 to 2050, indicated a mildly increasing trend. Furthermore, the increase in smoking-related ADOD burden was mainly due to males.

### Limitations

This study has several limitations. The GBD study’s methodology has recognized limitations, such as biases and gaps, particularly in low- and middle-income countries with high smoking rates and inadequate healthcare resources. Secondly, the study did not analyze smoking dose (daily cigarettes), duration (age of initiation, years since cessation), and secondhand smoke exposure, which may have had an impact on the estimate of the burden of smoking-related ADOD. Finally, the long latency period of ADOD (up to decades) may lead to underestimation of smoking’s long-term effects due to insufficient follow-up in many studies.

## CONCLUSIONS

The study found that from 1990 to 2021, the ARS burden of smoking-related ADOD declined, accompanied by an increase in deaths, DALYs, YLLs, and YLDs associated with smoking-related ADOD. The study suggests that future strategies should prioritize addressing the disparities in smoking-related ADOD burden among males, the elderly, high- and middle-SDI regions, and areas with high smoking prevalence.

## Supplementary Material



## Data Availability

The data supporting this research are available from the authors on reasonable request.
