# Supplementary material for: An analysis of the global, regional, and national epidemiology and trends of Alzheimer's disease and other dementias linked to smoking from 1990 to 2021 and projections to 2050
Source: Tob Induc Dis. 2025 Jun 29;23:10.18332/tid/207127. doi: 10.18332/tid/207127 (PMC12306450; doi:10.18332/tid/207127)
Supplement: Supplementary file 1 [file TID-23-110-s1.pdf]

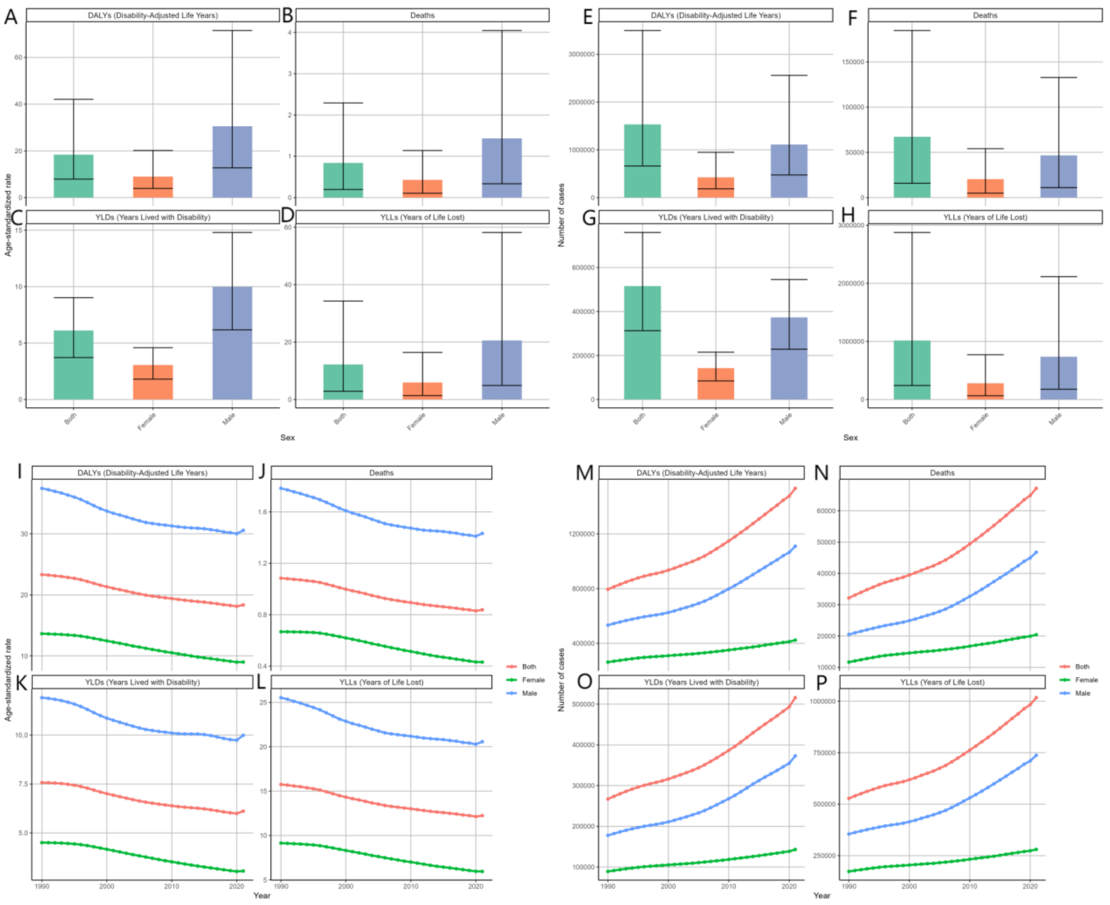

Supplementary Figure S1. Global burden and evolving trends in smoking-related Alzheimer's disease and other dementias by sex from 1990 to 2021.(A, I) ASDAR.(B, J) ASDR.(C, K) age-standardized YLDs rate.(D, L) age-standardized YLLs rate.(E,M) number of DALY cases.(F, N) number of death cases.(G, O)number of YLD cases.(H, P) number of YLL cases. DALY: disability-adjusted life years; ASDR: age-standardized death rate; ASDAR: age-standardized DALYs rate; YLLs: years of life lost; YLDs: years lived with disability.

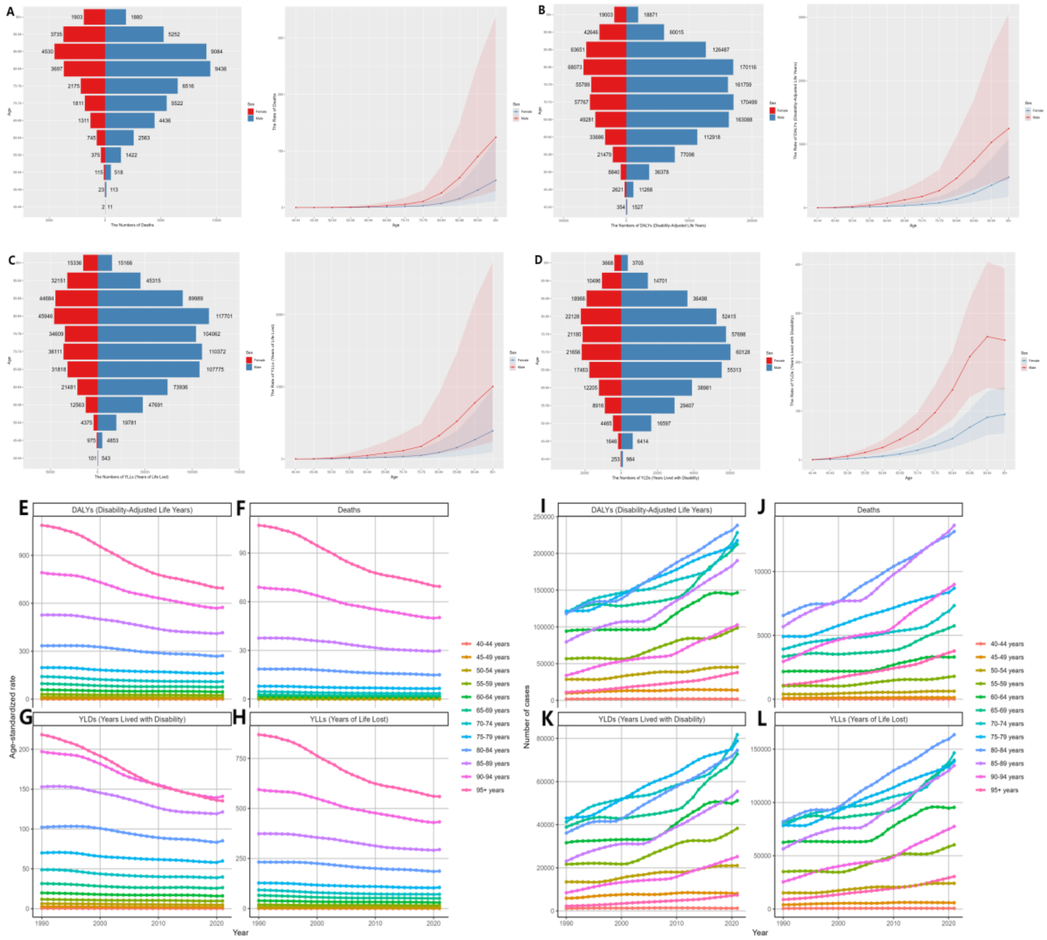

Supplementary Figure S2. The 2021 global burden distribution and changing patterns of smoking-related Alzheimer's disease and other dementias from 1990 to 2021, analyzed by sex and age group.(A)Number of death cases and ASDR.The study examines the number of Disability-Adjusted Life Years (DALYs) cases and the Age-Standardized DALYs Rate (ASDAR).Number and age-standardized rate of YLLs (Years of Life Lost) and YLDs (Years Lived with Disability).DALYs stands for disability-adjusted life years; ASDR refers to the age-standardized death rate; ASDAR denotes the age-standardized DALYs rate; YLLs represent years of life lost; YLDs indicate years lived with disability.

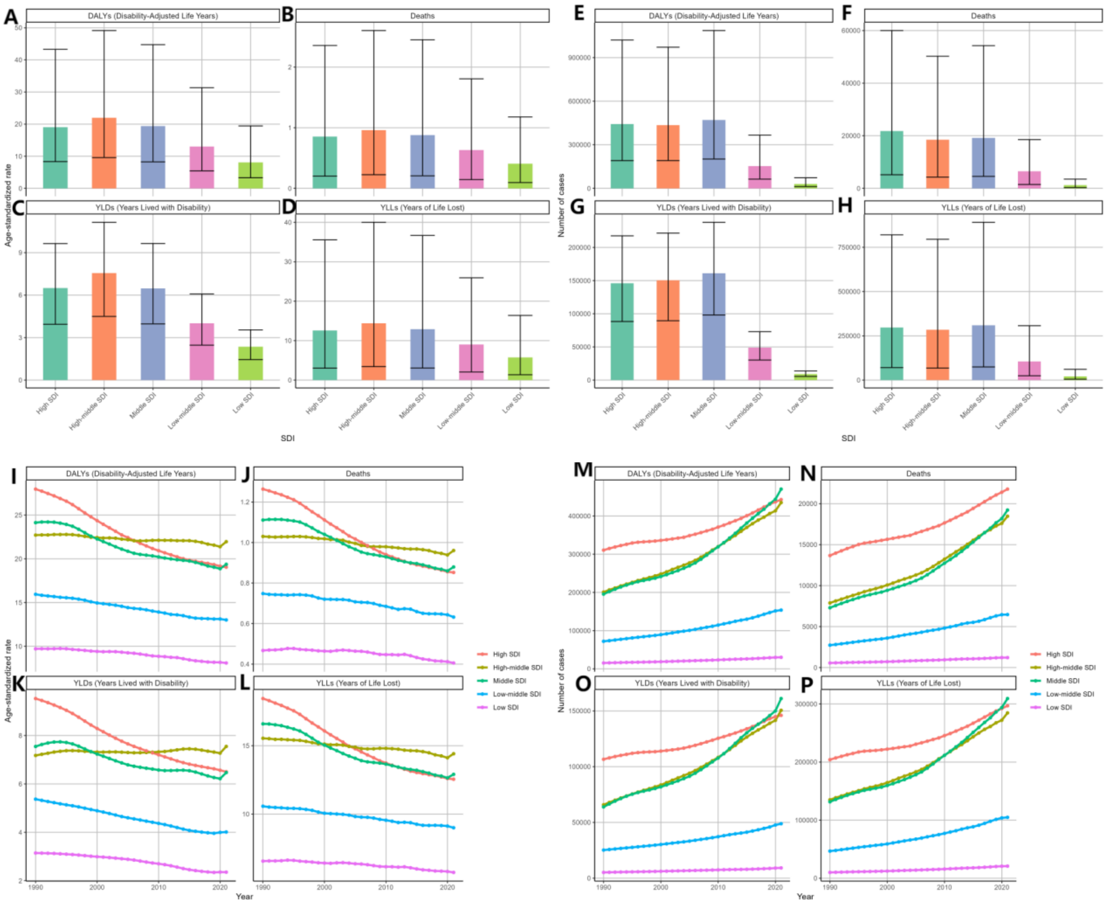

Supplementary Figure S3. Global burden and evolving trends of smoking-related Alzheimer's disease and other dementias across various socio-demographic indices from 1990 to 2021.(A, I) ASDAR.(B, J) ASDR.(C, K) age-standardized YLDs rate.(D, L) age-standardized YLLs rate.(E,M) Number of DALY cases.(F, N) Number of death cases.(G, O)Number of YLD cases.YLL cases are quantified by the number of life years lost. Key metrics include DALY (disability-adjusted life years), ASDR (age-standardized death rate), ASDAR (age-standardized DALYs rate), YLLs (years of life lost), and YLDs (years lived with disability).

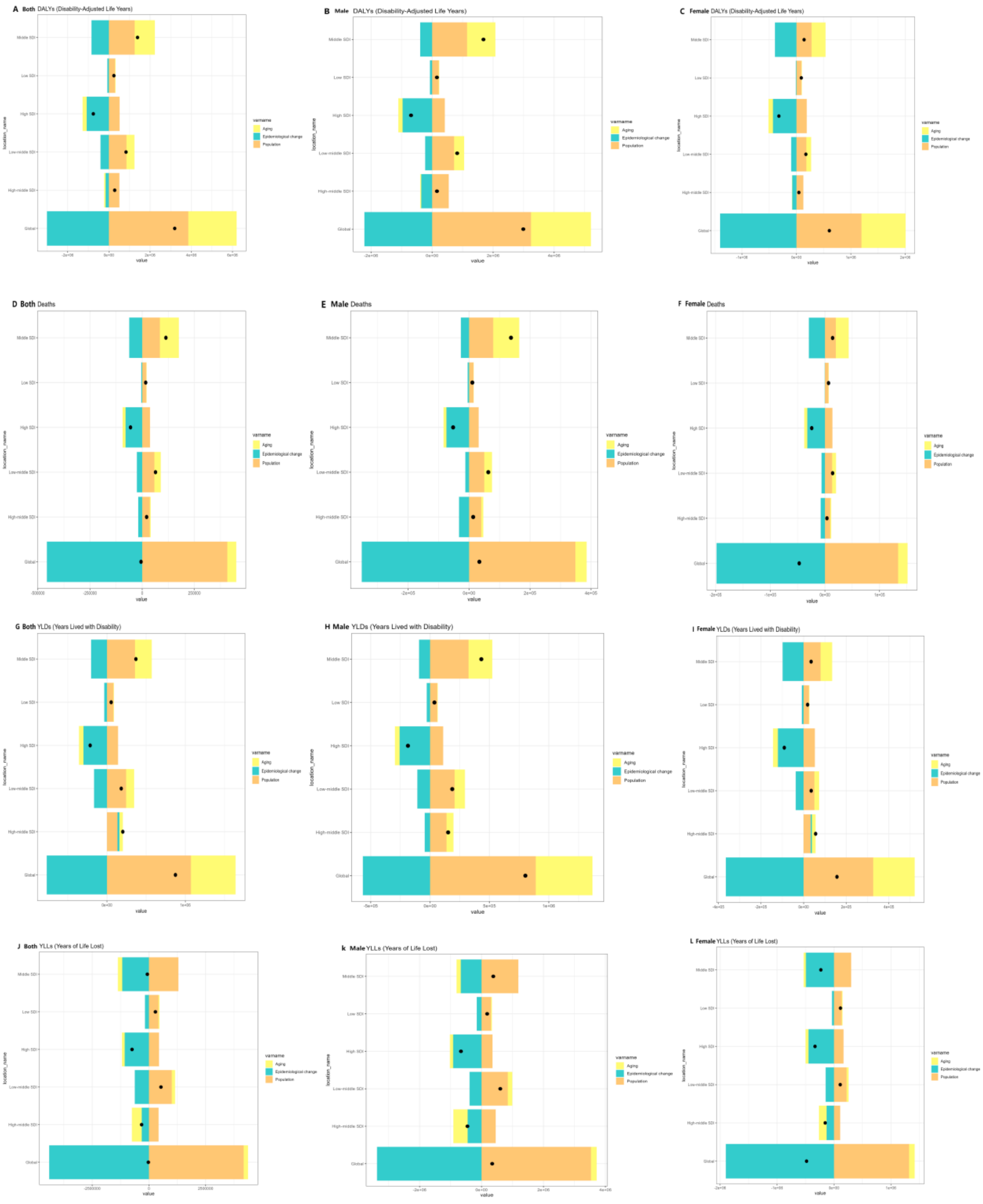

Supplementary Figure S4. An analysis of global population-level factors influencing changes in deaths, DALYs, YLD, and YLLs due to smoking-related Alzheimer's disease and other dementias, categorized by SDI levels and gender from 1990 to 2021. Variations in mortality rates based on global population determinants, categorized by SDI for (A) both genders, (B) males, and (C) females. Variations in DALYs based on global population determinants, categorized by SDI for (D) both genders, (E) males, and (F) females. Variations in DALYs based on global population determinants, stratified by SDI, are analyzed for (G) both genders, (H) males, and (I) females. Additionally, these variations are examined for (J) both genders, (K) males, and (L) females. The global determinants at the population level consist of three components: ageing (yellow),

epidemiological change (Tiffany blue), and population (orange). A positive value's magnitude signifies an increase in indicators of smoking-related Alzheimer's disease and other dementias attributed to the component, whereas a negative value's magnitude indicates a decrease in the related component's indicators. The black dot signifies the cumulative change value from all three components.SDI stands for socio-demographic index, DALY refers to disability-adjusted life years, YLLs denote years of life lost, and YLDs represent years lived with disability.

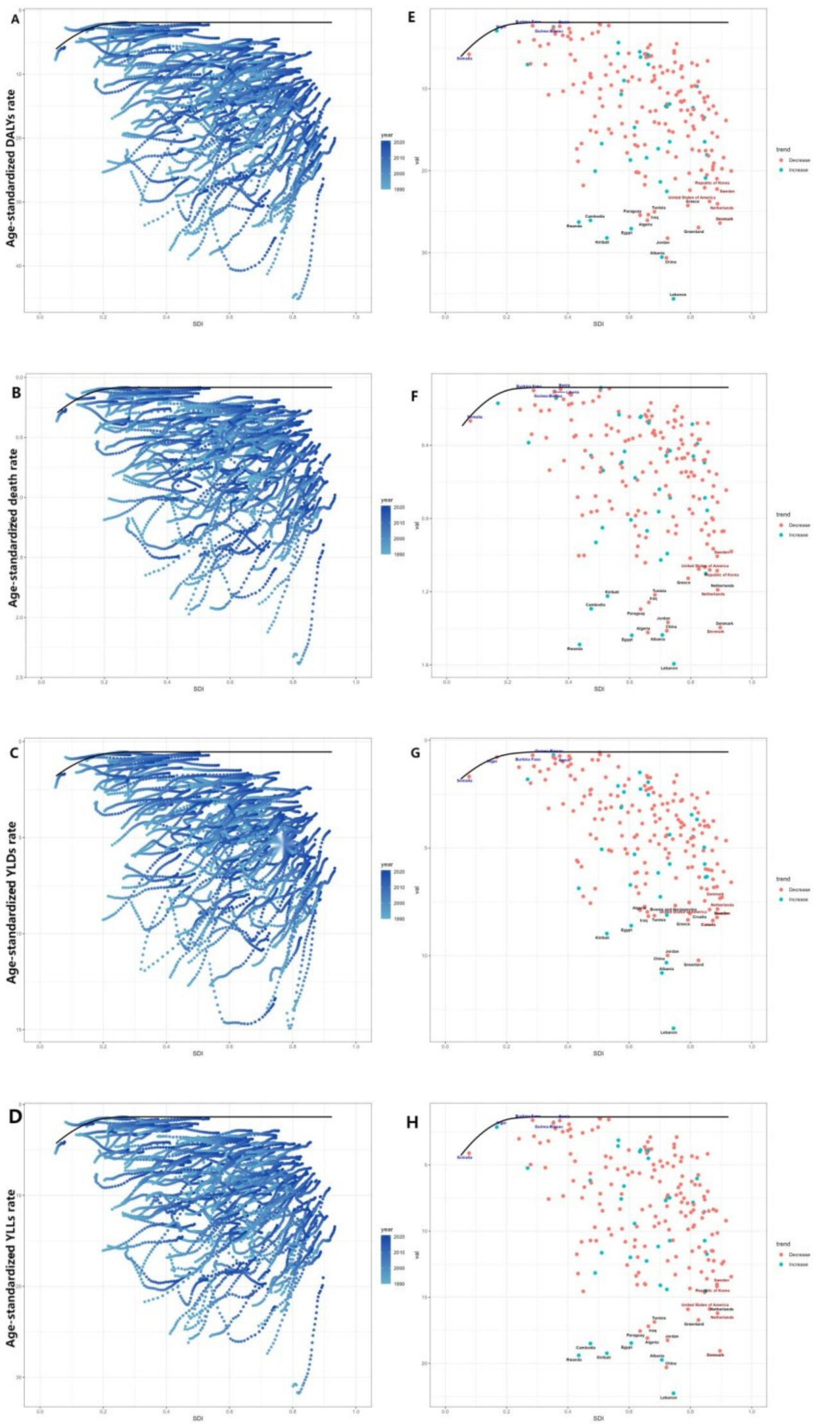

Supplementary Figure S5. The trends of (A) ASDAR, (B) ASDR, (C) age-standardized YLDs rate, and (D) age-standardized YLLs rate from 1990 to 2021 are analyzed based on SDI and country data. Dots represent countries, with the black line indicating the frontier. In 1990, frontier analysis shows orange dots for decreased ASDR, ASDAR, age-standardized YLDs rate, and age-standardized YLLs rate, while tiffany blue dots indicate increases in these metrics by 2021.The frontiers

were outlined in black, and the spaces between the dots and the frontier were evaluated as significant differences. The 15 countries with the greatest effective differences were highlighted in black. The five countries with low SDI levels and minimal effective differences were indicated in blue, while the five countries with high SDI levels and significant effective differences were marked in red. SDI stands for socio-demographic index, DALY refers to disability-adjusted life years, ASDR is the age-standardized death rate, ASDAR denotes the age-standardized DALYs rate, YLLs represent years of life lost, and YLDs indicate years lived with disability.

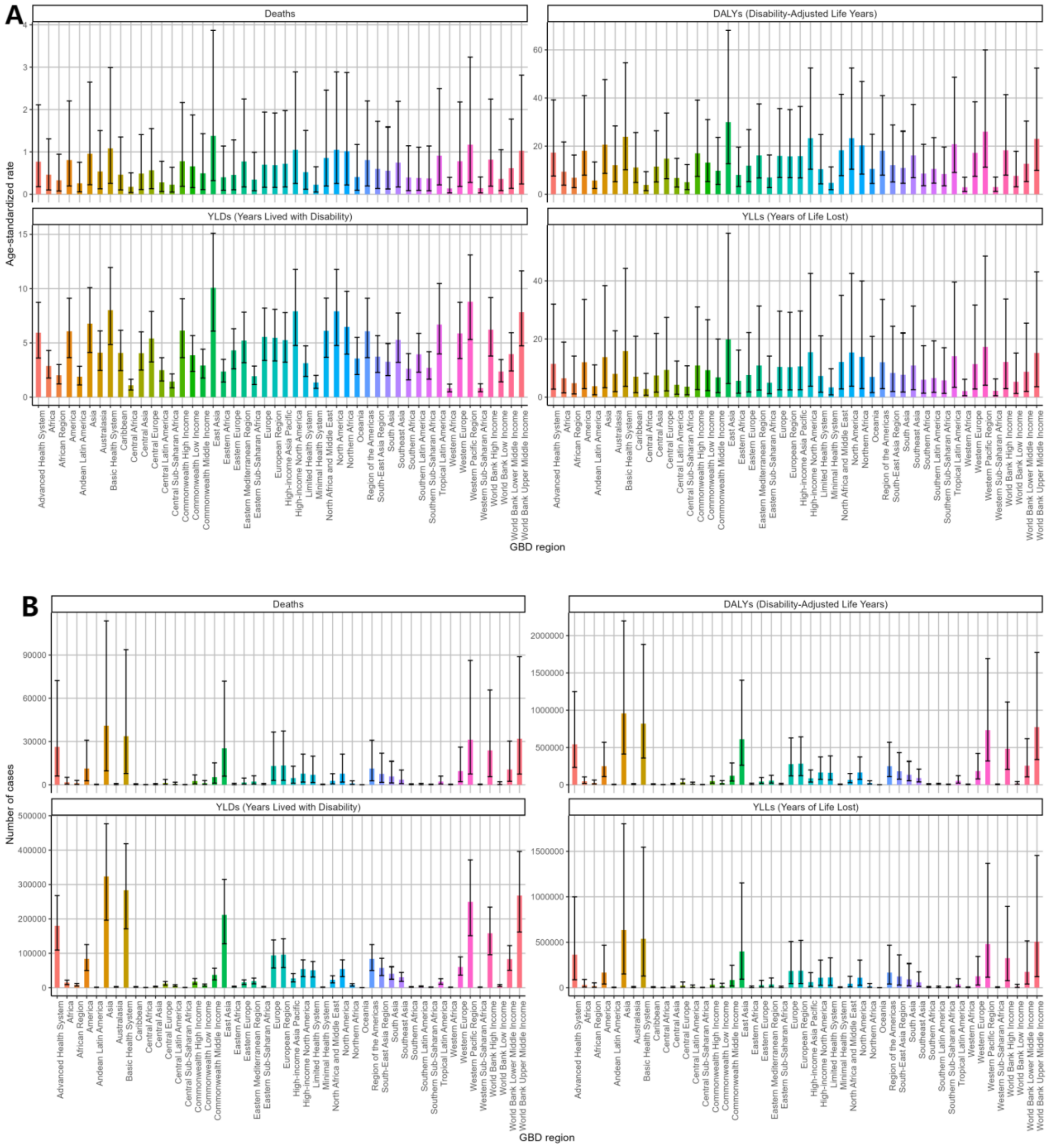

Supplementary Figure S6. In 2021, global burden indicators for smoking-related Alzheimer's disease and other dementias were analyzed according to GBD regions.(A) the ASR of deaths, DALY, YLDs, and YLLs.(B) Number of DALYs, deaths, DALY, YLDs, and YLLs cases.DALY refers to disability-adjusted life years, ASR denotes the age-standardized rate, YLLs represent years of life lost, and YLDs indicate years lived with disability.

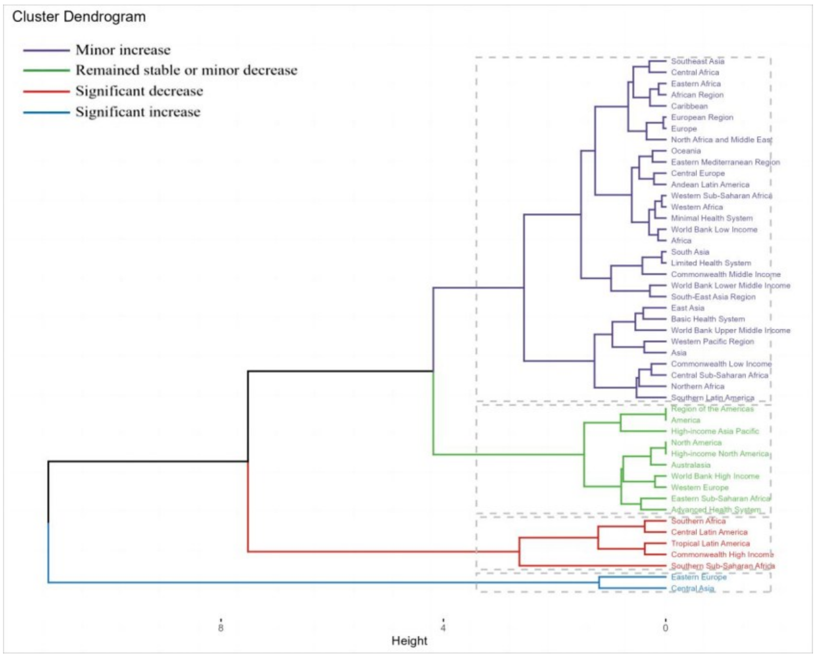

Supplementary Figure S7. Trends in the burden of smoking-related Alzheimer's disease and other dementias across GBD regions from 1990 to 2021.

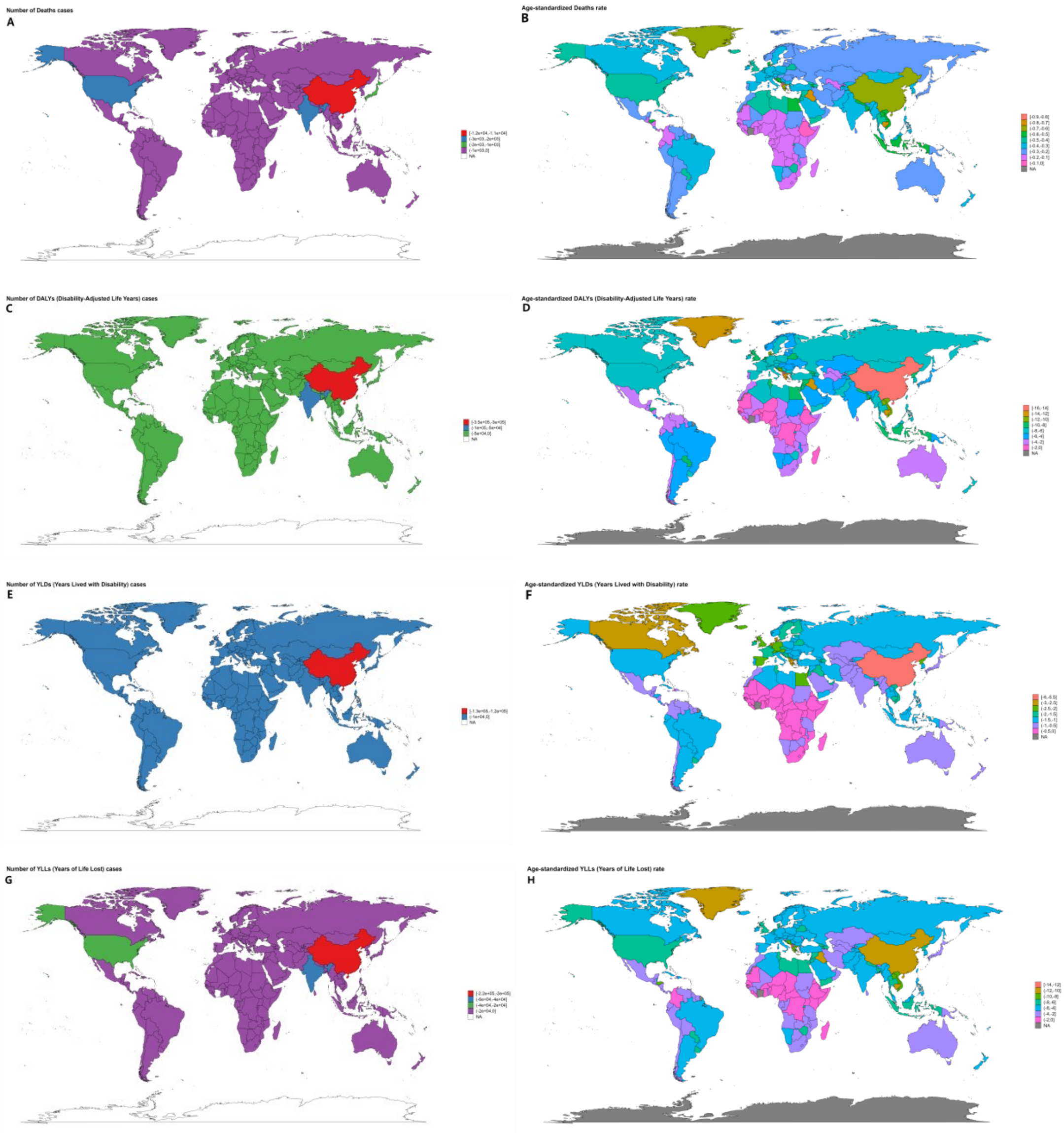

Supplementary Figure S8. The 2021 summary map illustrates the burden of smoking-related Alzheimer's disease and other dementias across 204 countries.(A)number of deaths.(B) age-standardized deaths.(C) number of DALYs.(D) age-standardized DALYs.(E) number of YLDs.(F) age-standardized of YLDs.(G)number of YLLs.Age-standardized metrics include YLLs (Years of Life Lost), DALY (Disability-Adjusted Life Years), ASDR(Age-Standardized Death Rate), and ASDAR (Age-Standardized DALYs Rate).

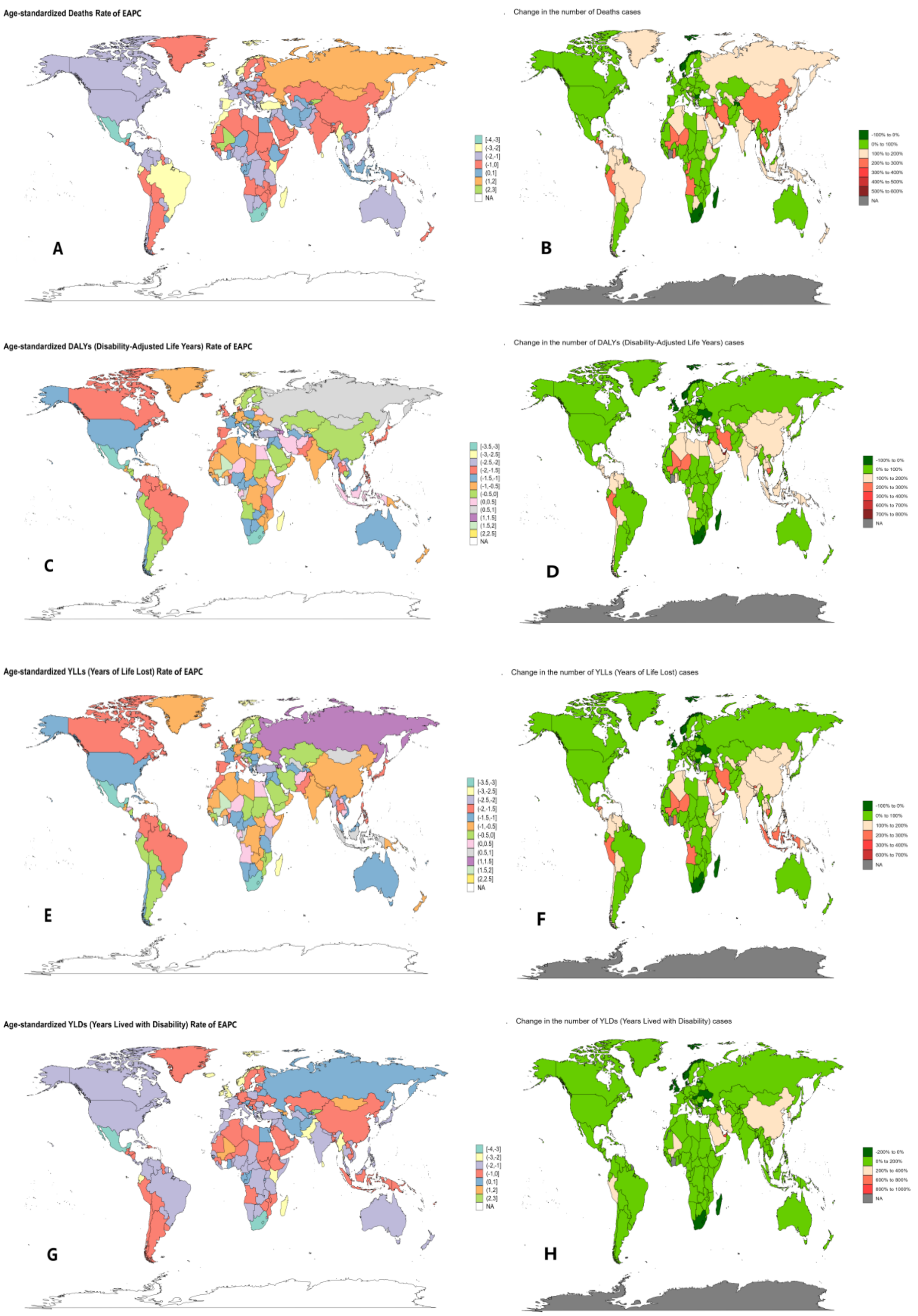

Supplementary Figure S9. The map illustrates changes in smoking-related Alzheimer's disease and other dementias across 204 countries, detailing: (A) EAPC for ASDR, (B) changes in death counts, (C) EAPC for ASDAR, (D) changes in DALYs, (E) EAPC for age-standardized YLLs rate, (F) changes in YLLs, (G) EAPC for age-standardized YLDs rate, and (H) changes in YLDs.EAPC stands for estimated annual percentage change; DALY refers to disability-adjusted life years; YLLs denote life lost years; YLDs indicate years lived with a disability; ASDR is the age-standardized death rate; and ASDAR represents the age-standardized DALYs rate.

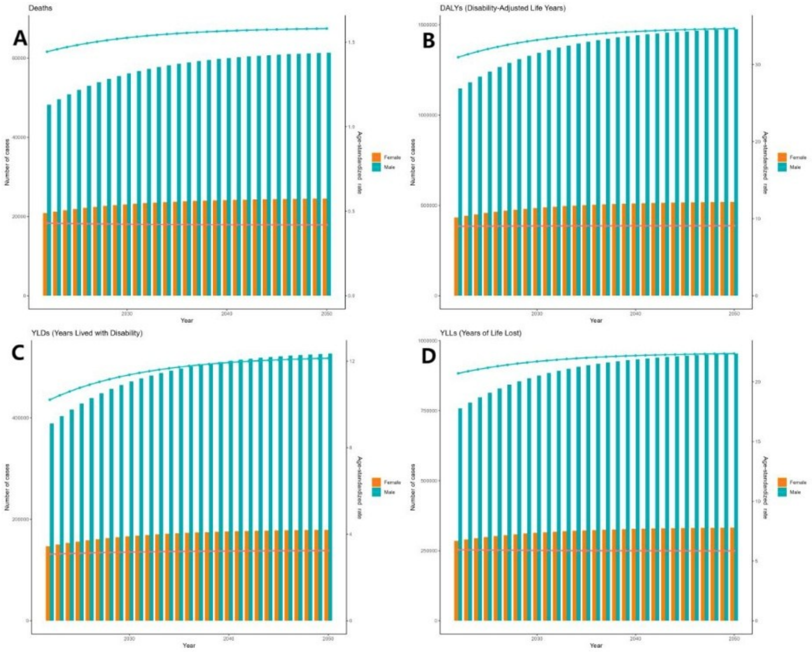

Supplementary Figure S10. Forecasting the burden of smoking-related Alzheimer's disease and other dementias from 2020 to 2050 using the ARIMA model.The ARIMA model forecasts (A) ASDR and death cases, (B) ASDAR and DALYs, (C) ASR and YLDs, and (D) ASR and YLLs.DALY stands for disability-adjusted life years; ASDR refers to age-standardized death rate; ASDAR denotes age-standardized DALYs rate; ASR is the abbreviation for age-standardized rate; YLLs represent years of life lost; YLDs indicate years lived with disability.

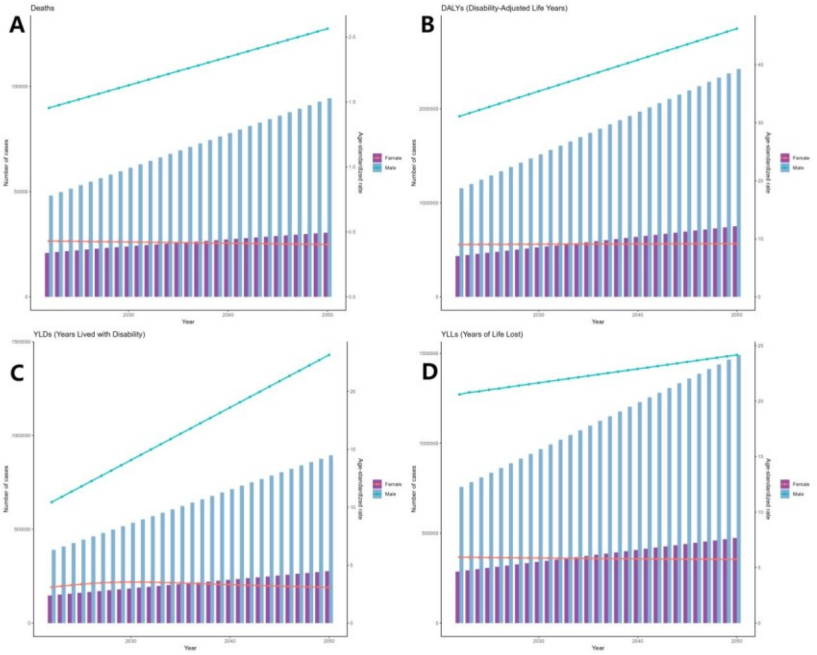

Supplementary Figure S11. The exponential smoothing (ES) model forecasts the burden of smoking-related Alzheimer's disease and other dementias from 2020 to 2050.The ES model predicts: (A) ASDR and death cases, (B) ASDAR and DALYs, (C) ASR and YLDs, and (D) ASR and YLLs.DALY stands for disability-adjusted life years; ASDR refers to age-standardized death rate; ASDAR denotes age-standardized DALYs rate; ASR is the abbreviation for age-standardized rate; YLLs represent years of life lost; YLDs indicate years lived with disability.

Supplementary Table S1 The number of death cases and the ASDR of smoking-attributable burden of ADOD stratified by sex, age , SDI region, and country with EAPC from 1990 to 2021 globally.

location	1990		2021		
	Number_95%UI	ASR	Number_95%UI	ASR	EAPC_95%CI
**Global**	32165 (7446-89320)	1.08 (0.25-3.03)	67176 (15695-184665)	0.84 (0.19-2.29)	0.04 (-0.45-0.53)
**Sex**					
Female	11665 (2789-31824)	0.67 (0.16-1.83)	20420 (4946-53998)	0.43 (0.1-1.14)	-1.6 (-1.68--1.52)
Male	20500 (4680-57870)	1.78 (0.4-5.14)	46755 (11061-132714)	1.43 (0.34-4.05)	-0.79 (-0.85--0.73)
**Age**					
40-44 years	12 (1-40)	0 (0-0.01)	13 (1-48)	0 (0-0.01)	-1.38 (-1.44--1.33)
45-49 years	94 (13-315)	0.04 (0.01-0.14)	136 (18-458)	0.03 (0-0.1)	-1.15 (-1.21--1.09)
50-54 years	396 (64-1244)	0.19 (0.03-0.59)	633 (97-1991)	0.14 (0.02-0.45)	-0.88 (-0.91--0.85)
55-59 years	1048 (179-3206)	0.57 (0.1-1.73)	1798 (302-5770)	0.45 (0.08-1.46)	-0.65 (-0.71--0.59)
60-64 years	2167 (421-6199)	1.35 (0.26-3.86)	3307 (644-10066)	1.03 (0.2-3.15)	-0.75 (-0.84--0.67)
65-69 years	3339 (717-10122)	2.7 (0.58-8.19)	5747 (1196-16463)	2.08 (0.43-5.97)	-0.83 (-0.95--0.72)
70-74 years	3922 (866-11408)	4.63 (1.02-13.47)	7333 (1669-22341)	3.56 (0.81-10.85)	-0.9 (-1.01--0.8)
75-79 years	4908 (1122-14065)	7.97 (1.82-22.85)	8691 (2048-24218)	6.59 (1.55-18.36)	-0.74 (-0.79--0.69)
80-84 years	6556 (1524-1786	18.53 (4.31-50.5)	13134 (3128-3669	15 (3.57-41.8	-0.83 (-0.88--0.

	5)		2)	9)	77)
85-89 years	5680 (1321-15540)	37.59 (8.74-102.84)	13614 (3189-36465)	29.78 (6.97-79.75)	-0.96 (-1.02--0.9)
90-94 years	2955 (683-7998)	68.96 (15.94-186.64)	8987 (2116-23600)	50.24 (11.83-131.92)	-1.18 (-1.22--1.13)
95+ years	1090 (255-2874)	107.03 (25.08-282.26)	3783 (927-9942)	69.41 (17-182.41)	-1.56 (-1.63--1.49)
**Regional levels**					
High-middle SDI	7881 (1768-22010)	1.03 (0.23-2.89)	18463 (4279-50254)	0.96 (0.22-2.6)	-0.31 (-0.33--0.28)
High SDI	13666 (3193-38023)	1.26 (0.29-3.55)	21771 (5160-60012)	0.85 (0.2-2.36)	-1.41 (-1.47--1.35)
Low-middle SDI	2734 (626-7836)	0.75 (0.17-2.14)	6466 (1473-18527)	0.63 (0.14-1.81)	-0.55 (-0.59--0.51)
Low SDI	560 (131-1626)	0.47 (0.11-1.36)	1216 (287-3511)	0.41 (0.1-1.18)	-0.46 (-0.54--0.39)
Middle SDI	7296 (1667-20480)	1.11 (0.26-3.14)	19215 (4536-54276)	0.88 (0.21-2.45)	-0.95 (-1.01--0.89)
World Bank High Income	15719 (3675-43690)	1.24 (0.29-3.5)	23911 (5627-65701)	0.82 (0.19-2.25)	-0.44 (-1.23-0.35)
World Bank Low Income	373 (88-1077)	0.48 (0.11-1.37)	727 (171-2102)	0.36 (0.09-1.05)	-0.59 (-2.44-1.29)
World Bank Lower Middle Income	4413 (1013-12561)	0.7 (0.16-2.02)	10560 (2396-30287)	0.61 (0.14-1.77)	0.28 (-0.97-1.55)
World Bank Upper Middle Income	11631 (2621-32473)	1.14 (0.26-3.18)	31932 (7452-88803)	1.03 (0.24-2.81)	0.99 (0.43-1.56)
Advanced Health System	17118 (3998-47532)	1.12 (0.26-3.14)	26263 (6087-72285)	0.77 (0.18-2.11)	-0.27 (-0.96-0.42)
Africa	931 (217-2606)	0.57 (0.13-1.67)	1782 (399-5191)	0.46 (0.1-1.31)	-0.63 (-2.39-1.16)

African Region	579 (136-1640)	0.46 (0.11-1.32)	1029 (235-3027)	0.33 (0.08-0.94)	-1.02 (-2.82-0.82)
America	7171 (1664-20019)	1.26 (0.29-3.51)	11353 (2727-30947)	0.81 (0.2-2.2)	-0.72 (-0.96--0.49)
Andean Latin America	53 (12-158)	0.32 (0.07-0.97)	141 (31-417)	0.26 (0.06-0.75)	0.38 (-0.36-1.12)
Asia	14612 (3340-41008)	1.15 (0.27-3.24)	40890 (9619-113621)	0.95 (0.22-2.65)	0.66 (-0.14-1.46)
Australasia	173 (40-485)	0.77 (0.17-2.16)	337 (80-937)	0.54 (0.13-1.5)	-0.13 (-0.75-0.5)
Basic Health System	12125 (2743-34082)	1.26 (0.29-3.53)	33752 (7852-93716)	1.08 (0.26-2.99)	0.81 (0.07-1.56)
Caribbean	131 (29-370)	0.61 (0.13-1.74)	258 (57-749)	0.46 (0.1-1.35)	-0.01 (-0.35-0.33)
Central Africa	38 (8-111)	0.24 (0.05-0.69)	69 (16-205)	0.17 (0.04-0.51)	-1.01 (-2.94-0.97)
Central Asia	161 (38-463)	0.42 (0.1-1.2)	300 (69-877)	0.49 (0.11-1.41)	1.15 (0.42-1.89)
Central Europe	947 (227-2674)	0.76 (0.18-2.21)	1350 (309-3691)	0.56 (0.13-1.55)	0.25 (-0.3-0.81)
Central Latin America	337 (76-983)	0.55 (0.12-1.61)	655 (147-1826)	0.28 (0.06-0.78)	-0.86 (-1.68--0.03)
Central Sub-Saharan Africa	31 (7-94)	0.27 (0.06-0.79)	72 (16-219)	0.22 (0.05-0.64)	-0.35 (-2.35-1.7)
Commonwealth High Income	2016 (466-5856)	1.34 (0.31-3.89)	2502 (589-6869)	0.78 (0.19-2.17)	-1.09 (-1.86--0.32)
Commonwealth Low Income	396 (89-1139)	0.72 (0.16-2.05)	1029 (247-2951)	0.66 (0.15-1.87)	0.36 (-1.33-2.08)
Commonwealth Middle Income	2163 (482-6214)	0.62 (0.14-1.76)	5239 (1163-1520	0.49 (0.11-1.43	0.12 (-1.21-1.

			3)	)	46)
East Asia	8275 (1858-22998)	1.67 (0.37-4.69)	25333 (5967-71869)	1.38 (0.32-3.87)	1.13 (0.42-1.84)
Eastern Africa	192 (46-544)	0.54 (0.13-1.53)	379 (89-1084)	0.4 (0.09-1.14)	-0.77 (-2.8-1.3)
Eastern Europe	993 (226-2831)	0.42 (0.09-1.21)	1647 (378-4664)	0.45 (0.1-1.28)	1.23 (0.77-1.69)
Eastern Mediterranean Region	1084 (249-3024)	0.96 (0.22-2.68)	2185 (491-6257)	0.77 (0.17-2.25)	-0.36 (-1.85-1.15)
Eastern Sub-Saharan Africa	181 (43-535)	0.48 (0.11-1.37)	340 (81-973)	0.35 (0.08-0.99)	-0.96 (-3.05-1.18)
Europe	9406 (2181-26677)	0.98 (0.23-2.77)	13068 (3027-36594)	0.69 (0.16-1.94)	-0.17 (-0.86-0.52)
European Region	9544 (2214-27071)	0.97 (0.22-2.73)	13310 (3079-37267)	0.69 (0.16-1.92)	-0.2 (-0.81-0.42)
High-income Asia Pacific	1982 (465-5542)	1.19 (0.27-3.29)	4738 (1133-12873)	0.72 (0.17-1.97)	0.33 (-0.69-1.36)
High-income North America	5457 (1298-15099)	1.48 (0.35-4.09)	7774 (1888-21272)	1.05 (0.25-2.89)	-0.59 (-1.24-0.06)
Limited Health System	2794 (632-7971)	0.63 (0.14-1.8)	6954 (1569-19851)	0.52 (0.12-1.51)	0.2 (-1.23-1.65)
Minimal Health System	99 (23-287)	0.29 (0.07-0.82)	161 (38-468)	0.22 (0.05-0.65)	-0.99 (-2.93-1)
North Africa and Middle East	1184 (273-3295)	1.12 (0.25-3.26)	2664 (623-7705)	0.85 (0.19-2.46)	-0.13 (-1.4-1.17)
North America	5457 (1298-15099)	1.48 (0.35-4.09)	7774 (1888-21272)	1.05 (0.25-2.89)	-0.59 (-1.24-0.06)
Northern Africa	403 (93-1166)	1.11 (0.25-3.25)	941 (202-2726)	1.02 (0.22-2.87)	0.34 (-1.04-1.74)
Oceania	9 (2-26)	0.51	20 (4-60)	0.41	-0.39

		(0.11-1.42)		(0.09-1.17)	(-1.99-1.24)
Region of the Americas	7171 (1664-20019)	1.26 (0.29-3.51)	11353 (2727-30947)	0.81 (0.2-2.2)	-0.72 (-0.96--0.49)
South-East Asia Region	2717 (611-7824)	0.7 (0.15-2.03)	7610 (1733-21908)	0.6 (0.14-1.72)	0.65 (-0.58-1.9)
South Asia	2128 (473-6148)	0.67 (0.15-1.93)	5664 (1272-16142)	0.55 (0.13-1.59)	0.44 (-0.49-1.37)
Southeast Asia	1510 (344-4245)	0.93 (0.21-2.68)	3542 (832-10246)	0.75 (0.17-2.19)	0.12 (-0.93-1.19)
Southern Africa	212 (50-600)	0.78 (0.19-2.18)	239 (54-704)	0.39 (0.09-1.14)	-2.15 (-3.69--0.58)
Southern Latin America	185 (42-537)	0.44 (0.1-1.28)	351 (82-1017)	0.38 (0.09-1.11)	0.37 (0.14-0.61)
Southern Sub-Saharan Africa	168 (40-468)	0.88 (0.21-2.37)	151 (34-450)	0.37 (0.08-1.14)	-2.4 (-3.55--1.22)
Tropical Latin America	1028 (234-2945)	1.61 (0.36-4.56)	2220 (532-6035)	0.91 (0.22-2.49)	-0.5 (-1.17-0.18)
Western Africa	86 (19-246)	0.18 (0.04-0.51)	154 (36-452)	0.14 (0.03-0.4)	-0.93 (-2.69-0.86)
Western Europe	7135 (1659-20443)	1.21 (0.28-3.41)	9444 (2169-26106)	0.78 (0.18-2.18)	-0.55 (-1.54-0.45)
Western Pacific Region	10941 (2471-30561)	1.47 (0.34-4.14)	31308 (7498-86240)	1.17 (0.28-3.24)	0.88 (0.34-1.41)
Western Sub-Saharan Africa	98 (22-277)	0.18 (0.04-0.52)	175 (40-511)	0.14 (0.03-0.41)	-0.96 (-2.73-0.83)
**Country levels**					
Afghanistan	24 (5-67)	0.56 (0.12-1.59)	29 (6-81)	0.54 (0.11-1.49)	-1.19 (-3.01-0.67)
Albania	19 (4-57)	1.27 (0.3-3.73)	58 (13-168)	1.44 (0.31-4.2)	2.69 (2.09-3.2

					8)
Algeria	102 (23-297)	1.93 (0.41-6.01)	289 (59-835)	1.42 (0.3-4.15)	0.41 (-1.13-1.97)
American Samoa	0 (0-0)	0.57 (0.13-1.6)	0 (0-1)	0.49 (0.11-1.45)	1.01 (-0.21-2.23)
Andorra	0 (0-1)	1.07 (0.24-2.91)	1 (0-4)	0.8 (0.19-2.09)	0.43 (-0.2-1.06)
Angola	9 (2-26)	0.45 (0.1-1.28)	27 (6-79)	0.43 (0.09-1.21)	0.01 (-2.26-2.34)
Antigua and Barbuda	0 (0-0)	0.29 (0.07-0.84)	0 (0-1)	0.27 (0.06-0.78)	-0.31 (-0.62-0.01)
Argentina	141 (32-410)	0.48 (0.11-1.38)	255 (58-734)	0.44 (0.1-1.26)	0.43 (0.2-0.66)
Armenia	17 (4-46)	0.82 (0.19-2.27)	44 (10-127)	1.03 (0.24-2.95)	2.79 (2.29-3.3)
Australia	134 (31-375)	0.72 (0.16-2)	259 (60-714)	0.49 (0.11-1.36)	-0.2 (-0.84-0.45)
Austria	78 (18-225)	0.64 (0.14-1.83)	169 (38-467)	0.76 (0.17-2.07)	1.49 (0.52-2.46)
Azerbaijan	21 (5-60)	0.51 (0.11-1.49)	54 (12-159)	0.76 (0.17-2.21)	2.07 (1.34-2.8)
Bahamas	0 (0-1)	0.28 (0.06-0.83)	1 (0-3)	0.28 (0.06-0.81)	1.03 (0.35-1.72)
Bahrain	1 (0-2)	1.05 (0.22-3.16)	3 (1-9)	0.84 (0.19-2.42)	-0.4 (-2.8-2.07)
Bangladesh	267 (59-774)	0.82 (0.18-2.32)	781 (187-2194)	0.76 (0.18-2.15)	1.05 (-0.42-2.53)
Barbados	1 (0-3)	0.3 (0.07-0.88)	1 (0-4)	0.26 (0.06-0.76)	-0.37 (-0.89-0.16)
Belarus	72 (16-202)	0.61	91 (21-269)	0.54	0.58

		(0.13-1.73)		(0.13-1.62)	(0.06-1.1)
Belgium	218 (49-648)	1.38 (0.31-4.08)	286 (67-782)	0.94 (0.22-2.58)	-0.38 (-1.38-0. 62)
Belize	0 (0-1)	0.42 (0.1-1.21)	1 (0-3)	0.36 (0.08-1.02)	-0.07 (-1.07-0. 93)
Benin	3 (1-8)	0.16 (0.04-0.47)	4 (1-11)	0.09 (0.02-0.29)	-2.21 (-3.82--0. 57)
Bermuda	0 (0-1)	0.36 (0.08-1.06)	1 (0-2)	0.39 (0.09-1.06)	1.98 (1.36-2.6 1)
Bhutan	1 (0-2)	0.42 (0.08-1.24)	2 (1-6)	0.46 (0.1-1.28)	2.31 (0.89-3.7 4)
Bolivia (Plurinational State of)	7 (2-20)	0.3 (0.06-0.86)	17 (4-49)	0.24 (0.05-0.69)	0.56 (-0.52-1. 66)
Bosnia and Herzegovina	28 (7-77)	0.96 (0.23-2.63)	62 (14-172)	0.99 (0.22-2.75)	1.88 (1.3-2.45)
Botswana	2 (1-7)	0.78 (0.16-2.38)	5 (1-14)	0.55 (0.12-1.62)	-0.31 (-1.94-1. 35)
Brazil	992 (226-2842)	1.59 (0.36-4.51)	2153 (516-5881)	0.9 (0.22-2.47)	-0.48 (-1.15-0. 19)
Brunei Darussalam	1 (0-2)	1 (0.22-3.11)	1 (0-3)	0.57 (0.12-1.69)	-0.92 (-2.54-0. 73)
Bulgaria	56 (13-161)	0.57 (0.13-1.72)	54 (12-159)	0.37 (0.08-1.09)	-0.49 (-1.22-0. 24)
Burkina Faso	4 (1-10)	0.13 (0.03-0.38)	6 (1-19)	0.1 (0.02-0.29)	-0.86 (-2.54-0. 85)
Burundi	5 (1-13)	0.33 (0.07-0.95)	5 (1-15)	0.18 (0.04-0.54)	-2.08 (-4.04--0. 08)
Cabo Verde	0 (0-1)	0.16 (0.04-0.5)	0 (0-1)	0.09 (0.02-0.27)	-1.96 (-2.48--1. 43)

Cambodia	29 (7-86)	1.18 (0.26-3.45)	96 (22-277)	1.29 (0.28-3.81)	1.32 (-0.29-2.96)
Cameroon	6 (1-17)	0.22 (0.04-0.6)	12 (2-34)	0.16 (0.03-0.47)	-1.25 (-3.19-0.74)
Canada	403 (90-1145)	1.29 (0.28-3.63)	683 (162-1838)	0.81 (0.19-2.17)	-0.49 (-1.2-0.22)
Central African Republic	2 (0-5)	0.31 (0.06-0.94)	2 (0-7)	0.21 (0.04-0.62)	-1.46 (-3.7-0.84)
Chad	5 (1-14)	0.23 (0.05-0.69)	7 (1-21)	0.21 (0.04-0.59)	-1.36 (-3.18-0.5)
Chile	27 (6-76)	0.32 (0.07-0.87)	65 (15-191)	0.25 (0.06-0.72)	0.22 (-0.1-0.55)
China	8126 (1822-22578)	1.72 (0.39-4.84)	24897 (5867-70648)	1.41 (0.33-3.96)	1.13 (0.41-1.85)
Colombia	52 (12-154)	0.38 (0.09-1.12)	143 (34-382)	0.24 (0.06-0.66)	0.19 (-0.47-0.85)
Comoros	1 (0-2)	0.51 (0.11-1.51)	1 (0-4)	0.47 (0.1-1.32)	0.69 (-0.93-2.35)
Congo	2 (0-6)	0.35 (0.08-1.02)	6 (1-18)	0.43 (0.1-1.21)	1.03 (-0.87-2.98)
Cook Islands	0 (0-0)	0.62 (0.14-1.83)	0 (0-0)	0.47 (0.1-1.46)	0.83 (0.27-1.38)
Costa Rica	10 (2-27)	0.63 (0.15-1.74)	24 (5-67)	0.42 (0.09-1.17)	0.17 (-0.38-0.73)
Cmte d'Ivoire	5 (1-15)	0.25 (0.05-0.75)	12 (3-37)	0.19 (0.04-0.56)	-0.85 (-3.01-1.36)
Croatia	58 (13-158)	1.21 (0.26-3.24)	102 (23-284)	1.02 (0.23-2.82)	1.39 (0.71-2.07)
Cuba	66 (14-192)	0.73 (0.15-2.14)	107 (23-310)	0.5 (0.11-1.46	-0.14 (-0.62-0.

				)	35)
Cyprus	6 (2-18)	1.12 (0.25-3.23)	17 (4-47)	0.86 (0.2-2.34)	0.05 (-0.25-0.35)
Czechia	105 (24-292)	0.83 (0.18-2.24)	163 (38-459)	0.69 (0.16-1.95)	0.4 (-0.28-1.08)
Democratic People's Republic of Korea	66 (14-186)	0.63 (0.14-1.8)	140 (32-423)	0.5 (0.12-1.49)	0.32 (-0.29-0.94)
Democratic Republic of the Congo	17 (4-54)	0.21 (0.04-0.67)	34 (7-98)	0.15 (0.03-0.42)	-0.83 (-2.76-1.13)
Denmark	205 (47-579)	2.33 (0.53-6.49)	197 (46-562)	1.4 (0.33-3.98)	-1.35 (-2.29--0.41)
Djibouti	0 (0-1)	0.74 (0.15-2.12)	2 (0-6)	0.68 (0.14-1.87)	0.73 (-1.61-3.13)
Dominica	0 (0-0)	0.25 (0.05-0.71)	0 (0-0)	0.21 (0.05-0.62)	0.08 (-0.14-0.29)
Dominican Republic	26 (6-79)	1.03 (0.23-3.03)	73 (16-212)	0.78 (0.17-2.25)	0.44 (-0.35-1.24)
Ecuador	18 (4-52)	0.43 (0.09-1.24)	31 (7-94)	0.21 (0.04-0.62)	-1.13 (-1.88--0.38)
Egypt	168 (39-487)	1.36 (0.3-4.01)	395 (85-1138)	1.44 (0.33-4.13)	0.49 (-1.42-2.44)
El Salvador	6 (1-17)	0.22 (0.05-0.63)	15 (3-41)	0.22 (0.05-0.62)	1.29 (0.8-1.78)
Equatorial Guinea	0 (0-1)	0.3 (0.06-0.92)	1 (0-2)	0.27 (0.06-0.78)	-0.6 (-2.68-1.53)
Eritrea	1 (0-2)	0.13 (0.03-0.38)	2 (0-5)	0.12 (0.02-0.36)	0.19 (-2.07-2.5)
Estonia	8 (2-23)	0.42 (0.09-1.21)	16 (4-43)	0.49 (0.11-1.35)	1.74 (0.95-2.55)
Eswatini	1 (0-3)	0.56	1 (0-3)	0.38	-0.97

		(0.12-1.72)		(0.07-1.13)	(-3.18-1.29)
Ethiopia	18 (4-52)	0.14 (0.03-0.41)	43 (10-134)	0.14 (0.03-0.43)	0.86 (-1.03-2.79)
Fiji	1 (0-3)	0.46 (0.1-1.35)	2 (0-5)	0.33 (0.07-0.98)	0.04 (-1.14-1.25)
Finland	43 (11-127)	0.61 (0.15-1.81)	85 (19-235)	0.54 (0.12-1.48)	0.98 (0.01-1.96)
France	773 (174-2163)	0.85 (0.19-2.39)	1101 (264-3051)	0.59 (0.14-1.63)	-0.4 (-1.39-0.61)
Gabon	1 (0-3)	0.24 (0.06-0.65)	2 (0-5)	0.25 (0.05-0.69)	-0.16 (-1.48-1.16)
Gambia	1 (0-2)	0.28 (0.06-0.8)	1 (0-3)	0.17 (0.04-0.48)	-1.36 (-3.07-0.38)
Georgia	26 (6-80)	0.47 (0.11-1.44)	47 (11-139)	0.69 (0.15-2.05)	2.82 (2.17-3.48)
Germany	1540 (365-4156)	1.17 (0.28-3.16)	2199 (555-6098)	0.87 (0.22-2.42)	-0.07 (-1.14-1.01)
Ghana	7 (2-20)	0.23 (0.05-0.67)	21 (5-63)	0.23 (0.05-0.7)	0.98 (-0.98-2.97)
Greece	220 (48-612)	1.59 (0.36-4.48)	383 (87-1072)	1.13 (0.26-3.16)	0.41 (-0.57-1.39)
Greenland	0 (0-1)	1.33 (0.31-3.87)	1 (0-1)	1.08 (0.24-3.08)	0.8 (-0.18-1.79)
Grenada	0 (0-1)	0.25 (0.06-0.76)	0 (0-0)	0.17 (0.04-0.51)	-1.6 (-1.98--1.22)
Guam	0 (0-1)	0.38 (0.09-1.11)	1 (0-2)	0.34 (0.07-0.96)	2.09 (1.35-2.83)
Guatemala	8 (2-25)	0.4 (0.09-1.21)	28 (6-85)	0.31 (0.07-0.92)	0.65 (-0.84-2.15)

Guinea	6 (1-16)	0.24 (0.05-0.67)	8 (2-24)	0.21 (0.04-0.6)	-0.9 (-2.23-0.44)
Guinea-Bissau	0 (0-1)	0.1 (0.02-0.3)	0 (0-1)	0.11 (0.02-0.33)	0.24 (-1.83-2.35)
Guyana	1 (0-2)	0.28 (0.06-0.8)	1 (0-3)	0.23 (0.05-0.7)	0.54 (-0.58-1.66)
Haiti	4 (1-12)	0.2 (0.04-0.58)	7 (2-21)	0.17 (0.04-0.47)	-0.34 (-1.85-1.19)
Honduras	8 (2-25)	0.53 (0.12-1.57)	25 (5-73)	0.54 (0.12-1.56)	0.62 (-0.68-1.93)
Hungary	86 (21-250)	0.64 (0.15-1.86)	96 (21-269)	0.45 (0.1-1.25)	-0.29 (-1.01-0.44)
Iceland	5 (1-13)	1.52 (0.35-4.2)	6 (1-16)	0.87 (0.21-2.33)	-1.27 (-1.85--0.69)
India	1469 (319-4366)	0.61 (0.13-1.81)	4321 (957-12557)	0.52 (0.12-1.53)	0.74 (-0.55-2.05)
Indonesia	387 (84-1148)	0.68 (0.15-1.98)	1156 (276-3447)	0.87 (0.2-2.55)	1.36 (0.03-2.7)
Iran (Islamic Republic of)	102 (24-289)	0.64 (0.15-1.79)	400 (88-1168)	0.64 (0.14-1.85)	1.96 (0.78-3.16)
Iraq	125 (28-341)	1.84 (0.41-4.99)	176 (38-516)	1.26 (0.28-3.59)	-1.36 (-2.86-0.16)
Ireland	75 (16-208)	2.12 (0.46-5.88)	84 (20-231)	0.97 (0.23-2.65)	-2.02 (-2.4--1.63)
Israel	45 (10-132)	1.06 (0.23-3.15)	97 (22-262)	0.68 (0.16-1.83)	-0.62 (-0.91--0.34)
Italy	1138 (263-3155)	1.33 (0.3-3.65)	1500 (334-3901)	0.77 (0.18-2.03)	-0.62 (-1.76-0.53)
Jamaica	8 (2-25)	0.44 (0.1-1.36)	12 (3-35)	0.35 (0.07-0.99	-0.24 (-0.44--0.

				)	03)
Japan	1686 (402-4781)	1.14 (0.26-3.19)	3728 (904-9970)	0.68 (0.16-1.85)	0.26 (-1-1.54)
Jordan	14 (3-40)	1.92 (0.44-5.35)	57 (14-154)	1.37 (0.32-3.66)	-0.44 (-2.39-1. 55)
Kazakhstan	33 (7-95)	0.32 (0.07-0.88)	39 (9-117)	0.28 (0.06-0.81)	-0.1 (-0.59-0. 4)
Kenya	26 (6-77)	0.52 (0.12-1.53)	39 (9-109)	0.29 (0.07-0.8)	-2.03 (-3.93--0. 1)
Kiribati	0 (0-1)	1.02 (0.23-3)	0 (0-1)	1.22 (0.27-3.53)	0.55 (-1.07-2. 21)
Kuwait	4 (1-11)	1.14 (0.25-3.16)	21 (5-59)	1.07 (0.26-3.03)	0.63 (-0.92-2. 22)
Kyrgyzstan	13 (3-37)	0.53 (0.12-1.53)	28 (6-81)	0.81 (0.17-2.29)	2.14 (1.2-3.09)
Lao People's Democratic Republic	9 (2-27)	0.82 (0.17-2.33)	26 (6-73)	0.93 (0.23-2.54)	1.09 (-0.51-2. 72)
Latvia	16 (3-47)	0.47 (0.1-1.36)	17 (4-50)	0.37 (0.08-1.1)	0.42 (-0.37-1. 21)
Lebanon	21 (4-61)	1.35 (0.29-3.81)	108 (27-299)	1.59 (0.39-4.44)	2.07 (1.51-2.6 3)
Lesotho	4 (1-12)	0.76 (0.15-2.24)	5 (1-13)	0.85 (0.18-2.39)	0.14 (-1.32-1. 63)
Liberia	1 (0-4)	0.15 (0.03-0.44)	2 (0-5)	0.11 (0.02-0.31)	-2.12 (-3.68--0. 54)
Libya	17 (4-47)	1.12 (0.26-3.06)	32 (7-96)	0.87 (0.19-2.58)	-0.09 (-1.21-1. 05)
Lithuania	26 (6-73)	0.6 (0.13-1.71)	30 (7-91)	0.44 (0.1-1.33)	0.22 (-0.53-0. 97)
Luxembourg	3 (1-10)	0.62	7 (2-20)	0.56	0.22

		(0.14-1.86)		(0.13-1.63)	(-0.43-0.87)
Madagascar	11 (3-33)	0.41 (0.09-1.25)	9 (2-26)	0.18 (0.04-0.51)	-3.34 (-5.56--1.06)
Malawi	12 (3-36)	0.6 (0.12-1.84)	22 (5-63)	0.48 (0.11-1.46)	-0.56 (-2.56-1.48)
Malaysia	81 (18-234)	1.09 (0.25-3.2)	156 (35-455)	0.79 (0.18-2.32)	-0.75 (-1.88-0.4)
Maldives	1 (0-2)	1.65 (0.36-4.9)	3 (1-7)	1.04 (0.25-2.96)	0.2 (-1.52-1.95)
Mali	5 (1-13)	0.23 (0.05-0.67)	17 (4-52)	0.39 (0.08-1.14)	1.77 (-0.39-3.97)
Malta	3 (1-9)	0.86 (0.19-2.41)	6 (1-17)	0.55 (0.13-1.48)	-0.31 (-1.02-0.41)
Marshall Islands	0 (0-0)	0.47 (0.1-1.37)	0 (0-0)	0.49 (0.1-1.52)	0.74 (-1.31-2.83)
Mauritania	1 (0-3)	0.16 (0.04-0.44)	2 (0-7)	0.13 (0.03-0.41)	-0.44 (-1.82-0.96)
Mauritius	3 (1-10)	0.65 (0.15-1.92)	10 (2-29)	0.62 (0.14-1.8)	1.65 (0.97-2.33)
Mexico	191 (42-550)	0.68 (0.15-1.94)	287 (61-800)	0.26 (0.06-0.73)	-1.74 (-2.67--0.8)
Micronesia (Federated States of)	0 (0-1)	0.7 (0.15-1.98)	0 (0-1)	0.62 (0.13-1.87)	0.14 (-1.22-1.51)
Monaco	1 (0-2)	1.01 (0.23-2.84)	1 (0-3)	0.87 (0.2-2.48)	-0.24 (-1.78-1.32)
Mongolia	4 (1-11)	0.46 (0.1-1.32)	9 (2-25)	0.58 (0.13-1.66)	1.35 (-0.03-2.74)
Montenegro	5 (1-14)	0.88 (0.2-2.46)	7 (2-19)	0.75 (0.17-2.16)	0.21 (-0.15-0.57)

Morocco	64 (14-180)	0.58 (0.13-1.68)	89 (18-252)	0.32 (0.07-0.91)	-1.18 (-2.03--0.32)
Mozambique	14 (3-42)	0.46 (0.1-1.37)	21 (5-62)	0.36 (0.08-1.03)	-1.02 (-3.03-1.03)
Myanmar	170 (38-484)	1.33 (0.3-3.79)	240 (53-729)	0.71 (0.16-2.08)	-1.14 (-2.29-0.02)
Namibia	3 (1-11)	1.14 (0.26-3.6)	6 (1-17)	0.84 (0.19-2.31)	-0.54 (-2.42-1.37)
Nauru	0 (0-0)	0.82 (0.19-2.37)	0 (0-0)	0.59 (0.12-1.69)	-0.69 (-2.47-1.12)
Nepal	59 (13-169)	1.19 (0.27-3.43)	150 (33-445)	1 (0.22-2.95)	0.51 (-0.95-1.99)
Netherlands	314 (76-907)	1.55 (0.37-4.46)	486 (109-1298)	1.19 (0.27-3.19)	0.47 (-0.32-1.28)
New Zealand	39 (9-111)	1.04 (0.24-2.95)	78 (18-215)	0.83 (0.2-2.27)	0.2 (-0.33-0.73)
Nicaragua	5 (1-13)	0.39 (0.09-1.16)	14 (3-40)	0.34 (0.07-0.98)	1.1 (-0.02-2.24)
Niger	2 (0-5)	0.14 (0.03-0.42)	6 (1-19)	0.17 (0.04-0.52)	0.41 (-2.1-2.98)
Nigeria	43 (9-125)	0.16 (0.03-0.45)	58 (13-173)	0.1 (0.02-0.29)	-1.87 (-3.59--0.13)
Niue	0 (0-0)	0.46 (0.1-1.31)	0 (0-0)	0.42 (0.1-1.18)	0.01 (-0.21-0.23)
North Macedonia	15 (3-43)	0.99 (0.21-2.8)	20 (5-59)	0.88 (0.19-2.4)	0.18 (-0.19-0.54)
Northern Mariana Islands	0 (0-0)	0.59 (0.14-1.71)	0 (0-0)	0.47 (0.11-1.38)	1.73 (-0.02-3.5)
Norway	96 (21-266)	1.25 (0.27-3.44)	79 (18-223)	0.65 (0.15-1.85	-2.3 (-3.26--1.

				)	33)
Oman	2 (0-7)	0.53 (0.11-1.5)	4 (1-12)	0.39 (0.09-1.07)	-0.75 (-2.67-1.21)
Pakistan	332 (72-948)	0.86 (0.19-2.44)	410 (94-1253)	0.55 (0.13-1.7)	-1.83 (-3.28--0.36)
Palau	0 (0-0)	0.52 (0.12-1.42)	0 (0-0)	0.47 (0.11-1.36)	0.56 (-0.36-1.48)
Palestine	8 (2-22)	1.2 (0.26-3.62)	14 (3-42)	0.89 (0.2-2.66)	-1.15 (-2.93-0.66)
Panama	7 (2-21)	0.58 (0.13-1.64)	17 (3-47)	0.36 (0.07-1)	-0.6 (-1.05--0.16)
Papua New Guinea	4 (1-13)	0.41 (0.09-1.12)	11 (2-33)	0.35 (0.08-1.05)	-0.4 (-2.11-1.34)
Paraguay	37 (8-105)	1.97 (0.45-5.55)	67 (14-178)	1.3 (0.28-3.47)	-0.69 (-1.41-0.04)
Peru	28 (6-84)	0.28 (0.06-0.84)	93 (20-267)	0.28 (0.06-0.79)	1.04 (0.4-1.68)
Philippines	206 (47-600)	1.34 (0.28-3.81)	429 (98-1274)	0.77 (0.17-2.31)	-0.8 (-2.19-0.61)
Poland	333 (78-967)	0.85 (0.19-2.54)	446 (107-1276)	0.57 (0.13-1.61)	-0.23 (-0.77-0.31)
Portugal	64 (14-194)	0.49 (0.11-1.47)	90 (21-259)	0.3 (0.07-0.84)	-0.5 (-1.41-0.41)
Puerto Rico	15 (3-42)	0.45 (0.1-1.24)	35 (8-98)	0.37 (0.09-1.06)	1.01 (0.25-1.78)
Qatar	0 (0-1)	0.63 (0.14-1.87)	2 (0-6)	0.48 (0.11-1.31)	-0.88 (-3.68-2)
Republic of Korea	289 (66-798)	1.89 (0.43-5.35)	986 (231-2770)	1.09 (0.25-3.05)	0.75 (-0.03-1.53)
Republic of	14 (3-39)	0.39	27 (6-75)	0.43	1.86

Moldova		(0.09-1.14)		(0.09-1.22)	(1.47-2.24)
Romania	121 (27-351)	0.57 (0.13-1.66)	151 (34-435)	0.37 (0.08-1.07)	-0.22 (-0.77-0.34)
Russian Federation	535 (119-1544)	0.35 (0.07-1)	1131 (263-3227)	0.46 (0.11-1.31)	1.91 (1.46-2.36)
Rwanda	15 (3-44)	1.18 (0.26-3.67)	48 (11-134)	1.49 (0.33-4.06)	1.42 (-0.87-3.76)
Saint Kitts and Nevis	0 (0-0)	0.23 (0.05-0.69)	0 (0-0)	0.19 (0.04-0.55)	-0.79 (-1.11--0.47)
Saint Lucia	0 (0-1)	0.32 (0.07-1)	1 (0-2)	0.24 (0.05-0.73)	0.44 (-0.06-0.95)
Saint Vincent and the Grenadines	0 (0-0)	0.23 (0.05-0.68)	0 (0-1)	0.24 (0.05-0.7)	1.18 (0.76-1.61)
Samoa	1 (0-2)	0.83 (0.18-2.39)	1 (0-2)	0.78 (0.18-2.33)	0.19 (-0.89-1.3)
San Marino	0 (0-1)	0.96 (0.22-2.75)	1 (0-2)	0.71 (0.16-1.81)	0.32 (-0.94-1.6)
Sao Tome and Principe	0 (0-0)	0.08 (0.02-0.24)	0 (0-0)	0.09 (0.02-0.25)	-0.65 (-1.9-0.62)
Saudi Arabia	17 (4-48)	0.43 (0.09-1.24)	40 (8-116)	0.4 (0.09-1.14)	-0.32 (-2.21-1.61)
Senegal	4 (1-12)	0.15 (0.03-0.46)	8 (2-22)	0.12 (0.03-0.36)	-0.34 (-1.74-1.08)
Serbia	59 (13-172)	0.67 (0.15-1.92)	100 (22-283)	0.57 (0.13-1.62)	0.89 (0.33-1.46)
Seychelles	0 (0-1)	0.84 (0.18-2.37)	1 (0-2)	0.67 (0.15-1.97)	-0.61 (-0.96--0.27)
Sierra Leone	3 (1-8)	0.18 (0.04-0.5)	3 (1-10)	0.13 (0.03-0.37)	-1.65 (-3.09--0.2)

Singapore	7 (2-21)	0.47 (0.11-1.33)	23 (5-61)	0.28 (0.06-0.73)	-0.05 (-0.62-0.53)
Slovakia	33 (7-97)	0.62 (0.14-1.83)	46 (10-130)	0.48 (0.11-1.36)	0.23 (-0.22-0.68)
Slovenia	12 (3-36)	0.53 (0.12-1.51)	25 (6-70)	0.48 (0.11-1.38)	1.16 (0.39-1.94)
Solomon Islands	1 (0-2)	0.76 (0.17-2.22)	2 (0-5)	0.68 (0.14-2.05)	0.36 (-1.39-2.14)
Somalia	4 (1-12)	0.36 (0.08-1.1)	7 (1-20)	0.27 (0.05-0.75)	-1.06 (-3.75-1.71)
South Africa	135 (32-374)	0.87 (0.2-2.35)	109 (24-330)	0.32 (0.07-0.98)	-2.86 (-3.83--1.87)
South Sudan	7 (1-18)	0.39 (0.08-1.15)	7 (2-22)	0.34 (0.07-1)	-0.92 (-2.81-1)
Spain	551 (127-1503)	1.04 (0.24-2.84)	696 (166-1868)	0.55 (0.13-1.48)	-1.11 (-2.06--0.14)
Sri Lanka	48 (11-140)	0.78 (0.17-2.29)	71 (16-206)	0.33 (0.07-0.96)	-1.55 (-2.33--0.75)
Sudan	47 (11-137)	0.83 (0.19-2.38)	94 (21-285)	0.75 (0.16-2.3)	-0.28 (-1.85-1.32)
Suriname	1 (0-3)	0.52 (0.11-1.46)	2 (0-6)	0.35 (0.07-1.07)	-0.86 (-1.4--0.31)
Sweden	190 (41-535)	1.1 (0.24-3.11)	281 (68-788)	1.01 (0.24-2.84)	0.27 (-0.93-1.48)
Switzerland	138 (32-401)	1.2 (0.28-3.49)	237 (60-604)	0.98 (0.24-2.52)	0.28 (-0.78-1.35)
Syrian Arab Republic	51 (11-150)	1.37 (0.3-4)	74 (16-207)	1 (0.22-2.88)	-0.14 (-1.96-1.72)
Taiwan (Province of China)	83 (20-234)	0.85 (0.19-2.25)	296 (66-863)	0.62 (0.14-1.81)	1.1 (0.49-1.7)

Tajikistan	17 (4-50)	0.75 (0.17-2.25)	16 (3-47)	0.45 (0.09-1.29)	-1.62 (-3.03--0.19)
Thailand	248 (53-693)	1.03 (0.22-2.91)	743 (171-2055)	0.67 (0.15-1.85)	0.49 (-0.19-1.18)
Timor-Leste	1 (0-4)	0.85 (0.18-2.45)	4 (1-12)	0.78 (0.17-2.23)	1.02 (-0.83-2.91)
Togo	3 (1-9)	0.44 (0.1-1.24)	6 (1-17)	0.26 (0.06-0.75)	-1.27 (-3.31-0.81)
Tokelau	0 (0-0)	0.69 (0.15-2.01)	0 (0-0)	0.54 (0.11-1.6)	-0.13 (-0.37-0.12)
Tonga	0 (0-1)	1.18 (0.28-3.13)	1 (0-2)	0.95 (0.2-2.69)	0.07 (-0.77-0.92)
Trinidad and Tobago	3 (1-8)	0.39 (0.09-1.2)	5 (1-17)	0.29 (0.06-0.91)	0.39 (-0.01-0.79)
Tunisia	52 (13-141)	1.65 (0.39-4.35)	132 (28-382)	1.22 (0.26-3.43)	0.34 (-0.45-1.13)
Tmrkiye	334 (73-962)	1.3 (0.28-3.81)	606 (141-1758)	0.75 (0.18-2.21)	-0.71 (-1.38--0.04)
Turkmenistan	7 (2-20)	0.53 (0.11-1.47)	10 (2-29)	0.34 (0.07-0.98)	-0.51 (-1.64-0.63)
Tuvalu	0 (0-0)	0.6 (0.13-1.77)	0 (0-0)	0.6 (0.13-1.69)	0.36 (-0.43-1.14)
Uganda	13 (3-36)	0.38 (0.08-1.07)	29 (6-84)	0.35 (0.07-0.98)	-0.87 (-3.15-1.46)
Ukraine	322 (74-958)	0.53 (0.12-1.56)	336 (72-992)	0.42 (0.09-1.23)	0 (-0.52-0.51)
United Arab Emirates	2 (0-4)	0.84 (0.19-2.34)	9 (2-27)	1.1 (0.24-3.04)	0.06 (-3.53-3.77)
United Kingdom	1419 (329-4164)	1.51 (0.35-4.34)	1425 (335-3854)	0.89 (0.21-2.44	-1.28 (-2.22--0.

				)	33)
United Republic of Tanzania	45 (11-129)	0.78 (0.18-2.16)	86 (20-238)	0.52 (0.12-1.4)	-1.24 (-3.06-0.62)
United States of America	5054 (1203-13946)	1.49 (0.35-4.13)	7091 (1721-19159)	1.08 (0.26-2.93)	-0.59 (-1.23-0.05)
United States Virgin Islands	0 (0-1)	0.33 (0.07-0.94)	1 (0-1)	0.28 (0.06-0.78)	1.59 (1.06-2.13)
Uruguay	16 (4-48)	0.43 (0.1-1.23)	30 (7-88)	0.46 (0.11-1.33)	0.95 (0.34-1.57)
Uzbekistan	24 (5-69)	0.23 (0.05-0.68)	52 (12-162)	0.28 (0.06-0.87)	0.89 (-0.12-1.91)
Vanuatu	0 (0-0)	0.46 (0.1-1.31)	0 (0-1)	0.35 (0.08-0.96)	-0.65 (-2.34-1.08)
Venezuela (Bolivarian Republic of)	49 (11-136)	0.67 (0.15-1.81)	103 (22-293)	0.37 (0.08-1.05)	-0.53 (-1.26-0.2)
Viet Nam	323 (73-902)	0.99 (0.22-2.81)	601 (133-1641)	0.76 (0.17-2.06)	-0.5 (-1.26-0.27)
Yemen	29 (7-86)	1.01 (0.23-3.04)	86 (19-248)	1 (0.23-2.86)	0.28 (-1.68-2.28)
Zambia	10 (2-27)	0.65 (0.14-1.81)	18 (4-53)	0.52 (0.11-1.52)	-0.94 (-3.17-1.35)
Zimbabwe	22 (5-64)	0.99 (0.22-2.93)	25 (5-73)	0.76 (0.17-2.21)	-0.92 (-2.91-1.11)

Supplementary Table S2 The number of DALYs cases and ASDAR of smoking-attributable burden of ADOD stratified by sex, age , SDI region, and country with EAPC from 1990 to 2021 globally.

location	1990		2021		EAPC_95%CI
	Number_95%UI	ASR	Number_95%UI	ASR	
**Global**	794915 (344378-1839709)	23.33 (9.99-54.46)	1533214 (662723-3496420)	18.36 (7.9-42.07)	-0.05 (-0.42-0.32)
**Sex**					
Female	262310 (112857-602151)	13.65 (5.8-31.32)	423191 (185085-947536)	9.01 (3.94-20.16)	-1.5 (-1.56--1.45)
Male	532606 (227759-1241825)	37.45 (15.98-87.69)	1110023 (474038-2558503)	30.56 (12.72-71.5)	-0.73 (-0.8--0.66)
**Age**					
40-44 years	1707 (706-3175)	0.6 (0.25-1.11)	1881 (733-3569)	0.38 (0.15-0.71)	-1.55 (-1.59--1.51)
45-49 years	9916 (4405-21080)	4.27 (1.9-9.08)	13889 (5976-29822)	2.93 (1.26-6.3)	-1.26 (-1.31--1.21)
50-54 years	28591 (12252-63566)	13.45 (5.76-29.9)	45218 (18886-101558)	10.16 (4.24-22.83)	-0.92 (-0.95--0.9)
55-59 years	56717 (22716-134303)	30.62 (12.27-72.52)	98577 (39090-232623)	24.91 (9.88-58.78)	-0.62 (-0.68--0.57)
60-64 years	94166 (36645-215744)	58.63 (22.82-134.33)	146604 (57506-350090)	45.81 (17.97-109.39)	-0.71 (-0.79--0.63)
65-69 years	120040 (50021-287288)	97.11 (40.47-232.42)	212369 (89433-494844)	76.99 (32.42-179.39)	-0.77 (-0.88--0.66)
70-74 years	119682	141.37	228266	110.9	-0.87

	(53018-285686)	(62.62-337.45)	(102175-536104)	(49.64-260.45)	(-0.97--0.77)
75-79 years	121415 (52336-270756)	197.24 (85.02-439.86)	217548 (94558-480191)	164.95 (71.7-364.1)	-0.72 (-0.77--0.68)
80-84 years	118156 (48775-268661)	334 (137.88-759.44)	238189 (100160-562421)	271.96 (114.36-642.16)	-0.83 (-0.89--0.77)
85-89 years	79533 (30354-177054)	526.32 (200.88-1171.68)	190137 (73634-430240)	415.86 (161.05-941)	-0.98 (-1.04--0.92)
90-94 years	33923 (12951-79093)	791.63 (302.22-1845.72)	102662 (39652-230871)	573.87 (221.65-1290.55)	-1.2 (-1.26--1.15)
95+ years	11071 (3981-25901)	1087.4 (391.04-2544.08)	37874 (13692-88378)	694.9 (251.21-1621.53)	-1.62 (-1.69--1.55)
**Regional levels**					
High-middle SDI	200798 (84592-461915)	22.72 (9.46-52.39)	435096 (191160-971669)	21.96 (9.6-49.15)	-0.17 (-0.19--0.15)
High SDI	310666 (136519-716665)	27.97 (12.27-64.53)	442915 (190965-1020744)	19.05 (8.33-43.29)	-1.34 (-1.4--1.28)
Low-middle SDI	71967 (31552-171280)	15.95 (6.82-37.64)	153657 (63421-366742)	13.02 (5.45-31.35)	-0.7 (-0.72--0.68)
Low SDI	15215 (6575-36754)	9.72 (4.09-23.07)	30072 (11978-72620)	8.1 (3.29-19.44)	-0.64 (-0.69--0.59)
Middle SDI	195493 (83543-446430)	24.14 (10.01-56.67)	470385 (201779-1086306)	19.38 (8.25-44.76)	-0.88 (-0.94--0.82)
World Bank High Income	357282 (157216-822456)	27.52 (12.05-63.36)	482229 (206895-1110123)	18.27 (7.94-41.39)	-0.58 (-1.29-0.14)
World Bank Low Income	10124 (4308-23658)	9.79 (4-22.71)	18958 (7663-43089)	7.66 (3.1-17.85)	-0.68 (-2.21-0.88)
World Bank Lower Middle Income	118461 (52178-277721)	15.21 (6.6-35.71)	256910 (106369-618259)	12.67 (5.27-30.4)	0 (-1.01-1.02)
World Bank Upper Middle	308269 (129646-70	24.9 (10.2-57.7)	774023 (338340-177	23.03 (9.91-52.42	0.9 (0.5-1.3

Income	6591)		4004)	)	1)
Advanced Health System	397350 (175229-911453)	24.68 (10.8-56.61)	543646 (233336-1249864)	17.34 (7.56-39.22)	-0.38 (-1.02-0.26)
Africa	24167 (10326-56358)	11.75 (4.96-26.85)	45109 (18279-105773)	9.35 (3.77-21.73)	-0.68 (-2.17-0.82)
African Region	14998 (6325-35172)	9.3 (3.94-21.58)	26089 (10400-63195)	6.81 (2.72-16.28)	-1.02 (-2.55-0.53)
America	170678 (75385-397674)	28.8 (12.66-66.74)	250108 (109851-569671)	18.05 (7.93-40.99)	-0.87 (-1.09--0.66)
Andean Latin America	1262 (530-2998)	7.05 (2.96-16.84)	3188 (1353-7589)	5.65 (2.4-13.4)	0.26 (-0.4-0.93)
Asia	379970 (160384-879887)	24.22 (10.05-57.02)	958325 (412336-2196528)	20.56 (8.66-47.66)	0.53 (-0.07-1.13)
Australasia	4412 (1975-10040)	18.86 (8.37-42.69)	7016 (3017-16580)	12.11 (5.19-28.39)	-0.63 (-1.19--0.06)
Basic Health System	319746 (135258-733914)	27.14 (11.09-63.45)	820988 (358884-1881669)	23.83 (10.19-54.64)	0.73 (0.18-1.29)
Caribbean	3466 (1490-7884)	14.49 (6.29-33.31)	6059 (2666-14005)	11.08 (4.89-25.64)	-0.2 (-0.49-0.09)
Central Africa	1101 (456-2598)	5.18 (2.12-12.49)	2018 (783-4751)	3.86 (1.48-9.38)	-0.95 (-2.54-0.65)
Central Asia	4360 (1950-10095)	10.16 (4.53-23.61)	8070 (3529-18936)	11.45 (5.08-26.38)	0.98 (0.39-1.58)
Central Europe	26911 (11851-61193)	19.07 (8.36-43.19)	33947 (14977-77857)	14.77 (6.44-33.75)	0.13 (-0.45-0.72)
Central Latin America	8966 (3992-20344)	12.89 (5.72-29.41)	16187 (7134-35872)	6.74 (2.97-14.9)	-0.92 (-1.64--0.19)
Central Sub-Saharan Africa	964 (391-2326)	5.88 (2.41-14.34)	2170 (823-5189)	5.05 (1.92-12.35)	-0.34 (-1.97-1.32)
Commonwealth	45317	29.41	51026	17.04	-1.29

High Income	(20043-103030)	(12.97-67.43)	(21940-116169)	(7.41-39.15)	(-1.99--0.59)
Commonwealth Low Income	9593 (4025-22313)	14.59 (6.08-33.9)	24087 (9824-55995)	13.14 (5.25-31.03)	0.36 (-1.08-1.83)
Commonwealth Middle Income	56760 (24601-135747)	12.88 (5.45-30.33)	121309 (49687-292812)	9.75 (4.06-23.64)	-0.23 (-1.3-0.84)
East Asia	218922 (90302-508009)	33.68 (13.33-78.42)	611761 (262060-1401979)	29.95 (12.67-68.1)	1.11 (0.62-1.6)
Eastern Africa	5082 (2137-12112)	10.72 (4.36-25.23)	9343 (3685-22490)	7.98 (3.2-19.6)	-0.89 (-2.62-0.87)
Eastern Europe	28706 (12568-65937)	10.81 (4.76-24.69)	43178 (18713-97785)	11.92 (5.23-26.87)	1.06 (0.54-1.59)
Eastern Mediterranean Region	27597 (11904-65395)	20.14 (8.61-47.33)	54934 (23179-127050)	16.12 (6.76-37.55)	-0.42 (-1.67-0.85)
Eastern Sub-Saharan Africa	4798 (1953-11611)	9.54 (3.88-22.72)	8554 (3337-21035)	6.91 (2.76-16.31)	-0.99 (-2.76-0.81)
Europe	218922 (96515-497113)	21.52 (9.39-49.13)	277774 (119426-627870)	15.99 (6.95-35.6)	-0.21 (-0.86-0.45)
European Region	222487 (98094-505517)	21.18 (9.25-48.35)	284048 (122060-642004)	15.77 (6.86-35.18)	-0.23 (-0.83-0.36)
High-income Asia Pacific	45858 (19475-106212)	24.84 (10.44-57.2)	87434 (36859-198890)	15.81 (6.77-36.48)	0.1 (-0.82-1.02)
High-income North America	125527 (55484-291114)	34.48 (15.23-79.44)	164284 (72810-373190)	23.32 (10.39-52.46)	-0.79 (-1.37--0.21)
Limited Health System	74347 (32406-176854)	13.28 (5.68-31.12)	163106 (66140-387751)	10.42 (4.3-24.82)	-0.15 (-1.31-1.03)
Minimal Health System	2697 (1084-6536)	6.11 (2.44-14.69)	4385 (1717-10496)	4.73 (1.89-11.37)	-1.03 (-2.65-0.63)
North Africa and Middle East	30637 (13261-69330)	23.87 (10.23-54.52)	66397 (28430-151668)	18.22 (7.72-41.54)	-0.22 (-1.28-0.84)

North America	125521 (55481-291098)	34.47 (15.23-79.43)	164279 (72807-373184)	23.32 (10.39-52.45)	-0.79 (-1.37--0.21)
Northern Africa	10496 (4478-24154)	23.16 (9.75-53.47)	23240 (9698-53320)	20.28 (8.35-46.85)	0.17 (-0.94-1.29)
Oceania	299 (124-699)	12.78 (5.38-29.87)	661 (279-1524)	10.53 (4.46-24.93)	-0.35 (-1.63-0.94)
Region of the Americas	170678 (75385-397674)	28.8 (12.66-66.74)	250108 (109851-569671)	18.05 (7.93-40.99)	-0.87 (-1.09--0.66)
South-East Asia Region	73544 (32240-172113)	14.87 (6.41-34.49)	179658 (74747-431087)	12.08 (5.08-28.78)	0.3 (-0.67-1.28)
South Asia	55864 (24134-134195)	13.94 (5.84-32.94)	129918 (52973-310723)	10.95 (4.55-26.31)	0.06 (-0.68-0.81)
Southeast Asia	40981 (18279-93081)	20.78 (9.1-47.13)	89722 (38569-210580)	16.17 (6.85-37.31)	0.02 (-0.82-0.86)
Southern Africa	5296 (2293-11857)	16.13 (6.88-35.95)	6489 (2673-15677)	8.58 (3.53-20.75)	-1.89 (-3.18--0.57)
Southern Latin America	5659 (2543-12845)	12.59 (5.64-28.39)	9343 (4055-20860)	10.53 (4.59-23.53)	0.03 (-0.16-0.23)
Southern Sub-Saharan Africa	4087 (1794-9195)	18.6 (8-41.45)	4104 (1729-9566)	8.43 (3.5-19.63)	-2.07 (-3.03--1.11)
Tropical Latin America	26318 (10755-62011)	34.9 (14.37-82.59)	52048 (22656-122054)	20.81 (9.05-48.62)	-0.57 (-1.12--0.01)
Western Africa	2192 (890-5247)	3.62 (1.45-8.51)	4019 (1574-9979)	2.89 (1.12-7.07)	-0.88 (-2.36-0.62)
Western Europe	154412 (67714-351221)	25.78 (11.25-59.3)	184594 (78899-420813)	17.23 (7.43-38.39)	-0.62 (-1.53-0.29)
Western Pacific Region	282231 (118260-654716)	30.64 (12.38-71.64)	730977 (318248-1692496)	26.05 (11.24-59.98)	0.84 (0.44-1.24)
Western Sub-Saharan Africa	2506 (1014-5965)	3.72 (1.48-8.68)	4580 (1794-11406	2.95 (1.15-7.14)	-0.92 (-2.41-0.

			)		58)
**Country levels**					
Afghanistan	591 (228-1428)	11.22 (4.31-26.64)	706 (289-1676)	10.74 (4.24-25.32)	-1.31 (-2.88-0.29)
Albania	471 (207-1085)	27.61 (11.58-64.74)	1313 (562-2978)	30.54 (12.98-69.92)	2.51 (1.99-3.03)
Algeria	2674 (1144-6436)	35.04 (14.41-86.04)	6709 (2714-15287)	26.05 (10.06-60.2)	0.16 (-1.01-1.35)
American Samoa	3 (1-6)	13.81 (5.72-30.79)	5 (2-12)	12.21 (5.1-28.79)	0.94 (0.01-1.88)
Andorra	12 (5-28)	23.31 (9.58-53.24)	29 (12-62)	17.53 (7.56-36.7)	0.1 (-0.44-0.64)
Angola	273 (111-666)	10.05 (4.06-24.55)	809 (303-1894)	9.28 (3.45-21.84)	-0.11 (-1.96-1.77)
Antigua and Barbuda	4 (2-9)	7.34 (3.31-16.68)	7 (3-15)	6.77 (2.87-15.3)	-0.01 (-0.31-0.3)
Argentina	4295 (1926-9775)	13.63 (6.09-30.7)	6749 (2908-15233)	11.88 (5.15-26.74)	0.05 (-0.12-0.23)
Armenia	427 (186-971)	18.1 (7.82-40.56)	944 (395-2153)	21.37 (8.97-48.36)	2.25 (1.89-2.61)
Australia	3477 (1544-7899)	17.83 (7.89-40.22)	5419 (2306-12741)	11.13 (4.75-26.1)	-0.71 (-1.28--0.13)
Austria	1873 (804-4385)	15.32 (6.64-35.91)	3588 (1535-7914)	18.07 (7.8-39.91)	1.25 (0.38-2.13)
Azerbaijan	572 (245-1322)	12.62 (5.29-28.7)	1439 (636-3260)	17.32 (7.62-39.58)	1.75 (1.17-2.35)
Bahamas	10 (4-22)	7.05 (3.03-16.24)	25 (10-55)	6.86 (2.84-14.99)	0.94 (0.36-1.52)
Bahrain	26 (11-61)	22.99 (9.1-57.91)	109 (48-243)	18.33 (7.72-41.91)	-0.18 (-2.06-1.74)

Bangladesh	6305 (2616-14712)	16.93 (7-39.76)	17971 (7385-42014)	15.29 (6.15-35.46)	1.06 (-0.18-2.32)
Barbados	22 (9-50)	6.84 (2.92-15.42)	30 (13-69)	5.64 (2.39-13.02)	-0.43 (-0.93-0.07)
Belarus	1835 (763-3987)	14.64 (6.17-31.56)	2322 (1010-5421)	14.01 (6.12-32.39)	0.67 (0.12-1.21)
Belgium	4885 (2106-11361)	30.55 (13.17-71.96)	5504 (2250-12449)	20.51 (8.7-46.98)	-0.67 (-1.57-0.24)
Belize	9 (4-21)	10.18 (4.56-23.23)	23 (9-51)	8.56 (3.53-19.25)	-0.02 (-0.97-0.93)
Benin	73 (30-176)	4.05 (1.63-9.79)	106 (43-260)	2.37 (0.95-5.85)	-2.1 (-3.53--0.63)
Bermuda	5 (2-12)	9.06 (3.95-20.78)	14 (6-31)	9.7 (4.2-21.09)	1.67 (1.07-2.27)
Bhutan	14 (5-33)	8.58 (3.15-20.68)	45 (17-102)	8.49 (3.17-18.94)	1.61 (0.43-2.8)
Bolivia (Plurinational State of)	189 (78-444)	7.04 (2.89-16.69)	456 (186-1104)	5.68 (2.26-13.38)	0.44 (-0.44-1.33)
Bosnia and Herzegovina	752 (321-1599)	22.05 (9.29-48.06)	1436 (644-3240)	22.52 (10.17-50.2)	1.68 (1.09-2.26)
Botswana	71 (29-167)	17.25 (6.7-40.67)	140 (55-333)	12.24 (4.76-29.44)	-0.43 (-1.75-0.92)
Brazil	25559 (10426-60213)	34.69 (14.31-82.16)	50697 (22037-118830)	20.71 (8.99-48.4)	-0.55 (-1.1-0)
Brunei Darussalam	18 (8-44)	22.16 (9.22-55.7)	33 (14-78)	12.54 (5.21-29.74)	-0.73 (-2.08-0.63)
Bulgaria	1948 (865-4163)	16.42 (7.11-35.78)	1644 (728-3797)	11.54 (4.97-26.98)	-0.55 (-1.32-0.23)
Burkina Faso	105 (41-252)	2.94 (1.13-7.27)	180 (72-437)	2.31 (0.89-5.57)	-0.92 (-2.32-0.

					5)
Burundi	128 (52-291)	7.13 (2.91-16.67)	154 (61-360)	4.03 (1.61-9.59)	-1.9 (-3.56--0.22)
Cabo Verde	9 (4-23)	4.03 (1.63-9.84)	10 (4-25)	2.29 (0.87-5.62)	-1.76 (-2.36--1.16)
Cambodia	815 (348-1890)	25.27 (10.83-60.19)	2456 (1005-5893)	26.06 (10.55-63.42)	0.97 (-0.33-2.28)
Cameroon	163 (62-387)	4.63 (1.68-11.09)	315 (121-766)	3.26 (1.15-8.07)	-1.26 (-2.83-0.34)
Canada	10492 (4984-22254)	32.58 (15.46-69.3)	15559 (7149-33726)	19.87 (9.19-43.67)	-0.85 (-1.49--0.2)
Central African Republic	55 (22-135)	6.91 (2.68-17.17)	73 (28-186)	4.77 (1.82-11.73)	-1.37 (-3.15-0.45)
Chad	117 (48-282)	4.95 (2.03-12.19)	185 (71-435)	4.27 (1.62-10.13)	-1.4 (-2.98-0.21)
Chile	890 (405-1984)	9.3 (4.2-20.77)	1884 (825-4237)	7.23 (3.17-16.22)	0.09 (-0.18-0.37)
China	214824 (88552-498169)	34.5 (13.61-80.31)	602501 (257945-1379583)	30.63 (12.98-69.65)	1.11 (0.62-1.61)
Colombia	1493 (667-3401)	9.71 (4.3-21.85)	3503 (1514-7788)	6.18 (2.68-13.79)	-0.06 (-0.63-0.52)
Comoros	14 (6-34)	10.4 (3.9-24.71)	32 (12-79)	8.63 (3.14-20.85)	0.16 (-1.16-1.5)
Congo	59 (23-145)	7.46 (2.89-18.43)	174 (66-407)	9 (3.43-21.69)	0.97 (-0.57-2.53)
Cook Islands	2 (1-4)	14.58 (5.97-34.96)	3 (1-7)	11.45 (4.85-27.31)	0.67 (0.25-1.09)
Costa Rica	237 (103-515)	14.68 (6.38-32.1)	552 (239-1254)	9.9 (4.27-22.45)	0.09 (-0.44-0.62)
Cote d'Ivoire	152	5.35	352	4.08	-0.9

	(58-372)	(1.98-13)	(142-853)	(1.65-10.01)	(-2.68-0.92)
Croatia	1451 (611-3310)	26.65 (11.23-59.96)	2163 (934-4942)	22.38 (9.73-51.34)	1.08 (0.35-1.8)
Cuba	1775 (753-3906)	18.17 (7.69-40.43)	2621 (1087-6094)	12.77 (5.31-29.71)	-0.26 (-0.7-0.18)
Cyprus	174 (73-407)	24.34 (9.82-57.03)	420 (186-924)	19.94 (8.82-43.96)	0.1 (-0.18-0.38)
Czechia	2784 (1247-6266)	20.47 (8.93-45.85)	3955 (1742-9023)	17.57 (7.8-40.33)	0.31 (-0.38-1)
Democratic People's Republic of Korea	1904 (788-4486)	14.53 (6.03-33.33)	3774 (1498-8982)	12.16 (4.79-29.73)	0.23 (-0.11-0.57)
Democratic Republic of the Congo	541 (224-1289)	4.67 (1.88-11.69)	1044 (389-2434)	3.49 (1.31-8.23)	-0.78 (-2.35-0.81)
Denmark	3932 (1628-8936)	44.94 (18.73-103.15)	3541 (1368-8406)	26.39 (10.06-62.59)	-1.46 (-2.33--0.59)
Djibouti	13 (5-30)	15.03 (5.9-35.62)	56 (22-130)	13.36 (5.09-30.54)	0.57 (-1.31-2.49)
Dominica	4 (2-8)	6.17 (2.65-13.76)	4 (2-10)	5.37 (2.24-12.85)	0.21 (-0.02-0.43)
Dominican Republic	640 (277-1481)	21.7 (9.2-50.74)	1601 (670-3655)	16.84 (7.02-38.61)	0.26 (-0.45-0.98)
Ecuador	454 (191-1042)	9.77 (4.09-22.86)	783 (329-1838)	4.99 (2.08-11.69)	-1.12 (-1.78--0.45)
Egypt	4436 (1810-10396)	26.42 (10.55-63.56)	10259 (4295-23341)	27.07 (11.54-62.68)	0.4 (-1.12-1.93)
El Salvador	156 (67-351)	5.54 (2.39-12.44)	363 (155-846)	5.69 (2.43-13.31)	1.27 (0.77-1.78)
Equatorial Guinea	10 (4-24)	6.66 (2.52-17.07)	22 (9-52)	5.76 (2.21-13.82)	-0.83 (-2.57-0.94)

Eritrea	26 (10-60)	3.1 (1.18-7.35)	57 (21-138)	2.66 (1-6.72)	-0.21 (-1.95-1.56)
Estonia	245 (107-553)	11.94 (5.18-26.77)	385 (168-838)	13.52 (5.91-29.81)	1.39 (0.62-2.16)
Eswatini	21 (8-51)	10.98 (4.02-27.15)	26 (10-61)	7.32 (2.65-18.09)	-0.92 (-2.74-0.94)
Ethiopia	565 (218-1368)	3.41 (1.32-8.33)	1109 (430-2765)	3.09 (1.2-7.73)	0.16 (-1.45-1.79)
Fiji	37 (15-86)	12.56 (5.18-29.01)	64 (26-144)	9.12 (3.8-21.36)	0.09 (-0.79-0.98)
Finland	1023 (425-2419)	14.21 (5.95-33.73)	1750 (713-3914)	12.5 (5.19-28)	0.74 (-0.15-1.64)
France	16294 (6950-37437)	18.34 (7.78-42.23)	21153 (8756-48498)	13.32 (5.69-30.35)	-0.38 (-1.26-0.5)
Gabon	26 (10-64)	5.19 (2.03-12.67)	48 (18-119)	5.49 (2.1-13.38)	0.1 (-0.96-1.17)
Gambia	19 (8-45)	6.72 (2.65-15.62)	33 (12-80)	3.96 (1.49-9.63)	-1.66 (-3.11--0.19)
Georgia	729 (333-1676)	12.03 (5.42-28.03)	1062 (459-2542)	16.47 (7.15-39.3)	2.33 (1.73-2.93)
Germany	34213 (15509-76345)	25.88 (11.66-57.88)	44416 (19910-99500)	19.76 (8.82-43.8)	-0.08 (-1.07-0.92)
Ghana	172 (68-404)	4.28 (1.7-10.06)	500 (191-1215)	4.36 (1.64-10.62)	0.88 (-0.74-2.51)
Greece	5020 (2110-11403)	33.76 (14.06-76.65)	7055 (2896-15944)	24.25 (10.19-54.61)	0.13 (-0.8-1.06)
Greenland	8 (4-18)	33.07 (14.82-73.33)	17 (7-38)	26.93 (11.82-61.12)	0.71 (0.09-1.33)
Grenada	5 (2-12)	6.34 (2.8-14.74)	5 (2-12)	4.63 (2.06-10.8)	-0.92 (-1.25--

					0.59)
Guam	7 (3-16)	10.06 (4.26-24.18)	22 (9-51)	9.7 (3.97-23)	1.68 (1.14-2.22)
Guatemala	233 (101-550)	9.06 (3.87-21.17)	691 (297-1557)	6.99 (2.99-16.16)	0.39 (-0.84-1.64)
Guinea	147 (59-343)	5.27 (2.12-12.51)	212 (86-496)	4.54 (1.77-10.52)	-0.96 (-2.1-0.19)
Guinea-Bissau	7 (3-18)	2.29 (0.85-5.73)	14 (5-35)	2.47 (0.92-6.51)	0.4 (-1.3-2.14)
Guyana	22 (10-49)	6.83 (2.98-15.53)	32 (14-76)	5.75 (2.48-13.4)	0.53 (-0.39-1.46)
Haiti	131 (54-306)	5.03 (2.04-11.78)	217 (95-498)	3.94 (1.72-8.73)	-0.7 (-1.9-0.51)
Honduras	226 (97-512)	12.85 (5.42-29.15)	656 (255-1596)	12.14 (4.76-29.12)	0.43 (-0.69-1.56)
Hungary	2527 (1103-5756)	17.39 (7.51-39.69)	2527 (1083-5482)	12.62 (5.44-27.44)	-0.38 (-1.1-0.35)
Iceland	100 (43-215)	33.01 (14.25-70.74)	123 (52-267)	19.08 (8.13-40.9)	-1.3 (-1.81--0.79)
India	39665 (17119-95221)	12.7 (5.39-30.15)	98344 (40168-236215)	10.25 (4.28-24.93)	0.26 (-0.76-1.29)
Indonesia	11537 (5302-26170)	16.01 (7.14-36.82)	32682 (13974-79988)	18.42 (7.78-44.41)	1.13 (0.12-2.15)
Iran (Islamic Republic of)	3053 (1307-7012)	14.74 (6.31-34.53)	9752 (4044-22313)	14.22 (5.85-32.91)	1.41 (0.48-2.35)
Iraq	2614 (1098-5830)	37.47 (15.8-83.3)	4309 (1745-10588)	25.36 (10.28-59.46)	-1.14 (-2.51-0.24)
Ireland	1609 (707-3650)	41.25 (17.96-96.14)	1647 (709-3755)	19.48 (8.36-44.55)	-2.09 (-2.47--1.7)
Israel	1054	22.79	1939	14.58	-0.86

	(425-2421)	(9.18-52.68)	(772-4244)	(5.88-32.1)	(-1.08--0.63)
Italy	24651 (10167-56683)	27.62 (11.44-63.38)	29263 (12216-64625)	17.4 (7.47-37.94)	-0.62 (-1.65-0.43)
Jamaica	213 (96-493)	11.66 (5.23-26.99)	288 (124-635)	8.87 (3.89-19.39)	-0.46 (-0.66--0.27)
Japan	39011 (16396-89583)	24.26 (10.15-55.19)	67192 (28223-150479)	15.37 (6.6-35.52)	0.02 (-1.11-1.16)
Jordan	347 (149-797)	38.17 (16.33-88.33)	1492 (668-3304)	28.24 (12.25-63.16)	-0.22 (-1.87-1.45)
Kazakhstan	1013 (442-2303)	8.59 (3.72-19.82)	1312 (558-2973)	7.79 (3.34-17.96)	0.06 (-0.26-0.39)
Kenya	652 (278-1523)	10.5 (4.43-25.21)	1057 (426-2451)	6.06 (2.37-13.97)	-1.8 (-3.44--0.13)
Kiribati	7 (3-17)	24.63 (10.06-57.99)	16 (7-35)	28.21 (11.93-65.35)	0.51 (-0.77-1.8)
Kuwait	106 (46-236)	25.51 (10.89-56.31)	475 (217-1076)	22.08 (9.83-51.15)	0.21 (-1.18-1.62)
Kyrgyzstan	328 (145-735)	12.09 (5.24-27.6)	753 (316-1722)	18.7 (7.88-43.49)	2.18 (1.37-3)
Lao People's Democratic Republic	294 (128-658)	19.34 (8.5-43.51)	695 (299-1605)	20.05 (8.63-45.1)	0.64 (-0.65-1.95)
Latvia	436 (192-976)	12.21 (5.29-27.5)	433 (188-988)	10.45 (4.57-24.04)	0.48 (-0.27-1.25)
Lebanon	557 (246-1240)	30.97 (13.66-69.25)	2292 (979-5109)	35.63 (15.39-80.46)	1.52 (1.09-1.95)
Lesotho	98 (38-231)	15.03 (5.57-35.63)	121 (47-274)	16.7 (6.26-37.66)	0.31 (-0.87-1.52)
Liberia	34 (14-83)	3.56 (1.4-8.48)	47 (19-113)	2.62 (1.03-6.38)	-1.9 (-3.22--0.55)

Libya	370 (151-847)	22.67 (9.23-50.81)	740 (311-1743)	17.71 (7.25-42.21)	-0.04 (-1.09-1.03)
Lithuania	602 (265-1373)	13.48 (5.92-30.84)	691 (293-1675)	10.95 (4.65-26.18)	0.44 (-0.28-1.16)
Luxembourg	84 (36-197)	15.46 (6.62-35.88)	146 (61-335)	13.01 (5.38-29.38)	-0.22 (-0.83-0.38)
Madagascar	289 (123-681)	8.25 (3.39-19.3)	255 (108-589)	3.62 (1.47-8.59)	-3.08 (-4.93--1.19)
Malawi	313 (126-761)	11.97 (4.78-29.57)	573 (234-1415)	10.3 (4.03-25.42)	-0.47 (-2.17-1.26)
Malaysia	1725 (723-3928)	22.01 (9.25-50.65)	3533 (1523-8417)	15.29 (6.5-36.71)	-0.55 (-1.5-0.41)
Maldives	18 (8-40)	33.61 (14.82-77.68)	58 (26-132)	21.71 (9.41-49.02)	-0.24 (-1.63-1.17)
Mali	121 (47-282)	4.44 (1.71-10.35)	422 (162-1044)	7.04 (2.61-17.71)	1.56 (-0.2-3.35)
Malta	82 (35-184)	20.29 (8.5-45.17)	137 (57-295)	13.14 (5.49-28.4)	-0.4 (-1.09-0.29)
Marshall Islands	1 (1-3)	10.38 (4.01-25.73)	3 (1-6)	10.7 (4.16-26.64)	0.97 (-0.68-2.64)
Mauritania	32 (13-72)	3.72 (1.51-8.42)	58 (23-137)	3.06 (1.17-7.3)	-0.64 (-1.81-0.54)
Mauritius	93 (40-215)	15.25 (6.54-36.11)	254 (113-554)	14.29 (6.26-32.35)	1.42 (0.86-1.99)
Mexico	5022 (2274-11259)	14.94 (6.79-34.71)	7065 (3027-15520)	6.08 (2.6-13.28)	-1.77 (-2.56--0.97)
Micronesia (Federated States of)	7 (3-18)	17.42 (6.97-41.14)	10 (4-25)	15.85 (5.99-37.29)	0.43 (-0.66-1.53)
Monaco	18 (8-39)	22.83 (9.65-50.85	21 (8-49)	18.4 (7.66-42.22	-0.55 (-1.92-0.

		)		)	84)
Mongolia	113 (48-261)	12.05 (5.09-27.61)	273 (118-641)	14.72 (6.5-35)	1.37 (0.23-2.52)
Montenegro	141 (63-327)	23.56 (10.45-55.2)	199 (86-463)	20.31 (8.63-47.49)	0.26 (-0.17-0.69)
Morocco	1635 (715-3677)	13.27 (5.79-30.49)	2500 (1021-5621)	7.92 (3.19-17.96)	-0.88 (-1.59--0.15)
Mozambique	364 (148-862)	8.85 (3.55-21.27)	551 (209-1363)	7.04 (2.66-16.98)	-0.97 (-2.66-0.74)
Myanmar	5031 (2305-11011)	30.42 (13.63-66.63)	5924 (2466-14356)	14.93 (6.01-36.3)	-1.61 (-2.49--0.72)
Namibia	87 (37-212)	21.76 (8.94-55.88)	147 (60-349)	15.68 (6.34-36.99)	-0.78 (-2.31-0.78)
Nauru	1 (0-1)	18.35 (7.17-45.63)	1 (0-2)	14.06 (5.83-33.77)	-0.55 (-1.91-0.82)
Nepal	1592 (710-3561)	25.34 (10.79-56.26)	3350 (1386-7936)	18.86 (7.51-45.64)	-0.14 (-1.32-1.07)
Netherlands	6357 (2732-14716)	31.08 (13.28-72.44)	9400 (3802-21339)	24.04 (9.85-55.02)	0.38 (-0.38-1.14)
New Zealand	935 (404-2146)	23.92 (10.24-55.11)	1596 (711-3645)	17.55 (7.8-40.04)	-0.27 (-0.77-0.24)
Nicaragua	126 (55-281)	9.57 (4.18-21.44)	367 (160-804)	8.33 (3.61-18.21)	0.96 (-0.09-2.02)
Niger	48 (19-117)	2.75 (1.11-6.55)	146 (58-356)	2.92 (1.1-7.37)	-0.04 (-2.16-2.13)
Nigeria	1016 (407-2407)	3.08 (1.17-7.29)	1489 (572-3818)	2.04 (0.77-5.13)	-1.63 (-3.09--0.15)
Niue	0 (0-1)	11.6 (4.49-26.98)	0 (0-1)	10.53 (4.22-23.53)	0.09 (-0.18-0.36)
North	409	24.04	607	20.96	0.25

Macedonia	(177-931)	(10.51-53.58)	(272-1369)	(9.11-46.61)	(0.05-0.45)
Northern Mariana Islands	2 (1-4)	14.17 (6.03-33.65)	5 (2-13)	11.63 (4.82-28.2)	1.74 (0.38-3.13)
Norway	2051 (908-4627)	27.68 (12.14-62.18)	1538 (650-3560)	13.87 (6.05-31.63)	-2.51 (-3.34--1.67)
Oman	60 (25-142)	11.77 (4.71-27.66)	123 (51-286)	8.61 (3.47-19.63)	-0.69 (-2.38-1.03)
Pakistan	8288 (3501-20119)	18.15 (7.56-43.69)	10208 (4046-25451)	11.15 (4.35-27.71)	-1.88 (-3.13--0.63)
Palau	1 (0-2)	12.05 (4.75-27.98)	2 (1-5)	10.76 (4.33-25.13)	0.65 (-0.01-1.31)
Palestine	182 (77-419)	25.59 (10.58-60.15)	395 (166-904)	20.24 (8.31-47.28)	-0.81 (-2.35-0.75)
Panama	169 (75-374)	12.68 (5.55-28.31)	365 (156-812)	8.06 (3.46-17.95)	-0.66 (-1.1--0.22)
Papua New Guinea	160 (65-382)	10.94 (4.43-24.42)	401 (166-936)	9.48 (3.87-22.18)	-0.42 (-1.79-0.96)
Paraguay	758 (319-1752)	38.88 (16.23-90.66)	1352 (554-2895)	25.43 (10.31-54.44)	-0.8 (-1.51--0.09)
Peru	619 (258-1416)	5.83 (2.45-13.39)	1949 (826-4654)	5.88 (2.5-14.07)	0.94 (0.34-1.53)
Philippines	5902 (2678-12871)	28.31 (12.03-63.5)	11239 (4919-26001)	16.83 (7.16-39.08)	-0.86 (-1.99-0.28)
Poland	9341 (4075-21009)	22.08 (9.56-50.28)	11161 (4637-25585)	14.93 (6.23-34.23)	-0.34 (-0.88-0.2)
Portugal	1744 (757-4060)	12.47 (5.35-29.31)	2052 (884-4608)	7.92 (3.46-17.86)	-0.71 (-1.54-0.14)
Puerto Rico	380 (161-859)	10.76 (4.58-24.2)	748 (321-1717)	9.12 (3.89-20.99)	0.83 (0.17-1.5)

Qatar	10 (4-24)	14.44 (5.81-34.44)	78 (34-176)	11.6 (4.6-26.51)	-0.38 (-2.56-1.86)
Republic of Korea	6644 (2695-15649)	33.71 (12.91-80.41)	19644 (8044-46422)	20.98 (8.54-49.36)	0.61 (0-1.22)
Republic of Moldova	423 (183-982)	10.42 (4.44-24.2)	729 (307-1628)	11.86 (5.04-26.42)	1.63 (1.24-2.01)
Romania	3629 (1589-8582)	14.26 (6.22-33.02)	3927 (1715-8701)	10.27 (4.43-22.81)	-0.25 (-0.83-0.34)
Russian Federation	16434 (7239-37589)	9.47 (4.16-21.8)	30294 (13293-68273)	12.31 (5.45-27.66)	1.58 (1.08-2.09)
Rwanda	378 (158-893)	21.85 (8.86-53.97)	1071 (390-2613)	26.26 (9.46-61.29)	1.13 (-0.79-3.09)
Saint Kitts and Nevis	2 (1-5)	5.58 (2.38-12.81)	3 (1-6)	4.52 (1.89-10.51)	-0.8 (-1.05--0.55)
Saint Lucia	7 (3-16)	8.07 (3.39-19.45)	14 (6-34)	6 (2.55-14.32)	0.17 (-0.24-0.59)
Saint Vincent and the Grenadines	4 (2-10)	6 (2.57-13.95)	9 (4-19)	6.13 (2.63-13.81)	1.17 (0.79-1.54)
Samoa	15 (6-36)	19.56 (7.89-47.67)	23 (9-53)	17.82 (6.98-41.65)	0.04 (-0.85-0.94)
San Marino	8 (4-18)	21.81 (9.68-47.94)	14 (6-30)	15.88 (6.73-32.83)	-0.12 (-1.18-0.94)
Sao Tome and Principe	1 (1-3)	2.3 (0.91-5.51)	2 (1-5)	2.17 (0.84-5.34)	-0.79 (-1.89-0.31)
Saudi Arabia	472 (188-1111)	10.1 (3.97-23.92)	1350 (545-3053)	9.64 (3.86-22.34)	0.1 (-1.45-1.68)
Senegal	123 (50-300)	4.12 (1.65-10.31)	225 (85-565)	3.14 (1.19-7.68)	-0.64 (-1.82-0.57)
Serbia	1812 (780-4216)	17.4 (7.45-40.68	2677 (1196-5978)	15.78 (7.12-35.32	0.66 (0.05-1.

		)		)	27)
Seychelles	10 (4-24)	18.5 (7.57-42.39)	16 (7-36)	15.13 (6.38-34.48)	-0.41 (-0.71--0.11)
Sierra Leone	81 (34-185)	4.53 (1.9-10.45)	109 (43-260)	3.31 (1.3-8.15)	-1.46 (-2.69--0.22)
Singapore	185 (76-426)	10.26 (4.16-23.66)	565 (247-1181)	6.6 (2.86-13.86)	0.01 (-0.44-0.47)
Slovakia	860 (353-2002)	14.91 (6.09-35.67)	1211 (509-2747)	12.49 (5.29-28.36)	0.42 (-0.05-0.9)
Slovenia	356 (154-822)	14.54 (6.26-33.86)	632 (265-1400)	13.73 (5.75-30.04)	1.13 (0.4-1.86)
Solomon Islands	20 (8-47)	18.58 (7.79-44.8)	49 (20-116)	17.33 (7.1-41.02)	0.37 (-1.03-1.78)
Somalia	117 (45-290)	7.76 (2.88-19.28)	233 (86-537)	5.8 (2.08-12.75)	-0.97 (-3.14-1.25)
South Africa	3269 (1435-7213)	18.84 (8.3-41.85)	2993 (1288-7008)	7.4 (3.07-17.37)	-2.47 (-3.27--1.67)
South Sudan	176 (66-406)	8.51 (3.32-19.35)	193 (77-471)	6.96 (2.76-16.56)	-1.14 (-2.81-0.55)
Spain	12314 (5206-28103)	22.32 (9.39-51.37)	13723 (5710-31276)	12.62 (5.39-28.29)	-1.14 (-1.98--0.29)
Sri Lanka	1294 (561-2831)	16.37 (6.96-36.77)	1775 (777-4149)	7.28 (3.19-17.1)	-1.53 (-2.12--0.93)
Sudan	1249 (531-2922)	17.68 (7.25-41.57)	2258 (935-5503)	15.43 (6.25-37.6)	-0.56 (-1.96-0.85)
Suriname	32 (14-73)	13.58 (5.99-30.71)	54 (23-125)	8.9 (3.7-20.62)	-0.89 (-1.35--0.42)
Sweden	4071 (1752-9013)	24.37 (10.55-53.33)	5611 (2484-12538)	22.24 (9.97-49.67)	0.14 (-0.92-1.22)
Switzerland	2887	25.76	4289	20.03	-0.06

	(1216-6623)	(10.98-58.84)	(1788-9503)	(8.38-44.81)	(-0.99-0.88)
Syrian Arab Republic	1205 (518-2833)	28.98 (12.39-67.77)	1960 (841-4428)	20.19 (8.13-45.95)	-0.1 (-1.59-1.41)
Taiwan (Province of China)	2194 (932-5134)	17.64 (7.46-40.6)	5485 (2113-13408)	12.11 (4.69-29.43)	0.45 (-0.04-0.95)
Tajikistan	388 (170-922)	16.02 (6.98-38.31)	426 (169-1007)	9.57 (3.81-22.31)	-1.49 (-2.71--0.25)
Thailand	6148 (2531-13959)	21.81 (8.86-50.68)	15622 (6409-34864)	14.06 (5.79-31.32)	0.38 (-0.18-0.94)
Timor-Leste	36 (15-77)	19.54 (8.37-43.38)	112 (49-255)	16.68 (7.01-38.15)	0.71 (-0.77-2.21)
Togo	85 (33-199)	9.33 (3.52-21.67)	174 (68-420)	5.81 (2.23-13.99)	-1.01 (-2.69-0.7)
Tokelau	0 (0-0)	15.64 (5.9-38.21)	0 (0-0)	12.68 (4.88-31.1)	-0.26 (-0.43--0.08)
Tonga	11 (4-25)	25.04 (9.69-55.53)	15 (6-36)	20.18 (7.98-47.32)	-0.15 (-0.87-0.57)
Trinidad and Tobago	78 (35-187)	10.15 (4.41-24.78)	146 (63-346)	7.55 (3.28-17.68)	0.25 (-0.09-0.6)
Tunisia	1349 (604-3036)	34.76 (15.34-77.07)	2973 (1229-6817)	25.01 (10.16-57.46)	0.11 (-0.5-0.73)
Turkiye	8768 (3748-19479)	29.94 (12.83-68.27)	15189 (6485-34599)	17.34 (7.48-39.77)	-0.83 (-1.36--0.29)
Turkmenistan	197 (85-436)	12.25 (5.28-26.86)	290 (129-646)	8.41 (3.69-19.05)	-0.45 (-1.38-0.48)
Tuvalu	1 (0-2)	14.64 (5.5-37.04)	1 (1-3)	14.26 (5.87-34.21)	0.15 (-0.35-0.66)
Uganda	324 (132-729)	7.34 (2.85-16.92)	682 (263-1642)	6.53 (2.56-15.41)	-0.93 (-2.9-1.09)

Ukraine	8730 (3928-20308)	12.83 (5.67-29.86)	8324 (3482-19319)	10.39 (4.37-24.23)	-0.12 (-0.72-0.48)
United Arab Emirates	44 (18-99)	17.11 (6.72-40.04)	374 (150-807)	20.89 (8.21-49.29)	0.52 (-2.4-3.52)
United Kingdom	29828 (12892-68462)	31.32 (13.6-72.19)	27070 (11197-62977)	18.18 (7.59-42.28)	-1.5 (-2.37--0.63)
United Republic of Tanzania	1176 (484-2788)	15.39 (6.19-37.21)	2072 (810-4805)	10.43 (3.9-24.24)	-1.28 (-2.83-0.28)
United States of America	115023 (50586-268832)	34.69 (15.28-80.53)	148705 (65331-336695)	23.75 (10.43-53.44)	-0.78 (-1.35--0.21)
United States Virgin Islands	6 (2-13)	8.2 (3.21-18.36)	14 (6-30)	7.12 (3.08-15.58)	1.5 (0.96-2.05)
Uruguay	474 (207-1070)	12.02 (5.23-27.13)	709 (306-1650)	12.14 (5.19-28.04)	0.47 (-0.05-0.99)
Uzbekistan	594 (251-1408)	5.47 (2.3-13.03)	1571 (663-3666)	7.03 (3.01-16.56)	1.29 (0.42-2.16)
Vanuatu	5 (2-12)	11.03 (4.64-25.95)	11 (5-27)	8.15 (3.3-18.88)	-0.73 (-2.09-0.65)
Venezuela (Bolivarian Republic of)	1304 (589-2905)	15.75 (7.04-34.46)	2624 (1122-5992)	9.09 (3.87-20.64)	-0.51 (-1.19-0.18)
Viet Nam	8019 (3341-18249)	22.23 (9.31-50.31)	15230 (6109-36091)	17.01 (6.99-40.53)	-0.42 (-1.06-0.22)
Yemen	874 (361-1994)	23.61 (9.81-54.83)	2292 (967-5234)	21.8 (9.17-50.42)	-0.07 (-1.75-1.64)
Zambia	260 (103-586)	13.19 (5.12-30.26)	452 (178-1068)	10.04 (3.72-24.33)	-1.2 (-3.1-0.74)
Zimbabwe	540 (213-1243)	18.99 (7.24-45.22)	678 (257-1585)	15.09 (5.55-36.43)	-0.77 (-2.43-0.91)

Supplementary Table S3 The number of YLDs cases and corresponding ASR of smoking-attributable burden of ADOD stratified by sex, age , SDI region, and country with EAPC from 1990 to 2021 globally.

location	1990		2021		
	Number_95%UI	ASR	Number_95%UI	ASR	EAPC_95%CI
**Global**	266894 (162663-391752)	7.57 (4.64-11.22)	515882 (313106-759417)	6.12 (3.72-9.03)	-0.03 (-0.37-0.31)
**Sex**					
Female	89156 (53275-133867)	4.51 (2.71-6.8)	143042 (84582-215146)	3.06 (1.81-4.59)	-1.44 (-1.5--1.38)
Male	177737 (108959-258945)	11.91 (7.35-17.49)	372840 (228453-545265)	9.99 (6.16-14.8)	-0.68 (-0.74--0.61)
**Age**					
40-44 years	1153 (422-2349)	0.4 (0.15-0.82)	1237 (451-2600)	0.25 (0.09-0.52)	-1.63 (-1.66--1.6)
45-49 years	5888 (2908-10268)	2.54 (1.25-4.42)	8061 (3864-14186)	1.7 (0.82-3)	-1.34 (-1.38--1.3)
50-54 years	13463 (7236-21403)	6.33 (3.4-10.07)	21062 (11342-33575)	4.73 (2.55-7.55)	-0.97 (-0.99--0.94)
55-59 years	21647 (12245-33142)	11.69 (6.61-17.9)	38323 (21888-59387)	9.68 (5.53-15.01)	-0.59 (-0.64--0.54)
60-64 years	31634 (18130-50000)	19.7 (11.29-31.13)	51186 (29108-82624)	15.99 (9.09-25.82)	-0.62 (-0.69--0.55)
65-69 years	38892 (22634-60039)	31.46 (18.31-48.57)	72776 (42440-111310)	26.38 (15.39-40.35)	-0.66 (-0.76--0.55)
70-74 years	41297 (23990-63403)	48.78 (28.34-74.89)	81784 (45556-125511)	39.73 (22.13-60.98)	-0.8 (-0.89--0.71)
75-79 years	43041 (25864-65997)	69.92 (42.02-107.21)	78878 (46418-125283)	59.81 (35.2-94.99)	-0.67 (-0.72--0.63)
80-84 years	36122 (20994-552	102.11 (59.34-156.	74542 (42929-113	85.11 (49.01-129	-0.81 (-0.87--0

	72)	24)	784)	.92)	.74)
85-89 years	23092 (13595-35277)	152.81 (89.97-233.45)	55464 (32996-86765)	121.31 (72.17-189.77)	-1.01 (-1.08--0.94)
90-94 years	8442 (5037-13282)	197.01 (117.53-309.95)	25197 (14759-40026)	140.85 (82.5-223.74)	-1.29 (-1.35--1.23)
95+ years	2223 (1332-3423)	218.31 (130.83-336.26)	7373 (4297-11796)	135.28 (78.84-216.42)	-1.7 (-1.77--1.64)
**Regional levels**					
High-middle SDI	65779 (39935-95722)	7.18 (4.38-10.54)	150460 (89486-221640)	7.55 (4.5-11.14)	0.05 (0.02-0.08)
High SDI	106575 (64112-158855)	9.53 (5.75-14.27)	145973 (88322-217525)	6.5 (3.94-9.64)	-1.31 (-1.36--1.25)
Low-middle SDI	25208 (15292-36799)	5.37 (3.31-7.89)	48836 (30239-72916)	4.01 (2.47-6.08)	-1.06 (-1.1--1.03)
Low SDI	5171 (3176-7555)	3.14 (1.92-4.62)	9209 (5510-13664)	2.35 (1.45-3.55)	-1.07 (-1.15--0.99)
Middle SDI	63878 (39162-92208)	7.55 (4.71-11.04)	161022 (98089-237964)	6.47 (3.97-9.65)	-0.74 (-0.81--0.67)
World Bank High Income	122414 (73774-182798)	9.37 (5.66-13.97)	158113 (95848-234314)	6.22 (3.78-9.17)	-0.62 (-1.31-0.07)
World Bank Low Income	3410 (2071-5014)	3.1 (1.91-4.59)	6131 (3684-9123)	2.35 (1.41-3.46)	-0.82 (-2.28-0.66)
World Bank Lower Middle Income	42210 (26050-61615)	5.2 (3.21-7.62)	83275 (50675-122417)	3.96 (2.41-5.94)	-0.35 (-1.3-0.61)
World Bank Upper Middle Income	98577 (60242-142233)	7.63 (4.7-11.27)	267979 (161984-396128)	7.83 (4.73-11.64)	1.12 (0.75-1.49)
Advanced Health System	137205 (83178-204168)	8.41 (5.11-12.54)	180208 (109409-267722)	5.94 (3.6-8.73)	-0.42 (-1.03-0.21)
Africa	8160 (4943-12079)	3.74 (2.27-5.61)	14653 (8900-21968)	2.87 (1.76-4.3)	-0.79 (-2.19-0.64)

African Region	5013 (3054-7451)	2.93 (1.79-4.36)	8196 (4888-12221)	2.02 (1.22-3.01)	-1.19 (-2.64-0.28)
America	59491 (35511-89426)	9.94 (5.95-15)	83528 (50026-125500)	6.08 (3.64-9.11)	-0.99 (-1.2--0.78)
Andean Latin America	427 (254-650)	2.34 (1.41-3.6)	1066 (635-1608)	1.88 (1.12-2.84)	0.26 (-0.38-0.91)
Asia	122534 (74963-176421)	7.45 (4.59-10.94)	323094 (196254-476426)	6.78 (4.12-10.09)	0.65 (0.1-1.2)
Australasia	1589 (950-2410)	6.74 (4.05-10.29)	2304 (1363-3463)	4.09 (2.46-6.1)	-0.9 (-1.45--0.36)
Basic Health System	102681 (62911-148320)	8.33 (5.14-12.25)	283263 (171154-418529)	8.03 (4.85-11.94)	0.92 (0.41-1.44)
Caribbean	1303 (783-1928)	5.31 (3.19-7.82)	2211 (1332-3355)	4.06 (2.44-6.16)	-0.3 (-0.58--0.02)
Central Africa	357 (216-538)	1.57 (0.96-2.35)	618 (366-924)	1.1 (0.65-1.65)	-1.14 (-2.62-0.37)
Central Asia	1609 (979-2378)	3.66 (2.23-5.47)	2920 (1780-4296)	4.05 (2.49-6.01)	0.98 (0.41-1.54)
Central Europe	9931 (6013-14486)	6.92 (4.2-10.14)	12223 (7337-17931)	5.4 (3.24-7.9)	0.12 (-0.47-0.71)
Central Latin America	3467 (2109-5070)	4.87 (2.99-7.07)	5981 (3568-8761)	2.49 (1.49-3.63)	-1 (-1.7--0.29)
Central Sub-Saharan Africa	316 (188-473)	1.78 (1.07-2.64)	664 (392-1005)	1.43 (0.85-2.14)	-0.55 (-2.06-0.99)
Commonwealth High Income	16627 (10047-24971)	10.71 (6.49-16.24)	17898 (10657-26659)	6.14 (3.64-9.07)	-1.42 (-2.09--0.74)
Commonwealth Low Income	3119 (1907-4587)	4.58 (2.8-6.71)	7346 (4544-10678)	3.86 (2.37-5.67)	0.2 (-1.19-1.61)
Commonwealth Middle Income	19687 (12091-287	4.28 (2.63-6.34)	37487 (22660-565	2.91 (1.76-4.42	-0.71 (-1.72-0.

	60)		56)	)	31)
East Asia	66908 (41105-96851)	9.6 (5.89-14.08)	211849 (127635-314995)	10.09 (6.08-15.1)	1.46 (1.01-1.91)
Eastern Africa	1691 (1008-2487)	3.33 (2-4.88)	2923 (1742-4402)	2.35 (1.39-3.48)	-1.06 (-2.7-0.61)
Eastern Europe	10675 (6556-15403)	3.94 (2.42-5.72)	15404 (9297-22676)	4.32 (2.6-6.3)	1.01 (0.48-1.56)
Eastern Mediterranean Region	9373 (5713-13820)	6.54 (4.02-9.7)	18614 (11254-27929)	5.2 (3.17-7.83)	-0.4 (-1.59-0.8)
Eastern Sub-Saharan Africa	1558 (944-2302)	2.87 (1.77-4.22)	2563 (1534-3815)	1.93 (1.16-2.86)	-1.23 (-2.9-0.47)
Europe	76276 (46880-111842)	7.38 (4.53-10.84)	93932 (57202-138836)	5.55 (3.38-8.2)	-0.21 (-0.86-0.44)
European Region	77573 (47658-113764)	7.26 (4.46-10.68)	96169 (58531-142187)	5.47 (3.33-8.09)	-0.24 (-0.83-0.35)
High-income Asia Pacific	14735 (8870-21963)	7.75 (4.67-11.51)	27349 (16671-41171)	5.26 (3.22-7.79)	0.25 (-0.64-1.14)
High-income North America	43724 (26066-66127)	12.12 (7.24-18.27)	54348 (32133-80927)	7.91 (4.76-11.77)	-0.94 (-1.5--0.39)
Limited Health System	25861 (15826-37711)	4.43 (2.71-6.56)	50685 (31041-76131)	3.13 (1.91-4.72)	-0.6 (-1.71-0.52)
Minimal Health System	864 (528-1295)	1.84 (1.14-2.75)	1343 (809-2019)	1.35 (0.81-2.01)	-1.2 (-2.74-0.36)
North Africa and Middle East	10642 (6480-15990)	7.92 (4.86-11.92)	23191 (13950-34674)	6.11 (3.67-9.12)	-0.18 (-1.19-0.83)
North America	43722 (26064-66124)	12.12 (7.24-18.27)	54346 (32131-80924)	7.91 (4.76-11.77)	-0.94 (-1.5--0.39)
Northern Africa	3581 (2173-5455)	7.47 (4.48-11.33)	7819 (4763-11749)	6.46 (3.97-9.75)	0.14 (-0.9-1.19)
Oceania	104	4.25	233	3.57	-0.3

	(60-155)	(2.53-6.43)	(137-361)	(2.08-5.51)	(-1.51-0.93)
Region of the Americas	59491 (35511-89426)	9.94 (5.95-15)	83528 (50026-125500)	6.08 (3.64-9.11)	-0.99 (-1.2--0.78)
South-East Asia Region	26406 (16333-38658)	5.12 (3.15-7.58)	57408 (35287-85429)	3.73 (2.28-5.67)	-0.14 (-1.05-0.78)
South Asia	19288 (11801-28001)	4.63 (2.83-6.82)	39883 (24350-60237)	3.26 (1.99-4.95)	-0.42 (-1.13-0.28)
Southeast Asia	15144 (9369-22047)	7.38 (4.55-10.86)	30228 (18279-44344)	5.26 (3.2-7.75)	-0.22 (-1-0.56)
Southern Africa	1832 (1114-2766)	5.32 (3.27-7.97)	2086 (1253-3195)	2.61 (1.59-4.01)	-2.09 (-3.31--0.85)
Southern Latin America	2232 (1309-3280)	4.9 (2.89-7.18)	3465 (2018-5207)	3.93 (2.29-5.87)	-0.15 (-0.34-0.04)
Southern Sub-Saharan Africa	1440 (881-2180)	6.32 (3.9-9.56)	1369 (832-2104)	2.69 (1.65-4.18)	-2.2 (-3.1--1.29)
Tropical Latin America	8533 (5161-12771)	10.9 (6.64-16.25)	16819 (10026-26232)	6.7 (3.98-10.47)	-0.54 (-1.06--0.02)
Western Africa	698 (417-1024)	1.08 (0.64-1.58)	1207 (715-1795)	0.81 (0.49-1.2)	-1.12 (-2.51-0.29)
Western Europe	52472 (31955-77671)	8.72 (5.32-12.93)	60436 (36438-89446)	5.88 (3.56-8.74)	-0.66 (-1.53-0.23)
Western Pacific Region	87882 (53843-126616)	9.05 (5.6-13.39)	249628 (151146-371545)	8.79 (5.31-13.1)	1.12 (0.75-1.49)
Western Sub-Saharan Africa	797 (482-1169)	1.11 (0.67-1.63)	1378 (822-2044)	0.83 (0.5-1.23)	-1.15 (-2.54-0.26)
**Country levels**					
Afghanistan	177 (104-278)	3.23 (1.89-5.06)	224 (133-342)	3.16 (1.9-4.91)	-1.19 (-2.7-0.35)
Albania	164 (99-241)	9.42 (5.73-14.13	472 (274-703)	10.8 (6.36-16.0	2.65 (2.14-3.1

		)		3)	7)
Algeria	909 (534-1396)	10.65 (6.16-16.68)	2219 (1307-3289)	7.96 (4.73-11.88)	0.11 (-0.95-1.19)
American Samoa	1 (1-1)	4.47 (2.66-6.79)	2 (1-3)	4.09 (2.48-6.27)	1.02 (0.15-1.9)
Andorra	4 (2-7)	7.89 (4.62-12.45)	10 (6-15)	6.05 (3.45-9.31)	0.02 (-0.49-0.54)
Angola	91 (54-136)	3.1 (1.83-4.72)	248 (143-381)	2.6 (1.49-3.99)	-0.41 (-2.14-1.34)
Antigua and Barbuda	2 (1-2)	2.68 (1.58-4.06)	3 (1-4)	2.49 (1.4-3.7)	0.03 (-0.26-0.33)
Argentina	1689 (997-2508)	5.29 (3.14-7.82)	2487 (1458-3818)	4.4 (2.59-6.73)	-0.16 (-0.33-0.01)
Armenia	158 (94-233)	6.48 (3.87-9.59)	321 (193-485)	7.27 (4.4-10.97)	2.01 (1.66-2.35)
Australia	1259 (748-1931)	6.41 (3.83-9.87)	1770 (1053-2701)	3.74 (2.24-5.69)	-1.01 (-1.57--0.46)
Austria	667 (409-993)	5.45 (3.27-8.06)	1212 (697-1821)	6.33 (3.71-9.49)	1.08 (0.23-1.93)
Azerbaijan	213 (130-327)	4.58 (2.81-7.07)	526 (319-788)	6.16 (3.8-9.17)	1.73 (1.17-2.29)
Bahamas	4 (2-6)	2.58 (1.52-4)	9 (5-14)	2.53 (1.4-3.84)	0.94 (0.38-1.51)
Bahrain	9 (5-14)	7.51 (4.35-11.72)	43 (25-65)	6.39 (3.69-9.81)	0.17 (-1.55-1.93)
Bangladesh	2056 (1248-3010)	5.4 (3.3-8)	5528 (3432-8092)	4.56 (2.81-6.67)	0.91 (-0.29-2.13)
Barbados	8 (5-12)	2.47 (1.47-3.72)	10 (6-16)	1.95 (1.14-3.02)	-0.55 (-1.04--0.05)
Belarus	680	5.36	843	5.14	0.7

	(402-1027)	(3.15-8.16)	(510-1233)	(3.13-7.54)	(0.15-1.25)
Belgium	1714 (1020-2582)	10.69 (6.31-16.07)	1771 (1049-2722)	6.91 (4.1-10.55)	-0.9 (-1.77--0.02)
Belize	3 (2-5)	3.65 (2.22-5.54)	8 (5-12)	3.03 (1.85-4.61)	-0.09 (-1.03-0.85)
Benin	24 (14-37)	1.3 (0.76-1.98)	34 (19-52)	0.73 (0.41-1.12)	-2.13 (-3.52--0.72)
Bermuda	2 (1-3)	3.44 (1.97-5.24)	5 (3-8)	3.67 (2.11-5.69)	1.62 (1.03-2.22)
Bhutan	5 (3-7)	2.75 (1.49-4.42)	13 (7-20)	2.31 (1.3-3.6)	0.98 (-0.14-2.11)
Bolivia (Plurinational State of)	63 (38-95)	2.28 (1.36-3.38)	149 (86-222)	1.8 (1.06-2.67)	0.38 (-0.46-1.24)
Bosnia and Herzegovina	276 (163-408)	7.82 (4.72-11.63)	516 (316-772)	8.11 (4.99-12.08)	1.74 (1.15-2.34)
Botswana	24 (14-36)	5.58 (3.33-8.49)	46 (27-71)	3.87 (2.26-5.89)	-0.5 (-1.77-0.77)
Brazil	8283 (5004-12376)	10.83 (6.59-16.1)	16400 (9798-25543)	6.67 (3.98-10.41)	-0.52 (-1.04-0)
Brunei Darussalam	6 (4-10)	7.45 (4.55-11.42)	12 (7-18)	4.14 (2.43-6.41)	-0.8 (-2.06-0.47)
Bulgaria	756 (450-1120)	6.24 (3.73-9.26)	623 (360-923)	4.41 (2.54-6.52)	-0.54 (-1.31-0.24)
Burkina Faso	33 (19-49)	0.87 (0.51-1.31)	57 (32-88)	0.69 (0.4-1.05)	-0.88 (-2.2-0.45)
Burundi	44 (26-66)	2.31 (1.4-3.52)	49 (28-77)	1.21 (0.69-1.89)	-2.12 (-3.69--0.53)
Cabo Verde	3 (2-5)	1.34 (0.79-2)	3 (2-5)	0.71 (0.42-1.11)	-1.85 (-2.45--1.23)

Cambodia	285 (177-425)	8.37 (5.21-12.63)	753 (434-1147)	7.56 (4.36-11.42)	0.42 (-0.8-1.66)
Cameroon	49 (28-75)	1.29 (0.75-1.97)	94 (52-146)	0.9 (0.51-1.41)	-1.25 (-2.72-0.24)
Canada	4589 (2766-7024)	14.14 (8.52-21.62)	6349 (3697-9629)	8.36 (4.89-12.6)	-1.11 (-1.74--0.49)
Central African Republic	18 (11-28)	2.08 (1.25-3.19)	24 (14-37)	1.44 (0.85-2.16)	-1.31 (-2.98-0.38)
Chad	40 (23-61)	1.61 (0.94-2.47)	57 (31-89)	1.25 (0.69-1.94)	-1.74 (-3.25--0.21)
Chile	357 (215-541)	3.66 (2.2-5.56)	725 (425-1077)	2.8 (1.64-4.17)	0.08 (-0.19-0.35)
China	65545 (40297-94833)	9.8 (6-14.4)	208981 (125851-310724)	10.32 (6.22-15.46)	1.47 (1.02-1.93)
Colombia	573 (351-862)	3.66 (2.23-5.51)	1298 (777-1985)	2.32 (1.38-3.54)	-0.1 (-0.66-0.47)
Comoros	5 (3-7)	3.13 (1.91-4.95)	9 (5-14)	2.28 (1.27-3.58)	-0.27 (-1.51-0.98)
Congo	17 (11-26)	1.99 (1.21-3.02)	51 (30-76)	2.4 (1.4-3.55)	1 (-0.42-2.45)
Cook Islands	1 (0-1)	4.59 (2.56-7.09)	1 (1-2)	3.94 (2.29-5.93)	0.87 (0.48-1.27)
Costa Rica	88 (53-131)	5.44 (3.27-8.13)	201 (121-307)	3.64 (2.2-5.58)	0.04 (-0.49-0.56)
Cmte d'Ivoire	48 (28-72)	1.56 (0.91-2.39)	109 (63-168)	1.18 (0.7-1.79)	-0.94 (-2.62-0.77)
Croatia	535 (317-807)	9.56 (5.59-14.65)	769 (461-1163)	8.04 (4.85-12.02)	1.05 (0.31-1.79)
Cuba	664 (397-1004)	6.63 (3.94-10.01	952 (562-1428)	4.67 (2.74-6.99	-0.38 (-0.81-0.

		)		)	06)
Cyprus	57 (34-87)	7.54 (4.59-11.3)	146 (85-218)	6.86 (4.04-10.16)	0.44 (0.14-0.74)
Czechia	1021 (615-1554)	7.41 (4.48-11.3)	1415 (837-2123)	6.35 (3.78-9.51)	0.23 (-0.46-0.92)
Democratic People's Republic of Korea	644 (389-947)	4.73 (2.82-6.93)	1210 (685-1824)	3.83 (2.19-5.77)	0.04 (-0.27-0.35)
Democratic Republic of the Congo	179 (105-275)	1.42 (0.86-2.14)	320 (188-499)	0.99 (0.6-1.56)	-1 (-2.45-0.48)
Denmark	1221 (745-1820)	13.95 (8.49-20.71)	959 (584-1435)	7.33 (4.43-10.98)	-1.94 (-2.77--1.11)
Djibouti	4 (2-6)	4.51 (2.65-6.82)	17 (10-26)	3.65 (2.13-5.61)	0.28 (-1.47-2.06)
Dominica	1 (1-2)	2.24 (1.31-3.37)	2 (1-2)	1.93 (1.09-2.98)	0.16 (-0.06-0.38)
Dominican Republic	239 (142-371)	7.8 (4.54-12.18)	588 (346-950)	6.17 (3.62-10.01)	0.16 (-0.53-0.85)
Ecuador	160 (91-248)	3.36 (1.93-5.21)	276 (161-418)	1.74 (1.02-2.63)	-1.08 (-1.72--0.44)
Egypt	1503 (912-2313)	8.23 (4.89-12.75)	3515 (2158-5419)	8.61 (5.27-13.32)	0.47 (-0.94-1.91)
El Salvador	57 (33-86)	2.02 (1.18-3.07)	134 (77-205)	2.12 (1.22-3.23)	1.37 (0.86-1.89)
Equatorial Guinea	3 (2-5)	1.97 (1.12-3.16)	7 (4-10)	1.65 (0.94-2.55)	-0.91 (-2.55-0.76)
Eritrea	9 (5-16)	0.99 (0.55-1.66)	17 (10-27)	0.74 (0.42-1.18)	-0.65 (-2.26-0.99)
Estonia	94 (57-142)	4.55 (2.73-6.84)	137 (81-207)	4.98 (2.89-7.43)	1.26 (0.5-2.02)
Eswatini	7 (4-11)	3.33	8 (4-12)	2.1	-1.17

		(1.82-5.25)		(1.15-3.39)	(-2.9-0.59)
Ethiopia	173 (100-259)	1 (0.61-1.47)	322 (193-485)	0.85 (0.51-1.31)	-0.06 (-1.59-1.49)
Fiji	13 (8-20)	4.25 (2.54-6.39)	23 (14-34)	3.18 (1.87-4.64)	0.2 (-0.63-1.04)
Finland	338 (194-516)	4.69 (2.7-7.11)	538 (314-813)	4.02 (2.34-6.05)	0.53 (-0.32-1.4)
France	5237 (3194-7856)	5.92 (3.56-8.8)	6452 (3765-9676)	4.31 (2.56-6.32)	-0.44 (-1.27-0.41)
Gabon	7 (4-11)	1.43 (0.85-2.16)	14 (8-22)	1.49 (0.85-2.34)	0.15 (-0.84-1.14)
Gambia	6 (4-10)	2.08 (1.25-3.17)	10 (6-14)	1.11 (0.65-1.69)	-1.93 (-3.31--0.52)
Georgia	272 (162-408)	4.43 (2.65-6.69)	362 (215-539)	5.75 (3.42-8.51)	2.12 (1.54-2.71)
Germany	12473 (7568-18548)	9.42 (5.73-14.06)	15686 (9423-23120)	7.29 (4.4-10.72)	-0.02 (-1-0.96)
Ghana	57 (33-89)	1.31 (0.77-2.04)	151 (85-230)	1.23 (0.69-1.85)	0.57 (-0.94-2.1)
Greece	1770 (1053-2639)	11.65 (6.94-17.3)	2304 (1372-3519)	8.33 (4.99-12.38)	0.03 (-0.88-0.96)
Greenland	3 (2-5)	12.39 (7.23-18.73)	7 (4-10)	10.21 (6.18-15.57)	0.7 (0.16-1.25)
Grenada	2 (1-3)	2.37 (1.38-3.59)	2 (1-3)	1.73 (1.05-2.64)	-0.92 (-1.25--0.58)
Guam	2 (1-4)	3.35 (1.93-5.22)	8 (4-12)	3.46 (2-5.38)	1.79 (1.28-2.3)
Guatemala	87 (51-129)	3.26 (1.94-4.86)	255 (149-385)	2.53 (1.49-3.82)	0.41 (-0.78-1.61)

Guinea	49 (29-74)	1.68 (1.01-2.55)	65 (38-100)	1.34 (0.79-2.07)	-1.23 (-2.31--0.13)
Guinea-Bissau	2 (1-3)	0.65 (0.37-1)	4 (2-6)	0.68 (0.38-1.05)	0.31 (-1.33-1.97)
Guyana	8 (5-13)	2.58 (1.51-3.89)	12 (7-18)	2.11 (1.23-3.2)	0.44 (-0.44-1.33)
Haiti	48 (28-72)	1.79 (1.06-2.71)	78 (45-120)	1.36 (0.79-2.07)	-0.81 (-1.97-0.35)
Honduras	77 (45-118)	4.32 (2.52-6.67)	203 (118-320)	3.67 (2.19-5.75)	0.07 (-1.01-1.17)
Hungary	938 (558-1404)	6.39 (3.81-9.49)	929 (564-1406)	4.72 (2.87-7.05)	-0.37 (-1.08-0.36)
Iceland	36 (21-56)	12.08 (7.13-18.45)	43 (25-67)	6.83 (4.01-10.63)	-1.44 (-1.93--0.96)
India	13888 (8471-20449)	4.28 (2.63-6.43)	30197 (18243-45443)	3.06 (1.83-4.67)	-0.32 (-1.29-0.66)
Indonesia	4650 (2843-6916)	6.21 (3.78-9.17)	11735 (7078-17660)	6.16 (3.75-9.29)	0.73 (-0.2-1.67)
Iran (Islamic Republic of)	1066 (635-1589)	4.91 (2.93-7.38)	3393 (1967-5202)	4.82 (2.75-7.42)	1.46 (0.59-2.35)
Iraq	862 (530-1296)	12.27 (7.51-18.4)	1460 (866-2208)	8.17 (4.87-12.34)	-1.08 (-2.41-0.27)
Ireland	562 (336-846)	13.87 (8.32-20.65)	541 (318-823)	6.46 (3.77-9.81)	-2.23 (-2.62--1.84)
Israel	375 (217-578)	7.88 (4.58-12.11)	635 (373-950)	4.88 (2.88-7.3)	-1.05 (-1.25--0.84)
Italy	7721 (4702-11262)	8.55 (5.22-12.38)	9476 (5664-14350)	5.88 (3.54-8.84)	-0.51 (-1.51-0.49)
Jamaica	85 (49-130)	4.67 (2.69-7.09)	110 (65-171)	3.47 (2.06-5.4)	-0.57 (-0.77--0

					.38)
Japan	12636 (7636-18755)	7.68 (4.64-11.37)	20700 (12605-31004)	5.11 (3.13-7.61)	0.16 (-0.92-1.25)
Jordan	121 (74-182)	12.68 (7.68-19.13)	564 (345-823)	9.99 (6.15-14.59)	0.11 (-1.47-1.71)
Kazakhstan	385 (236-577)	3.19 (1.99-4.79)	503 (307-744)	2.93 (1.78-4.39)	0.24 (-0.06-0.53)
Kenya	218 (130-323)	3.29 (2-5.07)	323 (199-482)	1.72 (1.06-2.6)	-2.08 (-3.64--0.5)
Kiribati	2 (1-4)	8.14 (4.95-12.38)	5 (3-8)	8.97 (5.42-13.38)	0.41 (-0.81-1.64)
Kuwait	39 (23-59)	8.79 (5.16-13.41)	168 (102-257)	7.45 (4.45-11.36)	0.16 (-1.17-1.5)
Kyrgyzstan	119 (72-179)	4.29 (2.61-6.49)	275 (163-405)	6.72 (3.99-9.88)	2.29 (1.51-3.07)
Lao People's Democratic Republic	114 (69-178)	7.13 (4.3-10.97)	251 (149-373)	6.89 (4.13-10.2)	0.4 (-0.83-1.63)
Latvia	162 (99-239)	4.5 (2.74-6.63)	159 (95-237)	3.93 (2.34-5.86)	0.57 (-0.18-1.32)
Lebanon	215 (128-322)	11.57 (6.87-17.26)	848 (515-1285)	13.37 (8.11-20.29)	1.38 (1-1.77)
Lesotho	33 (19-53)	4.86 (2.68-8.03)	38 (22-60)	5.06 (2.79-8.2)	0.05 (-1.08-1.18)
Liberia	11 (7-17)	1.1 (0.66-1.61)	15 (9-23)	0.79 (0.44-1.19)	-1.94 (-3.21--0.66)
Libya	113 (67-169)	6.85 (4.08-10.23)	238 (140-359)	5.46 (3.15-8.3)	0.1 (-0.93-1.14)
Lithuania	216 (128-323)	4.77 (2.85-7.17)	243 (145-363)	3.95 (2.35-5.95)	0.52 (-0.19-1.24)
Luxembourg	32 (17-51)	5.82	50 (28-77)	4.54	-0.57

		(3.11-9.12)		(2.53-7.03)	(-1.17-0.02)
Madagascar	107 (64-158)	2.88 (1.72-4.33)	94 (57-140)	1.21 (0.71-1.84)	-3.12 (-4.86--1.34)
Malawi	102 (60-150)	3.63 (2.17-5.41)	173 (98-259)	2.95 (1.73-4.47)	-0.68 (-2.29-0.97)
Malaysia	556 (343-837)	6.98 (4.21-10.55)	1104 (662-1647)	4.59 (2.78-6.87)	-0.53 (-1.42-0.38)
Maldives	7 (4-10)	11.91 (7.53-17.61)	21 (13-31)	7.73 (4.79-11.42)	-0.28 (-1.59-1.06)
Mali	35 (21-54)	1.19 (0.7-1.83)	119 (69-175)	1.81 (1.07-2.74)	1.35 (-0.28-3.01)
Malta	30 (18-45)	7.19 (4.3-10.84)	47 (27-70)	4.55 (2.6-6.79)	-0.47 (-1.15-0.21)
Marshall Islands	0 (0-1)	3.04 (1.66-4.75)	1 (0-1)	3.13 (1.81-4.76)	0.99 (-0.56-2.57)
Mauritania	11 (6-16)	1.2 (0.72-1.78)	18 (11-28)	0.92 (0.53-1.38)	-0.85 (-1.96-0.26)
Mauritius	33 (21-50)	5.28 (3.24-7.84)	90 (55-138)	5.04 (3.08-7.65)	1.46 (0.93-2)
Mexico	1978 (1229-2901)	5.66 (3.54-8.31)	2649 (1560-3924)	2.25 (1.33-3.36)	-1.84 (-2.6--1.08)
Micronesia (Federated States of)	2 (1-4)	5.39 (3.25-8.13)	3 (2-5)	5.05 (2.96-7.77)	0.54 (-0.5-1.59)
Monaco	6 (3-10)	8.01 (4.62-13.22)	7 (4-11)	6.1 (3.33-9.69)	-0.82 (-2.13-0.5)
Mongolia	41 (24-61)	4.27 (2.49-6.31)	103 (62-152)	5.29 (3.2-7.94)	1.54 (0.45-2.63)
Montenegro	52 (31-78)	8.66 (5.12-12.88)	75 (45-112)	7.53 (4.47-11.32)	0.32 (-0.13-0.77)

Morocco	576 (339-865)	4.54 (2.7-6.77)	838 (497-1263)	2.59 (1.53-3.89)	-1.05 (-1.73--0.37)
Mozambique	114 (68-171)	2.5 (1.51-3.82)	157 (93-235)	1.83 (1.06-2.76)	-1.31 (-2.88-0.28)
Myanmar	2047 (1232-3133)	11.85 (7.07-18.28)	2098 (1233-3201)	5.1 (2.94-7.78)	-2.03 (-2.85--1.2)
Namibia	29 (17-44)	6.92 (3.97-10.89)	44 (26-66)	4.5 (2.58-7.06)	-1.12 (-2.57-0.36)
Nauru	0 (0-0)	5.55 (3.25-8.53)	0 (0-0)	4.4 (2.61-6.73)	-0.5 (-1.77-0.79)
Nepal	606 (373-881)	9.28 (5.7-13.65)	1051 (636-1558)	5.64 (3.42-8.61)	-0.88 (-2.01-0.26)
Netherlands	2084 (1249-3123)	10.14 (6.08-15.12)	3012 (1794-4505)	7.83 (4.66-11.7)	0.38 (-0.37-1.13)
New Zealand	329 (200-487)	8.35 (5.09-12.35)	534 (313-809)	5.98 (3.5-9.03)	-0.44 (-0.93-0.06)
Nicaragua	48 (28-74)	3.59 (2.1-5.53)	141 (82-222)	3.16 (1.82-5.05)	1 (-0.03-2.05)
Niger	16 (9-23)	0.82 (0.47-1.22)	43 (25-66)	0.78 (0.43-1.24)	-0.39 (-2.38-1.64)
Nigeria	319 (184-481)	0.9 (0.53-1.35)	443 (257-667)	0.56 (0.33-0.84)	-1.88 (-3.24--0.51)
Niue	0 (0-0)	3.38 (1.96-5.08)	0 (0-0)	3.26 (1.87-5.03)	0.3 (0.02-0.59)
North Macedonia	152 (93-231)	8.84 (5.45-13.61)	232 (138-344)	7.67 (4.64-11.45)	0.32 (0.1-0.55)
Northern Mariana Islands	1 (0-1)	4.63 (2.76-7.22)	2 (1-3)	3.82 (2.19-6.03)	1.68 (0.42-2.95)
Norway	714 (432-1093)	9.74 (5.89-14.76	497 (292-771)	4.66 (2.77-7.19	-2.75 (-3.54--1

		)		)	.95)
Oman	20 (12-30)	3.74 (2.22-5.63)	43 (25-65)	2.76 (1.55-4.2)	-0.59 (-2.2-1.05)
Pakistan	2732 (1674-3992)	5.77 (3.52-8.42)	3095 (1873-4680)	3.2 (1.96-5.01)	-2.14 (-3.33--0.95)
Palau	0 (0-0)	3.7 (2.16-5.64)	1 (0-1)	3.48 (2-5.59)	0.82 (0.22-1.42)
Palestine	64 (37-94)	8.76 (5.06-12.99)	146 (87-227)	7.1 (4.27-10.96)	-0.66 (-2.14-0.83)
Panama	63 (38-95)	4.68 (2.78-7.12)	133 (77-207)	2.96 (1.72-4.63)	-0.73 (-1.16--0.29)
Papua New Guinea	58 (32-88)	3.8 (2.12-5.78)	144 (81-231)	3.28 (1.82-5.25)	-0.41 (-1.73-0.92)
Paraguay	250 (147-381)	12.7 (7.42-19.35)	420 (248-666)	7.88 (4.66-12.45)	-1.02 (-1.73--0.31)
Peru	204 (123-309)	1.9 (1.14-2.87)	641 (378-971)	1.95 (1.15-2.95)	0.96 (0.37-1.55)
Philippines	2287 (1374-3402)	10.1 (6.09-15.08)	3950 (2386-5899)	5.68 (3.41-8.55)	-1.13 (-2.19--0.05)
Poland	3419 (2092-4945)	8.01 (4.91-11.73)	3930 (2355-5759)	5.38 (3.24-7.89)	-0.39 (-0.93-0.15)
Portugal	621 (373-920)	4.37 (2.63-6.47)	701 (407-1065)	2.81 (1.64-4.25)	-0.74 (-1.56-0.08)
Puerto Rico	142 (79-225)	3.94 (2.21-6.25)	269 (154-427)	3.36 (1.96-5.31)	0.79 (0.15-1.44)
Qatar	4 (2-6)	4.56 (2.58-7.23)	31 (19-50)	3.98 (2.41-6.33)	-0.03 (-2-1.98)
Republic of Korea	2026 (1242-3047)	9.36 (5.55-14.6)	6428 (3859-9786)	6.8 (4.1-10.32)	0.87 (0.32-1.43)
Republic of	157	3.79	266	4.38	1.65

Moldova	(92-237)	(2.21-5.8)	(161-396)	(2.66-6.53)	(1.26-2.04)
Romania	1352 (828-2031)	5.16 (3.15-7.82)	1432 (841-2121)	3.8 (2.22-5.64)	-0.23 (-0.82-0.37)
Russian Federation	6121 (3758-8864)	3.47 (2.11-5.06)	10834 (6576-16055)	4.47 (2.69-6.6)	1.53 (1-2.05)
Rwanda	120 (70-184)	6.4 (3.61-10.04)	303 (165-467)	6.87 (3.8-10.8)	0.81 (-0.99-2.65)
Saint Kitts and Nevis	1 (0-1)	2.04 (1.17-3.25)	1 (1-2)	1.62 (0.96-2.55)	-0.97 (-1.2--0.74)
Saint Lucia	3 (1-4)	2.98 (1.74-4.5)	5 (3-8)	2.2 (1.26-3.31)	0.11 (-0.28-0.51)
Saint Vincent and the Grenadines	2 (1-2)	2.19 (1.33-3.23)	3 (2-5)	2.27 (1.35-3.38)	1.19 (0.83-1.54)
Samoa	4 (3-7)	5.63 (3.42-8.44)	7 (4-10)	5.26 (3.12-7.97)	0.09 (-0.76-0.95)
San Marino	3 (2-5)	8.1 (4.8-12.46)	5 (3-8)	5.74 (3.33-8.77)	-0.42 (-1.4-0.57)
Sao Tome and Principe	0 (0-1)	0.76 (0.44-1.16)	1 (0-1)	0.66 (0.38-1.03)	-1.02 (-2.09-0.05)
Saudi Arabia	158 (90-246)	3.2 (1.84-4.99)	487 (262-757)	3.15 (1.75-4.88)	0.3 (-1.15-1.77)
Senegal	39 (23-60)	1.28 (0.77-1.93)	63 (36-98)	0.85 (0.49-1.33)	-1.15 (-2.28-0)
Serbia	673 (393-1025)	6.38 (3.68-9.72)	990 (594-1498)	5.91 (3.54-8.82)	0.69 (0.08-1.31)
Seychelles	3 (2-5)	6.21 (3.72-9.48)	6 (3-8)	5.24 (3.15-7.91)	-0.28 (-0.56-0)
Sierra Leone	29 (17-44)	1.56 (0.94-2.35)	37 (22-58)	1.08 (0.63-1.65)	-1.64 (-2.81--0.45)

Singapore	67 (39-102)	3.53 (2.03-5.41)	209 (124-310)	2.44 (1.45-3.61)	0.11 (-0.33-0.55)
Slovakia	304 (174-464)	5.21 (2.99-7.88)	434 (257-643)	4.5 (2.66-6.65)	0.48 (0-0.96)
Slovenia	130 (75-195)	5.29 (3.05-7.87)	228 (134-342)	5.12 (2.98-7.66)	1.2 (0.48-1.92)
Solomon Islands	7 (4-10)	5.98 (3.53-8.93)	16 (10-24)	5.58 (3.51-8.48)	0.38 (-0.96-1.74)
Somalia	39 (23-61)	2.39 (1.38-3.7)	73 (39-112)	1.68 (0.91-2.51)	-1.25 (-3.3-0.84)
South Africa	1176 (726-1765)	6.57 (4.07-9.88)	1024 (618-1583)	2.43 (1.44-3.8)	-2.59 (-3.34--1.83)
South Sudan	57 (33-89)	2.63 (1.53-4.18)	59 (35-96)	1.99 (1.18-3.26)	-1.35 (-2.94-0.27)
Spain	4033 (2397-5972)	7.26 (4.31-10.76)	4183 (2499-6180)	4.05 (2.42-6.06)	-1.2 (-1.99--0.39)
Sri Lanka	497 (299-756)	5.91 (3.57-9.04)	644 (389-972)	2.57 (1.55-3.87)	-1.66 (-2.21--1.11)
Sudan	442 (248-673)	5.93 (3.34-9.17)	785 (446-1232)	5.14 (2.92-8.23)	-0.61 (-1.96-0.75)
Suriname	12 (7-18)	5.16 (3.14-7.6)	20 (11-31)	3.27 (1.85-5.03)	-0.95 (-1.4--0.5)
Sweden	1497 (904-2280)	9 (5.46-13.7)	2011 (1159-3090)	8.22 (4.73-12.51)	0.11 (-0.91-1.15)
Switzerland	991 (598-1478)	8.88 (5.34-13.14)	1350 (805-2040)	6.59 (3.85-9.97)	-0.3 (-1.19-0.59)
Syrian Arab Republic	431 (268-637)	10.14 (6.29-15.02)	711 (432-1065)	6.8 (4.03-10.3)	-0.14 (-1.53-1.28)
Taiwan (Province of China)	719 (439-1070)	5.5 (3.36-8.13)	1658 (985-2471)	3.75 (2.24-5.56	0.56 (0.1-1.03

				)	)
Tajikistan	137 (81-207)	5.5 (3.21-8.41)	144 (82-222)	3.1 (1.76-4.72)	-1.62 (-2.79--0.43)
Thailand	1990 (1226-2980)	6.79 (4.24-10.27)	4869 (2982-7195)	4.41 (2.69-6.5)	0.55 (0.03-1.08)
Timor-Leste	15 (9-21)	7.61 (4.62-10.99)	42 (25-65)	5.87 (3.58-8.96)	0.37 (-1.02-1.78)
Togo	27 (15-42)	2.8 (1.6-4.26)	52 (28-83)	1.64 (0.89-2.64)	-1.17 (-2.76-0.45)
Tokelau	0 (0-0)	4.37 (2.6-6.68)	0 (0-0)	3.78 (2.19-5.94)	-0.05 (-0.22-0.13)
Tonga	4 (2-5)	7.73 (4.64-11.27)	5 (3-7)	6.13 (3.58-9.57)	-0.27 (-0.95-0.43)
Trinidad and Tobago	31 (18-46)	3.84 (2.26-5.82)	54 (32-85)	2.82 (1.63-4.36)	0.12 (-0.21-0.45)
Tunisia	470 (281-695)	11.5 (6.96-17.16)	991 (604-1465)	8.14 (4.93-12.06)	0.06 (-0.5-0.64)
Turkiye	3130 (1889-4687)	10.33 (6.18-15.68)	5521 (3283-8292)	6.18 (3.71-9.31)	-0.73 (-1.23--0.22)
Turkmenistan	73 (43-110)	4.36 (2.56-6.61)	107 (64-160)	3.03 (1.8-4.61)	-0.39 (-1.27-0.5)
Tuvalu	0 (0-0)	4.47 (2.58-6.95)	0 (0-1)	4.44 (2.53-6.87)	0.22 (-0.24-0.67)
Uganda	109 (63-169)	2.28 (1.32-3.6)	205 (123-321)	1.82 (1.05-2.85)	-1.28 (-3.15-0.62)
Ukraine	3244 (1966-4808)	4.66 (2.84-6.94)	2922 (1730-4462)	3.68 (2.19-5.59)	-0.21 (-0.83-0.42)
United Arab Emirates	15 (8-22)	5.09 (2.87-7.7)	141 (78-218)	6.36 (3.66-10.02)	0.71 (-1.99-3.49)
United Kingdom	10241	10.69	8748	6.01	-1.67

	(6216-15372)	(6.5-16.04)	(5139-13125)	(3.54-8.95)	(-2.51--0.82)
United Republic of Tanzania	371 (222-556)	4.51 (2.71-6.88)	619 (350-925)	2.93 (1.71-4.37)	-1.38 (-2.84-0.1)
United States of America	39131 (23248-59764)	11.93 (7.11-18.16)	47991 (28485-71481)	7.86 (4.7-11.69)	-0.93 (-1.48--0.38)
United States Virgin Islands	2 (1-4)	2.92 (1.6-4.9)	5 (3-8)	2.68 (1.57-4.21)	1.64 (1.09-2.19)
Uruguay	185 (108-269)	4.69 (2.77-6.82)	253 (145-379)	4.46 (2.59-6.64)	0.17 (-0.32-0.67)
Uzbekistan	212 (124-326)	1.92 (1.12-2.93)	580 (347-891)	2.53 (1.48-3.92)	1.43 (0.59-2.27)
Vanuatu	2 (1-3)	3.48 (2.1-5.19)	4 (2-6)	2.51 (1.49-3.89)	-0.77 (-2.07-0.56)
Venezuela (Bolivarian Republic of)	496 (285-751)	5.85 (3.35-9.07)	966 (542-1451)	3.35 (1.89-5.05)	-0.55 (-1.23-0.13)
Viet Nam	2637 (1591-3847)	7.18 (4.32-10.51)	4623 (2710-6779)	5.04 (2.98-7.42)	-0.69 (-1.29--0.08)
Yemen	312 (190-483)	8.05 (4.87-12.38)	802 (472-1200)	7.25 (4.31-10.93)	-0.15 (-1.76-1.5)
Zambia	86 (51-130)	4.07 (2.46-6.49)	139 (79-211)	2.87 (1.61-4.5)	-1.44 (-3.25-0.41)
Zimbabwe	171 (100-260)	5.61 (3.17-8.69)	208 (121-319)	4.37 (2.57-6.86)	-0.81 (-2.38-0.79)

Supplementary Table S4 The number of YLLs cases and corresponding ASR of smoking-attributable burden of ADOD stratified by sex, age , SDI region, and country with EAPC from 1990 to 2021 globally.

location	1990		2021		
	**Number_95%UI**	**ASR**	**Number_95%UI**	**ASR**	**EAPC_95%CI**
**Global**	528022 (122674-1511230)	15.76 (3.62-44.13)	1017331 (241832-2876261)	12.24 (2.9-34.28)	-0.05 (-0.44-0.33)
**Sex**					
Female	173153 (39948-486172)	9.15 (2.12-25.62)	280148 (65583-771699)	5.95 (1.4-16.41)	-1.53 (-1.59--1.48)
Male	354868 (80668-1025587)	25.54 (5.82-71.79)	737183 (177427-2115307)	20.58 (4.9-58.2)	-0.76 (-0.82--0.69)
**Age**					
40-44 years	554 (52-1932)	0.19 (0.02-0.67)	644 (58-2317)	0.13 (0.01-0.46)	-1.39 (-1.45--1.33)
45-49 years	4028 (543-13517)	1.73 (0.23-5.82)	5828 (754-19644)	1.23 (0.16-4.15)	-1.16 (-1.22--1.09)
50-54 years	15128 (2424-47459)	7.12 (1.14-22.33)	24156 (3708-75971)	5.43 (0.83-17.08)	-0.88 (-0.91--0.85)
55-59 years	35070 (5998-107327)	18.94 (3.24-57.95)	60254 (10137-193415)	15.23 (2.56-48.88)	-0.65 (-0.71--0.59)
60-64 years	62532 (12165-178915)	38.93 (7.57-111.4)	95417 (18566-290384)	29.81 (5.8-90.73)	-0.75 (-0.84--0.67)
65-69 years	81148 (17428-245999)	65.65 (14.1-199.01)	139593 (29059-399945)	50.61 (10.53-144.99)	-0.83 (-0.94--0.71)
70-74 years	78384 (17303-228012)	92.59 (20.44-269.32)	146482 (33328-446297)	71.16 (16.19-216.82)	-0.91 (-1.02--0.8)
75-79 years	78374 (17921-224580)	127.32 (29.11-364.84)	138671 (32669-386407)	105.15 (24.77-292.99)	-0.75 (-0.8--0.7)
80-84 years	82035 (19073-223	231.89 (53.91-632	163647 (38959-457	186.85 (44.48-521	-0.84 (-0.9--0.

	579)	.01)	175)	.99)	79)
85-89 years	56441 (13126-154463)	373.51 (86.86-1022.19)	134673 (31543-360814)	294.55 (68.99-789.15)	-0.97 (-1.03--0.91)
90-94 years	25480 (5890-68964)	594.62 (137.44-1609.35)	77465 (18236-203473)	433.02 (101.94-1137.4)	-1.18 (-1.22--1.13)
95+ years	8848 (2073-23339)	869.09 (203.62-2292.48)	30501 (7464-80388)	559.62 (136.95-1474.93)	-1.6 (-1.68--1.53)
**Regional levels**					
High-middle SDI	135019 (31195-377054)	15.54 (3.58-42.17)	284636 (67457-795109)	14.41 (3.4-40)	-0.28 (-0.3--0.26)
High SDI	204091 (47630-591998)	18.44 (4.31-53.5)	296943 (70641-820141)	12.55 (3-35.53)	-1.36 (-1.42--1.29)
Low-middle SDI	46759 (10795-138328)	10.58 (2.4-30.2)	104821 (24130-306814)	9.01 (2.05-25.92)	-0.54 (-0.56--0.52)
Low SDI	10044 (2294-29752)	6.57 (1.52-19.22)	20862 (4884-60484)	5.75 (1.35-16.39)	-0.45 (-0.5--0.41)
Middle SDI	131615 (29878-371448)	16.59 (3.76-46.41)	309363 (74088-891174)	12.91 (3.05-36.65)	-0.95 (-1.01--0.88)
World Bank High Income	234869 (54832-678864)	18.16 (4.24-52.49)	324116 (77413-894714)	12.05 (2.92-33.74)	-0.55 (-1.28-0.18)
World Bank Low Income	6714 (1517-19986)	6.68 (1.57-18.88)	12827 (2926-37069)	5.31 (1.24-15.16)	-0.62 (-2.18-0.98)
World Bank Lower Middle Income	76251 (17092-223365)	10.01 (2.27-28.63)	173635 (40389-515302)	8.71 (1.96-25.46)	0.17 (-0.86-1.22)
World Bank Upper Middle Income	209692 (47559-589066)	17.27 (3.91-47.73)	506044 (121071-1455206)	15.19 (3.59-43.1)	0.79 (0.37-1.22)
Advanced Health System	260145 (60698-753119)	16.27 (3.79-46.9)	363437 (87313-999541)	11.41 (2.75-32)	-0.37 (-1.01-0.28)
Africa	16007 (3724-46934)	8.01 (1.88-22.51)	30456 (6726-90387)	6.48 (1.45-18.93)	-0.64 (-2.15-0.91)

African Region	9985 (2307-29034)	6.38 (1.49-18.04)	17893 (4102-53405)	4.79 (1.09-14.12)	-0.94 (-2.5-0.65)
America	111187 (25858-323059)	18.85 (4.38-54.77)	166580 (40554-467009)	11.97 (2.92-33.61)	-0.81 (-1.03--0.59)
Andean Latin America	834 (188-2439)	4.71 (1.05-13.54)	2122 (456-6259)	3.77 (0.81-11.08)	0.26 (-0.41-0.94)
Asia	257436 (58823-724377)	16.77 (3.86-46.75)	635231 (151073-1802835)	13.78 (3.23-38.36)	0.48 (-0.14-1.1)
Australasia	2823 (658-8129)	12.12 (2.81-34.81)	4712 (1098-13365)	8.02 (1.88-22.81)	-0.48 (-1.05-0.09)
Basic Health System	217064 (49510-609813)	18.81 (4.27-52.05)	537725 (128544-1545622)	15.81 (3.71-44.28)	0.64 (0.06-1.22)
Caribbean	2163 (493-6215)	9.18 (2.09-26.05)	3849 (835-11488)	7.01 (1.53-20.94)	-0.14 (-0.44-0.15)
Central Africa	744 (163-2208)	3.61 (0.8-10.68)	1400 (305-4151)	2.76 (0.66-8.17)	-0.87 (-2.5-0.78)
Central Asia	2751 (618-8120)	6.49 (1.51-18.92)	5150 (1126-15186)	7.4 (1.7-21.91)	0.99 (0.38-1.6)
Central Europe	16980 (3844-50139)	12.16 (2.86-35.83)	21724 (5046-62802)	9.37 (2.15-27.42)	0.14 (-0.44-0.72)
Central Latin America	5499 (1247-16089)	8.03 (1.85-23.35)	10206 (2345-29859)	4.26 (0.98-12.41)	-0.87 (-1.61--0.13)
Central Sub-Saharan Africa	648 (140-1994)	4.11 (0.91-12.34)	1506 (328-4485)	3.62 (0.81-10.77)	-0.25 (-1.93-1.46)
Commonwealth High Income	28691 (6706-81158)	18.7 (4.35-53.11)	33128 (7821-94126)	10.9 (2.6-31.03)	-1.22 (-1.93--0.5)
Commonwealth Low Income	6474 (1484-18685)	10.01 (2.26-28.91)	16741 (4007-48903)	9.28 (2.23-26.64)	0.44 (-1.03-1.93)
Commonwealth Middle Income	37073 (8354-1096	8.6 (1.91-24.6	83823 (19084-246	6.83 (1.54-19.9	-0.01 (-1.1-1.

	13)	6)	549)	8)	09)
East Asia	152014 (34295-423475)	24.08 (5.43-66.46)	399912 (94117-1152847)	19.86 (4.66-56.37)	0.94 (0.43-1.46)
Eastern Africa	3391 (803-9885)	7.39 (1.79-20.77)	6421 (1489-18489)	5.63 (1.33-16.18)	-0.81 (-2.58-0.99)
Eastern Europe	18031 (3975-53776)	6.87 (1.53-20.11)	27774 (6498-81010)	7.6 (1.77-22.23)	1.09 (0.57-1.6)
Eastern Mediterranean Region	18225 (4208-52357)	13.59 (3.13-38.12)	36319 (8171-107109)	10.92 (2.44-31.35)	-0.42 (-1.7-0.87)
Eastern Sub-Saharan Africa	3240 (762-9682)	6.67 (1.58-19.26)	5991 (1399-17364)	4.98 (1.19-14.12)	-0.89 (-2.7-0.95)
Europe	142646 (33159-408313)	14.14 (3.3-40.26)	183841 (44608-508958)	10.43 (2.52-29.58)	-0.2 (-0.86-0.46)
European Region	144913 (33695-415182)	13.92 (3.24-39.65)	187879 (45557-521061)	10.3 (2.49-29.18)	-0.23 (-0.83-0.37)
High-income Asia Pacific	31123 (7335-89186)	17.1 (4.02-48.44)	60084 (14427-165788)	10.55 (2.55-29.63)	0.02 (-0.92-0.96)
High-income North America	81803 (19184-234382)	22.36 (5.24-64.83)	109936 (26667-304095)	15.41 (3.76-42.55)	-0.71 (-1.3--0.12)
Limited Health System	48486 (11007-143170)	8.85 (1.99-25.29)	112421 (25888-327990)	7.29 (1.67-21.08)	0.06 (-1.13-1.27)
Minimal Health System	1833 (421-5376)	4.27 (1.01-12.27)	3042 (701-9065)	3.38 (0.79-9.76)	-0.95 (-2.61-0.74)
North Africa and Middle East	19995 (4589-57553)	15.95 (3.67-44.38)	43206 (9667-125032)	12.11 (2.81-35.02)	-0.25 (-1.33-0.85)
North America	81799 (19183-234372)	22.35 (5.24-64.82)	109933 (26666-304093)	15.4 (3.76-42.54)	-0.71 (-1.3--0.12)
Northern Africa	6914 (1609-19799)	15.69 (3.59-44.73)	15421 (3297-46101)	13.81 (2.94-39.96)	0.19 (-0.96-1.34)
Oceania	195	8.52	428	6.96	-0.38

	(40-574)	(1.85-24.29)	(92-1270)	(1.55-20.9)	(-1.69-0.94)
Region of the Americas	111187 (25858-323059)	18.85 (4.38-54.77)	166580 (40554-467009)	11.97 (2.92-33.61)	-0.81 (-1.03--0.59)
South-East Asia Region	47138 (10407-138932)	9.74 (2.17-28.14)	122250 (28516-360953)	8.35 (1.92-24.38)	0.51 (-0.49-1.52)
South Asia	36576 (8236-108597)	9.31 (2.07-26.84)	91684 (21054-268268)	7.63 (1.74-21.99)	0.29 (-0.47-1.05)
Southeast Asia	25837 (5861-75102)	13.4 (3.04-37.88)	59494 (13753-174778)	10.91 (2.57-31.32)	0.14 (-0.72-1.01)
Southern Africa	3464 (809-9510)	10.81 (2.55-30)	4403 (977-13219)	5.96 (1.34-17.69)	-1.79 (-3.12--0.44)
Southern Latin America	3428 (805-9920)	7.69 (1.78-22.28)	5878 (1453-17266)	6.59 (1.63-19.35)	0.15 (-0.05-0.35)
Southern Sub-Saharan Africa	2647 (616-7247)	12.28 (2.9-34.21)	2735 (596-8148)	5.74 (1.29-16.99)	-2.01 (-3--1.01)
Tropical Latin America	17785 (4187-52047)	24 (5.5-69.16)	35229 (8392-98540)	14.11 (3.37-39.58)	-0.58 (-1.15-0)
Western Africa	1494 (334-4317)	2.54 (0.56-7.3)	2812 (655-8578)	2.08 (0.49-6.14)	-0.78 (-2.3-0.76)
Western Europe	101941 (23580-288737)	17.06 (3.97-48.59)	124158 (29809-343766)	11.35 (2.79-31.68)	-0.61 (-1.53-0.32)
Western Pacific Region	194349 (44721-542453)	21.58 (4.95-59.48)	481349 (114987-1368046)	17.25 (4.1-48.53)	0.71 (0.29-1.12)
Western Sub-Saharan Africa	1708 (382-4969)	2.61 (0.58-7.43)	3202 (730-9730)	2.12 (0.49-6.25)	-0.83 (-2.35-0.72)
**Country Levels**					
Afghanistan	414 (86-1170)	7.98 (1.71-22.32)	482 (96-1398)	7.58 (1.53-21.7)	-1.36 (-2.96-0.26)
Albania	307 (69-900)	18.18 (4.11-52.7	841 (190-2450)	19.74 (4.39-57.7	2.43 (1.91-2.

		2)		6)	96)
Algeria	1764 (401-5267)	24.38 (5.38-71.98)	4490 (925-13027)	18.09 (3.81-51.37)	0.19 (-1.04-1.43)
American Samoa	2 (0-5)	9.33 (2.01-24.97)	3 (1-10)	8.12 (1.76-23.53)	0.9 (-0.06-1.87)
Andorra	8 (2-23)	15.42 (3.49-43.95)	19 (4-51)	11.49 (2.69-31.24)	0.14 (-0.41-0.7)
Angola	182 (40-551)	6.95 (1.57-20.3)	561 (117-1701)	6.68 (1.44-18.9)	0.03 (-1.88-1.97)
Antigua and Barbuda	3 (1-7)	4.66 (1.08-13.19)	4 (1-12)	4.27 (0.95-12.34)	-0.03 (-0.34-0.28)
Argentina	2606 (597-7568)	8.34 (1.88-24.35)	4262 (1035-12198)	7.48 (1.83-21.41)	0.19 (0-0.37)
Armenia	269 (63-755)	11.62 (2.7-32.82)	623 (139-1778)	14.1 (3.16-40.23)	2.38 (2.01-2.75)
Australia	2217 (516-6303)	11.42 (2.63-32.63)	3650 (841-10400)	7.38 (1.74-21.09)	-0.54 (-1.13-0.04)
Austria	1206 (281-3479)	9.87 (2.28-28.83)	2376 (555-6592)	11.74 (2.81-33.03)	1.34 (0.46-2.24)
Azerbaijan	360 (78-1040)	8.04 (1.78-23.38)	913 (205-2679)	11.16 (2.62-33.18)	1.77 (1.17-2.37)
Bahamas	6 (1-18)	4.47 (1.01-13.16)	16 (3-46)	4.33 (0.88-12.47)	0.94 (0.35-1.53)
Bahrain	16 (3-49)	15.48 (3.37-46.28)	66 (14-197)	11.94 (2.73-33.98)	-0.37 (-2.34-1.63)
Bangladesh	4249 (950-12375)	11.53 (2.48-33.91)	12443 (2996-36290)	10.73 (2.58-30.74)	1.13 (-0.13-2.4)
Barbados	14 (3-41)	4.36 (0.98-12.99)	19 (4-57)	3.69 (0.81-10.69)	-0.37 (-0.87-0.14)
Belarus	1155	9.28	1480	8.86	0.64

	(255-3237)	(2.06-25.88)	(339-4357)	(2.04-26.19)	(0.11-1.19)
Belgium	3171 (735-9281)	19.86 (4.57-58.1)	3734 (886-10479)	13.6 (3.21-38.9)	-0.55 (-1.47-0.38)
Belize	6 (1-17)	6.53 (1.59-18.52)	15 (3-43)	5.54 (1.18-16)	0.01 (-0.94-0.98)
Benin	49 (11-140)	2.76 (0.59-7.92)	72 (16-217)	1.64 (0.35-4.86)	-2.08 (-3.54--0.6)
Bermuda	3 (1-9)	5.62 (1.28-16.57)	9 (2-25)	6.03 (1.29-16.98)	1.69 (1.09-2.3)
Bhutan	9 (2-27)	5.83 (1.17-17.12)	33 (7-89)	6.18 (1.37-16.67)	1.87 (0.67-3.09)
Bolivia (Plurinational State of)	126 (28-370)	4.75 (1.05-13.91)	308 (62-930)	3.87 (0.8-11.35)	0.47 (-0.44-1.38)
Bosnia and Herzegovina	476 (111-1327)	14.22 (3.42-39)	920 (203-2641)	14.41 (3.19-41.02)	1.64 (1.06-2.22)
Botswana	47 (10-137)	11.66 (2.47-33.34)	94 (20-274)	8.37 (1.86-23.71)	-0.39 (-1.74-0.99)
Brazil	17277 (4066-50598)	23.87 (5.47-68.78)	34297 (8186-95843)	14.04 (3.36-39.34)	-0.57 (-1.13-0)
Brunei Darussalam	12 (3-35)	14.7 (3.26-45.17)	21 (4-64)	8.41 (1.76-25.16)	-0.7 (-2.08-0.71)
Bulgaria	1192 (256-3472)	10.18 (2.18-29.32)	1021 (214-3031)	7.13 (1.51-21.12)	-0.55 (-1.32-0.22)
Burkina Faso	72 (16-209)	2.08 (0.47-6.24)	123 (26-368)	1.61 (0.35-4.76)	-0.94 (-2.38-0.52)
Burundi	84 (17-248)	4.82 (0.99-14.08)	105 (23-308)	2.83 (0.65-8.02)	-1.8 (-3.49--0.07)
Cabo Verde	6 (1-19)	2.69 (0.59-8.11)	7 (1-20)	1.57 (0.32-4.6)	-1.73 (-2.32--1.12)

Cambodia	530 (125-1569)	16.9 (3.88-49.4)	1704 (381-4937)	18.5 (4.18-53.18)	1.22 (-0.11-2.57)
Cameroon	114 (23-338)	3.34 (0.69-9.55)	221 (44-653)	2.36 (0.47-6.9)	-1.26 (-2.88-0.38)
Canada	5903 (1334-16795)	18.44 (4.17-53.11)	9210 (2164-25322)	11.51 (2.76-31.92)	-0.64 (-1.3-0.02)
Central African Republic	37 (8-111)	4.83 (1.04-14.55)	49 (10-145)	3.33 (0.7-9.82)	-1.4 (-3.24-0.47)
Chad	77 (17-227)	3.34 (0.73-10.27)	128 (27-383)	3.02 (0.64-8.96)	-1.24 (-2.85-0.4)
Chile	532 (122-1507)	5.64 (1.3-15.98)	1159 (269-3486)	4.43 (1.03-13.3)	0.1 (-0.18-0.39)
China	149279 (33699-415217)	24.71 (5.55-68.31)	393521 (92582-1133291)	20.31 (4.76-57.59)	0.95 (0.42-1.47)
Colombia	920 (206-2714)	6.05 (1.37-17.8)	2204 (505-6156)	3.87 (0.88-10.84)	-0.03 (-0.61-0.56)
Comoros	10 (2-29)	7.27 (1.51-21.42)	23 (5-68)	6.35 (1.34-18.49)	0.33 (-1.03-1.71)
Congo	42 (10-128)	5.47 (1.26-16.1)	123 (27-360)	6.6 (1.52-18.8)	0.95 (-0.63-2.57)
Cook Islands	1 (0-3)	9.99 (2.17-30.13)	2 (0-6)	7.51 (1.71-22.16)	0.57 (0.14-1.01)
Costa Rica	148 (36-429)	9.25 (2.24-26.61)	351 (80-996)	6.26 (1.42-17.79)	0.12 (-0.41-0.65)
Cmte d'Ivoire	104 (22-319)	3.79 (0.79-11.04)	243 (49-729)	2.89 (0.61-8.79)	-0.88 (-2.7-0.98)
Croatia	916 (206-2656)	17.09 (3.86-48.87)	1394 (307-3952)	14.34 (3.1-40.86)	1.09 (0.38-1.81)
Cuba	1111 (248-3049)	11.54 (2.6-31.97)	1670 (354-5032)	8.1 (1.72-24.5	-0.19 (-0.63-0

				8)	.26)
Cyprus	117 (27-342)	16.8 (3.76-47.95)	273 (65-737)	13.09 (3.04-35.71)	-0.07 (-0.34-0.2)
Czechia	1763 (410-5096)	13.06 (3-37.68)	2540 (561-7309)	11.22 (2.49-31.84)	0.36 (-0.33-1.05)
Democratic People's Republic of Korea	1261 (265-3747)	9.8 (2.09-27.71)	2564 (553-7764)	8.34 (1.82-25.88)	0.32 (-0.03-0.68)
Democratic Republic of the Congo	362 (79-1137)	3.25 (0.69-10.38)	723 (152-2157)	2.49 (0.56-7.15)	-0.68 (-2.3-0.96)
Denmark	2711 (637-7460)	30.99 (7.27-84.59)	2583 (601-7308)	19.05 (4.41-53.86)	-1.27 (-2.15--0.39)
Djibouti	8 (2-26)	10.52 (2.26-30.58)	39 (8-115)	9.71 (2-27.38)	0.7 (-1.24-2.67)
Dominica	2 (1-7)	3.93 (0.86-11.11)	3 (1-9)	3.44 (0.73-10.57)	0.24 (0.01-0.46)
Dominican Republic	401 (88-1185)	13.9 (3.13-42.34)	1013 (224-2930)	10.67 (2.37-30.95)	0.32 (-0.4-1.05)
Ecuador	293 (67-858)	6.41 (1.47-18.49)	507 (109-1545)	3.26 (0.7-9.83)	-1.14 (-1.81--0.46)
Egypt	2934 (675-8581)	18.19 (4.09-53.63)	6744 (1474-20003)	18.46 (3.99-52.22)	0.36 (-1.2-1.95)
El Salvador	99 (23-291)	3.53 (0.82-10.28)	229 (51-694)	3.57 (0.8-10.9)	1.21 (0.71-1.71)
Equatorial Guinea	7 (1-20)	4.69 (0.99-14.34)	15 (3-44)	4.12 (0.84-12.25)	-0.8 (-2.58-1.02)
Eritrea	17 (3-49)	2.1 (0.45-6.33)	39 (8-117)	1.92 (0.39-5.88)	-0.01 (-1.81-1.82)
Estonia	151 (33-438)	7.39 (1.59-21.38)	248 (56-686)	8.54 (1.87-24.16)	1.46 (0.69-2.24)
Eswatini	14 (3-44)	7.65	18 (4-53)	5.23	-0.81

		(1.58-23.13)		(1.04-15.17)	(-2.67-1.08)
Ethiopia	392 (78-1147)	2.41 (0.48-7.09)	787 (173-2439)	2.25 (0.51-6.97)	0.25 (-1.39-1.91)
Fiji	24 (5-71)	8.31 (1.83-24.38)	41 (9-118)	5.93 (1.25-17.58)	0.03 (-0.88-0.95)
Finland	685 (164-2033)	9.52 (2.28-28.53)	1212 (280-3311)	8.49 (1.96-23.1)	0.84 (-0.07-1.75)
France	11057 (2537-32031)	12.42 (2.82-36.12)	14701 (3448-40493)	9.01 (2.07-24.59)	-0.36 (-1.25-0.55)
Gabon	18 (4-54)	3.76 (0.87-11.04)	34 (7-102)	4 (0.83-11.63)	0.08 (-1.01-1.18)
Gambia	13 (3-38)	4.64 (1.02-13.18)	23 (5-70)	2.85 (0.65-8.33)	-1.54 (-3.03--0.04)
Georgia	457 (104-1337)	7.6 (1.76-22.43)	700 (153-2103)	10.72 (2.34-32.3)	2.44 (1.84-3.05)
Germany	21740 (5299-60610)	16.46 (4.05-46.07)	28730 (7142-80073)	12.47 (3-35.63)	-0.11 (-1.12-0.9)
Ghana	115 (26-340)	2.97 (0.64-8.71)	349 (77-1073)	3.13 (0.7-9.42)	1.01 (-0.65-2.7)
Greece	3250 (745-9191)	22.11 (5.02-61.76)	4751 (1084-13327)	15.91 (3.74-44.76)	0.18 (-0.75-1.12)
Greenland	5 (1-15)	20.68 (4.85-59.52)	10 (2-30)	16.71 (3.58-47.82)	0.71 (0.04-1.38)
Grenada	3 (1-10)	3.98 (0.9-12.04)	3 (1-9)	2.9 (0.66-8.5)	-0.92 (-1.25--0.59)
Guam	4 (1-13)	6.71 (1.52-19.44)	14 (3-42)	6.25 (1.38-18.85)	1.62 (1.06-2.18)
Guatemala	146 (32-440)	5.8 (1.28-17.75)	436 (95-1266)	4.46 (0.97-13.17)	0.38 (-0.88-1.66)

Guinea	98 (21-288)	3.59 (0.73-10.05)	147 (32-421)	3.19 (0.7-9.15)	-0.84 (-2-0.33)
Guinea-Bissau	5 (1-15)	1.64 (0.35-4.95)	10 (2-31)	1.78 (0.38-5.51)	0.44 (-1.29-2.21)
Guyana	13 (3-38)	4.25 (0.92-12.39)	20 (4-61)	3.64 (0.78-11.03)	0.59 (-0.36-1.55)
Haiti	83 (18-248)	3.24 (0.72-9.64)	139 (30-411)	2.57 (0.59-7.48)	-0.64 (-1.87-0.6)
Honduras	149 (35-450)	8.54 (1.99-25.75)	453 (96-1347)	8.46 (1.82-24.71)	0.6 (-0.54-1.74)
Hungary	1589 (378-4706)	11 (2.67-32.21)	1598 (361-4391)	7.89 (1.76-22.32)	-0.39 (-1.11-0.34)
Iceland	64 (15-180)	20.93 (4.77-59.01)	80 (19-214)	12.25 (2.86-33.04)	-1.22 (-1.74--0.7)
India	25777 (5732-77024)	8.42 (1.82-24.97)	68147 (15520-196628)	7.2 (1.62-21.03)	0.53 (-0.52-1.59)
Indonesia	6887 (1497-20942)	9.8 (2.13-29)	20947 (4801-64809)	12.26 (2.88-36.29)	1.36 (0.31-2.42)
Iran (Islamic Republic of)	1987 (449-5664)	9.83 (2.28-27.71)	6359 (1410-18032)	9.41 (2.08-27.09)	1.39 (0.43-2.36)
Iraq	1752 (388-4821)	25.2 (5.58-69.36)	2849 (610-8713)	17.2 (3.71-50.76)	-1.17 (-2.56-0.23)
Ireland	1047 (233-2922)	27.37 (5.96-76.42)	1106 (263-3099)	13.02 (3.1-36.58)	-2.01 (-2.4--1.63)
Israel	679 (151-1979)	14.91 (3.31-44.09)	1304 (289-3531)	9.69 (2.15-26.23)	-0.76 (-0.99--0.53)
Italy	16930 (3886-46438)	19.08 (4.39-52.22)	19787 (4600-52641)	11.52 (2.74-31.29)	-0.67 (-1.72-0.4)
Jamaica	128 (29-379)	6.99 (1.61-20.8	178 (38-508)	5.4 (1.15-15.8)	-0.39 (-0.59--

		6)			0.19)
Japan	26375 (6170-75419)	16.58 (3.9-47.24)	46492 (11027-126052)	10.26 (2.41-28.32)	-0.05 (-1.2-1.11)
Jordan	225 (53-635)	25.49 (6-72.57)	928 (222-2666)	18.25 (4.35-49.55)	-0.41 (-2.09-1.31)
Kazakhstan	628 (135-1814)	5.4 (1.19-15.45)	809 (166-2420)	4.86 (1.04-14.6)	-0.04 (-0.38-0.31)
Kenya	434 (95-1279)	7.2 (1.62-21.25)	734 (155-2040)	4.34 (0.98-11.93)	-1.68 (-3.36-0.03)
Kiribati	5 (1-14)	16.49 (3.86-47.48)	10 (2-30)	19.24 (4.22-55.17)	0.56 (-0.75-1.88)
Kuwait	67 (15-187)	16.72 (3.8-46.12)	307 (73-880)	14.62 (3.52-41.8)	0.24 (-1.19-1.68)
Kyrgyzstan	209 (49-613)	7.8 (1.84-23.14)	477 (103-1448)	11.98 (2.67-35.22)	2.12 (1.29-2.95)
Lao People's Democratic Republic	180 (39-540)	12.21 (2.65-35.27)	444 (105-1284)	13.16 (3.22-37.04)	0.78 (-0.55-2.13)
Latvia	274 (58-832)	7.71 (1.62-23.3)	274 (62-834)	6.51 (1.46-20.31)	0.44 (-0.33-1.2)
Lebanon	341 (75-1008)	19.4 (4.19-57.27)	1443 (352-4120)	22.26 (5.41-63.5)	1.6 (1.14-2.06)
Lesotho	65 (14-194)	10.17 (2.06-29.72)	82 (18-233)	11.64 (2.5-31.88)	0.44 (-0.78-1.67)
Liberia	23 (5-68)	2.46 (0.53-7.09)	32 (6-98)	1.83 (0.39-5.53)	-1.87 (-3.23--0.49)
Libya	256 (58-696)	15.82 (3.55-42.3)	502 (110-1494)	12.25 (2.71-36.65)	-0.1 (-1.17-0.97)
Lithuania	386 (85-1141)	8.7 (1.91-25.64)	447 (103-1383)	7 (1.61-21.87)	0.39 (-0.33-1.11)
Luxembourg	52 (12-156)	9.64	96 (22-277)	8.47	-0.03

		(2.19-29.05)		(1.89-23.87)	(-0.64-0.58)
Madagascar	182 (42-525)	5.37 (1.25-15.79)	160 (34-478)	2.41 (0.52-6.99)	-3.06 (-4.97--1.1)
Malawi	211 (45-651)	8.34 (1.79-25.58)	400 (85-1204)	7.35 (1.58-21.72)	-0.38 (-2.11-1.39)
Malaysia	1169 (261-3293)	15.03 (3.38-42.84)	2429 (576-7268)	10.7 (2.46-31.23)	-0.56 (-1.53-0.42)
Maldives	11 (2-33)	21.7 (4.89-64.52)	37 (9-103)	13.99 (3.36-39.6)	-0.22 (-1.65-1.24)
Mali	85 (19-242)	3.25 (0.72-8.82)	304 (66-900)	5.23 (1.16-15.41)	1.64 (-0.17-3.48)
Malta	52 (12-149)	13.1 (2.96-36.57)	91 (20-248)	8.58 (1.91-23.68)	-0.36 (-1.05-0.34)
Marshall Islands	1 (0-3)	7.35 (1.56-22.14)	2 (0-5)	7.57 (1.55-23.13)	0.96 (-0.73-2.67)
Mauritania	21 (5-61)	2.53 (0.55-7.06)	40 (8-117)	2.15 (0.46-6.29)	-0.54 (-1.74-0.67)
Mauritius	59 (14-175)	9.97 (2.32-29.36)	163 (38-452)	9.25 (2.16-26.16)	1.4 (0.82-2)
Mexico	3044 (688-8842)	9.28 (2.02-26.89)	4416 (992-12356)	3.83 (0.85-10.73)	-1.72 (-2.53--0.91)
Micronesia (Federated States of)	5 (1-15)	12.03 (2.54-33.68)	7 (1-21)	10.81 (2.27-31.95)	0.37 (-0.74-1.5)
Monaco	12 (3-33)	14.81 (3.44-42.73)	14 (3-41)	12.3 (2.8-35.86)	-0.41 (-1.82-1.01)
Mongolia	72 (16-212)	7.78 (1.73-23.01)	170 (38-505)	9.43 (2.11-27.36)	1.28 (0.1-2.46)
Montenegro	89 (20-248)	14.9 (3.45-42.38)	124 (29-372)	12.78 (3-37.51)	0.22 (-0.2-0.65)

Morocco	1060 (238-3016)	8.73 (1.93-24.62)	1662 (346-4623)	5.33 (1.1-14.88)	-0.79 (-1.52--0.04)
Mozambique	250 (59-746)	6.34 (1.44-18.19)	394 (88-1190)	5.21 (1.14-15.12)	-0.83 (-2.57-0.93)
Myanmar	2985 (635-8592)	18.57 (4.16-52.72)	3826 (833-11527)	9.83 (2.17-30)	-1.36 (-2.28--0.44)
Namibia	58 (14-177)	14.84 (3.44-47.74)	102 (23-293)	11.18 (2.45-30.74)	-0.63 (-2.2-0.97)
Nauru	0 (0-1)	12.8 (2.87-38.04)	0 (0-1)	9.66 (2.09-28.26)	-0.58 (-1.98-0.84)
Nepal	986 (226-2866)	16.06 (3.54-46.8)	2299 (516-6738)	13.21 (2.92-39.05)	0.25 (-0.97-1.49)
Netherlands	4273 (1026-12505)	20.93 (5.02-61.22)	6388 (1424-17569)	16.21 (3.66-44.93)	0.37 (-0.39-1.15)
New Zealand	606 (145-1726)	15.58 (3.71-44.39)	1062 (253-2953)	11.58 (2.77-31.97)	-0.18 (-0.69-0.33)
Nicaragua	78 (18-226)	5.99 (1.42-16.79)	226 (48-642)	5.17 (1.11-14.6)	0.93 (-0.12-2)
Niger	33 (7-100)	1.94 (0.44-5.64)	102 (21-305)	2.14 (0.46-6.55)	0.11 (-2.07-2.34)
Nigeria	698 (147-1951)	2.18 (0.45-6.33)	1046 (235-3229)	1.49 (0.34-4.43)	-1.52 (-3.02-0)
Niue	0 (0-1)	8.22 (1.81-23.43)	0 (0-0)	7.28 (1.56-20.27)	0 (-0.26-0.26)
North Macedonia	257 (56-732)	15.21 (3.35-43.58)	376 (84-1076)	13.29 (2.95-38.13)	0.21 (0.01-0.41)
Northern Mariana Islands	1 (0-3)	9.54 (2.16-29.01)	4 (1-11)	7.82 (1.75-22.77)	1.78 (0.36-3.22)
Norway	1338 (299-3752)	17.94 (4.07-49.4	1042 (243-2914)	9.21 (2.17-25.7	-2.39 (-3.24--

		6)		2)	1.52)
Oman	40 (9-119)	8.03 (1.74-23.07)	80 (17-235)	5.85 (1.37-16.44)	-0.74 (-2.46-1.02)
Pakistan	5556 (1229-16547)	12.38 (2.71-36.09)	7113 (1618-21600)	7.95 (1.83-24.44)	-1.77 (-3.04--0.48)
Palau	1 (0-2)	8.35 (1.8-24.38)	1 (0-4)	7.27 (1.76-21.02)	0.56 (-0.12-1.25)
Palestine	118 (24-334)	16.84 (3.58-48.51)	249 (53-737)	13.14 (2.82-38.4)	-0.89 (-2.46-0.7)
Panama	106 (23-297)	7.99 (1.74-22.29)	232 (49-650)	5.1 (1.08-14.25)	-0.62 (-1.06--0.18)
Papua New Guinea	103 (20-304)	7.14 (1.46-19.97)	257 (55-771)	6.2 (1.37-18.27)	-0.42 (-1.82-0.99)
Paraguay	508 (122-1458)	26.19 (6.31-75.26)	932 (197-2526)	17.55 (3.69-47.08)	-0.7 (-1.41-0.02)
Peru	415 (93-1180)	3.93 (0.88-11.41)	1307 (285-3837)	3.94 (0.86-11.6)	0.92 (0.32-1.53)
Philippines	3615 (787-10567)	18.21 (4.09-52.5)	7290 (1624-21745)	11.15 (2.55-33.15)	-0.71 (-1.87-0.47)
Poland	5923 (1364-17463)	14.07 (3.26-41.31)	7231 (1713-20385)	9.55 (2.31-27.01)	-0.32 (-0.86-0.23)
Portugal	1123 (248-3307)	8.1 (1.8-23.92)	1351 (311-3711)	5.1 (1.14-14.42)	-0.69 (-1.53-0.17)
Puerto Rico	238 (55-696)	6.82 (1.58-19.7)	480 (110-1384)	5.76 (1.26-16.51)	0.86 (0.19-1.54)
Qatar	7 (1-19)	9.88 (2.14-29.85)	47 (9-146)	7.62 (1.75-21.79)	-0.57 (-2.87-1.78)
Republic of Korea	4618 (1080-13158)	24.34 (5.52-68.02)	13215 (3150-38266)	14.18 (3.37-40.74)	0.49 (-0.14-1.13)
Republic of	266	6.63	463	7.48	1.61

Moldova	(60-775)	(1.54-19.24)	(101-1324)	(1.63-21.46)	(1.23-2)
Romania	2277 (495-6771)	9.11 (1.97-26.14)	2495 (567-7228)	6.46 (1.46-18.66)	-0.26 (-0.84-0.32)
Russian Federation	10313 (2250-30758)	6 (1.33-17.78)	19461 (4593-56834)	7.83 (1.87-22.86)	1.62 (1.12-2.12)
Rwanda	258 (61-749)	15.46 (3.5-45.86)	768 (178-2245)	19.39 (4.44-53.71)	1.26 (-0.71-3.27)
Saint Kitts and Nevis	1 (0-4)	3.54 (0.78-10.4)	2 (0-5)	2.9 (0.61-8.7)	-0.69 (-0.96--0.43)
Saint Lucia	4 (1-13)	5.09 (1.15-15.95)	9 (2-28)	3.8 (0.79-11.72)	0.21 (-0.22-0.64)
Saint Vincent and the Grenadines	3 (1-8)	3.81 (0.88-11.3)	5 (1-16)	3.86 (0.85-11.35)	1.15 (0.77-1.54)
Samoa	10 (2-30)	13.92 (3.03-39.82)	16 (3-46)	12.57 (2.8-35.69)	0.01 (-0.89-0.93)
San Marino	5 (1-15)	13.7 (3.18-39.67)	10 (2-24)	10.14 (2.23-26.54)	0.03 (-1.06-1.14)
Sao Tome and Principe	1 (0-3)	1.54 (0.33-4.57)	1 (0-5)	1.51 (0.31-4.68)	-0.68 (-1.8-0.44)
Saudi Arabia	313 (67-938)	6.9 (1.49-19.66)	863 (172-2562)	6.49 (1.42-19.04)	0 (-1.61-1.63)
Senegal	83 (19-249)	2.84 (0.64-8.64)	161 (33-471)	2.28 (0.48-6.47)	-0.42 (-1.63-0.81)
Serbia	1140 (243-3329)	11.02 (2.41-32.16)	1687 (364-4863)	9.87 (2.15-28.64)	0.64 (0.04-1.25)
Seychelles	7 (2-19)	12.28 (2.73-33.9)	10 (2-29)	9.89 (2.2-28.82)	-0.47 (-0.78--0.16)
Sierra Leone	52 (11-155)	2.97 (0.64-8.55)	71 (15-219)	2.22 (0.51-6.57)	-1.37 (-2.64--0.1)

Singapore	118 (25-347)	6.73 (1.51-19.9)	355 (80-939)	4.17 (0.93-10.97)	-0.04 (-0.51-0.43)
Slovakia	556 (127-1582)	9.7 (2.21-27.69)	778 (173-2180)	7.99 (1.79-22.51)	0.4 (-0.08-0.88)
Slovenia	226 (49-674)	9.25 (1.97-27.71)	403 (92-1172)	8.6 (1.94-25.09)	1.09 (0.35-1.83)
Solomon Islands	13 (3-40)	12.6 (2.77-37.86)	32 (7-97)	11.75 (2.43-35.53)	0.36 (-1.07-1.81)
Somalia	78 (16-252)	5.37 (1.14-16.39)	160 (30-458)	4.11 (0.81-11.07)	-0.84 (-3.07-1.43)
South Africa	2093 (484-5721)	12.27 (2.87-33.43)	1969 (429-5851)	4.97 (1.1-14.86)	-2.42 (-3.24--1.58)
South Sudan	119 (24-348)	5.88 (1.24-16.36)	133 (27-396)	4.97 (1.03-14.7)	-1.05 (-2.75-0.68)
Spain	8281 (1904-22786)	15.07 (3.46-41.05)	9539 (2267-25782)	8.57 (2.03-23.4)	-1.11 (-1.97--0.24)
Sri Lanka	796 (181-2223)	10.46 (2.37-30.1)	1131 (248-3382)	4.71 (1.06-14.04)	-1.45 (-2.07--0.83)
Sudan	807 (188-2356)	11.74 (2.76-34.45)	1473 (315-4451)	10.28 (2.27-31.37)	-0.54 (-1.96-0.9)
Suriname	20 (4-58)	8.42 (1.8-24.67)	34 (7-102)	5.63 (1.16-17.01)	-0.84 (-1.31--0.37)
Sweden	2575 (571-7360)	15.37 (3.42-43.23)	3600 (849-10064)	14.02 (3.31-39.48)	0.16 (-0.93-1.26)
Switzerland	1896 (443-5479)	16.88 (3.91-48.47)	2940 (730-7848)	13.44 (3.35-36.38)	0.06 (-0.89-1.02)
Syrian Arab Republic	774 (180-2274)	18.84 (4.22-55.55)	1248 (270-3474)	13.39 (2.83-37.7)	-0.08 (-1.62-1.48)
Taiwan (Province of	1474 (343-4275)	12.15 (2.88-33.8	3827 (830-11269)	8.36 (1.81-24.6	0.4 (-0.1-0.

China)		1)		7)	91)
Tajikistan	251 (56-772)	10.52 (2.37-31.77)	282 (55-843)	6.47 (1.32-18.82)	-1.42 (-2.67--0.15)
Thailand	4157 (884-11755)	15.02 (3.28-42.53)	10753 (2435-29844)	9.65 (2.19-26.75)	0.29 (-0.28-0.87)
Timor-Leste	21 (4-61)	11.93 (2.5-34)	71 (16-210)	10.81 (2.48-32.22)	0.92 (-0.61-2.48)
Togo	58 (13-161)	6.52 (1.42-17.92)	121 (25-364)	4.17 (0.97-12.24)	-0.94 (-2.66-0.81)
Tokelau	0 (0-0)	11.27 (2.41-33.16)	0 (0-0)	8.9 (1.84-27.26)	-0.34 (-0.52--0.17)
Tonga	8 (2-21)	17.31 (3.97-46.45)	11 (2-31)	14.05 (2.85-40.86)	-0.1 (-0.83-0.64)
Trinidad and Tobago	48 (10-144)	6.31 (1.42-19.23)	92 (19-288)	4.73 (0.99-14.89)	0.33 (-0.02-0.69)
Tunisia	879 (218-2453)	23.26 (5.65-62.16)	1983 (416-5623)	16.87 (3.57-48.36)	0.14 (-0.5-0.78)
Tmrkiye	5638 (1254-16125)	19.6 (4.37-55.68)	9668 (2190-28340)	11.16 (2.56-32.55)	-0.88 (-1.44--0.32)
Turkmenistan	124 (28-344)	7.89 (1.72-21.98)	184 (40-523)	5.38 (1.14-15.52)	-0.49 (-1.44-0.47)
Tuvalu	1 (0-2)	10.17 (2.18-30.11)	1 (0-3)	9.83 (2.21-28.33)	0.12 (-0.4-0.65)
Uganda	215 (45-591)	5.06 (1.07-13.92)	477 (101-1397)	4.72 (1.01-13.54)	-0.77 (-2.79-1.29)
Ukraine	5486 (1259-16223)	8.17 (1.89-23.77)	5402 (1147-16466)	6.7 (1.43-20.57)	-0.07 (-0.66-0.52)
United Arab Emirates	29 (6-84)	12.03 (2.66-33.22)	233 (45-669)	14.53 (3.13-39.96)	0.41 (-2.61-3.53)
United	19587	20.63	18322	12.18	-1.42

Kingdom	(4607-55731)	(4.83-58.93)	(4314-51449)	(2.85-34.65)	(-2.3--0.54)
United Republic of Tanzania	806 (181-2362)	10.88 (2.66-30.76)	1453 (331-3995)	7.49 (1.76-20.64)	-1.24 (-2.82-0.36)
United States of America	75892 (17798-217611)	22.76 (5.34-66.07)	100714 (24593-278920)	15.89 (3.9-43.99)	-0.71 (-1.29--0.12)
United States Virgin Islands	4 (1-11)	5.28 (1.1-14.63)	9 (2-23)	4.45 (0.89-12.15)	1.43 (0.89-1.97)
Uruguay	289 (69-845)	7.33 (1.74-21.38)	457 (106-1339)	7.68 (1.72-22.91)	0.65 (0.12-1.18)
Uzbekistan	382 (88-1131)	3.55 (0.83-10.39)	992 (219-3008)	4.5 (1.06-13.7)	1.21 (0.32-2.1)
Vanuatu	4 (1-10)	7.54 (1.67-21.67)	8 (2-22)	5.64 (1.3-15.96)	-0.71 (-2.1-0.7)
Venezuela (Bolivarian Republic of)	809 (184-2327)	9.9 (2.22-27.88)	1658 (343-4991)	5.74 (1.2-17.11)	-0.48 (-1.17-0.22)
Viet Nam	5382 (1211-15389)	15.04 (3.39-42.51)	10607 (2287-30227)	11.97 (2.72-33.74)	-0.3 (-0.95-0.36)
Yemen	562 (129-1677)	15.57 (3.64-46.08)	1490 (320-4190)	14.56 (3.24-41.76)	-0.03 (-1.74-1.71)
Zambia	174 (39-487)	9.12 (2-25.07)	313 (66-889)	7.17 (1.6-21.32)	-1.1 (-3.04-0.88)
Zimbabwe	370 (87-1055)	13.38 (3.05-39.53)	470 (100-1353)	10.71 (2.35-31.75)	-0.75 (-2.45-0.97)

Supplementary Table S5 Analysis of global burden indicators for smoking-related ADOD from 1990 to 2021

measure_id	measure_name	location_id	location_name	sex_id	sex_name	age_id	age_name	cause_id	cause_name	rei_id	rei_name	metric_id	metric_name	year	val	upper	lower
1	Deaths	1	Global	3	Both	22	All ages	543	Alzheimer's disease and other dementias	99	Smoking	1	Number	1990	32164.8215771639	89320.2717364865	7445.50857756345
1	Deaths	1	Global	3	Both	22	All ages	543	Alzheimer's disease and other dementias	99	Smoking	3	Rate	1990	0.603057603	1.674664006	0.139595693
1	Deaths	1	Global	3	Both	22	All ages	543	Alzheimer's disease and other dementias	99	Smoking	1	Number	1991	33076.7626299927	92335.3047334272	7679.95154329063
1	Deaths	1	Global	3	Both	22	All ages	543	Alzheimer's disease and other dementias	99	Smoking	3	Rate	1991	0.610665145	1.704699849	0.141787719
1	Deaths	1	Gl	3	B	2	Al	5	Alzhei	9	S	1	N	1	33	94	79

			obal		oth	2	l ages	43	mer's disease and other dementias	9	moking		umber	992	934.627496678	364.9436536631	63.04865352222
1	Deaths	1	Global	3	Both	22	All ages	543	Alzheimer's disease and other dementias	99	Smoking	3	Rate	1992	0.617306633	1.716597763	0.148562246
1	Deaths	1	Global	3	Both	22	All ages	543	Alzheimer's disease and other dementias	99	Smoking	1	Number	1993	34813.6355750697	96542.2153066996	7987.27606461882
1	Deaths	1	Global	3	Both	22	All ages	543	Alzheimer's disease and other dementias	99	Smoking	3	Rate	1993	0.624407389	1.731553502	0.143257494
1	Deaths	1	Global	3	Both	22	All ages	543	Alzheimer's disease and other dementias	99	Smoking	1	Number	1994	35610.4675982803	99874.0259709058	8299.12381204134
1	Deaths	1	Global	3	Both	22	All ages	543	Alzheimer's disease and other dementias	99	Smoking	3	Rate	1994	0.630222551	1.767538246	0.146875212

1	Deaths	1	Global	3	Both	2	All ages	543	Alzheimer's disease and other dementias	99	Smoking	1	Number	1995	36414.5368906737	101349.653971224	8360.55257910997
1	Deaths	1	Global	3	Both	2	All ages	543	Alzheimer's disease and other dementias	99	Smoking	3	Rate	1995	0.636166096	1.770589968	0.146059803
1	Deaths	1	Global	3	Both	2	All ages	543	Alzheimer's disease and other dementias	99	Smoking	1	Number	1996	37119.2788902919	103983.586872991	8637.00043868813
1	Deaths	1	Global	3	Both	2	All ages	543	Alzheimer's disease and other dementias	99	Smoking	3	Rate	1996	0.640164792	1.793316931	0.148955038
1	Deaths	1	Global	3	Both	2	All ages	543	Alzheimer's disease and other dementias	99	Smoking	1	Number	1997	37686.5181874213	105844.384908588	8857.37764275985
1	Deaths	1	Global	3	Both	2	All ages	543	Alzheimer's disease and other dement	99	Smokin	3	Rate	1997	0.641682754	1.802196639	0.150813255

									ias		g						
1	Deaths	1	Global	3	Both	2	All ages	543	Alzheimer’s disease and other dementias	99	Smoking	1	Number	1998	38236.7474830255	108139.376992395	9017.85938933684
1	Deaths	1	Global	3	Both	2	All ages	543	Alzheimer’s disease and other dementias	99	Smoking	3	Rate	1998	0.642863231	1.818115657	0.151614627
1	Deaths	1	Global	3	Both	2	All ages	543	Alzheimer’s disease and other dementias	99	Smoking	1	Number	1999	38800.1803116645	109494.331566109	9062.26093
1	Deaths	1	Global	3	Both	2	All ages	543	Alzheimer’s disease and other dementias	99	Smoking	3	Rate	1999	0.644212651	1.817971799	0.150463815
1	Deaths	1	Global	3	Both	2	All ages	543	Alzheimer’s disease and other dementias	99	Smoking	1	Number	2000	39493.1814939931	111418.63536259	9272.73660659461
1	Deaths	1	Global	3	Both	2	All ages	543	Alzheimer’s disease and other	99	Smoki	3	Rate	2000	0.64755892	1.82690097	0.15204253

									dementias		ng				4		3
1	Deaths	1	Global	3	Both	22	All ages	543	Alzheimer’s disease and other dementias	99	Smoking	1	Number	2001	40210.6578139352	112865.260094444	9407.59836191312
1	Deaths	1	Global	3	Both	22	All ages	543	Alzheimer’s disease and other dementias	99	Smoking	3	Rate	2001	0.651057915	1.827421507	0.152320099
1	Deaths	1	Global	3	Both	22	All ages	543	Alzheimer’s disease and other dementias	99	Smoking	1	Number	2002	40994.4139186431	114882.146565572	9640.955178
1	Deaths	1	Global	3	Both	22	All ages	543	Alzheimer’s disease and other dementias	99	Smoking	3	Rate	2002	0.655346605	1.836533751	0.154122639
1	Deaths	1	Global	3	Both	22	All ages	543	Alzheimer’s disease and other dementias	99	Smoking	1	Number	2003	41727.317784668	116936.619805441	9826.96728
1	Deaths	1	Global	3	Both	22	All ages	543	Alzheimer’s disease and	99	Smok	3	Rate	2003	0.65856 2	1.845555	0.155094

									other dementias		ing				698	859	418
1	Deaths	1	Global	3	Both	22	All ages	543	Alzheimer's disease and other dementias	99	Smoking	1	Number	2004	424431.6313076565	118749.846985734	9871.61574292786
1	Deaths	1	Global	3	Both	22	All ages	543	Alzheimer's disease and other dementias	99	Smoking	3	Rate	2004	0.661096498	1.850155309	0.153802491
1	Deaths	1	Global	3	Both	22	All ages	543	Alzheimer's disease and other dementias	99	Smoking	1	Number	2005	43357.4716262231	122075.93167303	10045.6240933224
1	Deaths	1	Global	3	Both	22	All ages	543	Alzheimer's disease and other dementias	99	Smoking	3	Rate	2005	0.666797172	1.877413118	0.154492259
1	Deaths	1	Global	3	Both	22	All ages	543	Alzheimer's disease and other dementias	99	Smoking	1	Number	2006	44304.4304245358	124578.624908599	10423.2961952058
1	Deaths	1	Global	3	Bot	22	All ag	543	Alzheimer's diseas	99	Smo	3	Rat	20	0.6724	1.8907	0.1581

					h		es		e and other dementias		king		e	6	28521	86536	99114
1	Deaths	1	Global	3	Both	22	All ages	543	Alzheimer's disease and other dementias	99	Smoking	1	Number	2007	45468.1490968297	127146.89672465	10538.8526105519
1	Deaths	1	Global	3	Both	22	All ages	543	Alzheimer's disease and other dementias	99	Smoking	3	Rate	2007	0.680927469	1.90414205	0.157829038
1	Deaths	1	Global	3	Both	22	All ages	543	Alzheimer's disease and other dementias	99	Smoking	1	Number	2008	46706.1287230072	130947.069443532	10878.8661145605
1	Deaths	1	Global	3	Both	22	All ages	543	Alzheimer's disease and other dementias	99	Smoking	3	Rate	2008	0.690047332	1.934642806	0.160726927
1	Deaths	1	Global	3	Both	22	All ages	543	Alzheimer's disease and other dementias	99	Smoking	1	Number	2009	48004.9960524251	134384.568641223	11079.9221241796
1	Deaths	1	Glob	3	Bo	22	Al l	54	Alzheimer's	99	Sm	3	Ra	20	0.69	1.95	0.16

			al		th		ages	3	disease and other dementias		oking		te	09	9672966	8655504	1489899
1	Deaths	1	Global	3	Both	22	All ages	543	Alzheimer's disease and other dementias	99	Smoking	1	Number	2010	49415.5475916472	136773.542007644	11620.8585404757
1	Deaths	1	Global	3	Both	22	All ages	543	Alzheimer's disease and other dementias	99	Smoking	3	Rate	2010	0.710937741	1.967750591	0.167188412
1	Deaths	1	Global	3	Both	22	All ages	543	Alzheimer's disease and other dementias	99	Smoking	1	Number	2011	50816.5022028235	141152.044669737	11911.4288634391
1	Deaths	1	Global	3	Both	22	All ages	543	Alzheimer's disease and other dementias	99	Smoking	3	Rate	2011	0.722046402	2.005614743	0.169248256
1	Deaths	1	Global	3	Both	22	All ages	543	Alzheimer's disease and other dementias	99	Smoking	1	Number	2012	52273.7970378798	146183.097898319	12101.5661404152
1	Deaths	1	Gl	3	B	2	Al	5	Alzhei	9	S	3	R	2	0.	2.	0.

			obal		oth	2	l ages	43	mer's disease and other dementias	9	moking		ate	012	733530234	0513092222	1698148805
1	Deaths	1	Global	3	Both	22	All ages	543	Alzheimer's disease and other dementias	99	Smoking	1	Number	2013	53782.2709608514	152445.290621435	12535.6909093988 35
1	Deaths	1	Global	3	Both	22	All ages	543	Alzheimer's disease and other dementias	99	Smoking	3	Rate	2013	0.745297161	2.112537091	0.173715514
1	Deaths	1	Global	3	Both	22	All ages	543	Alzheimer's disease and other dementias	99	Smoking	1	Number	2014	55312.4020232481	156771.105603881	13015.0819187131
1	Deaths	1	Global	3	Both	22	All ages	543	Alzheimer's disease and other dementias	99	Smoking	3	Rate	2014	0.757069021	2.14574929	0.17813935
1	Deaths	1	Global	3	Both	22	All ages	543	Alzheimer's disease and other dementias	99	Smoking	1	Number	2015	56897.8837853151	161821.48558782	13362.0381968368

1	Deaths	1	Global	3	Both	2 2	All ages	543	Alzheimer’s disease and other dementias	99	Smoking	3	Rate	2015	0.769263405	2.187837907	0.1806557
1	Deaths	1	Global	3	Both	2 2	All ages	543	Alzheimer’s disease and other dementias	99	Smoking	1	Number	2016	58512.0664088988	165139.635349184	13656.9284176308
1	Deaths	1	Global	3	Both	2 2	All ages	543	Alzheimer’s disease and other dementias	99	Smoking	3	Rate	2016	0.781556013	2.205799297	0.182418007
1	Deaths	1	Global	3	Both	2 2	All ages	543	Alzheimer’s disease and other dementias	99	Smoking	1	Number	2017	60189.9634840381	171965.105422098	14255.7462388559
1	Deaths	1	Global	3	Both	2 2	All ages	543	Alzheimer’s disease and other dementias	99	Smoking	3	Rate	2017	0.794528792	2.270000173	0.181808889
1	Deaths	1	Global	3	Both	2 2	All ages	543	Alzheimer’s disease and other dementias	99	Smoking	1	Number	2018	61766.33899858	170823.2193983	14380.74522082

															98	5	1
1	Deaths	1	Global	3	Both	22	All ages	543	Alzheimer's disease and other dementias	99	Smoking	3	Rate	2018	0.806135743	2.229478145	0.18768852
1	Deaths	1	Global	3	Both	22	All ages	543	Alzheimer's disease and other dementias	99	Smoking	1	Number	2019	63530.0557327683	175645.800949771	14646.5053591188
1	Deaths	1	Global	3	Both	22	All ages	543	Alzheimer's disease and other dementias	99	Smoking	3	Rate	2019	0.820237076	2.26776439	0.189101152
1	Deaths	1	Global	3	Both	22	All ages	543	Alzheimer's disease and other dementias	99	Smoking	1	Number	2020	64886.3538169433	182793.126819357	15050.2144057839
1	Deaths	1	Global	3	Both	22	All ages	543	Alzheimer's disease and other dementias	99	Smoking	3	Rate	2020	0.829498044	2.33680169	0.19239983
1	Deaths	1	Global	3	Both	22	All ages	543	Alzheimer's disease and other dement	99	Smokin	1	Number	2021	67175.818042	184664.76413	15694.650005

									ias		g				4356	7772	4487
1	Deaths	1	Global	3	Both	22	All ages	543	Alzheimer's disease and other dementias	99	Smoking	3	Rate	2021	0.851258529	2.340089933	0.198884138
1	Deaths	1	Global	3	Both	27	Age-standardized	543	Alzheimer's disease and other dementias	99	Smoking	3	Rate	1990	1.083533298	3.027386815	0.251079326
1	Deaths	1	Global	3	Both	27	Age-standardized	543	Alzheimer's disease and other dementias	99	Smoking	3	Rate	1991	1.079615584	3.048572244	0.249418154
1	Deaths	1	Global	3	Both	27	Age-standardized	543	Alzheimer's disease and other dementias	99	Smoking	3	Rate	1992	1.074702119	3.038759874	0.249701343
1	Deaths	1	Global	3	Both	27	Age-standardized	543	Alzheimer's disease and other dementias	99	Smoking	3	Rate	1993	1.070739681	2.999883434	0.246243162
1	Deaths	1	Global	3	Bot	27	Age-st	543	Alzheimer's diseas	99	Smo	3	Rat	199	1.0643	3.0064	0.2468

					h		andardized		e and other dementias		king		e	4	86223	65059	80823
1	Deaths	1	Global	3	Both	27	Age-standardized	543	Alzheimer's disease and other dementias	99	Smoking	3	Rate	1995	1.0588533307	3.0063033304	0.2429129114
1	Deaths	1	Global	3	Both	27	Age-standardized	543	Alzheimer's disease and other dementias	99	Smoking	3	Rate	1996	1.0504658177	2.9721421622	0.2427369945
1	Deaths	1	Global	3	Both	27	Age-standardized	543	Alzheimer's disease and other dementias	99	Smoking	3	Rate	1997	1.03810264	2.95347488	0.240991264
1	Deaths	1	Global	3	Both	27	Age-standardized	543	Alzheimer's disease and other dementias	99	Smoking	3	Rate	1998	1.0246770081	2.9254320023	0.23755091
1	Deaths	1	Global	3	Both	27	Age-standardiz	543	Alzheimer's disease and other dementias	99	Smoking	3	Rate	1999	1.0104322733	2.8763502001	0.2332565661

							ed										
1	Deaths	1	Global	3	Both	27	Age-standardized	543	Alzheimer's disease and other dementias	99	Smoking	3	Rate	2000	0.997908495	2.838571377	0.23210568
1	Deaths	1	Global	3	Both	27	Age-standardized	543	Alzheimer's disease and other dementias	99	Smoking	3	Rate	2001	0.985617104	2.798984735	0.227056816
1	Deaths	1	Global	3	Both	27	Age-standardized	543	Alzheimer's disease and other dementias	99	Smoking	3	Rate	2002	0.974899854	2.766813073	0.225659352
1	Deaths	1	Global	3	Both	27	Age-standardized	543	Alzheimer's disease and other dementias	99	Smoking	3	Rate	2003	0.963268671	2.736824028	0.222688402
1	Deaths	1	Global	3	Both	27	Age-standardized	543	Alzheimer's disease and other dementias	99	Smoking	3	Rate	2004	0.949126495	2.680650947	0.219340071
1	Deaths	1	Global	3	Bot	27	Age-st	543	Alzheimer's diseas	99	Smo	3	Rat	20	0.9381	2.6612	0.2148

					h		andardized		e and other dementias		king		e	5	56374	823347	91557
1	Deaths	1	Global	3	Both	27	Age-standardized	543	Alzheimer's disease and other dementias	99	Smoking	3	Rate	2006	0.92595149	2.607577006	0.214681647
1	Deaths	1	Global	3	Both	27	Age-standardized	543	Alzheimer's disease and other dementias	99	Smoking	3	Rate	2007	0.916959279	2.588812109	0.210634366
1	Deaths	1	Global	3	Both	27	Age-standardized	543	Alzheimer's disease and other dementias	99	Smoking	3	Rate	2008	0.909112433	2.570508775	0.210263676
1	Deaths	1	Global	3	Both	27	Age-standardized	543	Alzheimer's disease and other dementias	99	Smoking	3	Rate	2009	0.900854119	2.539275577	0.205584952
1	Deaths	1	Global	3	Both	27	Age-standardiz	543	Alzheimer's disease and other dementias	99	Smoking	3	Rate	2010	0.893892869	2.494978124	0.207348294

							ed										
1	Deaths	1	Global	3	Both	27	Age-standardized	543	Alzheimer's disease and other dementias	99	Smoking	3	Rate	2011	0.886016827	2.47368696	0.204362118
1	Deaths	1	Global	3	Both	27	Age-standardized	543	Alzheimer's disease and other dementias	99	Smoking	3	Rate	2012	0.878472989	2.471860919	0.201274406
1	Deaths	1	Global	3	Both	27	Age-standardized	543	Alzheimer's disease and other dementias	99	Smoking	3	Rate	2013	0.872351859	2.482452615	0.200139883
1	Deaths	1	Global	3	Both	27	Age-standardized	543	Alzheimer's disease and other dementias	99	Smoking	3	Rate	2014	0.866698338	2.455358791	0.202910863
1	Deaths	1	Global	3	Both	27	Age-standardized	543	Alzheimer's disease and other dementias	99	Smoking	3	Rate	2015	0.861642515	2.448921148	0.199919602
1	Deaths	1	Global	3	Bot	27	Age-st	543	Alzheimer's diseas	99	Smo	3	Rat	201	0.8556	2.4147	0.1970

					h		andardized		e and other dementias		king		e	6	29198	16069	71615
1	Deaths	1	Global	3	Both	27	Age-standardized	543	Alzheimer's disease and other dementias	99	Smoking	3	Rate	2017	0.849930806	2.427685803	0.200409185
1	Deaths	1	Global	3	Both	27	Age-standardized	543	Alzheimer's disease and other dementias	99	Smoking	3	Rate	2018	0.842675858	2.354585438	0.195366338
1	Deaths	1	Global	3	Both	27	Age-standardized	543	Alzheimer's disease and other dementias	99	Smoking	3	Rate	2019	0.837225899	2.283632656	0.19261331
1	Deaths	1	Global	3	Both	27	Age-standardized	543	Alzheimer's disease and other dementias	99	Smoking	3	Rate	2020	0.829820529	2.323420956	0.19277174
1	Deaths	1	Global	3	Both	27	Age-standardiz	543	Alzheimer's disease and other dementias	99	Smoking	3	Rate	2021	0.837652906	2.29298703	0.194745737

							ed										
2	DALYs (Disability-Adjusted Life Years)	1	Global	3	Both	22	All ages	543	Alzheimer's disease and other dementias	99	Smoking	1	Number	1990	794915.200783867	1839709.4166161	344377.508851189
2	DALYs (Disability-Adjusted Life Years)	1	Global	3	Both	22	All ages	543	Alzheimer's disease and other dementias	99	Smoking	3	Rate	1990	14.90384936	34.49267543	6.456727095
2	DALYs (Disability-Adjusted Life Years)	1	Global	3	Both	22	All ages	543	Alzheimer's disease and other dementias	99	Smoking	1	Number	1991	813582.541922773	1859921.77514483	350102.098361677
2	DALYs (Disability-Adjusted Life Years)	1	Global	3	Both	22	All ages	543	Alzheimer's disease and other dementias	99	Smoking	3	Rate	1991	15.02040893	34.33798565	6.463605615
2	DALYs (Disability-Adjusted Life Years)	1	Global	3	Both	22	All ages	543	Alzheimer's disease and other dementias	99	Smoking	1	Number	1992	830798.957745391	1894160.61389953	361845.380549833
2	DALYs (Disability-Adjusted	1	Global	3	Both	22	All ages	543	Alzheimer's disease and other	99	Smoki	3	Rate	1992	15.1131085	34.4567775	6.58234877

	Life Years)								dementias		ng					5	
2	DALYs (Disability-Adjusted Life Years)	1	Global	3	Both	22	All ages	543	Alzheimer's disease and other dementias	99	Smoking	1	Number	1993	847999.593359036	1935360.71490659	370492.226391127
2	DALYs (Disability-Adjusted Life Years)	1	Global	3	Both	22	All ages	543	Alzheimer's disease and other dementias	99	Smoking	3	Rate	1993	15.2094776 5	34.7120750 5	6.645042379
2	DALYs (Disability-Adjusted Life Years)	1	Global	3	Both	22	All ages	543	Alzheimer's disease and other dementias	99	Smoking	1	Number	1994	863218.168499111	1973778.9803855	376655.569132528
2	DALYs (Disability-Adjusted Life Years)	1	Global	3	Both	22	All ages	543	Alzheimer's disease and other dementias	99	Smoking	3	Rate	1994	15.27695627	34.93130273	6.665928578
2	DALYs (Disability-Adjusted Life Years)	1	Global	3	Both	22	All ages	543	Alzheimer's disease and other dementias	99	Smoking	1	Number	1995	878049.762292486	2024378.66444585	382636.036634867
2	DALYs (Disability-Adjust	1	Global	3	Both	22	All ages	543	Alzheimer's disease and	99	Smok	3	Rate	1995	15.33962	35.36612	6.684695

	ed Life Years)								other dementias		ing				909	523	027
2	DALYs (Disability-Adjusted Life Years)	1	Global	3	Both	22	All ages	543	Alzheimer's disease and other dementias	99	Smoking	1	Number	1996	891072.368271821	2069134.964	386240.731309941
2	DALYs (Disability-Adjusted Life Years)	1	Global	3	Both	22	All ages	543	Alzheimer's disease and other dementias	99	Smoking	3	Rate	1996	15.3675711	35.6846198	6.661167052
2	DALYs (Disability-Adjusted Life Years)	1	Global	3	Both	22	All ages	543	Alzheimer's disease and other dementias	99	Smoking	1	Number	1997	901290.250451291	2074537.74753608	390595.450046286
2	DALYs (Disability-Adjusted Life Years)	1	Global	3	Both	22	All ages	543	Alzheimer's disease and other dementias	99	Smoking	3	Rate	1997	15.34613538	35.32284646	6.65061078
2	DALYs (Disability-Adjusted Life Years)	1	Global	3	Both	22	All ages	543	Alzheimer's disease and other dementias	99	Smoking	1	Number	1998	910794.434864204	2090695.11869448	395411.852991738
2	DALYs (Disability-	1	Global	3	Bot	22	All ag	543	Alzheimer's diseas	99	Smo	3	Rat	199	15.312	35.150	6.6479

	Adjusted Life Years)				h		es		e and other dementias		king		e	8	91994	2444	43617
2	DALYs (Disability-Adjusted Life Years)	1	Global	3	Both	22	All ages	543	Alzheimer's disease and other dementias	99	Smoking	1	Number	1999	921208.106180023	2122179.62378349	397474.84560184
2	DALYs (Disability-Adjusted Life Years)	1	Global	3	Both	22	All ages	543	Alzheimer's disease and other dementias	99	Smoking	3	Rate	1999	15.29513295	35.2352734	6.599410671
2	DALYs (Disability-Adjusted Life Years)	1	Global	3	Both	22	All ages	543	Alzheimer's disease and other dementias	99	Smoking	1	Number	2000	934624.763269996	2147266.38990309	402712.984171521
2	DALYs (Disability-Adjusted Life Years)	1	Global	3	Both	22	All ages	543	Alzheimer's disease and other dementias	99	Smoking	3	Rate	2000	15.32478729	35.20814125	6.603174946
2	DALYs (Disability-Adjusted Life Years)	1	Global	3	Both	22	All ages	543	Alzheimer's disease and other dementias	99	Smoking	1	Number	2001	949554.956430915	2167433.4724299	410505.486360519
2	DALYs (Disab	1	Glob	3	Bo	22	All	54	Alzheimer's	99	Sm	3	Ra	20	15.3	35.0	6.64

	ility-Adjusted Life Years)		al		th		ages	3	diseases and other dementias		oking		te	01	7441324	9330098	6567367
2	DALYs (Disability-Adjusted Life Years)	1	Global	3	Both	22	All ages	543	Alzheimer's disease and other dementias	99	Smoking	1	Number	2002	966322.327689517	2227886.66253955	414902.986601115
2	DALYs (Disability-Adjusted Life Years)	1	Global	3	Both	22	All ages	543	Alzheimer's disease and other dementias	99	Smoking	3	Rate	2002	15.4478622	35.61553445	6.632739386
2	DALYs (Disability-Adjusted Life Years)	1	Global	3	Both	22	All ages	543	Alzheimer's disease and other dementias	99	Smoking	1	Number	2003	982161.580604808	2244376.70024324	422393.611151187
2	DALYs (Disability-Adjusted Life Years)	1	Global	3	Both	22	All ages	543	Alzheimer's disease and other dementias	99	Smoking	3	Rate	2003	15.50099586	35.42194546	6.666440377
2	DALYs (Disability-Adjusted Life Years)	1	Global	3	Both	22	All ages	543	Alzheimer's disease and other dementias	99	Smoking	1	Number	2004	998409.433038729	2275836.73629314	428183.757262774
2	DALYs	1	Gl	3	B	2	Al	5	Alzhei	9	S	3	R	2	15	35	6.

	(Disability-Adjusted Life Years)		obal		oth	2	l ages	43	mer's disease and other dementias	9	moking		ate	004	.55549384	.45816292	671220822
2	DALYs (Disability-Adjusted Life Years)	1	Global	3	Both	22	All ages	543	Alzheimer's disease and other dementias	99	Smoking	1	Number	2005	1017842.71873591	2320600.87501322	437510.066929733
2	DALYs (Disability-Adjusted Life Years)	1	Global	3	Both	22	All ages	543	Alzheimer's disease and other dementias	99	Smoking	3	Rate	2005	15.65346457	35.68866085	6.728493712
2	DALYs (Disability-Adjusted Life Years)	1	Global	3	Both	22	All ages	543	Alzheimer's disease and other dementias	99	Smoking	1	Number	2006	1038781.20503322	2379762.12902479	447497.161326945
2	DALYs (Disability-Adjusted Life Years)	1	Global	3	Both	22	All ages	543	Alzheimer's disease and other dementias	99	Smoking	3	Rate	2006	15.76605551	36.11873382	6.791868253
2	DALYs (Disability-Adjusted Life Years)	1	Global	3	Both	22	All ages	543	Alzheimer's disease and other dementias	99	Smoking	1	Number	2007	1064394.31208956	2434424.08308974	459648.497681863

2	DALYs (Disability-Adjusted Life Years)	1	Global	3	Both	22	All ages	543	Alzheimer's disease and other dementias	99	Smoking	3	Rate	2007	15.94028655	36.45774599	6.883660202
2	DALYs (Disability-Adjusted Life Years)	1	Global	3	Both	22	All ages	543	Alzheimer's disease and other dementias	99	Smoking	1	Number	2008	1091281.25130873	2512988.44114822	471121.878246415
2	DALYs (Disability-Adjusted Life Years)	1	Global	3	Both	22	All ages	543	Alzheimer's disease and other dementias	99	Smoking	3	Rate	2008	16.1228459	37.12748235	6.960465449
2	DALYs (Disability-Adjusted Life Years)	1	Global	3	Both	22	All ages	543	Alzheimer's disease and other dementias	99	Smoking	1	Number	2009	1119890.04215194	2571890.62560125	480417.468828717
2	DALYs (Disability-Adjusted Life Years)	1	Global	3	Both	22	All ages	543	Alzheimer's disease and other dementias	99	Smoking	3	Rate	2009	16.32240083	37.48531383	7.002086095
2	DALYs (Disability-Adjusted Life Years)	1	Global	3	Both	22	All ages	543	Alzheimer's disease and other dementias	99	Smoking	1	Number	2010	1148830.352540	2639915.656163	493341.0720671

															36	96	83
2	DALYs (Disability-Adjusted Life Years)	1	Global	3	Both	22	All ages	543	Alzheimer's disease and other dementias	99	Smoking	3	Rate	2010	16.52813528	37.98026663	7.097660644
2	DALYs (Disability-Adjusted Life Years)	1	Global	3	Both	22	All ages	543	Alzheimer's disease and other dementias	99	Smoking	1	Number	2011	1178591.23407392	2704116.89019389	507175.587650877
2	DALYs (Disability-Adjusted Life Years)	1	Global	3	Both	22	All ages	543	Alzheimer's disease and other dementias	99	Smoking	3	Rate	2011	16.74648044	38.42251605	7.20640525
2	DALYs (Disability-Adjusted Life Years)	1	Global	3	Both	22	All ages	543	Alzheimer's disease and other dementias	99	Smoking	1	Number	2012	1210157.52380477	2816668.82059349	522822.660461908
2	DALYs (Disability-Adjusted Life Years)	1	Global	3	Both	22	All ages	543	Alzheimer's disease and other dementias	99	Smoking	3	Rate	2012	16.98149324	39.52480697	7.336490746
2	DALYs (Disability-Adjusted Life	1	Global	3	Both	22	All ages	543	Alzheimer's disease and other dement	99	Smokin	1	Number	2013	1242025.8436	2905622.0437	537214.20579

	Years)								ias		g				6675	6274	1714
2	DALYs (Disability-Adjusted Life Years)	1	Global	3	Both	22	All ages	543	Alzheimer's disease and other dementias	99	Smoking	3	Rate	2013	17.211588884	40.265162212	7.44453916
2	DALYs (Disability-Adjusted Life Years)	1	Global	3	Both	22	All ages	543	Alzheimer's disease and other dementias	99	Smoking	1	Number	2014	1275465.98551752	2928063.52295693	550420.955848273
2	DALYs (Disability-Adjusted Life Years)	1	Global	3	Both	22	All ages	543	Alzheimer's disease and other dementias	99	Smoking	3	Rate	2014	17.457491143	40.076838815	7.533692964
2	DALYs (Disability-Adjusted Life Years)	1	Global	3	Both	22	All ages	543	Alzheimer's disease and other dementias	99	Smoking	1	Number	2015	1309260.90584726	3027029.13278562	565830.758852226
2	DALYs (Disability-Adjusted Life Years)	1	Global	3	Both	22	All ages	543	Alzheimer's disease and other dementias	99	Smoking	3	Rate	2015	17.70129986	40.92564754	7.650071799
2	DALYs (Disability-Adjusted	1	Global	3	Both	22	All ages	543	Alzheimer's disease and other	99	Smoki	1	Numbe	2016	1342911.22	3117316.08	582351.917

	Life Years)								dementias		ng		r		492182	613557	948525
2	DALYs (Disability-Adjusted Life Years)	1	Global	3	Both	22	All ages	543	Alzheimer’s disease and other dementias	99	Smoking	3	Rate	2016	17.937502259	41.638541194	7.778577497
2	DALYs (Disability-Adjusted Life Years)	1	Global	3	Both	22	All ages	543	Alzheimer’s disease and other dementias	99	Smoking	1	Number	2017	1377387.1797072	3190151.46259718	594375.38274236
2	DALYs (Disability-Adjusted Life Years)	1	Global	3	Both	22	All ages	543	Alzheimer’s disease and other dementias	99	Smoking	3	Rate	2017	18.181997761	42.111126887	7.845965135
2	DALYs (Disability-Adjusted Life Years)	1	Global	3	Both	22	All ages	543	Alzheimer’s disease and other dementias	99	Smoking	1	Number	2018	1409956.57350884	3266980.60053638	605811.551850031
2	DALYs (Disability-Adjusted Life Years)	1	Global	3	Both	22	All ages	543	Alzheimer’s disease and other dementias	99	Smoking	3	Rate	2018	18.401874044	42.638593371	7.906674628
2	DALYs (Disability-Adjust	1	Global	3	Both	22	All ages	543	Alzheimer’s disease and	99	Smok	1	Numb	2019	1446474.	3326850.	626848.8

	ed Life Years)								other dementias		ing		er		42555068	29490789	10245056
2	DALYs (Disability-Adjusted Life Years)	1	Global	3	Both	22	All ages	543	Alzheimer's disease and other dementias	99	Smoking	3	Rate	2019	18.67544328	42.95299917	8.093250179
2	DALYs (Disability-Adjusted Life Years)	1	Global	3	Both	22	All ages	543	Alzheimer's disease and other dementias	99	Smoking	1	Number	2020	1476668.38348735	3348725.90612074	637687.525668535
2	DALYs (Disability-Adjusted Life Years)	1	Global	3	Both	22	All ages	543	Alzheimer's disease and other dementias	99	Smoking	3	Rate	2020	18.87752146	42.8096422	8.152107869
2	DALYs (Disability-Adjusted Life Years)	1	Global	3	Both	22	All ages	543	Alzheimer's disease and other dementias	99	Smoking	1	Number	2021	1533213.53770383	3496419.97439078	662722.708565097
2	DALYs (Disability-Adjusted Life Years)	1	Global	3	Both	22	All ages	543	Alzheimer's disease and other dementias	99	Smoking	3	Rate	2021	19.42903174	44.30697551	8.39808691
2	DALYs (Disability-	1	Global	3	Bot	27	Age-st	543	Alzheimer's diseas	99	Smo	3	Rat	199	23.327	54.455	9.9927

	Adjusted Life Years)				h	andardized		e and other dementias		king		e	0	97861	36232	48523	
2	DALYs (Disability-Adjusted Life Years)	1	Global	3	Both	27	Age-standardized	543	Alzheimer's disease and other dementias	99	Smoking	3	Rate	1991	23.24491689	53.69685945	9.898586706
2	DALYs (Disability-Adjusted Life Years)	1	Global	3	Both	27	Age-standardized	543	Alzheimer's disease and other dementias	99	Smoking	3	Rate	1992	23.13047853	52.7130718	9.888646047
2	DALYs (Disability-Adjusted Life Years)	1	Global	3	Both	27	Age-standardized	543	Alzheimer's disease and other dementias	99	Smoking	3	Rate	1993	23.0239978	53.07657035	9.887566194
2	DALYs (Disability-Adjusted Life Years)	1	Global	3	Both	27	Age-standardized	543	Alzheimer's disease and other dementias	99	Smoking	3	Rate	1994	22.86720672	52.69775916	9.831111227
2	DALYs (Disability-Adjusted Life Years)	1	Global	3	Both	27	Age-standardiz	543	Alzheimer's disease and other dementias	99	Smoking	3	Rate	1995	22.7107886	52.47531119	9.762974333

							ed									
2	DALYs (Disability-Adjusted Life Years)	1	Global	3	Both	27	Age-standardized	543	Alzheimer’s disease and other dementias	99	Smoking	3	Rate	1996	22.500296672	52.081068836	9.6258343454
2	DALYs (Disability-Adjusted Life Years)	1	Global	3	Both	27	Age-standardized	543	Alzheimer’s disease and other dementias	99	Smoking	3	Rate	1997	22.211470023	51.048176652	9.5081296 3
2	DALYs (Disability-Adjusted Life Years)	1	Global	3	Both	27	Age-standardized	543	Alzheimer’s disease and other dementias	99	Smoking	3	Rate	1998	21.900025117	50.400150075	9.379563214
2	DALYs (Disability-Adjusted Life Years)	1	Global	3	Both	27	Age-standardized	543	Alzheimer’s disease and other dementias	99	Smoking	3	Rate	1999	21.590073325	50.061304022	9.213866727
2	DALYs (Disability-Adjusted Life Years)	1	Global	3	Both	27	Age-standardized	543	Alzheimer’s disease and other dementias	99	Smoking	3	Rate	2000	21.329723314	49.1294309	9.111669475
2	DALYs (Disability-	1	Global	3	Bot	27	Age-st	543	Alzheimer’s diseas	99	Smo	3	Rat	200	21.081	48.913	9.0625

	Adjusted Life Years)				h		andardized		e and other dementias		king		e	1	73901	0285	36283
2	DALYs (Disability-Adjusted Life Years)	1	Global	3	Both	27	Age-standardized	543	Alzheimer's disease and other dementias	99	Smoking	3	Rate	2002	20.86466611	48.40662635	8.918664346
2	DALYs (Disability-Adjusted Life Years)	1	Global	3	Both	27	Age-standardized	543	Alzheimer's disease and other dementias	99	Smoking	3	Rate	2003	20.63057162	47.95224442	8.802333576
2	DALYs (Disability-Adjusted Life Years)	1	Global	3	Both	27	Age-standardized	543	Alzheimer's disease and other dementias	99	Smoking	3	Rate	2004	20.37639446	47.06006853	8.666018237
2	DALYs (Disability-Adjusted Life Years)	1	Global	3	Both	27	Age-standardized	543	Alzheimer's disease and other dementias	99	Smoking	3	Rate	2005	20.17219907	46.63432968	8.601651502
2	DALYs (Disability-Adjusted Life Years)	1	Global	3	Both	27	Age-standardiz	543	Alzheimer's disease and other dementias	99	Smoking	3	Rate	2006	19.95704239	45.91140937	8.534672466

							ed										
2	DALYs (Disability-Adjusted Life Years)	1	Global	3	Both	27	Age-standardized	543	Alzheimer's disease and other dementias	99	Smoking	3	Rate	2007	19.80044547	45.82814798	8.491322148
2	DALYs (Disability-Adjusted Life Years)	1	Global	3	Both	27	Age-standardized	543	Alzheimer's disease and other dementias	99	Smoking	3	Rate	2008	19.66212257	45.36995039	8.431673241
2	DALYs (Disability-Adjusted Life Years)	1	Global	3	Both	27	Age-standardized	543	Alzheimer's disease and other dementias	99	Smoking	3	Rate	2009	19.52845744	45.32594671	8.341019394
2	DALYs (Disability-Adjusted Life Years)	1	Global	3	Both	27	Age-standardized	543	Alzheimer's disease and other dementias	99	Smoking	3	Rate	2010	19.39981816	44.88310802	8.279838192
2	DALYs (Disability-Adjusted Life Years)	1	Global	3	Both	27	Age-standardized	543	Alzheimer's disease and other dementias	99	Smoking	3	Rate	2011	19.26185675	44.56094919	8.243981036
2	DALYs (Disability-	1	Global	3	Bot	27	Age-st	543	Alzheimer's diseas	99	Smo	3	Rat	201	19.133	44.368	8.1811

	Adjusted Life Years)				h		andardized		e and other dementias		king		e	2	98238	08413	90268
2	DALYs (Disability-Adjusted Life Years)	1	Global	3	Both	27	Age-standardized	543	Alzheimer's disease and other dementias	99	Smoking	3	Rate	2013	19.01422412	44.56025267	8.147627298
2	DALYs (Disability-Adjusted Life Years)	1	Global	3	Both	27	Age-standardized	543	Alzheimer's disease and other dementias	99	Smoking	3	Rate	2014	18.91053921	44.17019018	8.095514774
2	DALYs (Disability-Adjusted Life Years)	1	Global	3	Both	27	Age-standardized	543	Alzheimer's disease and other dementias	99	Smoking	3	Rate	2015	18.81223228	43.79666065	8.115080006
2	DALYs (Disability-Adjusted Life Years)	1	Global	3	Both	27	Age-standardized	543	Alzheimer's disease and other dementias	99	Smoking	3	Rate	2016	18.68459624	43.72478709	8.039999282
2	DALYs (Disability-Adjusted Life Years)	1	Global	3	Both	27	Age-standardiz	543	Alzheimer's disease and other dementias	99	Smoking	3	Rate	2017	18.55418145	43.24750551	7.980969395

							ed										
2	DALYs (Disability-Adjusted Life Years)	1	Global	3	Both	27	Age-standardized	543	Alzheimer's disease and other dementias	99	Smoking	3	Rate	2018	18.39537057	42.56085503	7.88361252
2	DALYs (Disability-Adjusted Life Years)	1	Global	3	Both	27	Age-standardized	543	Alzheimer's disease and other dementias	99	Smoking	3	Rate	2019	18.27369837	42.00540574	7.909247104
2	DALYs (Disability-Adjusted Life Years)	1	Global	3	Both	27	Age-standardized	543	Alzheimer's disease and other dementias	99	Smoking	3	Rate	2020	18.12911782	41.28998316	7.801428159
2	DALYs (Disability-Adjusted Life Years)	1	Global	3	Both	27	Age-standardized	543	Alzheimer's disease and other dementias	99	Smoking	3	Rate	2021	18.35809318	42.07070102	7.903082991
3	YLDs (Years Lived with Disability)	1	Global	3	Both	22	All ages	543	Alzheimer's disease and other dementias	99	Smoking	1	Number	1990	266893.69483836	391752.479841494	162663.225675174
3	YLDs (Years Lived	1	Global	3	Bot	22	All ag	543	Alzheimer's diseas	99	Smo	3	Rat	199	5.0039	7.3449	3.0497

	with Disability)				h		es		e and other dementias		king		e	0	846	59488	69597
3	YLDs (Years Lived with Disability)	1	Global	3	Both	22	All ages	543	Alzheimer's disease and other dementias	99	Smoking	1	Number	1991	273508.917189464	400563.854276282	166781.143421795
3	YLDs (Years Lived with Disability)	1	Global	3	Both	22	All ages	543	Alzheimer's disease and other dementias	99	Smoking	3	Rate	1991	5.049537781	7.395233532	3.079123319
3	YLDs (Years Lived with Disability)	1	Global	3	Both	22	All ages	543	Alzheimer's disease and other dementias	99	Smoking	1	Number	1992	279843.344336614	411087.517315575	170420.842895558
3	YLDs (Years Lived with Disability)	1	Global	3	Both	22	All ages	543	Alzheimer's disease and other dementias	99	Smoking	3	Rate	1992	5.090645321	7.478115127	3.100134715
3	YLDs (Years Lived with Disability)	1	Global	3	Both	22	All ages	543	Alzheimer's disease and other dementias	99	Smoking	1	Number	1993	285793.051269488	420196.159282703	174252.206463662
3	YLDs (Years	1	Glob	3	Bo	22	Al l	54	Alzheimer's	99	Sm	3	Ra	19	5.12	7.53	3.12

	Lived with Disability)		al		th		ages	3	disease and other dementias		oking		te	93	5902251	6517873	5337629
3	YLDs (Years Lived with Disability)	1	Global	3	Both	22	All ages	543	Alzheimer's disease and other dementias	99	Smoking	1	Number	1994	291266.938978782	428304.176093191	176720.024726782
3	YLDs (Years Lived with Disability)	1	Global	3	Both	22	All ages	543	Alzheimer's disease and other dementias	99	Smoking	3	Rate	1994	5.154748188	7.579988938	3.127533905
3	YLDs (Years Lived with Disability)	1	Global	3	Both	22	All ages	543	Alzheimer's disease and other dementias	99	Smoking	1	Number	1995	296301.377192572	437237.286439198	179719.246715014
3	YLDs (Years Lived with Disability)	1	Global	3	Both	22	All ages	543	Alzheimer's disease and other dementias	99	Smoking	3	Rate	1995	5.176418719	7.638585063	3.139715656
3	YLDs (Years Lived with Disability)	1	Global	3	Both	22	All ages	543	Alzheimer's disease and other dementias	99	Smoking	1	Number	1996	300717.029747125	443678.136135222	182000.733967363
3	YLDs	1	Gl	3	B	2	Al	5	Alzhei	9	S	3	R	1	5.	7.	3.

	(Years Lived with Disability)		obal		oth	2	l ages	43	mer's disease and other dementias	9	moking		ate	996	186212143	651741369	138812648
3	YLDs (Years Lived with Disability)	1	Global	3	Both	22	All ages	543	Alzheimer's disease and other dementias	99	Smoking	1	Number	1997	304557.479992346	448075.155323303	183983.634611061
3	YLDs (Years Lived with Disability)	1	Global	3	Both	22	All ages	543	Alzheimer's disease and other dementias	99	Smoking	3	Rate	1997	5.185655029	7.629309195	3.132662051
3	YLDs (Years Lived with Disability)	1	Global	3	Both	22	All ages	543	Alzheimer's disease and other dementias	99	Smoking	1	Number	1998	307941.163882635	452428.079887959	186343.699775249
3	YLDs (Years Lived with Disability)	1	Global	3	Both	22	All ages	543	Alzheimer's disease and other dementias	99	Smoking	3	Rate	1998	5.177324553	7.606540733	3.13294202
3	YLDs (Years Lived with Disability)	1	Global	3	Both	22	All ages	543	Alzheimer's disease and other dementias	99	Smoking	1	Number	1999	311658.60673148	458201.798514205	188775.067066344

3	YLDs (Years Lived with Disability)	1	Global	3	Both	22	All ages	543	Alzheimer's disease and other dementias	99	Smoking	3	Rate	1999	5.1745743335	7.6076810199	3.1342969399
3	YLDs (Years Lived with Disability)	1	Global	3	Both	22	All ages	543	Alzheimer's disease and other dementias	99	Smoking	1	Number	2000	316001.539641242	465359.47645864545	191784.076097005
3	YLDs (Years Lived with Disability)	1	Global	3	Both	22	All ages	543	Alzheimer's disease and other dementias	99	Smoking	3	Rate	2000	5.181391044	7.630372391	3.144631179
3	YLDs (Years Lived with Disability)	1	Global	3	Both	22	All ages	543	Alzheimer's disease and other dementias	99	Smoking	1	Number	2001	321157.903787948	472953.726285295	194429.771431279
3	YLDs (Years Lived with Disability)	1	Global	3	Both	22	All ages	543	Alzheimer's disease and other dementias	99	Smoking	3	Rate	2001	5.199924761	7.65767793	3.148047022
3	YLDs (Years Lived with Disability)	1	Global	3	Both	22	All ages	543	Alzheimer's disease and other dementias	99	Smoking	1	Number	2002	326408.5181142	480825.3492211	198075.7933878

															11	02	65
3	YLDs (Years Lived with Disability)	1	Global	3	Both	22	All ages	543	Alzheimer’s disease and other dementias	99	Smoking	3	Rate	2002	5.218045432	7.686590202	3.166487489
3	YLDs (Years Lived with Disability)	1	Global	3	Both	22	All ages	543	Alzheimer’s disease and other dementias	99	Smoking	1	Number	2003	331735.536838671	489102.412605327	201147.300723305
3	YLDs (Years Lived with Disability)	1	Global	3	Both	22	All ages	543	Alzheimer’s disease and other dementias	99	Smoking	3	Rate	2003	5.235626484	7.719274122	3.174613564
3	YLDs (Years Lived with Disability)	1	Global	3	Both	22	All ages	543	Alzheimer’s disease and other dementias	99	Smoking	1	Number	2004	337393.172965582	496764.014139953	204054.117835396
3	YLDs (Years Lived with Disability)	1	Global	3	Both	22	All ages	543	Alzheimer’s disease and other dementias	99	Smoking	3	Rate	2004	5.256678523	7.739720106	3.179219335
3	YLDs (Years Lived with Disability)	1	Global	3	Both	22	All ages	543	Alzheimer’s disease and other dement	99	Smokin	1	Number	2005	343592.93328	505569.30587	208207.08300

									ias		g				0907	3717	1819
3	YLDs (Years Lived with Disability)	1	Global	3	Both	22	All ages	543	Alzheimer’s disease and other dementias	99	Smoking	3	Rate	2005	5.284136448	7.775180854	3.20202929
3	YLDs (Years Lived with Disability)	1	Global	3	Both	22	All ages	543	Alzheimer’s disease and other dementias	99	Smoking	1	Number	2006	350929.870392033	516457.059242398	212300.105638412
3	YLDs (Years Lived with Disability)	1	Global	3	Both	22	All ages	543	Alzheimer’s disease and other dementias	99	Smoking	3	Rate	2006	5.326222492	7.838504035	3.222175406
3	YLDs (Years Lived with Disability)	1	Global	3	Both	22	All ages	543	Alzheimer’s disease and other dementias	99	Smoking	1	Number	2007	359329.467562831	529789.002365314	218444.664997314
3	YLDs (Years Lived with Disability)	1	Global	3	Both	22	All ages	543	Alzheimer’s disease and other dementias	99	Smoking	3	Rate	2007	5.381290198	7.934078952	3.271410337
3	YLDs (Years Lived with Disabi	1	Global	3	Both	22	All ages	543	Alzheimer’s disease and other	99	Smoki	1	Numbe	2008	367930.113	541047.581	223761.823

	lity)								dementias		ng		r		078088	640984	062059
3	YLDs (Years Lived with Disability)	1	Global	3	Both	22	All ages	543	Alzheimer's disease and other dementias	99	Smoking	3	Rate	2008	5.435886037	7.99356424	3.305909809
3	YLDs (Years Lived with Disability)	1	Global	3	Both	22	All ages	543	Alzheimer's disease and other dementias	99	Smoking	1	Number	2009	377116.866550555	554487.0151	229455.664966624
3	YLDs (Years Lived with Disability)	1	Global	3	Both	22	All ages	543	Alzheimer's disease and other dementias	99	Smoking	3	Rate	2009	5.496479497	8.081649962	3.344317027
3	YLDs (Years Lived with Disability)	1	Global	3	Both	22	All ages	543	Alzheimer's disease and other dementias	99	Smoking	1	Number	2010	386343.848848595	568009.31727177	234731.072412633
3	YLDs (Years Lived with Disability)	1	Global	3	Both	22	All ages	543	Alzheimer's disease and other dementias	99	Smoking	3	Rate	2010	5.558299696	8.171907033	3.377058163
3	YLDs (Years Lived with	1	Global	3	Both	22	All ages	543	Alzheimer's disease and	99	Smok	1	Numb	2011	396141.3	582277.1	240783.2

	Disability)								other dementias		ing		er		16899712	94760176	81473655
3	YLDs (Years Lived with Disability)	1	Global	3	Both	22	All ages	543	Alzheimer’s disease and other dementias	99	Smoking	3	Rate	2011	5.628730829	8.273516186	3.421264639
3	YLDs (Years Lived with Disability)	1	Global	3	Both	22	All ages	543	Alzheimer’s disease and other dementias	99	Smoking	1	Number	2012	407005.933583781	597770.535209811	248240.919376968
3	YLDs (Years Lived with Disability)	1	Global	3	Both	22	All ages	543	Alzheimer’s disease and other dementias	99	Smoking	3	Rate	2012	5.711296566	8.388194184	3.483432042
3	YLDs (Years Lived with Disability)	1	Global	3	Both	22	All ages	543	Alzheimer’s disease and other dementias	99	Smoking	1	Number	2013	418077.947227905	614676.626507164	254717.087031457
3	YLDs (Years Lived with Disability)	1	Global	3	Both	22	All ages	543	Alzheimer’s disease and other dementias	99	Smoking	3	Rate	2013	5.793587765	8.517988108	3.529786273
3	YLDs (Years Lived	1	Global	3	Bot	22	All ag	543	Alzheimer’s diseas	99	Smo	1	Num	201	429520	631367	261635

	with Disability)				h		es		e and other dementias		king		ber	4	.921623933	.983574078	.615913513
3	YLDs (Years Lived with Disability)	1	Global	3	Both	22	All ages	543	Alzheimer's disease and other dementias	99	Smoking	3	Rate	2014	5.878916329	8.641626895	3.58104534
3	YLDs (Years Lived with Disability)	1	Global	3	Both	22	All ages	543	Alzheimer's disease and other dementias	99	Smoking	1	Number	2015	440261.276275111	648456.469918831	268047.561743692
3	YLDs (Years Lived with Disability)	1	Global	3	Both	22	All ages	543	Alzheimer's disease and other dementias	99	Smoking	3	Rate	2015	5.952363531	8.767177244	3.624021955
3	YLDs (Years Lived with Disability)	1	Global	3	Both	22	All ages	543	Alzheimer's disease and other dementias	99	Smoking	1	Number	2016	450816.355216642	664358.534548353	273411.499320547
3	YLDs (Years Lived with Disability)	1	Global	3	Both	22	All ages	543	Alzheimer's disease and other dementias	99	Smoking	3	Rate	2016	6.021633737	8.873954369	3.652005721
3	YLDs (Years	1	Glob	3	Bo	22	All	54	Alzheimer's	99	Sm	1	Nu	20	4611	6793	2802

	Lived with Disability)		al		th		ages	3	disease and other dementias		oking		mber	17	24.952205104	54.0235	28.089217477
3	YLDs (Years Lived with Disability)	1	Global	3	Both	22	All ages	543	Alzheimer's disease and other dementias	99	Smoking	3	Rate	2017	6.087012354	8.967713229	3.699109825
3	YLDs (Years Lived with Disability)	1	Global	3	Both	22	All ages	543	Alzheimer's disease and other dementias	99	Smoking	1	Number	2018	471350.825842444	696266.675334867	285892.577029542
3	YLDs (Years Lived with Disability)	1	Global	3	Both	22	All ages	543	Alzheimer's disease and other dementias	99	Smoking	3	Rate	2018	6.151777074	9.087238498	3.731291651
3	YLDs (Years Lived with Disability)	1	Global	3	Both	22	All ages	543	Alzheimer's disease and other dementias	99	Smoking	1	Number	2019	482502.817295427	713812.746233609	292747.887691553
3	YLDs (Years Lived with Disability)	1	Global	3	Both	22	All ages	543	Alzheimer's disease and other dementias	99	Smoking	3	Rate	2019	6.229597869	9.216042277	3.779670402
3	YLDs	1	Gl	3	B	2	Al	5	Alzhei	9	S	1	N	2	49	72	30

	(Years Lived with Disability)		obal		oth	2	l ages	43	mer's disease and other dementias	9	moking		umber	020	3280.731449989	7734.884620703	0047.494466936
3	YLDs (Years Lived with Disability)	1	Global	3	Both	22	All ages	543	Alzheimer's disease and other dementias	99	Smoking	3	Rate	2020	6.30603167	9.303260673	3.835765076
3	YLDs (Years Lived with Disability)	1	Global	3	Both	22	All ages	543	Alzheimer's disease and other dementias	99	Smoking	1	Number	2021	515882.185133517	759417.349430448	313106.130063068
3	YLDs (Years Lived with Disability)	1	Global	3	Both	22	All ages	543	Alzheimer's disease and other dementias	99	Smoking	3	Rate	2021	6.537309451	9.623410846	3.96771147
3	YLDs (Years Lived with Disability)	1	Global	3	Both	27	Age-standardized	543	Alzheimer's disease and other dementias	99	Smoking	3	Rate	1990	7.571718937	11.21947526	4.638164698
3	YLDs (Years Lived with Disability)	1	Global	3	Both	27	Age-standardiz	543	Alzheimer's disease and other dementias	99	Smoking	3	Rate	1991	7.564045296	11.20831641	4.622088134

							ed										
3	YLDs (Years Lived with Disability)	1	Global	3	Both	27	Age-standardized	543	Alzheimer's disease and other dementias	99	Smoking	3	Rate	1992	7.548696973	11.179028444	4.600494193
3	YLDs (Years Lived with Disability)	1	Global	3	Both	27	Age-standardized	543	Alzheimer's disease and other dementias	99	Smoking	3	Rate	1993	7.524948594	11.151296658	4.583848335
3	YLDs (Years Lived with Disability)	1	Global	3	Both	27	Age-standardized	543	Alzheimer's disease and other dementias	99	Smoking	3	Rate	1994	7.487548364	11.094690777	4.564485718
3	YLDs (Years Lived with Disability)	1	Global	3	Both	27	Age-standardized	543	Alzheimer's disease and other dementias	99	Smoking	3	Rate	1995	7.440599623	11.027058826	4.529312295
3	YLDs (Years Lived with Disability)	1	Global	3	Both	27	Age-standardized	543	Alzheimer's disease and other dementias	99	Smoking	3	Rate	1996	7.374169781	10.9059241	4.480689163
3	YLDs (Years Lived	1	Global	3	Bot	27	Age-st	543	Alzheimer's diseas	99	Smo	3	Rat	199	7.2888	10.764	4.4199

	with Disability)				h		andardized		e and other dementias		king		e	7	72518	06815	32765
3	YLDs (Years Lived with Disability)	1	Global	3	Both	27	Age-standardized	543	Alzheimer's disease and other dementias	99	Smoking	3	Rate	1998	7.19070027	10.62917973	4.36765043
3	YLDs (Years Lived with Disability)	1	Global	3	Both	27	Age-standardized	543	Alzheimer's disease and other dementias	99	Smoking	3	Rate	1999	7.094033145	10.49289798	4.30494123
3	YLDs (Years Lived with Disability)	1	Global	3	Both	27	Age-standardized	543	Alzheimer's disease and other dementias	99	Smoking	3	Rate	2000	7.007844647	10.40635919	4.253139994
3	YLDs (Years Lived with Disability)	1	Global	3	Both	27	Age-standardized	543	Alzheimer's disease and other dementias	99	Smoking	3	Rate	2001	6.931064934	10.28730314	4.213049766
3	YLDs (Years Lived with Disability)	1	Global	3	Both	27	Age-standardiz	543	Alzheimer's disease and other dementias	99	Smoking	3	Rate	2002	6.854175439	10.18361345	4.164600829

							ed										
3	YLDs (Years Lived with Disability)	1	Global	3	Both	27	Age-standardized	543	Alzheimer’s disease and other dementias	99	Smoking	3	Rate	2003	6.778634784	10.06355162	4.121754796
3	YLDs (Years Lived with Disability)	1	Global	3	Both	27	Age-standardized	543	Alzheimer’s disease and other dementias	99	Smoking	3	Rate	2004	6.702927721	9.961449079	4.079361997
3	YLDs (Years Lived with Disability)	1	Global	3	Both	27	Age-standardized	543	Alzheimer’s disease and other dementias	99	Smoking	3	Rate	2005	6.633485531	9.837303817	4.041940632
3	YLDs (Years Lived with Disability)	1	Global	3	Both	27	Age-standardized	543	Alzheimer’s disease and other dementias	99	Smoking	3	Rate	2006	6.572521991	9.739678391	3.99417335
3	YLDs (Years Lived with Disability)	1	Global	3	Both	27	Age-standardized	543	Alzheimer’s disease and other dementias	99	Smoking	3	Rate	2007	6.521954539	9.668715936	3.980606475
3	YLDs (Years Lived	1	Global	3	Bot	27	Age-st	543	Alzheimer’s diseas	99	Smo	3	Rat	20	6.4738	9.5789	3.9521

	with Disability)				h		andardized		e and other dementias		king		e	8	97644	1744	61406
3	YLDs (Years Lived with Disability)	1	Global	3	Both	27	Age-standardized	543	Alzheimer's disease and other dementias	99	Smoking	3	Rate	2009	6.430413184	9.51267053	3.918539374
3	YLDs (Years Lived with Disability)	1	Global	3	Both	27	Age-standardized	543	Alzheimer's disease and other dementias	99	Smoking	3	Rate	2010	6.387340632	9.442688006	3.886024215
3	YLDs (Years Lived with Disability)	1	Global	3	Both	27	Age-standardized	543	Alzheimer's disease and other dementias	99	Smoking	3	Rate	2011	6.34670669	9.368378224	3.864734731
3	YLDs (Years Lived with Disability)	1	Global	3	Both	27	Age-standardized	543	Alzheimer's disease and other dementias	99	Smoking	3	Rate	2012	6.316240234	9.332662314	3.862950966
3	YLDs (Years Lived with Disability)	1	Global	3	Both	27	Age-standardiz	543	Alzheimer's disease and other dementias	99	Smoking	3	Rate	2013	6.288216784	9.286986606	3.833266642

ed

3	YLDs (Years Lived with Disability)	1	Global	3	Both	27	Age-standardized	543	Alzheimer's disease and other dementias	99	Smoking	3	Rate	2014	6.263152351	9.262489538	3.817296008
3	YLDs (Years Lived with Disability)	1	Global	3	Both	27	Age-standardized	543	Alzheimer's disease and other dementias	99	Smoking	3	Rate	2015	6.22940084	9.237624747	3.795986721
3	YLDs (Years Lived with Disability)	1	Global	3	Both	27	Age-standardized	543	Alzheimer's disease and other dementias	99	Smoking	3	Rate	2016	6.184569661	9.174071454	3.753629787
3	YLDs (Years Lived with Disability)	1	Global	3	Both	27	Age-standardized	543	Alzheimer's disease and other dementias	99	Smoking	3	Rate	2017	6.131439066	9.088033832	3.727913544
3	YLDs (Years Lived with Disability)	1	Global	3	Both	27	Age-standardized	543	Alzheimer's disease and other dementias	99	Smoking	3	Rate	2018	6.076855777	9.024167181	3.695162518
3	YLDs (Years Lived	1	Global	3	Bot	27	Age-st	543	Alzheimer's diseas	99	Smo	3	Rat	201	6.0295	8.9605	3.6678

	with Disability)				h		andardized		e and other dementias		king		e	9	00339	41907	07595
3	YLDs (Years Lived with Disability)	1	Global	3	Both	27	Age-standardized	543	Alzheimer's disease and other dementias	99	Smoking	3	Rate	2020	5.994738671	8.887457182	3.650051391
3	YLDs (Years Lived with Disability)	1	Global	3	Both	27	Age-standardized	543	Alzheimer's disease and other dementias	99	Smoking	3	Rate	2021	6.115306893	9.031404679	3.719107343
4	YLLs (Years of Life Lost)	1	Global	3	Both	22	All ages	543	Alzheimer's disease and other dementias	99	Smoking	1	Number	1990	528021.505945507	1511230.3461629	122674.099556672
4	YLLs (Years of Life Lost)	1	Global	3	Both	22	All ages	543	Alzheimer's disease and other dementias	99	Smoking	3	Rate	1990	9.89986476	28.33402784	2.300014264
4	YLLs (Years of Life Lost)	1	Global	3	Both	22	All ages	543	Alzheimer's disease and other dementias	99	Smoking	1	Number	1991	540073.624733309	1548504.27170874	124875.525006954

4	YLLs (Years of Life Lost)	1	Global	3	Both	22	All ages	543	Alzheimer's disease and other dementias	99	Smoking	3	Rate	1991	9.970871153	28.58857731	2.305459317
4	YLLs (Years of Life Lost)	1	Global	3	Both	22	All ages	543	Alzheimer's disease and other dementias	99	Smoking	1	Number	1992	550955.613408778	1579160.44221039	129801.296305164
4	YLLs (Years of Life Lost)	1	Global	3	Both	22	All ages	543	Alzheimer's disease and other dementias	99	Smoking	3	Rate	1992	10.02246318	28.7265925	2.361222359
4	YLLs (Years of Life Lost)	1	Global	3	Both	22	All ages	543	Alzheimer's disease and other dementias	99	Smoking	1	Number	1993	562206.542089547	1613924.3171902	129910.121393019
4	YLLs (Years of Life Lost)	1	Global	3	Both	22	All ages	543	Alzheimer's disease and other dementias	99	Smoking	3	Rate	1993	10.08357539	28.94688396	2.330030701
4	YLLs (Years of Life Lost)	1	Global	3	Both	22	All ages	543	Alzheimer's disease and other dementias	99	Smoking	1	Number	1994	571951.2295203	1634438.553236	134056.6472192

															29	59	64
4	YLLs (Years of Life Lost)	1	Global	3	Both	22	All ages	543	Alzheimer's disease and other dementias	99	Smoking	3	Rate	1994	10.122220808	28.925765548	2.372491233
4	YLLs (Years of Life Lost)	1	Global	3	Both	22	All ages	543	Alzheimer's disease and other dementias	99	Smoking	1	Number	1995	581748.385099915	1673274.85833363	133687.496453992
4	YLLs (Years of Life Lost)	1	Global	3	Both	22	All ages	543	Alzheimer's disease and other dementias	99	Smoking	3	Rate	1995	10.163210037	29.23230186	2.3355353575
4	YLLs (Years of Life Lost)	1	Global	3	Both	22	All ages	543	Alzheimer's disease and other dementias	99	Smoking	1	Number	1996	5903355.338524696	1700923.26958335	138500.324872458
4	YLLs (Years of Life Lost)	1	Global	3	Both	22	All ages	543	Alzheimer's disease and other dementias	99	Smoking	3	Rate	1996	10.181358896	29.334384429	2.388597903
4	YLLs (Years of Life Lost)	1	Global	3	Both	22	All ages	543	Alzheimer's disease and other dement	99	Smokin	1	Number	1997	5967332.77045	1729034.4788	140847.46774

									ias		g				8946	7663	1027
4	YLLs (Years of Life Lost)	1	Global	3	Both	22	All ages	543	Alzheimer's disease and other dementias	99	Smoking	3	Rate	1997	10.16048035	29.44001356	2.398188938
4	YLLs (Years of Life Lost)	1	Global	3	Both	22	All ages	543	Alzheimer's disease and other dementias	99	Smoking	1	Number	1998	602853.270981569	1727663.45495727	141872.020195591
4	YLLs (Years of Life Lost)	1	Global	3	Both	22	All ages	543	Alzheimer's disease and other dementias	99	Smoking	3	Rate	1998	10.13559539	29.04669941	2.385252703
4	YLLs (Years of Life Lost)	1	Global	3	Both	22	All ages	543	Alzheimer's disease and other dementias	99	Smoking	1	Number	1999	609549.499448542	1759477.34022068	143387.106426777
4	YLLs (Years of Life Lost)	1	Global	3	Both	22	All ages	543	Alzheimer's disease and other dementias	99	Smoking	3	Rate	1999	10.12055861	29.21320346	2.38070512
4	YLLs (Years of Life Lost)	1	Global	3	Both	22	All ages	543	Alzheimer's disease and other	99	Smoki	1	Numbe	2000	618623.223	1781128.96	145608.519

									dementias		ng		r		628753	347499	690805
4	YLLs (Years of Life Lost)	1	Global	3	Both	22	All ages	543	Alzheimer's disease and other dementias	99	Smoking	3	Rate	2000	10.14339624	29.20468574	2.387503177
4	YLLs (Years of Life Lost)	1	Global	3	Both	22	All ages	543	Alzheimer's disease and other dementias	99	Smoking	1	Number	2001	628397.052642968	1784519.90252927	147545.915790333
4	YLLs (Years of Life Lost)	1	Global	3	Both	22	All ages	543	Alzheimer's disease and other dementias	99	Smoking	3	Rate	2001	10.17448848	28.89347924	2.388942174
4	YLLs (Years of Life Lost)	1	Global	3	Both	22	All ages	543	Alzheimer's disease and other dementias	99	Smoking	1	Number	2002	639913.809575306	1849831.85555954	150403.137583641
4	YLLs (Years of Life Lost)	1	Global	3	Both	22	All ages	543	Alzheimer's disease and other dementias	99	Smoking	3	Rate	2002	10.22981677	29.57185897	2.404380895
4	YLLs (Years of Life	1	Global	3	Both	22	All ages	543	Alzheimer's disease and	99	Smok	1	Numb	2003	650426.0	1854038.	154144.3

	Lost)								other dementias		ing		er		43766137	01259653	84639125
4	YLLs (Years of Life Lost)	1	Global	3	Both	22	All ages	543	Alzheimer’s disease and other dementias	99	Smoking	3	Rate	2003	10.26536938	29.26141291	2.432788571
4	YLLs (Years of Life Lost)	1	Global	3	Both	22	All ages	543	Alzheimer’s disease and other dementias	99	Smoking	1	Number	2004	661016.260073147	1878256.01449931	156046.611671514
4	YLLs (Years of Life Lost)	1	Global	3	Both	22	All ages	543	Alzheimer’s disease and other dementias	99	Smoking	3	Rate	2004	10.29881532	29.26374581	2.431249171
4	YLLs (Years of Life Lost)	1	Global	3	Both	22	All ages	543	Alzheimer’s disease and other dementias	99	Smoking	1	Number	2005	674249.785455003	1911297.93591526	158163.427219473
4	YLLs (Years of Life Lost)	1	Global	3	Both	22	All ages	543	Alzheimer’s disease and other dementias	99	Smoking	3	Rate	2005	10.36932813	29.39396626	2.432404889
4	YLLs (Years of	1	Global	3	Bot	22	All ag	543	Alzheimer’s diseas	99	Smo	1	Num	200	687851	194676	162782

	Life Lost)				h		es		e and other dementias		king		ber	6	.334641185	0.682	.833012126
4	YLLs (Years of Life Lost)	1	Global	3	Both	22	All ages	543	Alzheimer’s disease and other dementias	99	Smoking	3	Rate	2006	10.43983302	29.54687362	2.470629204
4	YLLs (Years of Life Lost)	1	Global	3	Both	22	All ages	543	Alzheimer’s disease and other dementias	99	Smoking	1	Number	2007	705064.844526729	2010118.54642945	167664.8874
4	YLLs (Years of Life Lost)	1	Global	3	Both	22	All ages	543	Alzheimer’s disease and other dementias	99	Smoking	3	Rate	2007	10.55899635	30.10337923	2.510936331
4	YLLs (Years of Life Lost)	1	Global	3	Both	22	All ages	543	Alzheimer’s disease and other dementias	99	Smoking	1	Number	2008	723351.138230642	2070756.55951065	171857.39209763
4	YLLs (Years of Life Lost)	1	Global	3	Both	22	All ages	543	Alzheimer’s disease and other dementias	99	Smoking	3	Rate	2008	10.68695987	30.59384451	2.539061536
4	YLLs (Years	1	Glob	3	Bo	22	All	54	Alzheimer’s	99	Sm	1	Nu	20	7427	2099	1757

	of Life Lost)		al		th		ages	3	disease and other dementias		oking		mber	09	73.175601389	616.14495558	92.716312425
4	YLLs (Years of Life Lost)	1	Global	3	Both	22	All ages	543	Alzheimer's disease and other dementias	99	Smoking	3	Rate	2009	10.825921333	30.60191181	2.562179384
4	YLLs (Years of Life Lost)	1	Global	3	Both	22	All ages	543	Alzheimer's disease and other dementias	99	Smoking	1	Number	2010	762486.503691764	2169141.8589822	181760.473661224
4	YLLs (Years of Life Lost)	1	Global	3	Both	22	All ages	543	Alzheimer's disease and other dementias	99	Smoking	3	Rate	2010	10.96983559	31.20727966	2.61497417
4	YLLs (Years of Life Lost)	1	Global	3	Both	22	All ages	543	Alzheimer's disease and other dementias	99	Smoking	1	Number	2011	782449.917174207	2265885.4369531	186402.866392828
4	YLLs (Years of Life Lost)	1	Global	3	Both	22	All ages	543	Alzheimer's disease and other dementias	99	Smoking	3	Rate	2011	11.11774961	32.19573084	2.648578969
4	YLLs	1	Gl	3	B	2	Al	5	Alzhei	9	S	1	N	2	80	23	18

	(Years of Life Lost)		obal		oth	2	l ages	43	mer's disease and other dementias	9	moking		umber	012	3151.590220988	08152.39767296	7784.691289714
4	YLLs (Years of Life Lost)	1	Global	3	Both	22	All ages	543	Alzheimer's disease and other dementias	99	Smoking	3	Rate	2012	11.270196667	32.38906801	2.63508213
4	YLLs (Years of Life Lost)	1	Global	3	Both	22	All ages	543	Alzheimer's disease and other dementias	99	Smoking	1	Number	2013	823947.896438846	2386148.17361457	195346.753975014
4	YLLs (Years of Life Lost)	1	Global	3	Both	22	All ages	543	Alzheimer's disease and other dementias	99	Smoking	3	Rate	2013	11.41800108	33.06646274	2.707051571
4	YLLs (Years of Life Lost)	1	Global	3	Both	22	All ages	543	Alzheimer's disease and other dementias	99	Smoking	1	Number	2014	845945.06389359	2399935.14778515	199558.440138122
4	YLLs (Years of Life Lost)	1	Global	3	Both	22	All ages	543	Alzheimer's disease and other dementias	99	Smoking	3	Rate	2014	11.5785751	32.84826703	2.731385861

4	YLLs (Years of Life Lost)	1	Global	3	Both	22	All ages	543	Alzheimer's disease and other dementias	99	Smoking	1	Number	2015	868999.629572148	2455872.0710494	205361.428657442
4	YLLs (Years of Life Lost)	1	Global	3	Both	22	All ages	543	Alzheimer's disease and other dementias	99	Smoking	3	Rate	2015	11.74893633	33.20356375	2.776501011
4	YLLs (Years of Life Lost)	1	Global	3	Both	22	All ages	543	Alzheimer's disease and other dementias	99	Smoking	1	Number	2016	892094.869705179	2535465.38872226	210242.907917458
4	YLLs (Years of Life Lost)	1	Global	3	Both	22	All ages	543	Alzheimer's disease and other dementias	99	Smoking	3	Rate	2016	11.91586885	33.86665933	2.808251681
4	YLLs (Years of Life Lost)	1	Global	3	Both	22	All ages	543	Alzheimer's disease and other dementias	99	Smoking	1	Number	2017	916262.227502093	2613344.58859202	222656.595386433
4	YLLs (Years of Life Lost)	1	Global	3	Both	22	All ages	543	Alzheimer's disease and other dement	99	Smokin	3	Rate	2017	12.09498526	34.49707226	2.939145757

									ias		g						
4	YLLs (Years of Life Lost)	1	Global	3	Both	22	All ages	543	Alzheimer's disease and other dementias	99	Smoking	1	Number	2018	938605.74766639	2702110.6253519	217791.1424292339
4	YLLs (Years of Life Lost)	1	Global	3	Both	22	All ages	543	Alzheimer's disease and other dementias	99	Smoking	3	Rate	2018	12.25009696	35.26626301	2.84247419
4	YLLs (Years of Life Lost)	1	Global	3	Both	22	All ages	543	Alzheimer's disease and other dementias	99	Smoking	1	Number	2019	963971.608255254	2688764.24293951	229472.499255719
4	YLLs (Years of Life Lost)	1	Global	3	Both	22	All ages	543	Alzheimer's disease and other dementias	99	Smoking	3	Rate	2019	12.44584542	34.7146574	2.962721338
4	YLLs (Years of Life Lost)	1	Global	3	Both	22	All ages	543	Alzheimer's disease and other dementias	99	Smoking	1	Number	2020	983387.652037361	2744293.96958298	228214.487442023
4	YLLs (Years of Life Lost)	1	Global	3	Both	22	All ages	543	Alzheimer's disease and other	99	Smoki	3	Rate	2020	12.5714897	35.0826691	2.91746199

									dementias		ng				9	2	2
4	YLLs (Years of Life Lost)	1	Global	3	Both	22	All ages	543	Alzheimer's disease and other dementias	99	Smoking	1	Number	2021	1017331.35257031	2876261.38953396	241831.61577932
4	YLLs (Years of Life Lost)	1	Global	3	Both	22	All ages	543	Alzheimer's disease and other dementias	99	Smoking	3	Rate	2021	12.89172229	36.44826533	3.064513862
4	YLLs (Years of Life Lost)	1	Global	3	Both	27	Age-standardized	543	Alzheimer's disease and other dementias	99	Smoking	3	Rate	1990	15.75625967	44.13263242	3.615148889
4	YLLs (Years of Life Lost)	1	Global	3	Both	27	Age-standardized	543	Alzheimer's disease and other dementias	99	Smoking	3	Rate	1991	15.68087159	44.25802339	3.657911406
4	YLLs (Years of Life Lost)	1	Global	3	Both	27	Age-standardized	543	Alzheimer's disease and other dementias	99	Smoking	3	Rate	1992	15.58178155	43.7811185	3.645137493
4	YLLs (Years of	1	Global	3	Bot	27	Age-st	543	Alzheimer's diseas	99	Smo	3	Rat	199	15.499	44.202	3.5327

	Life Lost)				h		andardized		e and other dementias		king		e	3	049921	044	2808
4	YLLs (Years of Life Lost)	1	Global	3	Both	27	Age-standardized	543	Alzheimer's disease and other dementias	99	Smoking	3	Rate	1994	15.379658835	43.799666261	3.566482608
4	YLLs (Years of Life Lost)	1	Global	3	Both	27	Age-standardized	543	Alzheimer's disease and other dementias	99	Smoking	3	Rate	1995	15.270188898	43.422927752	3.504318445
4	YLLs (Years of Life Lost)	1	Global	3	Both	27	Age-standardized	543	Alzheimer's disease and other dementias	99	Smoking	3	Rate	1996	15.126126694	42.841372044	3.502677186
4	YLLs (Years of Life Lost)	1	Global	3	Both	27	Age-standardized	543	Alzheimer's disease and other dementias	99	Smoking	3	Rate	1997	14.922597771	42.249699975	3.477986286
4	YLLs (Years of Life Lost)	1	Global	3	Both	27	Age-standardiz	543	Alzheimer's disease and other dementias	99	Smoking	3	Rate	1998	14.709548847	41.756645504	3.447158626

							ed									
4	YLLs (Years of Life Lost)	1	Global	3	Both	27	Age-standardized	543	Alzheimer's disease and other dementias	99	Smoking	3	Rate	1999	14.49604011	41.34238379	3.391986322
4	YLLs (Years of Life Lost)	1	Global	3	Both	27	Age-standardized	543	Alzheimer's disease and other dementias	99	Smoking	3	Rate	2000	14.32187849	40.30403527	3.353455331
4	YLLs (Years of Life Lost)	1	Global	3	Both	27	Age-standardized	543	Alzheimer's disease and other dementias	99	Smoking	3	Rate	2001	14.15067407	39.75688058	3.30752445
4	YLLs (Years of Life Lost)	1	Global	3	Both	27	Age-standardized	543	Alzheimer's disease and other dementias	99	Smoking	3	Rate	2002	14.01048566	39.65020292	3.276444651
4	YLLs (Years of Life Lost)	1	Global	3	Both	27	Age-standardized	543	Alzheimer's disease and other dementias	99	Smoking	3	Rate	2003	13.85193684	39.02784331	3.264795557
4	YLLs (Years of	1	Global	3	Bot	27	Age-st	543	Alzheimer's diseas	99	Smo	3	Rat	200	13.673	38.452	3.2018

	Life Lost)				h		andardized		e and other dementias		king		e	4	46674	47019	54504
4	YLLs (Years of Life Lost)	1	Global	3	Both	27	Age-standardized	543	Alzheimer's disease and other dementias	99	Smoking	3	Rate	2005	13.53871354	37.89844987	3.175937018
4	YLLs (Years of Life Lost)	1	Global	3	Both	27	Age-standardized	543	Alzheimer's disease and other dementias	99	Smoking	3	Rate	2006	13.3845204	37.69537211	3.149930551
4	YLLs (Years of Life Lost)	1	Global	3	Both	27	Age-standardized	543	Alzheimer's disease and other dementias	99	Smoking	3	Rate	2007	13.27849093	37.3087004	3.15393824
4	YLLs (Years of Life Lost)	1	Global	3	Both	27	Age-standardized	543	Alzheimer's disease and other dementias	99	Smoking	3	Rate	2008	13.18822492	37.31569343	3.124766729
4	YLLs (Years of Life Lost)	1	Global	3	Both	27	Age-standardiz	543	Alzheimer's disease and other dementias	99	Smoking	3	Rate	2009	13.09804426	36.54224294	3.088939759

ed

4	YLLs (Years of Life Lost)	1	Global	3	Both	27	Age-standardized	543	Alzheimer's disease and other dementias	99	Smoking	3	Rate	2010	13.01247753	36.51540639	3.094416733
4	YLLs (Years of Life Lost)	1	Global	3	Both	27	Age-standardized	543	Alzheimer's disease and other dementias	99	Smoking	3	Rate	2011	12.91515006	36.78620545	3.070028489
4	YLLs (Years of Life Lost)	1	Global	3	Both	27	Age-standardized	543	Alzheimer's disease and other dementias	99	Smoking	3	Rate	2012	12.81774215	36.39058393	2.994129775
4	YLLs (Years of Life Lost)	1	Global	3	Both	27	Age-standardized	543	Alzheimer's disease and other dementias	99	Smoking	3	Rate	2013	12.72600733	36.69752424	3.009144989
4	YLLs (Years of Life Lost)	1	Global	3	Both	27	Age-standardized	543	Alzheimer's disease and other dementias	99	Smoking	3	Rate	2014	12.64738686	35.65261447	2.970361334
4	YLLs (Years of	1	Global	3	Bot	27	Age-st	543	Alzheimer's diseas	99	Smo	3	Rat	201	12.582	35.336	2.9690

	Life Lost)				h		andardized		e and other dementias		king		e	5	83144	17551	71663
4	YLLs (Years of Life Lost)	1	Global	3	Both	27	Age-standardized	543	Alzheimer's disease and other dementias	99	Smoking	3	Rate	2016	12.500026657	35.432671922	2.943927634
4	YLLs (Years of Life Lost)	1	Global	3	Both	27	Age-standardized	543	Alzheimer's disease and other dementias	99	Smoking	3	Rate	2017	12.422742238	35.134000122	3.022279687
4	YLLs (Years of Life Lost)	1	Global	3	Both	27	Age-standardized	543	Alzheimer's disease and other dementias	99	Smoking	3	Rate	2018	12.318514479	35.128134431	2.846930051
4	YLLs (Years of Life Lost)	1	Global	3	Both	27	Age-standardized	543	Alzheimer's disease and other dementias	99	Smoking	3	Rate	2019	12.244198003	34.143404419	2.912150368
4	YLLs (Years of Life Lost)	1	Global	3	Both	27	Age-standardiz	543	Alzheimer's disease and other dementias	99	Smoking	3	Rate	2020	12.134379915	33.463010999	2.805935757

4	YLLs (Years of Life Lost)	1	Global	3	Both	27	ed Age-standardized	543	Alzheimer's disease and other dementias	99	Smoking	3	Rate	2021	12.24278629	34.28057038	2.901140305

Supplementary Table S6 burden indicators for smoking-related ADOD according to sex

measure_id	measure_name	location_id	location_name	sex_id	sex_name	age_id	age_name	cause_id	cause_name	rei_id	rei_name	metric_id	metric_name	year	val	upper	lower
1	Deaths	1	Global	1	Male	22	All ages	543	Alzheimer's disease and other dementias	99	Smoking	1	Number	1990	20500.1695788443	57869.8910522901	4679.58913054426
1	Deaths	1	Global	2	Female	22	All ages	543	Alzheimer's disease and other dementias	99	Smoking	1	Number	1990	11664.6519983196	31824.2031436356	2788.54726042049
1	Deaths	1	Global	3	Both	22	All ages	543	Alzheimer's disease and	99	Smok	1	Numb	1990	32164.82	89320.27	7445.508

									other dementias		ing		er		15771639	17364865	57756345
1	Deaths	1	Global	1	Male	22	All ages	543	Alzheimer's disease and other dementias	99	Smoking	3	Rate	1990	0.763296023	2.154706941	0.174238157
1	Deaths	1	Global	2	Female	22	All ages	543	Alzheimer's disease and other dementias	99	Smoking	3	Rate	1990	0.4405279	1.201874638	0.105312432
1	Deaths	1	Global	3	Both	22	All ages	543	Alzheimer's disease and other dementias	99	Smoking	3	Rate	1990	0.603057603	1.674664006	0.139595693
1	Deaths	1	Global	1	Male	22	All ages	543	Alzheimer's disease and other dementias	99	Smoking	1	Number	1991	21023.3324802233	60269.7550608788	4857.07889029609
1	Deaths	1	Global	2	Female	22	All ages	543	Alzheimer's disease and other dementias	99	Smoking	1	Number	1991	12053.4301497694	33052.3462988188	2896.7092701727
1	Deaths	1	Global	3	Bot	22	All ag	543	Alzheimer's diseas	99	Smo	1	Num	199	33076.	92335.	7679.9

					h		es		e and other dementias		king		ber	1	7626299927	3047334272	5154329063
1	Deaths	1	Global	1	Male	22	All ages	543	Alzheimer's disease and other dementias	99	Smoking	3	Rate	1991	0.770488895	2.208840122	0.178008202
1	Deaths	1	Global	2	Female	22	All ages	543	Alzheimer's disease and other dementias	99	Smoking	3	Rate	1991	0.448425701	1.229651756	0.107766741
1	Deaths	1	Global	3	Both	22	All ages	543	Alzheimer's disease and other dementias	99	Smoking	3	Rate	1991	0.610665145	1.704699849	0.141787719
1	Deaths	1	Global	1	Male	22	All ages	543	Alzheimer's disease and other dementias	99	Smoking	1	Number	1992	21508.9419956555	61001.6592019026	5018.07037460727
1	Deaths	1	Global	2	Female	22	All ages	543	Alzheimer's disease and other dementias	99	Smoking	1	Number	1992	12425.6855010224	34523.3146449399	2965.35390868484
1	Deaths	1	Glob	3	Bo	22	Al	54	Alzheimer's	99	Sm	1	Nu	19	3393	9436	7963

			al		th		ages	3	disease and other dementias		oking		mber	92	4.62749678	4.9436536631	.04865352222
1	Deaths	1	Global	1	Male	22	All ages	543	Alzheimer's disease and other dementias	99	Smoking	3	Rate	1992	0.776456428	2.202113448	0.18114852
1	Deaths	1	Global	2	Female	22	All ages	543	Alzheimer's disease and other dementias	99	Smoking	3	Rate	1992	0.455642985	1.265950772	0.108737881
1	Deaths	1	Global	3	Both	22	All ages	543	Alzheimer's disease and other dementias	99	Smoking	3	Rate	1992	0.617306633	1.716597763	0.144856246
1	Deaths	1	Global	1	Male	22	All ages	543	Alzheimer's disease and other dementias	99	Smoking	1	Number	1993	22009.3624788248	62510.148957122	4969.0329017542
1	Deaths	1	Global	2	Female	22	All ages	543	Alzheimer's disease and other dementias	99	Smoking	1	Number	1993	12804.2730962448	35420.1283382977	3036.4656949393
1	Deaths	1	Gl	3	B	2	Al	5	Alzhei	9	S	1	N	1	34	96	79

			obal		oth	2	l ages	43	mer's disease and other dementias	9	moking		umber	993	813.6355750697	542.2153066996	87.27606461882
1	Deaths	1	Global	1	Male	22	All ages	543	Alzheimer's disease and other dementias	99	Smoking	3	Rate	1993	0.783193023	2.224394849	0.176820746
1	Deaths	1	Global	2	Female	22	All ages	543	Alzheimer's disease and other dementias	99	Smoking	3	Rate	1993	0.463040642	1.280897311	0.109807641
1	Deaths	1	Global	3	Both	22	All ages	543	Alzheimer's disease and other dementias	99	Smoking	3	Rate	1993	0.624407389	1.731553502	0.143257494
1	Deaths	1	Global	1	Male	22	All ages	543	Alzheimer's disease and other dementias	99	Smoking	1	Number	1994	22457.4160026761	64341.8772949134	5192.71394056868
1	Deaths	1	Global	2	Female	22	All ages	543	Alzheimer's disease and other dementias	99	Smoking	1	Number	1994	13153.0515956043	36448.4019130174	3135.23694115359

1	Deaths	1	Global	3	Both	22	All ages	543	Alzheimer's disease and other dementias	99	Smoking	1	Number	1994	35610.4675982803	99874.0259709058	8299.12381204134
1	Deaths	1	Global	1	Male	22	All ages	543	Alzheimer's disease and other dementias	99	Smoking	3	Rate	1994	0.788461512	2.258990698	0.18231194
1	Deaths	1	Global	2	Female	22	All ages	543	Alzheimer's disease and other dementias	99	Smoking	3	Rate	1994	0.46938281	1.300706014	0.111884783
1	Deaths	1	Global	3	Both	22	All ages	543	Alzheimer's disease and other dementias	99	Smoking	3	Rate	1994	0.630222551	1.767538246	0.146875212
1	Deaths	1	Global	1	Male	22	All ages	543	Alzheimer's disease and other dementias	99	Smoking	1	Number	1995	22918.4122493694	64927.3124562048	5185.09953796129
1	Deaths	1	Global	2	Female	22	All ages	543	Alzheimer's disease and other dementias	99	Smoking	1	Number	1995	13496.12464130	37609.95742912	3174.427952216

1	Deaths	1	Global	3	Both	22	All ages	543	Alzheimer's disease and other dementias	99	Smoking	1	Number	1995	4436414.5368906737	83101349.653971224	28360.55257910997
1	Deaths	1	Global	1	Male	22	All ages	543	Alzheimer's disease and other dementias	99	Smoking	3	Rate	1995	0.794283906	2.250187265	0.179700106
1	Deaths	1	Global	2	Female	22	All ages	543	Alzheimer's disease and other dementias	99	Smoking	3	Rate	1995	0.475442822	1.324927324	0.111829064
1	Deaths	1	Global	3	Both	22	All ages	543	Alzheimer's disease and other dementias	99	Smoking	3	Rate	1995	0.636166096	1.770589968	0.146059803
1	Deaths	1	Global	1	Male	22	All ages	543	Alzheimer's disease and other dementias	99	Smoking	1	Number	1996	233342.5310373471	658668.9606777447	5349.73302625606
1	Deaths	1	Global	2	Female	22	All ages	543	Alzheimer's disease and other dement	99	Smokin	1	Number	1996	137776.747852	382422.121066	3287.1437873

									ias		g				9447	5157	7616
1	Deaths	1	Global	3	Both	22	All ages	543	Alzheimer’s disease and other dementias	99	Smoking	1	Number	1996	37119.2788902919	103983.586872991	8637.00043868813
1	Deaths	1	Global	1	Male	22	All ages	543	Alzheimer’s disease and other dementias	99	Smoking	3	Rate	1996	0.798639977	2.253636728	0.18303545
1	Deaths	1	Global	2	Female	22	All ages	543	Alzheimer’s disease and other dementias	99	Smoking	3	Rate	1996	0.479089727	1.329878978	0.114311218
1	Deaths	1	Global	3	Both	22	All ages	543	Alzheimer’s disease and other dementias	99	Smoking	3	Rate	1996	0.640164792	1.793316931	0.148955038
1	Deaths	1	Global	1	Male	22	All ages	543	Alzheimer’s disease and other dementias	99	Smoking	1	Number	1997	23701.7480581689	67688.3802522915	5475.89251771357
1	Deaths	1	Global	2	Femal	22	All ages	543	Alzheimer’s disease and other	99	Smoki	1	Numbe	1997	13984.7701	38840.7295	3334.15942

					e				dementias		ng		r		292524	760511	639018
1	Deaths	1	Global	3	Both	22	All ages	543	Alzheimer's disease and other dementias	99	Smoking	1	Number	1997	37686.5181874213	105844.384908588	8857.37764275985
1	Deaths	1	Global	1	Male	22	All ages	543	Alzheimer's disease and other dementias	99	Smoking	3	Rate	1997	0.800708584	2.28669493	0.184990328
1	Deaths	1	Global	2	Female	22	All ages	543	Alzheimer's disease and other dementias	99	Smoking	3	Rate	1997	0.480084708	1.333366667	0.114458725
1	Deaths	1	Global	3	Both	22	All ages	543	Alzheimer's disease and other dementias	99	Smoking	3	Rate	1997	0.641682754	1.802196639	0.150813255
1	Deaths	1	Global	1	Male	22	All ages	543	Alzheimer's disease and other dementias	99	Smoking	1	Number	1998	24055.6411714221	67876.1829606271	5608.78898006292
1	Deaths	1	Global	2	Fema	22	All ages	543	Alzheimer's disease and	99	Smok	1	Numb	1998	14181.10	39484.73	3352.313

					le				other dementias		ing		er		63116034	61364966	42439443
1	Deaths	1	Global	3	Both	22	All ages	543	Alzheimer's disease and other dementias	99	Smoking	1	Number	1998	38236.7474830255	108139.376992395	9017.85938933684
1	Deaths	1	Global	1	Male	22	All ages	543	Alzheimer's disease and other dementias	99	Smoking	3	Rate	1998	0.802563117	2.264538309	0.187124805
1	Deaths	1	Global	2	Female	22	All ages	543	Alzheimer's disease and other dementias	99	Smoking	3	Rate	1998	0.480629081	1.338225101	0.113617322
1	Deaths	1	Global	3	Both	22	All ages	543	Alzheimer's disease and other dementias	99	Smoking	3	Rate	1998	0.642863231	1.818115657	0.151614627
1	Deaths	1	Global	1	Male	22	All ages	543	Alzheimer's disease and other dementias	99	Smoking	1	Number	1999	24426.3943928274	69917.8636630026	5614.50934340824
1	Deaths	1	Global	2	Fem	22	All ag	543	Alzheimer's diseas	99	Smo	1	Num	199	14373.	40171.	3404.4

					ale		es		e and other dementias		king		ber	9	7859188371	6548995661	8794880404
1	Deaths	1	Global	3	Both	22	All ages	543	Alzheimer's disease and other dementias	99	Smoking	1	Number	1999	38800.1803116645	109494.331566109	90622.6093
1	Deaths	1	Global	1	Male	22	All ages	543	Alzheimer's disease and other dementias	99	Smoking	3	Rate	1999	0.804931336	2.304027295	0.185016848
1	Deaths	1	Global	2	Female	22	All ages	543	Alzheimer's disease and other dementias	99	Smoking	3	Rate	1999	0.481003652	1.344302247	0.113927614
1	Deaths	1	Global	3	Both	22	All ages	543	Alzheimer's disease and other dementias	99	Smoking	3	Rate	1999	0.644212651	1.817971799	0.150463815
1	Deaths	1	Global	1	Male	22	All ages	543	Alzheimer's disease and other dementias	99	Smoking	1	Number	2000	24920.379208415	70371.4836580435	5739.37197262953
1	Deaths	1	Glob	2	Fe	22	Al l	54	Alzheimer's	99	Sm	1	Nu	20	1457	4055	3506

			al		male		ages	3	disease and other dementias		oking		mber	00	2.8022855781	6.8595960295	.41258116613
1	Deaths	1	Global	3	Both	22	All ages	543	Alzheimer's disease and other dementias	99	Smoking	1	Number	2000	39493.1814939931	111418.63536259	9272.7366065946 1
1	Deaths	1	Global	1	Male	22	All ages	543	Alzheimer's disease and other dementias	99	Smoking	3	Rate	2000	0.811160345	2.290597446	0.186817019
1	Deaths	1	Global	2	Female	22	All ages	543	Alzheimer's disease and other dementias	99	Smoking	3	Rate	2000	0.481492606	1.340018729	0.115853608
1	Deaths	1	Global	3	Both	22	All ages	543	Alzheimer's disease and other dementias	99	Smoking	3	Rate	2000	0.647558924	1.82690097	0.152042533
1	Deaths	1	Global	1	Male	22	All ages	543	Alzheimer's disease and other dementias	99	Smoking	1	Number	2001	25439.3712493463	71898.8792268252	5854.00396587063
1	Deaths	1	Gl	2	F	2	Al	5	Alzhei	9	S	1	N	2	14	40	35

			obal		emale	2	l ages	43	mer's disease and other dementias	9	moking		umber	001	771.2865645889	966.3808676145	02.14548270382
1	Deaths	1	Global	3	Both	22	All ages	543	Alzheimer's disease and other dementias	99	Smoking	1	Number	2001	40210.6578139352	112865.260094444	9407.59836191312
1	Deaths	1	Global	1	Male	22	All ages	543	Alzheimer's disease and other dementias	99	Smoking	3	Rate	2001	0.817859317	2.311502422	0.188202438
1	Deaths	1	Global	2	Female	22	All ages	543	Alzheimer's disease and other dementias	99	Smoking	3	Rate	2001	0.481821068	1.336272591	0.114235647
1	Deaths	1	Global	3	Both	22	All ages	543	Alzheimer's disease and other dementias	99	Smoking	3	Rate	2001	0.651057915	1.827421507	0.152320099
1	Deaths	1	Global	1	Male	22	All ages	543	Alzheimer's disease and other dementias	99	Smoking	1	Number	2002	26044.369762568	73618.1832943283	6027.91231954273

1	Deaths	1	Global	2	Female	22	All ages	543	Alzheimer's disease and other dementias	99	Smoking	1	Number	2002	14950.0441560751	41416.8466792618	3550.8695583685
1	Deaths	1	Global	3	Both	22	All ages	543	Alzheimer's disease and other dementias	99	Smoking	1	Number	2002	40994.4139186431	114882.146565572	9640.955178
1	Deaths	1	Global	1	Male	22	All ages	543	Alzheimer's disease and other dementias	99	Smoking	3	Rate	2002	0.826905765	2.33736891	0.191385528
1	Deaths	1	Global	2	Female	22	All ages	543	Alzheimer's disease and other dementias	99	Smoking	3	Rate	2002	0.48136487	1.333548904	0.114331693
1	Deaths	1	Global	3	Both	22	All ages	543	Alzheimer's disease and other dementias	99	Smoking	3	Rate	2002	0.655346605	1.836533751	0.154122639
1	Deaths	1	Global	1	Male	22	All ages	543	Alzheimer's disease and other dementias	99	Smoking	1	Number	2003	26615.66980565	75831.25594115	6251.276974

1	Deaths	1	Global	2	Female	22	All ages	543	Alzheimer's disease and other dementias	99	Smoking	1	Number	2003	7415111.6479790106	7241866.9396358744	3637.20454992275
1	Deaths	1	Global	3	Both	22	All ages	543	Alzheimer's disease and other dementias	99	Smoking	1	Number	2003	41727.317784668	116936.619805441	9826.96728
1	Deaths	1	Global	1	Male	22	All ages	543	Alzheimer's disease and other dementias	99	Smoking	3	Rate	2003	0.83448823	2.377557694	0.195997963
1	Deaths	1	Global	2	Female	22	All ages	543	Alzheimer's disease and other dementias	99	Smoking	3	Rate	2003	0.480244212	1.330520368	0.115589407
1	Deaths	1	Global	3	Both	22	All ages	543	Alzheimer's disease and other dementias	99	Smoking	3	Rate	2003	0.658562698	1.845555859	0.155094418
1	Deaths	1	Global	1	Male	22	All ages	543	Alzheimer's disease and other dement	99	Smokin	1	Number	2004	27189.388524	76825.848475	6221.1237152

									ias		g				7741	1148	3545
1	Deaths	1	Global	2	Female	22	All ages	543	Alzheimer's disease and other dementias	99	Smoking	1	Number	2004	15242.2427828824	42193.5498873769	3660.34330634693
1	Deaths	1	Global	3	Both	22	All ages	543	Alzheimer's disease and other dementias	99	Smoking	1	Number	2004	42431.6313076565	118749.846985734	9871.6157429278 6
1	Deaths	1	Global	1	Male	22	All ages	543	Alzheimer's disease and other dementias	99	Smoking	3	Rate	2004	0.841784397	2.378530892	0.192606203
1	Deaths	1	Global	2	Female	22	All ages	543	Alzheimer's disease and other dementias	99	Smoking	3	Rate	2004	0.478052846	1.323345055	0.114801841
1	Deaths	1	Global	3	Both	22	All ages	543	Alzheimer's disease and other dementias	99	Smoking	3	Rate	2004	0.661096498	1.850155309	0.153802491
1	Deaths	1	Global	1	Male	22	All ages	543	Alzheimer's disease and other	99	Smoki	1	Numbe	2005	27871.0885	79055.1650	6509.55610

									dementias		ng		r		529134	84611	158262
1	Deaths	1	Global	2	Female	22	All ages	543	Alzheimer's disease and other dementias	99	Smoking	1	Number	2005	15486.3830733097	43161.2822157172	3704.93010973942
1	Deaths	1	Global	3	Both	22	All ages	543	Alzheimer's disease and other dementias	99	Smoking	1	Number	2005	43357.4716262231	122075.93167303	10045.6240933224
1	Deaths	1	Global	1	Male	22	All ages	543	Alzheimer's disease and other dementias	99	Smoking	3	Rate	2005	0.851994644	2.416646811	0.1989914
1	Deaths	1	Global	2	Female	22	All ages	543	Alzheimer's disease and other dementias	99	Smoking	3	Rate	2005	0.479295397	1.335818945	0.114665635
1	Deaths	1	Global	3	Both	22	All ages	543	Alzheimer's disease and other dementias	99	Smoking	3	Rate	2005	0.666797172	1.877413118	0.154492259
1	Deaths	1	Global	1	Male	22	All ages	543	Alzheimer's disease and	99	Smok	1	Numb	2006	28606.70	80988.02	6742.506

									other dementias		ing		er		70447281	11244288	44857015
1	Deaths	1	Global	2	Female	22	All ages	543	Alzheimer's disease and other dementias	99	Smoking	1	Number	2006	15697.7233798077	43809.6294030783	3808.24262634199
1	Deaths	1	Global	3	Both	22	All ages	543	Alzheimer's disease and other dementias	99	Smoking	1	Number	2006	44304.4304245358	124578.624908599	10423.2961952058
1	Deaths	1	Global	1	Male	22	All ages	543	Alzheimer's disease and other dementias	99	Smoking	3	Rate	2006	0.863250031	2.443934271	0.203465184
1	Deaths	1	Global	2	Female	22	All ages	543	Alzheimer's disease and other dementias	99	Smoking	3	Rate	2006	0.479337104	1.337746907	0.116286416
1	Deaths	1	Global	3	Both	22	All ages	543	Alzheimer's disease and other dementias	99	Smoking	3	Rate	2006	0.672428521	1.890786536	0.158199114
1	Deaths	1	Global	1	Mal	22	All ag	543	Alzheimer's diseas	99	Smo	1	Num	200	29538.	83909.	6901.8

					e		es		e and other dementias		king		ber	7	4147838393	6350527209	0913390437
1	Deaths	1	Global	2	Female	22	All ages	543	Alzheimer's disease and other dementias	99	Smoking	1	Number	2007	15929.7343129903	44167.9743571562	3862.34953204972
1	Deaths	1	Global	3	Both	22	All ages	543	Alzheimer's disease and other dementias	99	Smoking	1	Number	2007	45468.1490968297	127146.89672465	10538.8526105519
1	Deaths	1	Global	1	Male	22	All ages	543	Alzheimer's disease and other dementias	99	Smoking	3	Rate	2007	0.879736533	2.499063405	0.205555162
1	Deaths	1	Global	2	Female	22	All ages	543	Alzheimer's disease and other dementias	99	Smoking	3	Rate	2007	0.479848486	1.330464164	0.116344879
1	Deaths	1	Global	3	Both	22	All ages	543	Alzheimer's disease and other dementias	99	Smoking	3	Rate	2007	0.680927469	1.90414205	0.157829038
1	Deaths	1	Glob	1	Ma	22	Al l	54	Alzheimer's	99	Sm	1	Nu	20	3052	8708	7131

			al		le		ages	3	disease and other dementias		oking		mber	08	0.299744829	1.7058566988	.0720563529
1	Deaths	1	Global	2	Female	22	All ages	543	Alzheimer's disease and other dementias	99	Smoking	1	Number	2008	16185.8289781781	44625.3330387719	3924.86908650132
1	Deaths	1	Global	3	Both	22	All ages	543	Alzheimer's disease and other dementias	99	Smoking	1	Number	2008	46706.1287230072	130947.069443532	10878.8661145605
1	Deaths	1	Global	1	Male	22	All ages	543	Alzheimer's disease and other dementias	99	Smoking	3	Rate	2008	0.896900756	2.559072108	0.20956098
1	Deaths	1	Global	2	Female	22	All ages	543	Alzheimer's disease and other dementias	99	Smoking	3	Rate	2008	0.480908518	1.325894573	0.116614539
1	Deaths	1	Global	3	Both	22	All ages	543	Alzheimer's disease and other dementias	99	Smoking	3	Rate	2008	0.690047332	1.934642806	0.160726927
1	Deaths	1	Gl	1	M	2	Al	5	Alzhei	9	S	1	N	2	31	89	72

			obal		ale	2	l ages	43	mer's disease and other dementias	9	moking		umber	009	571.4608798462	307.8575306693	97.31292277003
1	Deaths	1	Global	2	Female	22	All ages	543	Alzheimer's disease and other dementias	99	Smoking	1	Number	2009	16433.5351725789	45580.852254186	4000.84646529023
1	Deaths	1	Global	3	Both	22	All ages	543	Alzheimer's disease and other dementias	99	Smoking	1	Number	2009	48004.9960524251	134384.568641223	11079.9221241796
1	Deaths	1	Global	1	Male	22	All ages	543	Alzheimer's disease and other dementias	99	Smoking	3	Rate	2009	0.915457483	2.589602893	0.211595521
1	Deaths	1	Global	2	Female	22	All ages	543	Alzheimer's disease and other dementias	99	Smoking	3	Rate	2009	0.481589483	1.335760009	0.117245958
1	Deaths	1	Global	3	Both	22	All ages	543	Alzheimer's disease and other dementias	99	Smoking	3	Rate	2009	0.699672966	1.958655504	0.161489899

1	Deaths	1	Global	1	Male	22	All ages	543	Alzheimer’s disease and other dementias	99	Smoking	1	Number	2010	32653.7431753285	91680.313756501	7764.73209168283
1	Deaths	1	Global	2	Female	22	All ages	543	Alzheimer’s disease and other dementias	99	Smoking	1	Number	2010	16761.8044163187	45804.3052485865	4124.75760601141
1	Deaths	1	Global	3	Both	22	All ages	543	Alzheimer’s disease and other dementias	99	Smoking	1	Number	2010	49415.5475916472	136773.542007644	11620.8585404757
1	Deaths	1	Global	1	Male	22	All ages	543	Alzheimer’s disease and other dementias	99	Smoking	3	Rate	2010	0.934918137	2.624923816	0.222314141
1	Deaths	1	Global	2	Female	22	All ages	543	Alzheimer’s disease and other dementias	99	Smoking	3	Rate	2010	0.484715417	1.324562222	0.119279139
1	Deaths	1	Global	3	Both	22	All ages	543	Alzheimer’s disease and other dement	99	Smokin	3	Rate	2010	0.710937741	1.967750591	0.167188412

									ias		g						
1	Deaths	1	Global	1	Male	22	All ages	543	Alzheimer's disease and other dementias	99	Smoking	1	Number	2011	33741.3062659608	95358.3285422682	8062.60495971881
1	Deaths	1	Global	2	Female	22	All ages	543	Alzheimer's disease and other dementias	99	Smoking	1	Number	2011	17075.1959368627	46954.3764019386	4189.37634291112
1	Deaths	1	Global	3	Both	22	All ages	543	Alzheimer's disease and other dementias	99	Smoking	1	Number	2011	50816.5022028235	141152.044669737	11911.4288634391
1	Deaths	1	Global	1	Male	22	All ages	543	Alzheimer's disease and other dementias	99	Smoking	3	Rate	2011	0.954435877	2.697388454	0.228065842
1	Deaths	1	Global	2	Female	22	All ages	543	Alzheimer's disease and other dementias	99	Smoking	3	Rate	2011	0.487495719	1.340544354	0.119606419
1	Deaths	1	Global	3	Both	22	All ages	543	Alzheimer's disease and other	99	Smoki	3	Rate	2011	0.72204640	2.00561474	0.16924825

									dementias		ng				2	3	6
1	Deaths	1	Global	1	Male	22	All ages	543	Alzheimer's disease and other dementias	99	Smoking	1	Number	2012	34909.3731563639	98484.0204091394	8220.66316092633
1	Deaths	1	Global	2	Female	22	All ages	543	Alzheimer's disease and other dementias	99	Smoking	1	Number	2012	17364.4238815159	47434.5432313447	4250.25739425293
1	Deaths	1	Global	3	Both	22	All ages	543	Alzheimer's disease and other dementias	99	Smoking	1	Number	2012	52273.7970378798	146183.097898319	12101.5661404152
1	Deaths	1	Global	1	Male	22	All ages	543	Alzheimer's disease and other dementias	99	Smoking	3	Rate	2012	0.975485015	2.751973968	0.229712911
1	Deaths	1	Global	2	Female	22	All ages	543	Alzheimer's disease and other dementias	99	Smoking	3	Rate	2012	0.484960874	1.337064401	0.119804418
1	Deaths	1	Global	3	Both	22	All ages	543	Alzheimer's disease and	99	Smok	3	Rate	2012	0.733530	2.051309	0.169814

									other dementias		ing				234	222	805
1	Deaths	1	Global	1	Male	22	All ages	543	Alzheimer's disease and other dementias	99	Smoking	1	Number	2013	36141.6846997713	104360.604590308	8498.79784929237
1	Deaths	1	Global	2	Female	22	All ages	543	Alzheimer's disease and other dementias	99	Smoking	1	Number	2013	17640.5862610801	48098.7143438191	4291.49462169116
1	Deaths	1	Global	3	Both	22	All ages	543	Alzheimer's disease and other dementias	99	Smoking	1	Number	2013	53782.2709608514	152445.290621435	12535.6909398835
1	Deaths	1	Global	1	Male	22	All ages	543	Alzheimer's disease and other dementias	99	Smoking	3	Rate	2013	0.997538628	2.880433915	0.234573436
1	Deaths	1	Global	2	Female	22	All ages	543	Alzheimer's disease and other dementias	99	Smoking	3	Rate	2013	0.490952889	1.338629137	0.119436035
1	Deaths	1	Global	3	Bot	22	All ag	543	Alzheimer's diseas	99	Smo	3	Rat	201	0.7452	2.1125	0.1737

					h		es		e and other dementias		king		e	3	97161	37091	15514
1	Deaths	1	Global	1	Male	22	All ages	543	Alzheimer’s disease and other dementias	99	Smoking	1	Number	2014	37374.7694692129	107223.525667988	8651.81799074541
1	Deaths	1	Global	2	Female	22	All ages	543	Alzheimer’s disease and other dementias	99	Smoking	1	Number	2014	17937.6325540351	49089.2247537906	4314.02090562379
1	Deaths	1	Global	3	Both	22	All ages	543	Alzheimer’s disease and other dementias	99	Smoking	1	Number	2014	55312.4020232481	156771.105603881	13015.0819187131
1	Deaths	1	Global	1	Male	22	All ages	543	Alzheimer’s disease and other dementias	99	Smoking	3	Rate	2014	1.019021613	2.92344519	0.235891476
1	Deaths	1	Global	2	Female	22	All ages	543	Alzheimer’s disease and other dementias	99	Smoking	3	Rate	2014	0.49300706	1.349193341	0.118568755
1	Deaths	1	Glob	3	Bo	22	Al l	54	Alzhei mer’s	99	Sm	3	Ra	20	0.75	2.14	0.17

			al		th		ages	3	disease and other dementias		oking		te	14	7069021	574929	813935
1	Deaths	1	Global	1	Male	22	All ages	543	Alzheimer's disease and other dementias	99	Smoking	1	Number	2015	38617.6986497893	110634.603543461	9245.85928400026
1	Deaths	1	Global	2	Female	22	All ages	543	Alzheimer's disease and other dementias	99	Smoking	1	Number	2015	18280.1851355258	50112.4327812462	4398.63552059468
1	Deaths	1	Global	3	Both	22	All ages	543	Alzheimer's disease and other dementias	99	Smoking	1	Number	2015	56897.8837853151	161821.48558782	13362.0381968368
1	Deaths	1	Global	1	Male	22	All ages	543	Alzheimer's disease and other dementias	99	Smoking	3	Rate	2015	1.04012882	2.979831622	0.249027908
1	Deaths	1	Global	2	Female	22	All ages	543	Alzheimer's disease and other dementias	99	Smoking	3	Rate	2015	0.496254552	1.3604087	0.119410328
1	Deaths	1	Gl	3	B	2	Al	5	Alzhei	9	S	3	R	2	0.	2.	0.

			obal		oth	2	l ages	43	mer's disease and other dementias	9	moking		ate	015	769263405	187837907	1806557
1	Deaths	1	Global	1	Male	22	All ages	543	Alzheimer's disease and other dementias	99	Smoking	1	Number	2016	39859.27660796633	114520.854413089	9448.88772535448
1	Deaths	1	Global	2	Female	22	All ages	543	Alzheimer's disease and other dementias	99	Smoking	1	Number	2016	18652.7898009354	50683.4161052816	4409.64518339814
1	Deaths	1	Global	3	Both	22	All ages	543	Alzheimer's disease and other dementias	99	Smoking	1	Number	2016	58512.0664088988	165139.635349184	13656.9284176308
1	Deaths	1	Global	1	Male	22	All ages	543	Alzheimer's disease and other dementias	99	Smoking	3	Rate	2016	1.060654565	3.047397679	0.251434716
1	Deaths	1	Global	2	Female	22	All ages	543	Alzheimer's disease and other dementias	99	Smoking	3	Rate	2016	0.500259404	1.359306346	0.118264694

1	Deaths	1	Global	3	Both	22	All ages	543	Alzheimer’s disease and other dementias	99	Smoking	3	Rate	2016	0.781556013	2.205799297	0.182418007
1	Deaths	1	Global	1	Male	22	All ages	543	Alzheimer’s disease and other dementias	99	Smoking	1	Number	2017	41198.2597972633	120151.784476532	9981.12922174401
1	Deaths	1	Global	2	Female	22	All ages	543	Alzheimer’s disease and other dementias	99	Smoking	1	Number	2017	18991.7036867749	51589.8552860916	4475.88770394617
1	Deaths	1	Global	3	Both	22	All ages	543	Alzheimer’s disease and other dementias	99	Smoking	1	Number	2017	60189.9634840381	171965.105422098	14255.7462388559
1	Deaths	1	Global	1	Male	22	All ages	543	Alzheimer’s disease and other dementias	99	Smoking	3	Rate	2017	1.083415702	3.159704574	0.262479827
1	Deaths	1	Global	2	Female	22	All ages	543	Alzheimer’s disease and other dement	99	Smokin	3	Rate	2017	0.503367832	1.367369355	0.118631689

									ias		g						
1	Deaths	1	Global	3	Both	22	All ages	543	Alzheimer’s disease and other dementias	99	Smoking	3	Rate	2017	0.794528792	2.270000173	0.188180889
1	Deaths	1	Global	1	Male	22	All ages	543	Alzheimer’s disease and other dementias	99	Smoking	1	Number	2018	42435.7406861115	120491.369476179	10014.6493827107
1	Deaths	1	Global	2	Female	22	All ages	543	Alzheimer’s disease and other dementias	99	Smoking	1	Number	2018	19330.5983124783	52194.829540104	4721.47547254407
1	Deaths	1	Global	3	Both	22	All ages	543	Alzheimer’s disease and other dementias	99	Smoking	1	Number	2018	61766.3389985898	170823.21939835	14380.745220821
1	Deaths	1	Global	1	Male	22	All ages	543	Alzheimer’s disease and other dementias	99	Smoking	3	Rate	2018	1.103365651	3.132878941	0.260389473
1	Deaths	1	Global	2	Femal	22	All ages	543	Alzheimer’s disease and other	99	Smoki	3	Rate	2018	0.50656702	1.36778899	0.12372838

					e				dementias		ng				8	7	9
1	Deaths	1	Global	3	Both	22	All ages	543	Alzheimer's disease and other dementias	99	Smoking	3	Rate	2018	0.806135743	2.229478145	0.18768852
1	Deaths	1	Global	1	Male	22	All ages	543	Alzheimer's disease and other dementias	99	Smoking	1	Number	2019	43865.8886755695	123851.174213595	10251.4195255282
1	Deaths	1	Global	2	Female	22	All ages	543	Alzheimer's disease and other dementias	99	Smoking	1	Number	2019	19664.1670571986	51475.9827239443	4653.40960087196
1	Deaths	1	Global	3	Both	22	All ages	543	Alzheimer's disease and other dementias	99	Smoking	1	Number	2019	63530.0557327683	175645.800949771	14646.5053591188
1	Deaths	1	Global	1	Male	22	All ages	543	Alzheimer's disease and other dementias	99	Smoking	3	Rate	2019	1.128307439	3.185668989	0.263684454
1	Deaths	1	Global	2	Fema	22	All ages	543	Alzheimer's disease and	99	Smok	3	Rate	2019	0.509755	1.334415	0.120630

					le				other dementias		ing				53	374	651
1	Deaths	1	Global	3	Both	22	All ages	543	Alzheimer's disease and other dementias	99	Smoking	3	Rate	2019	0.820237076	2.26776439	0.189101152
1	Deaths	1	Global	1	Male	22	All ages	543	Alzheimer's disease and other dementias	99	Smoking	1	Number	2020	44950.0343816157	127769.317461194	10427.6115448127
1	Deaths	1	Global	2	Female	22	All ages	543	Alzheimer's disease and other dementias	99	Smoking	1	Number	2020	19936.3194353276	53036.9389859318	4757.4962941851
1	Deaths	1	Global	3	Both	22	All ages	543	Alzheimer's disease and other dementias	99	Smoking	1	Number	2020	64886.3538169433	182793.126819357	15050.2144057839
1	Deaths	1	Global	1	Male	22	All ages	543	Alzheimer's disease and other dementias	99	Smoking	3	Rate	2020	1.144955999	3.254508	0.265609505
1	Deaths	1	Global	2	Fem	22	All ag	543	Alzheimer's diseas	99	Smo	3	Rat	202	0.5116	1.3611	0.1220

					ale		es		e and other dementias		king		e	0	53988	62046	98362
1	Deaths	1	Global	3	Both	22	All ages	543	Alzheimer's disease and other dementias	99	Smoking	3	Rate	2020	0.829498044	2.33680169	0.19239983
1	Deaths	1	Global	1	Male	22	All ages	543	Alzheimer's disease and other dementias	99	Smoking	1	Number	2021	46755.4220333656	132714.143134631	11060.6308225498
1	Deaths	1	Global	2	Female	22	All ages	543	Alzheimer's disease and other dementias	99	Smoking	1	Number	2021	20420.39601	53997.881492263	4945.891684502889
1	Deaths	1	Global	3	Both	22	All ages	543	Alzheimer's disease and other dementias	99	Smoking	1	Number	2021	67175.8180424356	184664.764137772	15694.6500054487
1	Deaths	1	Global	1	Male	22	All ages	543	Alzheimer's disease and other dementias	99	Smoking	3	Rate	2021	1.180873949	3.351882358	0.279351789
1	Deaths	1	Glob	2	Fe	22	Al	54	Alzheimer's	99	Sm	3	Ra	20	0.51	1.37	0.12

			al		male		ages	3	disease and other dementias		oking		te	21	9343695	3306339	5786868
1	Deaths	1	Global	3	Both	22	All ages	543	Alzheimer's disease and other dementias	99	Smoking	3	Rate	2021	0.851258529	2.340089933	0.198884138
1	Deaths	1	Global	1	Male	27	Age-standardized	543	Alzheimer's disease and other dementias	99	Smoking	3	Rate	1990	1.782423405	5.135650727	0.39985816
1	Deaths	1	Global	2	Female	27	Age-standardized	543	Alzheimer's disease and other dementias	99	Smoking	3	Rate	1990	0.667257549	1.830956628	0.160620165
1	Deaths	1	Global	3	Both	27	Age-standardized	543	Alzheimer's disease and other dementias	99	Smoking	3	Rate	1990	1.083533298	3.027386815	0.251079326
1	Deaths	1	Global	1	Male	27	Age-standardized	543	Alzheimer's disease and other dementias	99	Smoking	3	Rate	1991	1.770131883	5.177277864	0.395647906

1	Deaths	1	Global	2	Female	27	Age-standardized	543	Alzheimer’s disease and other dementias	99	Smoking	3	Rate	1991	0.666874328	1.830572342	0.160368787
1	Deaths	1	Global	3	Both	27	Age-standardized	543	Alzheimer’s disease and other dementias	99	Smoking	3	Rate	1991	1.079615584	3.048572244	0.249418154
1	Deaths	1	Global	1	Male	27	Age-standardized	543	Alzheimer’s disease and other dementias	99	Smoking	3	Rate	1992	1.755341829	5.120970192	0.394318701
1	Deaths	1	Global	2	Female	27	Age-standardized	543	Alzheimer’s disease and other dementias	99	Smoking	3	Rate	1992	0.666011013	1.840967192	0.158654591
1	Deaths	1	Global	3	Both	27	Age-standardized	543	Alzheimer’s disease and other dementias	99	Smoking	3	Rate	1992	1.074702119	3.038759874	0.249701343
1	Deaths	1	Global	1	Male	27	Age-stan	543	Alzheimer’s disease and	99	Smok	3	Rate	1993	1.742342	5.054249	0.390997

							dardized		other dementias		ing				212	079	418
1	Deaths	1	Global	2	Female	27	Age-standardized	543	Alzheimer's disease and other dementias	99	Smoking	3	Rate	1993	0.665499518	1.835600199	0.158462371
1	Deaths	1	Global	3	Both	27	Age-standardized	543	Alzheimer's disease and other dementias	99	Smoking	3	Rate	1993	1.070739681	2.999883434	0.246243162
1	Deaths	1	Global	1	Male	27	Age-standardized	543	Alzheimer's disease and other dementias	99	Smoking	3	Rate	1994	1.726313958	4.999929826	0.391940217
1	Deaths	1	Global	2	Female	27	Age-standardized	543	Alzheimer's disease and other dementias	99	Smoking	3	Rate	1994	0.663312763	1.834299954	0.155870596
1	Deaths	1	Global	3	Both	27	Age-standardized	543	Alzheimer's disease and other dementias	99	Smoking	3	Rate	1994	1.064386223	3.006465059	0.246880823

1	Deaths	1	Global	1	Male	27	Age-standardized	543	Alzheimer's disease and other dementias	99	Smoking	3	Rate	1995	1.711357815	4.977253803	0.382971032
1	Deaths	1	Global	2	Female	27	Age-standardized	543	Alzheimer's disease and other dementias	99	Smoking	3	Rate	1995	0.66148342	1.837334147	0.155143091
1	Deaths	1	Global	3	Both	27	Age-standardized	543	Alzheimer's disease and other dementias	99	Smoking	3	Rate	1995	1.058853307	3.006303304	0.242912914
1	Deaths	1	Global	1	Male	27	Age-standardized	543	Alzheimer's disease and other dementias	99	Smoking	3	Rate	1996	1.694236795	4.900065922	0.38279621
1	Deaths	1	Global	2	Female	27	Age-standardized	543	Alzheimer's disease and other dementias	99	Smoking	3	Rate	1996	0.656694605	1.810169929	0.154565836
1	Deaths	1	Global	3	Both	27	Age-stan	543	Alzheimer's disease and	99	Smok	3	Rate	1996	1.050465	2.972142	0.242736

							dardized		other dementias		ing				817	162	945
1	Deaths	1	Global	1	Male	27	Age-standardized	543	Alzheimer's disease and other dementias	99	Smoking	3	Rate	1997	1.672926798	4.903889099	0.380913943
1	Deaths	1	Global	2	Female	27	Age-standardized	543	Alzheimer's disease and other dementias	99	Smoking	3	Rate	1997	0.648421997	1.799166173	0.15503166
1	Deaths	1	Global	3	Both	27	Age-standardized	543	Alzheimer's disease and other dementias	99	Smoking	3	Rate	1997	1.03810264	2.95347488	0.240991264
1	Deaths	1	Global	1	Male	27	Age-standardized	543	Alzheimer's disease and other dementias	99	Smoking	3	Rate	1998	1.650867178	4.81770047	0.375679324
1	Deaths	1	Global	2	Female	27	Age-standardized	543	Alzheimer's disease and other dementias	99	Smoking	3	Rate	1998	0.639171845	1.773463557	0.149984653

1	Deaths	1	Global	3	Both	27	Age-standardized	543	Alzheimer’s disease and other dementias	99	Smoking	3	Rate	1998	1.024677081	2.925432023	0.23755091
1	Deaths	1	Global	1	Male	27	Age-standardized	543	Alzheimer’s disease and other dementias	99	Smoking	3	Rate	1999	1.627511156	4.769530785	0.367386302
1	Deaths	1	Global	2	Female	27	Age-standardized	543	Alzheimer’s disease and other dementias	99	Smoking	3	Rate	1999	0.629206059	1.75503225	0.148411766
1	Deaths	1	Global	3	Both	27	Age-standardized	543	Alzheimer’s disease and other dementias	99	Smoking	3	Rate	1999	1.010432273	2.876350201	0.23325661
1	Deaths	1	Global	1	Male	27	Age-standardized	543	Alzheimer’s disease and other dementias	99	Smoking	3	Rate	2000	1.608402407	4.690815293	0.363769509
1	Deaths	1	Global	2	Fema	27	Age-stan	543	Alzheimer’s disease and	99	Smok	3	Rate	2000	0.618968	1.723999	0.149717

					le		dardized		other dementias		ing				795	214	752
1	Deaths	1	Global	3	Both	27	Age-standardized	543	Alzheimer's disease and other dementias	99	Smoking	3	Rate	2000	0.9979084495	2.838571377	0.23210568
1	Deaths	1	Global	1	Male	27	Age-standardized	543	Alzheimer's disease and other dementias	99	Smoking	3	Rate	2001	1.590183973	4.640145031	0.360124017
1	Deaths	1	Global	2	Female	27	Age-standardized	543	Alzheimer's disease and other dementias	99	Smoking	3	Rate	2001	0.608779784	1.684472949	0.144721476
1	Deaths	1	Global	3	Both	27	Age-standardized	543	Alzheimer's disease and other dementias	99	Smoking	3	Rate	2001	0.985617104	2.798984735	0.227056816
1	Deaths	1	Global	1	Male	27	Age-standardized	543	Alzheimer's disease and other dementias	99	Smoking	3	Rate	2002	1.575607326	4.603692283	0.360274894

1	Deaths	1	Global	2	Female	27	Age-standardized	543	Alzheimer's disease and other dementias	99	Smoking	3	Rate	2002	0.598456261	1.654104498	0.143220186
1	Deaths	1	Global	3	Both	27	Age-standardized	543	Alzheimer's disease and other dementias	99	Smoking	3	Rate	2002	0.974899854	2.766813073	0.225659352
1	Deaths	1	Global	1	Male	27	Age-standardized	543	Alzheimer's disease and other dementias	99	Smoking	3	Rate	2003	1.560269358	4.572040171	0.355840448
1	Deaths	1	Global	2	Female	27	Age-standardized	543	Alzheimer's disease and other dementias	99	Smoking	3	Rate	2003	0.587669926	1.631242016	0.142459169
1	Deaths	1	Global	3	Both	27	Age-standardized	543	Alzheimer's disease and other dementias	99	Smoking	3	Rate	2003	0.963268671	2.736824028	0.222688402
1	Deaths	1	Global	1	Male	27	Age-stan	543	Alzheimer's disease and	99	Smok	3	Rate	2004	1.541720	4.486133	0.347764

							dardized		other dementias		ing				924	749	687
1	Deaths	1	Global	2	Female	27	Age-standardized	543	Alzheimer’s disease and other dementias	99	Smoking	3	Rate	2004	0.5747556624	1.5941816659	0.1387225669
1	Deaths	1	Global	3	Both	27	Age-standardized	543	Alzheimer’s disease and other dementias	99	Smoking	3	Rate	2004	0.9491264955	2.6806509447	0.2193400771
1	Deaths	1	Global	1	Male	27	Age-standardized	543	Alzheimer’s disease and other dementias	99	Smoking	3	Rate	2005	1.5252092966	4.4442439557	0.3452581008
1	Deaths	1	Global	2	Female	27	Age-standardized	543	Alzheimer’s disease and other dementias	99	Smoking	3	Rate	2005	0.5651951422	1.5717800078	0.1361210447
1	Deaths	1	Global	3	Both	27	Age-standardized	543	Alzheimer’s disease and other dementias	99	Smoking	3	Rate	2005	0.9381563774	2.6612823447	0.2148915557

1	Deaths	1	Global	1	Male	27	Age-standardized	543	Alzheimer’s disease and other dementias	99	Smoking	3	Rate	2006	1.508114689	4.330824934	0.347593835
1	Deaths	1	Global	2	Female	27	Age-standardized	543	Alzheimer’s disease and other dementias	99	Smoking	3	Rate	2006	0.553893038	1.546746961	0.135159396
1	Deaths	1	Global	3	Both	27	Age-standardized	543	Alzheimer’s disease and other dementias	99	Smoking	3	Rate	2006	0.92595149	2.607577006	0.214681647
1	Deaths	1	Global	1	Male	27	Age-standardized	543	Alzheimer’s disease and other dementias	99	Smoking	3	Rate	2007	1.497711334	4.354471793	0.339119687
1	Deaths	1	Global	2	Female	27	Age-standardized	543	Alzheimer’s disease and other dementias	99	Smoking	3	Rate	2007	0.543197879	1.507747293	0.132447622
1	Deaths	1	Global	3	Both	27	Age-stan	543	Alzheimer’s disease and	99	Smok	3	Rate	2007	0.916959	2.588812	0.210634

							dardized		other dementias		ing				279	109	366
1	Deaths	1	Global	1	Male	27	Age-standardized	543	Alzheimer’s disease and other dementias	99	Smoking	3	Rate	2008	1.488672017	4.351015697	0.338041032
1	Deaths	1	Global	2	Female	27	Age-standardized	543	Alzheimer’s disease and other dementias	99	Smoking	3	Rate	2008	0.533432378	1.472379323	0.130012358
1	Deaths	1	Global	3	Both	27	Age-standardized	543	Alzheimer’s disease and other dementias	99	Smoking	3	Rate	2008	0.909112433	2.570508775	0.210263676
1	Deaths	1	Global	1	Male	27	Age-standardized	543	Alzheimer’s disease and other dementias	99	Smoking	3	Rate	2009	1.480252869	4.268168296	0.333233137
1	Deaths	1	Global	2	Female	27	Age-standardized	543	Alzheimer’s disease and other dementias	99	Smoking	3	Rate	2009	0.522648453	1.450878325	0.127813667

1	Deaths	1	Global	3	Both	27	Age-standardized	543	Alzheimer's disease and other dementias	99	Smoking	3	Rate	2009	0.900854119	2.539275577	0.205584952
1	Deaths	1	Global	1	Male	27	Age-standardized	543	Alzheimer's disease and other dementias	99	Smoking	3	Rate	2010	1.473071687	4.234625208	0.343642246
1	Deaths	1	Global	2	Female	27	Age-standardized	543	Alzheimer's disease and other dementias	99	Smoking	3	Rate	2010	0.513692599	1.405066813	0.126868521
1	Deaths	1	Global	3	Both	27	Age-standardized	543	Alzheimer's disease and other dementias	99	Smoking	3	Rate	2010	0.893892869	2.494978124	0.207348294
1	Deaths	1	Global	1	Male	27	Age-standardized	543	Alzheimer's disease and other dementias	99	Smoking	3	Rate	2011	1.464453251	4.210424255	0.340806946
1	Deaths	1	Global	2	Fema	27	Agestan	543	Alzheimer's disease and	99	Smok	3	Rate	2011	0.504243	1.387407	0.123942

					le		dardized		other dementias		ing				04	491	864
1	Deaths	1	Global	3	Both	27	Age-standardized	543	Alzheimer's disease and other dementias	99	Smoking	3	Rate	2011	0.886016827	2.47368696	0.204362118
1	Deaths	1	Global	1	Male	27	Age-standardized	543	Alzheimer's disease and other dementias	99	Smoking	3	Rate	2012	1.45688816	4.224322399	0.333840376
1	Deaths	1	Global	2	Female	27	Age-standardized	543	Alzheimer's disease and other dementias	99	Smoking	3	Rate	2012	0.494370571	1.351006306	0.121232537
1	Deaths	1	Global	3	Both	27	Age-standardized	543	Alzheimer's disease and other dementias	99	Smoking	3	Rate	2012	0.878472989	2.471860919	0.201274406
1	Deaths	1	Global	1	Male	27	Age-standardized	543	Alzheimer's disease and other dementias	99	Smoking	3	Rate	2013	1.453419821	4.290807667	0.334705184

1	Deaths	1	Global	2	Female	27	Age-standardized	543	Alzheimer's disease and other dementias	99	Smoking	3	Rate	2013	0.484761558	1.322046611	0.118103022
1	Deaths	1	Global	3	Both	27	Age-standardized	543	Alzheimer's disease and other dementias	99	Smoking	3	Rate	2013	0.872351859	2.482452615	0.200139883
1	Deaths	1	Global	1	Male	27	Age-standardized	543	Alzheimer's disease and other dementias	99	Smoking	3	Rate	2014	1.450308419	4.189153113	0.33881329
1	Deaths	1	Global	2	Female	27	Age-standardized	543	Alzheimer's disease and other dementias	99	Smoking	3	Rate	2014	0.47610654	1.302841318	0.114933284
1	Deaths	1	Global	3	Both	27	Age-standardized	543	Alzheimer's disease and other dementias	99	Smoking	3	Rate	2014	0.866698338	2.455358791	0.202910863
1	Deaths	1	Global	1	Male	27	Age-stan	543	Alzheimer's disease and	99	Smok	3	Rate	2015	1.446567	4.252269	0.334251

							dardized		other dementias		ing				964	926	825
1	Deaths	1	Global	2	Female	27	Age-standardized	543	Alzheimer’s disease and other dementias	99	Smoking	3	Rate	2015	0.468734354	1.284761587	0.113054914
1	Deaths	1	Global	3	Both	27	Age-standardized	543	Alzheimer’s disease and other dementias	99	Smoking	3	Rate	2015	0.861642515	2.448921148	0.199919602
1	Deaths	1	Global	1	Male	27	Age-standardized	543	Alzheimer’s disease and other dementias	99	Smoking	3	Rate	2016	1.439549852	4.146822132	0.336202537
1	Deaths	1	Global	2	Female	27	Age-standardized	543	Alzheimer’s disease and other dementias	99	Smoking	3	Rate	2016	0.461748174	1.254628724	0.109292946
1	Deaths	1	Global	3	Both	27	Age-standardized	543	Alzheimer’s disease and other dementias	99	Smoking	3	Rate	2016	0.855629198	2.414716069	0.197071615

1	Deaths	1	Global	1	Male	27	Age-standardized	543	Alzheimer's disease and other dementias	99	Smoking	3	Rate	2017	1.433526072	4.206945343	0.341401766
1	Deaths	1	Global	2	Female	27	Age-standardized	543	Alzheimer's disease and other dementias	99	Smoking	3	Rate	2017	0.454130626	1.233746872	0.107083938
1	Deaths	1	Global	3	Both	27	Age-standardized	543	Alzheimer's disease and other dementias	99	Smoking	3	Rate	2017	0.849930806	2.427685803	0.200409185
1	Deaths	1	Global	1	Male	27	Age-standardized	543	Alzheimer's disease and other dementias	99	Smoking	3	Rate	2018	1.423525279	4.065570948	0.32505436
1	Deaths	1	Global	2	Female	27	Age-standardized	543	Alzheimer's disease and other dementias	99	Smoking	3	Rate	2018	0.446707764	1.206481094	0.10917533
1	Deaths	1	Global	3	Both	27	Age-stan	543	Alzheimer's disease and	99	Smok	3	Rate	2018	0.842675	2.354585	0.195366

							dardized		other dementias		ing				858	438	338
1	Deaths	1	Global	1	Male	27	Age-standardized	543	Alzheimer’s disease and other dementias	99	Smoking	3	Rate	2019	1.418254477	3.954626828	0.326982988
1	Deaths	1	Global	2	Female	27	Age-standardized	543	Alzheimer’s disease and other dementias	99	Smoking	3	Rate	2019	0.439107238	1.151413622	0.104025527
1	Deaths	1	Global	3	Both	27	Age-standardized	543	Alzheimer’s disease and other dementias	99	Smoking	3	Rate	2019	0.837225899	2.283632656	0.19261331
1	Deaths	1	Global	1	Male	27	Age-standardized	543	Alzheimer’s disease and other dementias	99	Smoking	3	Rate	2020	1.410064005	3.949805363	0.3303434
1	Deaths	1	Global	2	Female	27	Age-standardized	543	Alzheimer’s disease and other dementias	99	Smoking	3	Rate	2020	0.431826008	1.148992948	0.10285162

1	Deaths	1	Global	3	Both	27	Age-standardized	543	Alzheimer's disease and other dementias	99	Smoking	3	Rate	2020	0.829820529	2.323420956	0.19277174
1	Deaths	1	Global	1	Male	27	Age-standardized	543	Alzheimer's disease and other dementias	99	Smoking	3	Rate	2021	1.431846835	4.046686865	0.335077838
1	Deaths	1	Global	2	Female	27	Age-standardized	543	Alzheimer's disease and other dementias	99	Smoking	3	Rate	2021	0.430887013	1.139897582	0.104437942
1	Deaths	1	Global	3	Both	27	Age-standardized	543	Alzheimer's disease and other dementias	99	Smoking	3	Rate	2021	0.837652906	2.29298703	0.19474573 7
2	DALYs (Disability-Adjusted Life Years)	1	Global	1	Male	22	All ages	543	Alzheimer's disease and other dementias	99	Smoking	1	Number	1990	532605.577089624	1241825.380682 12	227758.662234778
2	DALYs (Disability-Adjust	1	Global	2	Fema	22	All ages	543	Alzheimer's disease and	99	Smok	1	Numb	1990	262309.6	602150.5	112857.2

	ed Life Years)				le				other dementias		ing		er		23694243	55403158	9978498
2	DALYs (Disability-Adjusted Life Years)	1	Global	3	Both	22	All ages	543	Alzheimer's disease and other dementias	99	Smoking	1	Number	1990	794915.200783867	1839709.41166161	344377.508851189
2	DALYs (Disability-Adjusted Life Years)	1	Global	1	Male	22	All ages	543	Alzheimer's disease and other dementias	99	Smoking	3	Rate	1990	19.83084662	46.23768454	8.480285022
2	DALYs (Disability-Adjusted Life Years)	1	Global	2	Female	22	All ages	543	Alzheimer's disease and other dementias	99	Smoking	3	Rate	1990	9.906399933	22.74085159	4.262175102
2	DALYs (Disability-Adjusted Life Years)	1	Global	3	Both	22	All ages	543	Alzheimer's disease and other dementias	99	Smoking	3	Rate	1990	14.90384936	34.49267543	6.456727095
2	DALYs (Disability-Adjusted Life Years)	1	Global	1	Male	22	All ages	543	Alzheimer's disease and other dementias	99	Smoking	1	Number	1991	544660.261221368	1248835.01928013	229266.582477055
2	DALYs (Disability-	1	Global	2	Fem	22	All ag	543	Alzheimer's diseas	99	Smo	1	Num	199	268922	610301	115959

	Adjusted Life Years)				ale		es		e and other dementias		king		ber	1	.280701405	.347765936	.823318059
2	DALYs (Disability-Adjusted Life Years)	1	Global	3	Both	22	All ages	543	Alzheimer's disease and other dementias	99	Smoking	1	Number	1991	813582.541922773	1859921.77514483	350102.098361677
2	DALYs (Disability-Adjusted Life Years)	1	Global	1	Male	22	All ages	543	Alzheimer's disease and other dementias	99	Smoking	3	Rate	1991	19.96137925	45.7688842	8.402443739
2	DALYs (Disability-Adjusted Life Years)	1	Global	2	Female	22	All ages	543	Alzheimer's disease and other dementias	99	Smoking	3	Rate	1991	10.00475887	22.70513922	4.314071961
2	DALYs (Disability-Adjusted Life Years)	1	Global	3	Both	22	All ages	543	Alzheimer's disease and other dementias	99	Smoking	3	Rate	1991	15.02040893	34.33798565	6.463605615
2	DALYs (Disability-Adjusted Life Years)	1	Global	1	Male	22	All ages	543	Alzheimer's disease and other dementias	99	Smoking	1	Number	1992	555652.476400528	1268305.52140793	235825.133231695
2	DALYs (Disab	1	Glob	2	Fe	22	Al l	54	Alzheimer's	99	Sm	1	Nu	19	2751	6283	1190

	ility-Adjusted Life Years)		al		male		ages	3	disease and other dementias		oking		mber	92	46.481344864	77.810004068	12.415744855
2	DALYs (Disability-Adjusted Life Years)	1	Global	3	Both	22	All ages	543	Alzheimer's disease and other dementias	99	Smoking	1	Number	1992	830798.957745391	1894160.61389953	361845.380549833
2	DALYs (Disability-Adjusted Life Years)	1	Global	1	Male	22	All ages	543	Alzheimer's disease and other dementias	99	Smoking	3	Rate	1992	20.0586313	45.78486358	8.513107742
2	DALYs (Disability-Adjusted Life Years)	1	Global	2	Female	22	All ages	543	Alzheimer's disease and other dementias	99	Smoking	3	Rate	1992	10.08946864	23.04226525	4.364119178
2	DALYs (Disability-Adjusted Life Years)	1	Global	3	Both	22	All ages	543	Alzheimer's disease and other dementias	99	Smoking	3	Rate	1992	15.1131085	34.45677755	6.58234877
2	DALYs (Disability-Adjusted Life Years)	1	Global	1	Male	22	All ages	543	Alzheimer's disease and other dementias	99	Smoking	1	Number	1993	566636.754630084	1302331.45722256	240924.182986763
2	DALYs	1	Gl	2	F	2	Al	5	Alzhei	9	S	1	N	1	28	63	12

	(Disability-Adjusted Life Years)		obal		emale	2	1 ages	43	mer's disease and other dementias	9	moking		umber	993	1362.83872895284	5832.76910313519	0173.27318942237
2	DALYs (Disability-Adjusted Life Years)	1	Global	3	Both	22	All ages	543	Alzheimer's disease and other dementias	99	Smoking	1	Number	1993	7999.593359036	35360.71490659	0492.226391127
2	DALYs (Disability-Adjusted Life Years)	1	Global	1	Male	22	All ages	543	Alzheimer's disease and other dementias	99	Smoking	3	Rate	1993	20.16350784	46.34286485	8.573176046
2	DALYs (Disability-Adjusted Life Years)	1	Global	2	Female	22	All ages	543	Alzheimer's disease and other dementias	99	Smoking	3	Rate	1993	10.17491806	22.99360624	4.345823397
2	DALYs (Disability-Adjusted Life Years)	1	Global	3	Both	22	All ages	543	Alzheimer's disease and other dementias	99	Smoking	3	Rate	1993	15.20947765	34.71207505	6.645042379
2	DALYs (Disability-Adjusted Life Years)	1	Global	1	Male	22	All ages	543	Alzheimer's disease and other dementias	99	Smoking	1	Number	1994	576194.558482047	1322550.39570814	245968.932945236

2	DALYs (Disability-Adjusted Life Years)	1	Global	2	Female	22	All ages	543	Alzheimer's disease and other dementias	99	Smoking	1	Number	1994	287023.610017063	649280.889273037	123025.771887275
2	DALYs (Disability-Adjusted Life Years)	1	Global	3	Both	22	All ages	543	Alzheimer's disease and other dementias	99	Smoking	1	Number	1994	863218.16849911	1973778.9803855	376655.569132528
2	DALYs (Disability-Adjusted Life Years)	1	Global	1	Male	22	All ages	543	Alzheimer's disease and other dementias	99	Smoking	3	Rate	1994	20.22971978	46.43366291	8.6357681
2	DALYs (Disability-Adjusted Life Years)	1	Global	2	Female	22	All ages	543	Alzheimer's disease and other dementias	99	Smoking	3	Rate	1994	10.24279025	23.17038644	4.390325858
2	DALYs (Disability-Adjusted Life Years)	1	Global	3	Both	22	All ages	543	Alzheimer's disease and other dementias	99	Smoking	3	Rate	1994	15.27695627	34.93130273	6.665928578
2	DALYs (Disability-Adjusted Life Years)	1	Global	1	Male	22	All ages	543	Alzheimer's disease and other dementias	99	Smoking	1	Number	1995	585560.5129588	1374053.987033	249178.9503894

2	DALYs (Disability-Adjusted Life Years)	1	Global	2	Female	22	All ages	543	Alzheimer's disease and other dementias	99	Smoking	1	Number	1995	86292489.249333601	79670777.061897306	05124509.737127552
2	DALYs (Disability-Adjusted Life Years)	1	Global	3	Both	22	All ages	543	Alzheimer's disease and other dementias	99	Smoking	1	Number	1995	878049.762292486	2024378.66444585	382636.036634867
2	DALYs (Disability-Adjusted Life Years)	1	Global	1	Male	22	All ages	543	Alzheimer's disease and other dementias	99	Smoking	3	Rate	1995	20.2937833	47.6206186	8.635800246
2	DALYs (Disability-Adjusted Life Years)	1	Global	2	Female	22	All ages	543	Alzheimer's disease and other dementias	99	Smoking	3	Rate	1995	10.30384039	23.6302011	4.386241412
2	DALYs (Disability-Adjusted Life Years)	1	Global	3	Both	22	All ages	543	Alzheimer's disease and other dementias	99	Smoking	3	Rate	1995	15.33962909	35.36612523	6.684695027
2	DALYs (Disability-Adjusted Life	1	Global	1	Male	22	All ages	543	Alzheimer's disease and other dement	99	Smokin	1	Number	1996	594130.14929	1403506.6395	252742.20356

	Years)								ias		g				0987	305	4454
2	DALYs (Disability-Adjusted Life Years)	1	Global	2	Female	22	All ages	543	Alzheimer's disease and other dementias	99	Smoking	1	Number	1996	296942.218980834	680297.184	126334.837790599
2	DALYs (Disability-Adjusted Life Years)	1	Global	3	Both	22	All ages	543	Alzheimer's disease and other dementias	99	Smoking	1	Number	1996	891072.368271821	2069134.964	386240.731309941
2	DALYs (Disability-Adjusted Life Years)	1	Global	1	Male	22	All ages	543	Alzheimer's disease and other dementias	99	Smoking	3	Rate	1996	20.32753382	48.01949322	8.647306816
2	DALYs (Disability-Adjusted Life Years)	1	Global	2	Female	22	All ages	543	Alzheimer's disease and other dementias	99	Smoking	3	Rate	1996	10.32623724	23.65749855	4.393324435
2	DALYs (Disability-Adjusted Life Years)	1	Global	3	Both	22	All ages	543	Alzheimer's disease and other dementias	99	Smoking	3	Rate	1996	15.3675711	35.6846198	6.661167052
2	DALYs (Disability-Adjusted Life Years)	1	Global	1	Male	22	All ages	543	Alzheimer's disease and other	99	Smoki	1	Numbe	1997	601069.038	1401827.09	258813.241

	Life Years)								dementias		ng		r		14726	788495	479621
2	DALYs (Disability-Adjusted Life Years)	1	Global	2	Female	22	All ages	543	Alzheimer's disease and other dementias	99	Smoking	1	Number	1997	300221.212304032	685473.658510306	128006.664083009
2	DALYs (Disability-Adjusted Life Years)	1	Global	3	Both	22	All ages	543	Alzheimer's disease and other dementias	99	Smoking	1	Number	1997	901290.250451291	2074537.74753608	390595.450046286
2	DALYs (Disability-Adjusted Life Years)	1	Global	1	Male	22	All ages	543	Alzheimer's disease and other dementias	99	Smoking	3	Rate	1997	20.30572333	47.3574771	8.74340507
2	DALYs (Disability-Adjusted Life Years)	1	Global	2	Female	22	All ages	543	Alzheimer's disease and other dementias	99	Smoking	3	Rate	1997	10.30632694	23.53170044	4.394354817
2	DALYs (Disability-Adjusted Life Years)	1	Global	3	Both	22	All ages	543	Alzheimer's disease and other dementias	99	Smoking	3	Rate	1997	15.34613538	35.32284646	6.65061078
2	DALYs (Disability-Adjust	1	Global	1	Male	22	All ages	543	Alzheimer's disease and	99	Smok	1	Numb	1998	607622.2	1401787.	261860.8

	ed Life Years)								other dementias		ing		er		38530401	74752058	27638754
2	DALYs (Disability-Adjusted Life Years)	1	Global	2	Female	22	All ages	543	Alzheimer's disease and other dementias	99	Smoking	1	Number	1998	303172.19633803	693812.50441292	130155.06994502
2	DALYs (Disability-Adjusted Life Years)	1	Global	3	Both	22	All ages	543	Alzheimer's disease and other dementias	99	Smoking	1	Number	1998	910794.34864204	2090695.11869448	395411.852991738
2	DALYs (Disability-Adjusted Life Years)	1	Global	1	Male	22	All ages	543	Alzheimer's disease and other dementias	99	Smoking	3	Rate	1998	20.27196841	46.76753933	8.736405762
2	DALYs (Disability-Adjusted Life Years)	1	Global	2	Female	22	All ages	543	Alzheimer's disease and other dementias	99	Smoking	3	Rate	1998	10.27517676	23.51484143	4.411243434
2	DALYs (Disability-Adjusted Life Years)	1	Global	3	Both	22	All ages	543	Alzheimer's disease and other dementias	99	Smoking	3	Rate	1998	15.31291994	35.1502444	6.647943617
2	DALYs (Disability-	1	Global	1	Mal	22	All ag	543	Alzheimer's diseas	99	Smo	1	Num	199	615127	144385	266633

	Adjusted Life Years)				e		es		e and other dementias		king		ber	9	.45936076	0.43184702	.41408682
2	DALYs (Disability-Adjusted Life Years)	1	Global	2	Female	22	All ages	543	Alzheimer's disease and other dementias	99	Smoking	1	Number	1999	306080.646819262	699095.462584378	132533.954572784
2	DALYs (Disability-Adjusted Life Years)	1	Global	3	Both	22	All ages	543	Alzheimer's disease and other dementias	99	Smoking	1	Number	1999	921208.106180023	2122179.62378349	397474.84560184
2	DALYs (Disability-Adjusted Life Years)	1	Global	1	Male	22	All ages	543	Alzheimer's disease and other dementias	99	Smoking	3	Rate	1999	20.27050573	47.57969752	8.786462164
2	DALYs (Disability-Adjusted Life Years)	1	Global	2	Female	22	All ages	543	Alzheimer's disease and other dementias	99	Smoking	3	Rate	1999	10.24266743	23.39449553	4.435109616
2	DALYs (Disability-Adjusted Life Years)	1	Global	3	Both	22	All ages	543	Alzheimer's disease and other dementias	99	Smoking	3	Rate	1999	15.29513295	35.2352734	6.599410671
2	DALYs (Disab	1	Glob	1	Ma	22	All	54	Alzheimer's	99	Sm	1	Nu	20	6253	1444	2703

	ility-Adjusted Life Years)		al		le		ages	3	disease and other dementias		oking		mber	00	02.790503328	123.08040977	58.23350832
2	DALYs (Disability-Adjusted Life Years)	1	Global	2	Female	22	All ages	543	Alzheimer's disease and other dementias	99	Smoking	1	Number	2000	309321.97276668	704873.43794164	134303.554185751
2	DALYs (Disability-Adjusted Life Years)	1	Global	3	Both	22	All ages	543	Alzheimer's disease and other dementias	99	Smoking	1	Number	2000	934624.763269996	2147266.38990309	402712.984171521
2	DALYs (Disability-Adjusted Life Years)	1	Global	1	Male	22	All ages	543	Alzheimer's disease and other dementias	99	Smoking	3	Rate	2000	20.353656606	47.006322277	8.800182217
2	DALYs (Disability-Adjusted Life Years)	1	Global	2	Female	22	All ages	543	Alzheimer's disease and other dementias	99	Smoking	3	Rate	2000	10.220151119	23.289367522	4.437455952
2	DALYs (Disability-Adjusted Life Years)	1	Global	3	Both	22	All ages	543	Alzheimer's disease and other dementias	99	Smoking	3	Rate	2000	15.324787729	35.208141225	6.603174946
2	DALYs	1	Gl	1	M	2	Al	5	Alzhei	9	S	1	N	2	63	14	27

	(Disability-Adjusted Life Years)		obal		ale	2	l ages	43	mer's disease and other dementias	9	moking		umber	001	6793.06785350731	69826.9687170871	5647.95159884313
2	DALYs (Disability-Adjusted Life Years)	1	Global	2	Female	22	All ages	543	Alzheimer's disease and other dementias	99	Smoking	1	Number	2001	2761.88857740894	2841.20713286221	5188.51225929441
2	DALYs (Disability-Adjusted Life Years)	1	Global	3	Both	22	All ages	543	Alzheimer's disease and other dementias	99	Smoking	1	Number	2001	9554.956430915	67433.4724299	0505.486360519
2	DALYs (Disability-Adjusted Life Years)	1	Global	1	Male	22	All ages	543	Alzheimer's disease and other dementias	99	Smoking	3	Rate	2001	20.47248489	47.25398552	8.861903198
2	DALYs (Disability-Adjusted Life Years)	1	Global	2	Female	22	All ages	543	Alzheimer's disease and other dementias	99	Smoking	3	Rate	2001	10.20190532	23.25199704	4.409681785
2	DALYs (Disability-Adjusted Life Years)	1	Global	3	Both	22	All ages	543	Alzheimer's disease and other dementias	99	Smoking	3	Rate	2001	15.37441324	35.09330098	6.646567367

2	DALYs (Disability-Adjusted Life Years)	1	Global	1	Male	22	All ages	543	Alzheimer's disease and other dementias	99	Smoking	1	Number	2002	650325.335673657	1523005.68903111	280808.629855832
2	DALYs (Disability-Adjusted Life Years)	1	Global	2	Female	22	All ages	543	Alzheimer's disease and other dementias	99	Smoking	1	Number	2002	315996.99201586	716880.878229248	137034.831481603
2	DALYs (Disability-Adjusted Life Years)	1	Global	3	Both	22	All ages	543	Alzheimer's disease and other dementias	99	Smoking	1	Number	2002	966322.327689517	2227886.6625395	414902.986601115
2	DALYs (Disability-Adjusted Life Years)	1	Global	1	Male	22	All ages	543	Alzheimer's disease and other dementias	99	Smoking	3	Rate	2002	20.64775512	48.35525665	8.91564192
2	DALYs (Disability-Adjusted Life Years)	1	Global	2	Female	22	All ages	543	Alzheimer's disease and other dementias	99	Smoking	3	Rate	2002	10.17454191	23.08229105	4.412278191
2	DALYs (Disability-Adjusted Life	1	Global	3	Both	22	All ages	543	Alzheimer's disease and other dement	99	Smokin	3	Rate	2002	15.4478622	35.61553445	6.632739386

	Years)								ias		g						
2	DALYs (Disability-Adjusted Life Years)	1	Global	1	Male	22	All ages	543	Alzheimer's disease and other dementias	99	Smoking	1	Number	2003	663088.25341838	1530262.28631125	287394.102254083
2	DALYs (Disability-Adjusted Life Years)	1	Global	2	Female	22	All ages	543	Alzheimer's disease and other dementias	99	Smoking	1	Number	2003	319073.327186428	720545.589912902	138365.539757814
2	DALYs (Disability-Adjusted Life Years)	1	Global	3	Both	22	All ages	543	Alzheimer's disease and other dementias	99	Smoking	1	Number	2003	982161.580604808	2244376.70024324	422393.611151187
2	DALYs (Disability-Adjusted Life Years)	1	Global	1	Male	22	All ages	543	Alzheimer's disease and other dementias	99	Smoking	3	Rate	2003	20.78998375	47.97872365	9.010744324
2	DALYs (Disability-Adjusted Life Years)	1	Global	2	Female	22	All ages	543	Alzheimer's disease and other dementias	99	Smoking	3	Rate	2003	10.1400667	22.89874998	4.397220586
2	DALYs (Disability-Adjusted	1	Global	3	Both	22	All ages	543	Alzheimer's disease and other	99	Smoki	3	Rate	2003	15.5009958	35.4219454	6.66644037

	Life Years)								dementias		ng				6	6	7
2	DALYs (Disability-Adjusted Life Years)	1	Global	1	Male	22	All ages	543	Alzheimer's disease and other dementias	99	Smoking	1	Number	2004	676552.866836629	1554893.19141616	293612.278348751
2	DALYs (Disability-Adjusted Life Years)	1	Global	2	Female	22	All ages	543	Alzheimer's disease and other dementias	99	Smoking	1	Number	2004	321856.566202439	726615.319732558	138818.598968915
2	DALYs (Disability-Adjusted Life Years)	1	Global	3	Both	22	All ages	543	Alzheimer's disease and other dementias	99	Smoking	1	Number	2004	998409.433038729	2275836.73629314	428183.757262774
2	DALYs (Disability-Adjusted Life Years)	1	Global	1	Male	22	All ages	543	Alzheimer's disease and other dementias	99	Smoking	3	Rate	2004	20.94609986	48.1395463	9.090246164
2	DALYs (Disability-Adjusted Life Years)	1	Global	2	Female	22	All ages	543	Alzheimer's disease and other dementias	99	Smoking	3	Rate	2004	10.09460679	22.78933137	4.353862307
2	DALYs (Disability-Adjust	1	Global	3	Both	22	All ages	543	Alzheimer's disease and	99	Smok	3	Rate	2004	15.55549	35.45816	6.671220

	ed Life Years)								other dementias		ing				384	292	822
2	DALYs (Disability-Adjusted Life Years)	1	Global	1	Male	22	All ages	543	Alzheimer's disease and other dementias	99	Smoking	1	Number	2005	691516.418799881	1590779.40739332	299521.409086716
2	DALYs (Disability-Adjusted Life Years)	1	Global	2	Female	22	All ages	543	Alzheimer's disease and other dementias	99	Smoking	1	Number	2005	326326.29993603	744326.949836936	140754.362071973
2	DALYs (Disability-Adjusted Life Years)	1	Global	3	Both	22	All ages	543	Alzheimer's disease and other dementias	99	Smoking	1	Number	2005	1017842.71873591	2320600.87501322	437510.066929733
2	DALYs (Disability-Adjusted Life Years)	1	Global	1	Male	22	All ages	543	Alzheimer's disease and other dementias	99	Smoking	3	Rate	2005	21.13904824	48.62872625	9.156105832
2	DALYs (Disability-Adjusted Life Years)	1	Global	2	Female	22	All ages	543	Alzheimer's disease and other dementias	99	Smoking	3	Rate	2005	10.09962706	23.03652695	4.356273349
2	DALYs (Disability-	1	Global	3	Bot	22	All ag	543	Alzheimer's diseas	99	Smo	3	Rat	200	15.653	35.688	6.7284

	Adjusted Life Years)				h		es		e and other dementias		king		e	5	46457	66085	93712
2	DALYs (Disability-Adjusted Life Years)	1	Global	1	Male	22	All ages	543	Alzheimer's disease and other dementias	99	Smoking	1	Number	2006	708243.554512831	1635019.11553098	307165.11350649
2	DALYs (Disability-Adjusted Life Years)	1	Global	2	Female	22	All ages	543	Alzheimer's disease and other dementias	99	Smoking	1	Number	2006	330537.650520387	751449.333653719	142750.231771941
2	DALYs (Disability-Adjusted Life Years)	1	Global	3	Both	22	All ages	543	Alzheimer's disease and other dementias	99	Smoking	1	Number	2006	1038781.20503322	2379762.12902479	447497.161326945
2	DALYs (Disability-Adjusted Life Years)	1	Global	1	Male	22	All ages	543	Alzheimer's disease and other dementias	99	Smoking	3	Rate	2006	21.37230508	49.33913923	9.26916521
2	DALYs (Disability-Adjusted Life Years)	1	Global	2	Female	22	All ages	543	Alzheimer's disease and other dementias	99	Smoking	3	Rate	2006	10.0931171	22.94584628	4.358943083
2	DALYs (Disab	1	Glob	3	Bo	22	All	54	Alzheimer's	99	Sm	3	Ra	20	15.7	36.1	6.79

	ility-Adjusted Life Years)		al		th		ages	3	disease and other dementias		oking		te	06	6605551	1873382	1868253
2	DALYs (Disability-Adjusted Life Years)	1	Global	1	Male	22	All ages	543	Alzheimer's disease and other dementias	99	Smoking	1	Number	2007	729262.507609263	1694680.80769636	315622.156226834
2	DALYs (Disability-Adjusted Life Years)	1	Global	2	Female	22	All ages	543	Alzheimer's disease and other dementias	99	Smoking	1	Number	2007	335131.804480297	757633.01392516	144570.647155038
2	DALYs (Disability-Adjusted Life Years)	1	Global	3	Both	22	All ages	543	Alzheimer's disease and other dementias	99	Smoking	1	Number	2007	1064394.31208956	2434424.08308974	459648.497681863
2	DALYs (Disability-Adjusted Life Years)	1	Global	1	Male	22	All ages	543	Alzheimer's disease and other dementias	99	Smoking	3	Rate	2007	21.71947529	50.47232999	9.400109772
2	DALYs (Disability-Adjusted Life Years)	1	Global	2	Female	22	All ages	543	Alzheimer's disease and other dementias	99	Smoking	3	Rate	2007	10.09511671	22.82204672	4.354876309
2	DALYs	1	Gl	3	B	2	Al	5	Alzhei	9	S	3	R	2	15	36	6.

	(Disability-Adjusted Life Years)		obal		oth	2	l ages	43	mer's disease and other dementias	9	moking		ate	007	.94028655	.45774599	883660202
2	DALYs (Disability-Adjusted Life Years)	1	Global	1	Male	22	All ages	543	Alzheimer's disease and other dementias	99	Smoking	1	Number	2008	751291.72937657	1750104.35361314	326544.678103234
2	DALYs (Disability-Adjusted Life Years)	1	Global	2	Female	22	All ages	543	Alzheimer's disease and other dementias	99	Smoking	1	Number	2008	339989.521932161	775795.565289749	145693.753385623
2	DALYs (Disability-Adjusted Life Years)	1	Global	3	Both	22	All ages	543	Alzheimer's disease and other dementias	99	Smoking	1	Number	2008	1091281.25130873	2512988.44114822	471121.878246415
2	DALYs (Disability-Adjusted Life Years)	1	Global	1	Male	22	All ages	543	Alzheimer's disease and other dementias	99	Smoking	3	Rate	2008	22.07822746	51.43035719	9.596176022
2	DALYs (Disability-Adjusted Life Years)	1	Global	2	Female	22	All ages	543	Alzheimer's disease and other dementias	99	Smoking	3	Rate	2008	10.10166717	23.05020623	4.328809305

2	DALYs (Disability-Adjusted Life Years)	1	Global	3	Both	22	All ages	543	Alzheimer's disease and other dementias	99	Smoking	3	Rate	2008	16.1228459	37.12748235	6.960465449
2	DALYs (Disability-Adjusted Life Years)	1	Global	1	Male	22	All ages	543	Alzheimer's disease and other dementias	99	Smoking	1	Number	2009	775034.761653115	1790985.12983419	334798.55452951
2	DALYs (Disability-Adjusted Life Years)	1	Global	2	Female	22	All ages	543	Alzheimer's disease and other dementias	99	Smoking	1	Number	2009	344855.280498828	784961.828955907	148527.885718154
2	DALYs (Disability-Adjusted Life Years)	1	Global	3	Both	22	All ages	543	Alzheimer's disease and other dementias	99	Smoking	1	Number	2009	1119890.04215194	2571890.62560125	480417.468828717
2	DALYs (Disability-Adjusted Life Years)	1	Global	1	Male	22	All ages	543	Alzheimer's disease and other dementias	99	Smoking	3	Rate	2009	22.47318787	51.93205169	9.707939808
2	DALYs (Disability-Adjusted Life	1	Global	2	Female	22	All ages	543	Alzheimer's disease and other dement	99	Smokin	3	Rate	2009	10.10608336	23.00353258	4.352652488

	Years)								ias		g						
2	DALYs (Disability-Adjusted Life Years)	1	Global	3	Both	22	All ages	543	Alzheimer's disease and other dementias	99	Smoking	3	Rate	2009	16.32240083	37.48531383	7.002086095
2	DALYs (Disability-Adjusted Life Years)	1	Global	1	Male	22	All ages	543	Alzheimer's disease and other dementias	99	Smoking	1	Number	2010	798105.275484365	1860106.95013649	343618.674783699
2	DALYs (Disability-Adjusted Life Years)	1	Global	2	Female	22	All ages	543	Alzheimer's disease and other dementias	99	Smoking	1	Number	2010	350725.077055994	787230.436594007	151040.607279109
2	DALYs (Disability-Adjusted Life Years)	1	Global	3	Both	22	All ages	543	Alzheimer's disease and other dementias	99	Smoking	1	Number	2010	1148830.35254036	2639915.65616396	493341.072067183
2	DALYs (Disability-Adjusted Life Years)	1	Global	1	Male	22	All ages	543	Alzheimer's disease and other dementias	99	Smoking	3	Rate	2010	22.85076763	53.25722431	9.838239053
2	DALYs (Disability-Adjusted ed	1	Global	2	Femal	22	All ages	543	Alzheimer's disease and other	99	Smoki	3	Rate	2010	10.1422166	22.7650150	4.36777026

	Life Years)				e				dementias		ng				5	1	4
2	DALYs (Disability-Adjusted Life Years)	1	Global	3	Both	22	All ages	543	Alzheimer's disease and other dementias	99	Smoking	3	Rate	2010	16.52813528	37.98026663	7.097660644
2	DALYs (Disability-Adjusted Life Years)	1	Global	1	Male	22	All ages	543	Alzheimer's disease and other dementias	99	Smoking	1	Number	2011	822163.35756595	1892334.57633705	353281.501046912
2	DALYs (Disability-Adjusted Life Years)	1	Global	2	Female	22	All ages	543	Alzheimer's disease and other dementias	99	Smoking	1	Number	2011	356427.876507968	810025.904249206	153172.337756797
2	DALYs (Disability-Adjusted Life Years)	1	Global	3	Both	22	All ages	543	Alzheimer's disease and other dementias	99	Smoking	1	Number	2011	1178591.23407392	2704116.89019389	507175.587650877
2	DALYs (Disability-Adjusted Life Years)	1	Global	1	Male	22	All ages	543	Alzheimer's disease and other dementias	99	Smoking	3	Rate	2011	23.25642639	53.52821841	9.993227194
2	DALYs (Disability-Adjust	1	Global	2	Fema	22	All ages	543	Alzheimer's disease and	99	Smok	3	Rate	2011	10.17599	23.12618	4.373060

	ed Life Years)				le				other dementias		ing				24	621	154
2	DALYs (Disability-Adjusted Life Years)	1	Global	3	Both	22	All ages	543	Alzheimer's disease and other dementias	99	Smoking	3	Rate	2011	16.746480444	38.422516605	7.2064052 5
2	DALYs (Disability-Adjusted Life Years)	1	Global	1	Male	22	All ages	543	Alzheimer's disease and other dementias	99	Smoking	1	Number	2012	848168.053357998	2004530.67357884	367036.229604932
2	DALYs (Disability-Adjusted Life Years)	1	Global	2	Female	22	All ages	543	Alzheimer's disease and other dementias	99	Smoking	1	Number	2012	361989.470446773	819250.179909254	155757.138087831
2	DALYs (Disability-Adjusted Life Years)	1	Global	3	Both	22	All ages	543	Alzheimer's disease and other dementias	99	Smoking	1	Number	2012	1210157.52380477	2816668.82059349	522822.660461908
2	DALYs (Disability-Adjusted Life Years)	1	Global	1	Male	22	All ages	543	Alzheimer's disease and other dementias	99	Smoking	3	Rate	2012	23.700661222	56.013312271	10.256223375
2	DALYs (Disability-	1	Global	2	Fem	22	All ag	543	Alzheimer's diseas	99	Smo	3	Rat	201	10.203	23.092	4.3904

	Adjusted Life Years)				ale		es		e and other dementias		king		e	2	60272	66994	14881
2	DALYs (Disability-Adjusted Life Years)	1	Global	3	Both	22	All ages	543	Alzheimer's disease and other dementias	99	Smoking	3	Rate	2012	16.9814932 44	39.5248069 7	7.33649074 6
2	DALYs (Disability-Adjusted Life Years)	1	Global	1	Male	22	All ages	543	Alzheimer's disease and other dementias	99	Smoking	1	Number	2013	874805.524097812	2069969.63328615	377152.285293904
2	DALYs (Disability-Adjusted Life Years)	1	Global	2	Female	22	All ages	543	Alzheimer's disease and other dementias	99	Smoking	1	Number	2013	367220.31956894	824273.815828885	158278.830452465
2	DALYs (Disability-Adjusted Life Years)	1	Global	3	Both	22	All ages	543	Alzheimer's disease and other dementias	99	Smoking	1	Number	2013	1242025.84366675	2905622.04376274	537214.205791714
2	DALYs (Disability-Adjusted Life Years)	1	Global	1	Male	22	All ages	543	Alzheimer's disease and other dementias	99	Smoking	3	Rate	2013	24.14531337	57.13277303	10.40969663
2	DALYs (Disab	1	Glob	2	Fe	22	Al l	54	Alzheimer's	99	Sm	3	Ra	20	10.2	22.9	4.40

	ility-Adjusted Life Years)		al		male		ages	3	disease and other dementias		oking		te	13	2006152	40258622	5037787
2	DALYs (Disability-Adjusted Life Years)	1	Global	3	Both	22	All ages	543	Alzheimer's disease and other dementias	99	Smoking	3	Rate	2013	17.211158884	40.26516212	7.44453916
2	DALYs (Disability-Adjusted Life Years)	1	Global	1	Male	22	All ages	543	Alzheimer's disease and other dementias	99	Smoking	1	Number	2014	902493.274030974	2080909.61600246	389401.730858334
2	DALYs (Disability-Adjusted Life Years)	1	Global	2	Female	22	All ages	543	Alzheimer's disease and other dementias	99	Smoking	1	Number	2014	372972.711486547	848605.213979432	161887.651492198
2	DALYs (Disability-Adjusted Life Years)	1	Global	3	Both	22	All ages	543	Alzheimer's disease and other dementias	99	Smoking	1	Number	2014	1275465.98551752	2928063.52295693	550420.955848273
2	DALYs (Disability-Adjusted Life Years)	1	Global	1	Male	22	All ages	543	Alzheimer's disease and other dementias	99	Smoking	3	Rate	2014	24.60644345	56.73591844	10.6170228
2	DALYs	1	Gl	2	F	2	Al	5	Alzhei	9	S	3	R	2	10	23	4.

	(Disability-Adjusted Life Years)		obal		emale	2	l ages	43	mer's diseases and other dementias	9	moking		ate	014	.250972261	.323499939	449402948
2	DALYs (Disability-Adjusted Life Years)	1	Global	3	Both	22	All ages	543	Alzheimer's diseases and other dementias	99	Smoking	3	Rate	2014	17.457491433	40.076838815	7.533692964
2	DALYs (Disability-Adjusted Life Years)	1	Global	1	Male	22	All ages	543	Alzheimer's diseases and other dementias	99	Smoking	1	Number	2015	929792.494149018	2174686.64033883	398448.530298651
2	DALYs (Disability-Adjusted Life Years)	1	Global	2	Female	22	All ages	543	Alzheimer's diseases and other dementias	99	Smoking	1	Number	2015	379468.411698241	865511.010506315	164009.993872745
2	DALYs (Disability-Adjusted Life Years)	1	Global	3	Both	22	All ages	543	Alzheimer's diseases and other dementias	99	Smoking	1	Number	2015	1309260.90584726	3027029.13278562	565830.758852226
2	DALYs (Disability-Adjusted Life Years)	1	Global	1	Male	22	All ages	543	Alzheimer's diseases and other dementias	99	Smoking	3	Rate	2015	25.0430244	58.57299444	10.73180987

2	DALYs (Disability-Adjusted Life Years)	1	Global	2	Female	22	All ages	543	Alzheimer's disease and other dementias	99	Smoking	3	Rate	2015	10.30147481	23.49613945	4.452400537
2	DALYs (Disability-Adjusted Life Years)	1	Global	3	Both	22	All ages	543	Alzheimer's disease and other dementias	99	Smoking	3	Rate	2015	17.70129986	40.92564754	7.650071799
2	DALYs (Disability-Adjusted Life Years)	1	Global	1	Male	22	All ages	543	Alzheimer's disease and other dementias	99	Smoking	1	Number	2016	95645 5.572984208	2226052.32035585	415674.149106264
2	DALYs (Disability-Adjusted Life Years)	1	Global	2	Female	22	All ages	543	Alzheimer's disease and other dementias	99	Smoking	1	Number	2016	386455.651937613	882681.492240456	167032.988879882
2	DALYs (Disability-Adjusted Life Years)	1	Global	3	Both	22	All ages	543	Alzheimer's disease and other dementias	99	Smoking	1	Number	2016	1342911.22492182	3117316.08613557	582351.917948525
2	DALYs (Disability-Adjusted Life	1	Global	1	Male	22	All ages	543	Alzheimer's disease and other dement	99	Smokin	3	Rate	2016	25.45126395	59.23520838	11.06108092

	Years)								ias		g						
2	DALYs (Disability-Adjusted Life Years)	1	Global	2	Female	22	All ages	543	Alzheimer’s disease and other dementias	99	Smoking	3	Rate	2016	10.364566617	23.673119926	4.479749378
2	DALYs (Disability-Adjusted Life Years)	1	Global	3	Both	22	All ages	543	Alzheimer’s disease and other dementias	99	Smoking	3	Rate	2016	17.937502599	41.638541944	7.778577497
2	DALYs (Disability-Adjusted Life Years)	1	Global	1	Male	22	All ages	543	Alzheimer’s disease and other dementias	99	Smoking	1	Number	2017	984414.870833267	2289817.25934173	424973.900175287
2	DALYs (Disability-Adjusted Life Years)	1	Global	2	Female	22	All ages	543	Alzheimer’s disease and other dementias	99	Smoking	1	Number	2017	392972.30887393	892635.52391561	172704.617574248
2	DALYs (Disability-Adjusted Life Years)	1	Global	3	Both	22	All ages	543	Alzheimer’s disease and other dementias	99	Smoking	1	Number	2017	1377387.1797072	3190151.46259718	594375.38274236
2	DALYs (Disability-Adjusted	1	Global	1	Male	22	All ages	543	Alzheimer’s disease and other	99	Smoki	3	Rate	2017	25.8877567	60.2167175	11.1757972

	Life Years)								dementias		ng				5	3	
2	DALYs (Disability-Adjusted Life Years)	1	Global	2	Female	22	All ages	543	Alzheimer's disease and other dementias	99	Smoking	3	Rate	2017	10.41558054	23.65896268	4.577469743
2	DALYs (Disability-Adjusted Life Years)	1	Global	3	Both	22	All ages	543	Alzheimer's disease and other dementias	99	Smoking	3	Rate	2017	18.18199761	42.11112687	7.845965135
2	DALYs (Disability-Adjusted Life Years)	1	Global	1	Male	22	All ages	543	Alzheimer's disease and other dementias	99	Smoking	1	Number	2018	1010436.04367688	2358377.75069506	431232.103771516
2	DALYs (Disability-Adjusted Life Years)	1	Global	2	Female	22	All ages	543	Alzheimer's disease and other dementias	99	Smoking	1	Number	2018	399520.529831953	907401.602124433	175254.857982343
2	DALYs (Disability-Adjusted Life Years)	1	Global	3	Both	22	All ages	543	Alzheimer's disease and other dementias	99	Smoking	1	Number	2018	1409956.57350884	3266980.60053638	605811.551850031
2	DALYs (Disability-Adjust	1	Global	1	Male	22	All ages	543	Alzheimer's disease and	99	Smok	3	Rate	2018	26.27220	61.31984	11.21240

	ed Life Years)								other dementias		ing				37	41	453
2	DALYs (Disability-Adjusted Life Years)	1	Global	2	Female	22	All ages	543	Alzheimer's disease and other dementias	99	Smoking	3	Rate	2018	10.469615527	23.778867333	4.592632421
2	DALYs (Disability-Adjusted Life Years)	1	Global	3	Both	22	All ages	543	Alzheimer's disease and other dementias	99	Smoking	3	Rate	2018	18.401874044	42.638593771	7.906674628
2	DALYs (Disability-Adjusted Life Years)	1	Global	1	Male	22	All ages	543	Alzheimer's disease and other dementias	99	Smoking	1	Number	2019	1040399.98440429	2399554.34255912	444068.489169257
2	DALYs (Disability-Adjusted Life Years)	1	Global	2	Female	22	All ages	543	Alzheimer's disease and other dementias	99	Smoking	1	Number	2019	406074.41146397	915473.611673137	178568.074511172
2	DALYs (Disability-Adjusted Life Years)	1	Global	3	Both	22	All ages	543	Alzheimer's disease and other dementias	99	Smoking	1	Number	2019	1446474.42555068	3326850.29490789	626848.810245056
2	DALYs (Disability-	1	Global	1	Mal	22	All ag	543	Alzheimer's diseas	99	Smo	3	Rat	201	26.760	61.720	11.422

	Adjusted Life Years)				e		es		e and other dementias		king		e	9	90871	73785	21884
2	DALYs (Disability-Adjusted Life Years)	1	Global	2	Female	22	All ages	543	Alzheimer's disease and other dementias	99	Smoking	3	Rate	2019	10.52669514	23.73188421	4.629032246
2	DALYs (Disability-Adjusted Life Years)	1	Global	3	Both	22	All ages	543	Alzheimer's disease and other dementias	99	Smoking	3	Rate	2019	18.67544328	42.95299917	8.093250179
2	DALYs (Disability-Adjusted Life Years)	1	Global	1	Male	22	All ages	543	Alzheimer's disease and other dementias	99	Smoking	1	Number	2020	1064761.66779883	2413800.04354398	446959.377141455
2	DALYs (Disability-Adjusted Life Years)	1	Global	2	Female	22	All ages	543	Alzheimer's disease and other dementias	99	Smoking	1	Number	2020	411906.715688514	935222.312344447	179646.559281839
2	DALYs (Disability-Adjusted Life Years)	1	Global	3	Both	22	All ages	543	Alzheimer's disease and other dementias	99	Smoking	1	Number	2020	1476668.38348735	3348725.90612074	637687.525668535
2	DALYs (Disab	1	Glob	1	Ma	22	Al	54	Alzheimer's	99	Sm	3	Ra	20	27.1	61.4	11.3

	ility-Adjusted Life Years)		al		le		ages	3	disease and other dementias		oking		te	20	2134208	8370915	8483712
2	DALYs (Disability-Adjusted Life Years)	1	Global	2	Female	22	All ages	543	Alzheimer's disease and other dementias	99	Smoking	3	Rate	2020	10.57134516	24.00193414	4.610523965
2	DALYs (Disability-Adjusted Life Years)	1	Global	3	Both	22	All ages	543	Alzheimer's disease and other dementias	99	Smoking	3	Rate	2020	18.87752146	42.8096422	8.152107869
2	DALYs (Disability-Adjusted Life Years)	1	Global	1	Male	22	All ages	543	Alzheimer's disease and other dementias	99	Smoking	1	Number	2021	1110022.87585639	2558503.49853865	474038.472953299
2	DALYs (Disability-Adjusted Life Years)	1	Global	2	Female	22	All ages	543	Alzheimer's disease and other dementias	99	Smoking	1	Number	2021	423190.661847443	947536.46861841	185084.811077046
2	DALYs (Disability-Adjusted Life Years)	1	Global	3	Both	22	All ages	543	Alzheimer's disease and other dementias	99	Smoking	1	Number	2021	1533213.53770383	3496419.97439078	662722.708565097
2	DALYs	1	Gl	1	M	2	Al	5	Alzhei	9	S	3	R	2	28	64	11

	(Disability-Adjusted Life Years)		obal		ale	2	l ages	43	mer's disease and other dementias	9	moking		ate	021	.03518907	.6186046	.97250841
2	DALYs (Disability-Adjusted Life Years)	1	Global	2	Female	22	All ages	543	Alzheimer's disease and other dementias	99	Smoking	3	Rate	2021	10.7628374	24.0983128	4.707187343
2	DALYs (Disability-Adjusted Life Years)	1	Global	3	Both	22	All ages	543	Alzheimer's disease and other dementias	99	Smoking	3	Rate	2021	19.42903174	44.30697551	8.39808691
2	DALYs (Disability-Adjusted Life Years)	1	Global	1	Male	27	Age-standardized	543	Alzheimer's disease and other dementias	99	Smoking	3	Rate	1990	37.44794545	87.68818274	15.97604889
2	DALYs (Disability-Adjusted Life Years)	1	Global	2	Female	27	Age-standardized	543	Alzheimer's disease and other dementias	99	Smoking	3	Rate	1990	13.65227898	31.32023152	5.797071706
2	DALYs (Disability-Adjusted Life Years)	1	Global	3	Both	27	Age-standardized	543	Alzheimer's disease and other dementias	99	Smoking	3	Rate	1990	23.32797861	54.45536232	9.992748523

2	DALYs (Disability-Adjusted Life Years)	1	Global	1	Male	27	Age-standardized	543	Alzheimer's disease and other dementias	99	Smoking	3	Rate	1991	37.23095056	87.18332166	15.7681488
2	DALYs (Disability-Adjusted Life Years)	1	Global	2	Female	27	Age-standardized	543	Alzheimer's disease and other dementias	99	Smoking	3	Rate	1991	13.61760853	30.97813272	5.819379904
2	DALYs (Disability-Adjusted Life Years)	1	Global	3	Both	27	Age-standardized	543	Alzheimer's disease and other dementias	99	Smoking	3	Rate	1991	23.24491689	53.69685945	9.898586706
2	DALYs (Disability-Adjusted Life Years)	1	Global	1	Male	27	Age-standardized	543	Alzheimer's disease and other dementias	99	Smoking	3	Rate	1992	36.95204211	84.60722031	15.7403008
2	DALYs (Disability-Adjusted Life Years)	1	Global	2	Female	27	Age-standardized	543	Alzheimer's disease and other dementias	99	Smoking	3	Rate	1992	13.56884686	30.95686334	5.817507009
2	DALYs (Disability-Adjust	1	Global	3	Both	27	Age-stan	543	Alzheimer's disease and	99	Smok	3	Rate	1992	23.13047	52.71307	9.888646

	ed Life Years)						dardized		other dementias		ing				853	18	047
2	DALYs (Disability-Adjusted Life Years)	1	Global	1	Male	27	Age-standardized	543	Alzheimer's disease and other dementias	99	Smoking	3	Rate	1993	36.68955861	85.7539661	15.64043288
2	DALYs (Disability-Adjusted Life Years)	1	Global	2	Female	27	Age-standardized	543	Alzheimer's disease and other dementias	99	Smoking	3	Rate	1993	13.52252385	30.83468843	5.727870955
2	DALYs (Disability-Adjusted Life Years)	1	Global	3	Both	27	Age-standardized	543	Alzheimer's disease and other dementias	99	Smoking	3	Rate	1993	23.0239978	53.07657035	9.887566194
2	DALYs (Disability-Adjusted Life Years)	1	Global	1	Male	27	Age-standardized	543	Alzheimer's disease and other dementias	99	Smoking	3	Rate	1994	36.3542828	84.69583152	15.44884981
2	DALYs (Disability-Adjusted Life Years)	1	Global	2	Female	27	Age-standardized	543	Alzheimer's disease and other dementias	99	Smoking	3	Rate	1994	13.44947635	30.76469149	5.706788052

2	DALYs (Disability-Adjusted Life Years)	1	Global	3	Both	27	Age-standardized	543	Alzheimer's disease and other dementias	99	Smoking	3	Rate	1994	22.86720672	52.69775916	9.831111227
2	DALYs (Disability-Adjusted Life Years)	1	Global	1	Male	27	Age-standardized	543	Alzheimer's disease and other dementias	99	Smoking	3	Rate	1995	36.0185256	83.83211164	15.34757601
2	DALYs (Disability-Adjusted Life Years)	1	Global	2	Female	27	Age-standardized	543	Alzheimer's disease and other dementias	99	Smoking	3	Rate	1995	13.37638985	30.54739225	5.650263674
2	DALYs (Disability-Adjusted Life Years)	1	Global	3	Both	27	Age-standardized	543	Alzheimer's disease and other dementias	99	Smoking	3	Rate	1995	22.71078786	52.47531119	9.762974333
2	DALYs (Disability-Adjusted Life Years)	1	Global	1	Male	27	Age-standardized	543	Alzheimer's disease and other dementias	99	Smoking	3	Rate	1996	35.62641635	82.88259071	15.17339488
2	DALYs (Disability-Adjust	1	Global	2	Fema	27	Age-stan	543	Alzheimer's diseas e and	99	Smok	3	Rate	1996	13.25636	30.33653	5.600794

	ed Life Years)				le		dardized		other dementias		ing				097	368	207
2	DALYs (Disability-Adjusted Life Years)	1	Global	3	Both	27	Age-standardized	543	Alzheimer's disease and other dementias	99	Smoking	3	Rate	1996	22.500296672	52.081068836	9.62583454
2	DALYs (Disability-Adjusted Life Years)	1	Global	1	Male	27	Age-standardized	543	Alzheimer's disease and other dementias	99	Smoking	3	Rate	1997	35.134737757	81.338855541	14.978251198
2	DALYs (Disability-Adjusted Life Years)	1	Global	2	Female	27	Age-standardized	543	Alzheimer's disease and other dementias	99	Smoking	3	Rate	1997	13.080400555	29.859152666	5.542606829
2	DALYs (Disability-Adjusted Life Years)	1	Global	3	Both	27	Age-standardized	543	Alzheimer's disease and other dementias	99	Smoking	3	Rate	1997	22.211470023	51.048176652	9.50812963
2	DALYs (Disability-Adjusted Life Years)	1	Global	1	Male	27	Age-standardized	543	Alzheimer's disease and other dementias	99	Smoking	3	Rate	1998	34.623353315	80.598942236	14.7249626

2	DALYs (Disability-Adjusted Life Years)	1	Global	2	Female	27	Age-standardized	543	Alzheimer's disease and other dementias	99	Smoking	3	Rate	1998	12.885926643	29.470377044	5.497726743
2	DALYs (Disability-Adjusted Life Years)	1	Global	3	Both	27	Age-standardized	543	Alzheimer's disease and other dementias	99	Smoking	3	Rate	1998	21.900251117	50.400150075	9.379563214
2	DALYs (Disability-Adjusted Life Years)	1	Global	1	Male	27	Age-standardized	543	Alzheimer's disease and other dementias	99	Smoking	3	Rate	1999	34.125765538	79.932216626	14.478715223
2	DALYs (Disability-Adjusted Life Years)	1	Global	2	Female	27	Age-standardized	543	Alzheimer's disease and other dementias	99	Smoking	3	Rate	1999	12.679827996	28.838681022	5.421977773
2	DALYs (Disability-Adjusted Life Years)	1	Global	3	Both	27	Age-standardized	543	Alzheimer's disease and other dementias	99	Smoking	3	Rate	1999	21.590073325	50.061304402	9.213866727
2	DALYs (Disability-Adjust	1	Global	1	Male	27	Age-stan	543	Alzheimer's disease and	99	Smok	3	Rate	2000	33.73262	78.05083	14.25865

	ed Life Years)						dardized		other dementias		ing				218	542	038
2	DALYs (Disability-Adjusted Life Years)	1	Global	2	Female	27	Age-standardized	543	Alzheimer's disease and other dementias	99	Smoking	3	Rate	2000	12.479005888	28.330796288	5.33653707
2	DALYs (Disability-Adjusted Life Years)	1	Global	3	Both	27	Age-standardized	543	Alzheimer's disease and other dementias	99	Smoking	3	Rate	2000	21.329723314	49.1294309	9.111669475
2	DALYs (Disability-Adjusted Life Years)	1	Global	1	Male	27	Age-standardized	543	Alzheimer's disease and other dementias	99	Smoking	3	Rate	2001	33.373309934	77.755904748	14.20714517
2	DALYs (Disability-Adjusted Life Years)	1	Global	2	Female	27	Age-standardized	543	Alzheimer's disease and other dementias	99	Smoking	3	Rate	2001	12.278342201	27.876681711	5.274364411
2	DALYs (Disability-Adjusted Life Years)	1	Global	3	Both	27	Age-standardized	543	Alzheimer's disease and other dementias	99	Smoking	3	Rate	2001	21.081739901	48.9130285	9.062536283

2	DALYs (Disability-Adjusted Life Years)	1	Global	1	Male	27	Age-standardized	543	Alzheimer's disease and other dementias	99	Smoking	3	Rate	2002	33.08647275	77.07399432	14.08932102
2	DALYs (Disability-Adjusted Life Years)	1	Global	2	Female	27	Age-standardized	543	Alzheimer's disease and other dementias	99	Smoking	3	Rate	2002	12.07472145	27.29117898	5.203535846
2	DALYs (Disability-Adjusted Life Years)	1	Global	3	Both	27	Age-standardized	543	Alzheimer's disease and other dementias	99	Smoking	3	Rate	2002	20.86466611	48.40662635	8.918664346
2	DALYs (Disability-Adjusted Life Years)	1	Global	1	Male	27	Age-standardized	543	Alzheimer's disease and other dementias	99	Smoking	3	Rate	2003	32.77261612	76.35684966	13.91618896
2	DALYs (Disability-Adjusted Life Years)	1	Global	2	Female	27	Age-standardized	543	Alzheimer's disease and other dementias	99	Smoking	3	Rate	2003	11.86899537	26.69861346	5.130798621
2	DALYs (Disability-Adjust	1	Global	3	Both	27	Age-stan	543	Alzheimer's disease and	99	Smok	3	Rate	2003	20.63057	47.95224	8.802333

	ed Life Years)						dardized		other dementias		ing				162	442	576
2	DALYs (Disability-Adjusted Life Years)	1	Global	1	Male	27	Age-standardized	543	Alzheimer's disease and other dementias	99	Smoking	3	Rate	2004	32.442596659	75.114949494	13.706383363
2	DALYs (Disability-Adjusted Life Years)	1	Global	2	Female	27	Age-standardized	543	Alzheimer's disease and other dementias	99	Smoking	3	Rate	2004	11.639371185	26.345202275	5.002796851
2	DALYs (Disability-Adjusted Life Years)	1	Global	3	Both	27	Age-standardized	543	Alzheimer's disease and other dementias	99	Smoking	3	Rate	2004	20.376394446	47.060068533	8.666018237
2	DALYs (Disability-Adjusted Life Years)	1	Global	1	Male	27	Age-standardized	543	Alzheimer's disease and other dementias	99	Smoking	3	Rate	2005	32.150185334	74.924342866	13.593961288
2	DALYs (Disability-Adjusted Life Years)	1	Global	2	Female	27	Age-standardized	543	Alzheimer's disease and other dementias	99	Smoking	3	Rate	2005	11.462611116	26.201957566	4.925870466

2	DALYs (Disability-Adjusted Life Years)	1	Global	3	Both	27	Age-standardized	543	Alzheimer's disease and other dementias	99	Smoking	3	Rate	2005	20.172199907	46.634329968	8.601651502
2	DALYs (Disability-Adjusted Life Years)	1	Global	1	Male	27	Age-standardized	543	Alzheimer's disease and other dementias	99	Smoking	3	Rate	2006	31.8603753	73.43019128	13.46048283
2	DALYs (Disability-Adjusted Life Years)	1	Global	2	Female	27	Age-standardized	543	Alzheimer's disease and other dementias	99	Smoking	3	Rate	2006	11.26112762	25.64220201	4.844191774
2	DALYs (Disability-Adjusted Life Years)	1	Global	3	Both	27	Age-standardized	543	Alzheimer's disease and other dementias	99	Smoking	3	Rate	2006	19.95704239	45.91140937	8.534672466
2	DALYs (Disability-Adjusted Life Years)	1	Global	1	Male	27	Age-standardized	543	Alzheimer's disease and other dementias	99	Smoking	3	Rate	2007	31.69193297	74.1661146	13.46812072
2	DALYs (Disability-Adjust	1	Global	2	Fema	27	Age-stan	543	Alzheimer's disease and	99	Smok	3	Rate	2007	11.06656	25.06136	4.755624

	ed Life Years)				le		dardized		other dementias		ing				204	268	465
2	DALYs (Disability-Adjusted Life Years)	1	Global	3	Both	27	Age-standardized	543	Alzheimer’s disease and other dementias	99	Smoking	3	Rate	2007	19.80044547	45.82814798	8.491322148
2	DALYs (Disability-Adjusted Life Years)	1	Global	1	Male	27	Age-standardized	543	Alzheimer’s disease and other dementias	99	Smoking	3	Rate	2008	31.54872514	73.55356336	13.38688358
2	DALYs (Disability-Adjusted Life Years)	1	Global	2	Female	27	Age-standardized	543	Alzheimer’s disease and other dementias	99	Smoking	3	Rate	2008	10.88450654	24.86672087	4.640976092
2	DALYs (Disability-Adjusted Life Years)	1	Global	3	Both	27	Age-standardized	543	Alzheimer’s disease and other dementias	99	Smoking	3	Rate	2008	19.66212257	45.36995039	8.431673241
2	DALYs (Disability-Adjusted Life Years)	1	Global	1	Male	27	Age-standardized	543	Alzheimer’s disease and other dementias	99	Smoking	3	Rate	2009	31.42907223	74.02941181	13.37662688

2	DALYs (Disability-Adjusted Life Years)	1	Global	2	Female	27	Age-standardized	543	Alzheimer's disease and other dementias	99	Smoking	3	Rate	2009	10.69396601	24.36659236	4.586342635
2	DALYs (Disability-Adjusted Life Years)	1	Global	3	Both	27	Age-standardized	543	Alzheimer's disease and other dementias	99	Smoking	3	Rate	2009	19.52845744	45.32594671	8.341019394
2	DALYs (Disability-Adjusted Life Years)	1	Global	1	Male	27	Age-standardized	543	Alzheimer's disease and other dementias	99	Smoking	3	Rate	2010	31.29720329	73.52899734	13.21783866
2	DALYs (Disability-Adjusted Life Years)	1	Global	2	Female	27	Age-standardized	543	Alzheimer's disease and other dementias	99	Smoking	3	Rate	2010	10.53185575	23.6633412	4.51909381
2	DALYs (Disability-Adjusted Life Years)	1	Global	3	Both	27	Age-standardized	543	Alzheimer's disease and other dementias	99	Smoking	3	Rate	2010	19.39981816	44.88310802	8.279838192
2	DALYs (Disability-Adjust	1	Global	1	Male	27	Age-stan	543	Alzheimer's disease and	99	Smok	3	Rate	2011	31.15994	73.25874	13.13892

	ed Life Years)						dardized		other dementias		ing				432	469	806
2	DALYs (Disability-Adjusted Life Years)	1	Global	2	Female	27	Age-standardized	543	Alzheimer's disease and other dementias	99	Smoking	3	Rate	2011	10.358672213	23.510447766	4.439202909
2	DALYs (Disability-Adjusted Life Years)	1	Global	3	Both	27	Age-standardized	543	Alzheimer's disease and other dementias	99	Smoking	3	Rate	2011	19.261856775	44.560949919	8.243981036
2	DALYs (Disability-Adjusted Life Years)	1	Global	1	Male	27	Age-standardized	543	Alzheimer's disease and other dementias	99	Smoking	3	Rate	2012	31.048804885	72.701629941	13.127349917
2	DALYs (Disability-Adjusted Life Years)	1	Global	2	Female	27	Age-standardized	543	Alzheimer's disease and other dementias	99	Smoking	3	Rate	2012	10.181085811	23.047787741	4.372132746
2	DALYs (Disability-Adjusted Life Years)	1	Global	3	Both	27	Age-standardized	543	Alzheimer's disease and other dementias	99	Smoking	3	Rate	2012	19.133982238	44.368084413	8.181190268

2	DALYs (Disability-Adjusted Life Years)	1	Global	1	Male	27	Age-standardized	543	Alzheimer's disease and other dementias	99	Smoking	3	Rate	2013	30.970333264	73.331441662	13.164040065
2	DALYs (Disability-Adjusted Life Years)	1	Global	2	Female	27	Age-standardized	543	Alzheimer's disease and other dementias	99	Smoking	3	Rate	2013	10.002440886	22.467976639	4.306838832
2	DALYs (Disability-Adjusted Life Years)	1	Global	3	Both	27	Age-standardized	543	Alzheimer's disease and other dementias	99	Smoking	3	Rate	2013	19.014224412	44.560252267	8.147627298
2	DALYs (Disability-Adjusted Life Years)	1	Global	1	Male	27	Age-standardized	543	Alzheimer's disease and other dementias	99	Smoking	3	Rate	2014	30.914288835	73.8671811	13.121946677
2	DALYs (Disability-Adjusted Life Years)	1	Global	2	Female	27	Age-standardized	543	Alzheimer's disease and other dementias	99	Smoking	3	Rate	2014	9.83997678	22.383640013	4.265726665
2	DALYs (Disability-Adjusted	1	Global	3	Both	27	Age-stan	543	Alzheimer's disease and	99	Smok	3	Rate	2014	18.91053	44.17019	8.095514

	ed Life Years)						dardized		other dementias		ing				921	018	774
2	DALYs (Disability-Adjusted Life Years)	1	Global	1	Male	27	Age-standardized	543	Alzheimer’s disease and other dementias	99	Smoking	3	Rate	2015	30.83770248	72.26717221	13.17233016
2	DALYs (Disability-Adjusted Life Years)	1	Global	2	Female	27	Age-standardized	543	Alzheimer’s disease and other dementias	99	Smoking	3	Rate	2015	9.702730374	22.1201809	4.197622923
2	DALYs (Disability-Adjusted Life Years)	1	Global	3	Both	27	Age-standardized	543	Alzheimer’s disease and other dementias	99	Smoking	3	Rate	2015	18.81223228	43.79666065	8.115008006
2	DALYs (Disability-Adjusted Life Years)	1	Global	1	Male	27	Age-standardized	543	Alzheimer’s disease and other dementias	99	Smoking	3	Rate	2016	30.68594254	73.03516788	13.02600778
2	DALYs (Disability-Adjusted Life Years)	1	Global	2	Female	27	Age-standardized	543	Alzheimer’s disease and other dementias	99	Smoking	3	Rate	2016	9.567656923	21.87191003	4.138512415

2	DALYs (Disability-Adjusted Life Years)	1	Global	3	Both	27	Age-standardized	543	Alzheimer's disease and other dementias	99	Smoking	3	Rate	2016	18.684596244	43.724787709	8.039999282
2	DALYs (Disability-Adjusted Life Years)	1	Global	1	Male	27	Age-standardized	543	Alzheimer's disease and other dementias	99	Smoking	3	Rate	2017	30.537082999	71.574893555	12.983535594
2	DALYs (Disability-Adjusted Life Years)	1	Global	2	Female	27	Age-standardized	543	Alzheimer's disease and other dementias	99	Smoking	3	Rate	2017	9.4202865558	21.3912726 8	4.1443979757
2	DALYs (Disability-Adjusted Life Years)	1	Global	3	Both	27	Age-standardized	543	Alzheimer's disease and other dementias	99	Smoking	3	Rate	2017	18.554181145	43.247505551	7.980969395
2	DALYs (Disability-Adjusted Life Years)	1	Global	1	Male	27	Age-standardized	543	Alzheimer's disease and other dementias	99	Smoking	3	Rate	2018	30.318053566	70.216796623	12.739193365
2	DALYs (Disability-Adjust	1	Global	2	Fema	27	Age-stan	543	Alzheimer's diseas e and	99	Smok	3	Rate	2018	9.27634 8	21.08084	4.072387

	ed Life Years)				le		dardized		other dementias		ing				563	133	013
2	DALYs (Disability-Adjusted Life Years)	1	Global	3	Both	27	Age-standardized	543	Alzheimer's disease and other dementias	99	Smoking	3	Rate	2018	18.39537057	42.56085503	7.88361252
2	DALYs (Disability-Adjusted Life Years)	1	Global	1	Male	27	Age-standardized	543	Alzheimer's disease and other dementias	99	Smoking	3	Rate	2019	30.18809559	70.04136068	12.73746277
2	DALYs (Disability-Adjusted Life Years)	1	Global	2	Female	27	Age-standardized	543	Alzheimer's disease and other dementias	99	Smoking	3	Rate	2019	9.13017424	20.57758326	4.017721461
2	DALYs (Disability-Adjusted Life Years)	1	Global	3	Both	27	Age-standardized	543	Alzheimer's disease and other dementias	99	Smoking	3	Rate	2019	18.27369837	42.00540574	7.909247104
2	DALYs (Disability-Adjusted Life Years)	1	Global	1	Male	27	Age-standardized	543	Alzheimer's disease and other dementias	99	Smoking	3	Rate	2020	30.02520759	68.76829574	12.4684272

2	DALYs (Disability-Adjusted Life Years)	1	Global	2	Female	27	Age-standardized	543	Alzheimer's disease and other dementias	99	Smoking	3	Rate	2020	8.99623159	20.42739727	3.924443402
2	DALYs (Disability-Adjusted Life Years)	1	Global	3	Both	27	Age-standardized	543	Alzheimer's disease and other dementias	99	Smoking	3	Rate	2020	18.12911782	41.28998316	7.801428159
2	DALYs (Disability-Adjusted Life Years)	1	Global	1	Male	27	Age-standardized	543	Alzheimer's disease and other dementias	99	Smoking	3	Rate	2021	30.56404804	71.49660024	12.71560393
2	DALYs (Disability-Adjusted Life Years)	1	Global	2	Female	27	Age-standardized	543	Alzheimer's disease and other dementias	99	Smoking	3	Rate	2021	9.00580001	20.15524777	3.941251701
2	DALYs (Disability-Adjusted Life Years)	1	Global	3	Both	27	Age-standardized	543	Alzheimer's disease and other dementias	99	Smoking	3	Rate	2021	18.35809318	42.07070102	7.903082991
3	YLDs (Years Lived with	1	Global	1	Male	22	All ages	543	Alzheimer's disease and	99	Smok	1	Numb	1990	177737.3	258944.7	108959.2

	Disability)								other dementias		ing		er		26960568	00460268	21513366
3	YLDs (Years Lived with Disability)	1	Global	2	Female	22	All ages	543	Alzheimer's disease and other dementias	99	Smoking	1	Number	1990	89156.3678777924	133867.079309177	53275.1262766676
3	YLDs (Years Lived with Disability)	1	Global	3	Both	22	All ages	543	Alzheimer's disease and other dementias	99	Smoking	1	Number	1990	266893.694838836	391752.479841494	162663.225675174
3	YLDs (Years Lived with Disability)	1	Global	1	Male	22	All ages	543	Alzheimer's disease and other dementias	99	Smoking	3	Rate	1990	6.617808416	9.641454877	4.0569489
3	YLDs (Years Lived with Disability)	1	Global	2	Female	22	All ages	543	Alzheimer's disease and other dementias	99	Smoking	3	Rate	1990	3.367084381	5.055631612	2.011991401
3	YLDs (Years Lived with Disability)	1	Global	3	Both	22	All ages	543	Alzheimer's disease and other dementias	99	Smoking	3	Rate	1990	5.0039846	7.344959488	3.049769597
3	YLDs (Years Lived	1	Global	1	Mal	22	All ag	543	Alzheimer's diseas	99	Smo	1	Num	199	182125	265239	111731

	with Disability)				e		es		e and other dementias		king		ber	1	.410537271	.047618051	.951971803
3	YLDs (Years Lived with Disability)	1	Global	2	Female	22	All ages	543	Alzheimer's disease and other dementias	99	Smoking	1	Number	1991	91383.5066521938	137622.364424248	54835.4379467265
3	YLDs (Years Lived with Disability)	1	Global	3	Both	22	All ages	543	Alzheimer's disease and other dementias	99	Smoking	1	Number	1991	273508.917189464	400563.854276282	166781.143421795
3	YLDs (Years Lived with Disability)	1	Global	1	Male	22	All ages	543	Alzheimer's disease and other dementias	99	Smoking	3	Rate	1991	6.67475608	9.720806891	4.094890019
3	YLDs (Years Lived with Disability)	1	Global	2	Female	22	All ages	543	Alzheimer's disease and other dementias	99	Smoking	3	Rate	1991	3.399755298	5.119986962	2.040051619
3	YLDs (Years Lived with Disability)	1	Global	3	Both	22	All ages	543	Alzheimer's disease and other dementias	99	Smoking	3	Rate	1991	5.049537781	7.395233532	3.079123319
3	YLDs (Years	1	Glob	1	Ma	22	All	54	Alzheimer's	99	Sm	1	Nu	19	1862	2719	1144

	Lived with Disability)		al		le		ages	3	disease and other dementias		oking		mber	92	90.166493966	47.064979959	10.03741705
3	YLDs (Years Lived with Disability)	1	Global	2	Female	22	All ages	543	Alzheimer's disease and other dementias	99	Smoking	1	Number	1992	93553.1778426485	140619.1647325771	56014.6806810077
3	YLDs (Years Lived with Disability)	1	Global	3	Both	22	All ages	543	Alzheimer's disease and other dementias	99	Smoking	1	Number	1992	279843.3443366614	411087.5173155575	170420.84289558
3	YLDs (Years Lived with Disability)	1	Global	1	Male	22	All ages	543	Alzheimer's disease and other dementias	99	Smoking	3	Rate	1992	6.724933161	9.817081974	4.130115234
3	YLDs (Years Lived with Disability)	1	Global	2	Female	22	All ages	543	Alzheimer's disease and other dementias	99	Smoking	3	Rate	1992	3.430543066	5.156426662	2.054027226
3	YLDs (Years Lived with Disability)	1	Global	3	Both	22	All ages	543	Alzheimer's disease and other dementias	99	Smoking	3	Rate	1992	5.090645321	7.478115127	3.100134715
3	YLDs	1	Gl	1	M	2	Al	5	Alzhei	9	S	1	N	1	19	27	11

	(Years Lived with Disability)		obal		ale	2	l ages	43	mer's disease and other dementias	9	moking		umber	993	0189.195636414	7234.942366167	7069.036255708
3	YLDs (Years Lived with Disability)	1	Global	2	Female	22	All ages	543	Alzheimer's disease and other dementias	99	Smoking	1	Number	1993	95603.8556330738	143343.053730883	57256.3303637106
3	YLDs (Years Lived with Disability)	1	Global	3	Both	22	All ages	543	Alzheimer's disease and other dementias	99	Smoking	1	Number	1993	285793.0512694888	420196.159282703	174252.206463662
3	YLDs (Years Lived with Disability)	1	Global	1	Male	22	All ages	543	Alzheimer's disease and other dementias	99	Smoking	3	Rate	1993	6.767794897	9.865277688	4.165847715
3	YLDs (Years Lived with Disability)	1	Global	2	Female	22	All ages	543	Alzheimer's disease and other dementias	99	Smoking	3	Rate	1993	3.457320098	5.183711654	2.070559397
3	YLDs (Years Lived with Disability)	1	Global	3	Both	22	All ages	543	Alzheimer's disease and other dementias	99	Smoking	3	Rate	1993	5.125902251	7.536517873	3.125337629

3	YLDs (Years Lived with Disability)	1	Global	1	Male	22	All ages	543	Alzheimer's disease and other dementias	99	Smoking	1	Number	1994	193782.843229222	281579.108679155	118939.479712434
3	YLDs (Years Lived with Disability)	1	Global	2	Female	22	All ages	543	Alzheimer's disease and other dementias	99	Smoking	1	Number	1994	97484.0957495599	146371.285640762	58427.5203452242
3	YLDs (Years Lived with Disability)	1	Global	3	Both	22	All ages	543	Alzheimer's disease and other dementias	99	Smoking	1	Number	1994	291266.938978782	428304.176093191	176720.024726782
3	YLDs (Years Lived with Disability)	1	Global	1	Male	22	All ages	543	Alzheimer's disease and other dementias	99	Smoking	3	Rate	1994	6.803557164	9.886012251	4.175867873
3	YLDs (Years Lived with Disability)	1	Global	2	Female	22	All ages	543	Alzheimer's disease and other dementias	99	Smoking	3	Rate	1994	3.478839756	5.223439206	2.085057866
3	YLDs (Years Lived with Disability)	1	Global	3	Both	22	All ages	543	Alzheimer's disease and other dement	99	Smokin	3	Rate	1994	5.154748188	7.579988938	3.127533905

									ias		g						
3	YLDs (Years Lived with Disability)	1	Global	1	Male	22	All ages	543	Alzheimer's disease and other dementias	99	Smoking	1	Number	1995	197058.485439764	286984.018239854	121033.045738201
3	YLDs (Years Lived with Disability)	1	Global	2	Female	22	All ages	543	Alzheimer's disease and other dementias	99	Smoking	1	Number	1995	99242.8917528076	148749.558408982	59570.4676229625
3	YLDs (Years Lived with Disability)	1	Global	3	Both	22	All ages	543	Alzheimer's disease and other dementias	99	Smoking	1	Number	1995	296301.377192572	437237.286439198	179719.246715014
3	YLDs (Years Lived with Disability)	1	Global	1	Male	22	All ages	543	Alzheimer's disease and other dementias	99	Smoking	3	Rate	1995	6.82946017	9.946011296	4.194644871
3	YLDs (Years Lived with Disability)	1	Global	2	Female	22	All ages	543	Alzheimer's disease and other dementias	99	Smoking	3	Rate	1995	3.496138469	5.240164249	2.098554363
3	YLDs (Years Lived with Disabi	1	Global	3	Both	22	All ages	543	Alzheimer's disease and other	99	Smoki	3	Rate	1995	5.17641871	7.63858506	3.13971565

	lity)								dementias		ng				9	3	6
3	YLDs (Years Lived with Disability)	1	Global	1	Male	22	All ages	543	Alzheimer's disease and other dementias	99	Smoking	1	Number	1996	199971.405943653	291466.02458287	122466.558457708
3	YLDs (Years Lived with Disability)	1	Global	2	Female	22	All ages	543	Alzheimer's disease and other dementias	99	Smoking	1	Number	1996	100745.623803471	150955.777904072	60229.0778970882
3	YLDs (Years Lived with Disability)	1	Global	3	Both	22	All ages	543	Alzheimer's disease and other dementias	99	Smoking	1	Number	1996	300717.029747125	443678.136135222	182000.733967363
3	YLDs (Years Lived with Disability)	1	Global	1	Male	22	All ages	543	Alzheimer's disease and other dementias	99	Smoking	3	Rate	1996	6.841809865	9.97220134	4.190063593
3	YLDs (Years Lived with Disability)	1	Global	2	Female	22	All ages	543	Alzheimer's disease and other dementias	99	Smoking	3	Rate	1996	3.503453351	5.249523562	2.094480701
3	YLDs (Years Lived with	1	Global	3	Both	22	All ages	543	Alzheimer's disease and	99	Smok	3	Rate	1996	5.186212	7.651741	3.138812

	Disability)								other dementias		ing				143	369	648
3	YLDs (Years Lived with Disability)	1	Global	1	Male	22	All ages	543	Alzheimer's disease and other dementias	99	Smoking	1	Number	1997	202526.215157595	295180.232789381	124071.380244786
3	YLDs (Years Lived with Disability)	1	Global	2	Female	22	All ages	543	Alzheimer's disease and other dementias	99	Smoking	1	Number	1997	102031.264834751	152803.517432827	60899.3288466235
3	YLDs (Years Lived with Disability)	1	Global	3	Both	22	All ages	543	Alzheimer's disease and other dementias	99	Smoking	1	Number	1997	304557.479992346	448075.155323303	183983.634611061
3	YLDs (Years Lived with Disability)	1	Global	1	Male	22	All ages	543	Alzheimer's disease and other dementias	99	Smoking	3	Rate	1997	6.841878439	9.971979522	4.19146381
3	YLDs (Years Lived with Disability)	1	Global	2	Female	22	All ages	543	Alzheimer's disease and other dementias	99	Smoking	3	Rate	1997	3.502642486	5.245608716	2.090619742
3	YLDs (Years Lived	1	Global	3	Bot	22	All ag	543	Alzheimer's diseas	99	Smo	3	Rat	199	5.1856	7.6293	3.1326

	with Disability)				h		es		e and other dementias		king		e	7	55029	09195	62051
3	YLDs (Years Lived with Disability)	1	Global	1	Male	22	All ages	543	Alzheimer's disease and other dementias	99	Smoking	1	Number	1998	204827.104108657	298201.520160301	125533.099179961
3	YLDs (Years Lived with Disability)	1	Global	2	Female	22	All ages	543	Alzheimer's disease and other dementias	99	Smoking	1	Number	1998	103114.059773979	154566.048660507	61528.9574878498
3	YLDs (Years Lived with Disability)	1	Global	3	Both	22	All ages	543	Alzheimer's disease and other dementias	99	Smoking	1	Number	1998	307941.163882635	452428.079887959	186343.699775249
3	YLDs (Years Lived with Disability)	1	Global	1	Male	22	All ages	543	Alzheimer's disease and other dementias	99	Smoking	3	Rate	1998	6.833602065	9.948832372	4.188133448
3	YLDs (Years Lived with Disability)	1	Global	2	Female	22	All ages	543	Alzheimer's disease and other dementias	99	Smoking	3	Rate	1998	3.494763714	5.238585498	2.085352555
3	YLDs (Years	1	Glob	3	Bo	22	All	54	Alzheimer's	99	Sm	3	Ra	19	5.17	7.60	3.13

	Lived with Disability)		al		th		ages	3	disease and other dementias		oking		te	98	7324553	6540733	294202
3	YLDs (Years Lived with Disability)	1	Global	1	Male	22	All ages	543	Alzheimer's disease and other dementias	99	Smoking	1	Number	1999	207562.549801813	301758.724721777	126880.79544816
3	YLDs (Years Lived with Disability)	1	Global	2	Female	22	All ages	543	Alzheimer's disease and other dementias	99	Smoking	1	Number	1999	104096.056929667	156049.366037139	61978.5133348576
3	YLDs (Years Lived with Disability)	1	Global	3	Both	22	All ages	543	Alzheimer's disease and other dementias	99	Smoking	1	Number	1999	311658.60673148	458201.798514205	188775.067066344
3	YLDs (Years Lived with Disability)	1	Global	1	Male	22	All ages	543	Alzheimer's disease and other dementias	99	Smoking	3	Rate	1999	6.839879753	9.943958549	4.181146284
3	YLDs (Years Lived with Disability)	1	Global	2	Female	22	All ages	543	Alzheimer's disease and other dementias	99	Smoking	3	Rate	1999	3.483465234	5.222028167	2.074045865
3	YLDs	1	Gl	3	B	2	Al	5	Alzhei	9	S	3	R	1	5.	7.	3.

	(Years Lived with Disability)		obal		oth	2	l ages	43	mer's disease and other dementias	9	moking		ate	999	174574335	607681019	134296939
3	YLDs (Years Lived with Disability)	1	Global	1	Male	22	All ages	543	Alzheimer's disease and other dementias	99	Smoking	1	Number	2000	210805.203122687	306076.779067225	128739.094764413
3	YLDs (Years Lived with Disability)	1	Global	2	Female	22	All ages	543	Alzheimer's disease and other dementias	99	Smoking	1	Number	2000	105196.336518556	157708.764863087	62478.7287107968
3	YLDs (Years Lived with Disability)	1	Global	3	Both	22	All ages	543	Alzheimer's disease and other dementias	99	Smoking	1	Number	2000	316001.539641242	465359.476458645	191784.076097005
3	YLDs (Years Lived with Disability)	1	Global	1	Male	22	All ages	543	Alzheimer's disease and other dementias	99	Smoking	3	Rate	2000	6.8617262994	9.9628237444	4.1904678755
3	YLDs (Years Lived with Disability)	1	Global	2	Female	22	All ages	543	Alzheimer's disease and other dementias	99	Smoking	3	Rate	2000	3.4757390644	5.2107757706	2.0643281443

3	YLDs (Years Lived with Disability)	1	Global	3	Both	22	All ages	543	Alzheimer's disease and other dementias	99	Smoking	3	Rate	2000	5.181391044	7.630372391	3.144631179
3	YLDs (Years Lived with Disability)	1	Global	1	Male	22	All ages	543	Alzheimer's disease and other dementias	99	Smoking	1	Number	2001	214835.189611865	313053.024604931	131242.53173472
3	YLDs (Years Lived with Disability)	1	Global	2	Female	22	All ages	543	Alzheimer's disease and other dementias	99	Smoking	1	Number	2001	106322.714176083	160042.417739562	63103.6466466943
3	YLDs (Years Lived with Disability)	1	Global	3	Both	22	All ages	543	Alzheimer's disease and other dementias	99	Smoking	1	Number	2001	321157.903787948	472953.726285295	194429.771431279
3	YLDs (Years Lived with Disability)	1	Global	1	Male	22	All ages	543	Alzheimer's disease and other dementias	99	Smoking	3	Rate	2001	6.906812268	10.06445208	4.219362433
3	YLDs (Years Lived with Disability)	1	Global	2	Female	22	All ages	543	Alzheimer's disease and other dement	99	Smokin	3	Rate	2001	3.468115213	5.22038539	2.058362774

									ias		g						
3	YLDs (Years Lived with Disability)	1	Global	3	Both	22	All ages	543	Alzheimer's disease and other dementias	99	Smoking	3	Rate	2001	5.199924761	7.65767793	3.148047022
3	YLDs (Years Lived with Disability)	1	Global	1	Male	22	All ages	543	Alzheimer's disease and other dementias	99	Smoking	1	Number	2002	219083.49839512	319238.124073252	133512.51547802
3	YLDs (Years Lived with Disability)	1	Global	2	Female	22	All ages	543	Alzheimer's disease and other dementias	99	Smoking	1	Number	2002	107325.019719099	161218.698672615	63692.9381731919
3	YLDs (Years Lived with Disability)	1	Global	3	Both	22	All ages	543	Alzheimer's disease and other dementias	99	Smoking	1	Number	2002	326408.518114211	480825.349221102	198075.793387865
3	YLDs (Years Lived with Disability)	1	Global	1	Male	22	All ages	543	Alzheimer's disease and other dementias	99	Smoking	3	Rate	2002	6.955876047	10.13577397	4.239007115
3	YLDs (Years Lived with Disabi	1	Global	2	Femal	22	All ages	543	Alzheimer's disease and other	99	Smoki	3	Rate	2002	3.4556750 1	5.1909557 6	2.0507994 8

	lity)				e				dementias		ng				9	3	6
3	YLDs (Years Lived with Disability)	1	Global	3	Both	22	All ages	543	Alzheimer's disease and other dementias	99	Smoking	3	Rate	2002	5.218045432	7.686590202	3.166487489
3	YLDs (Years Lived with Disability)	1	Global	1	Male	22	All ages	543	Alzheimer's disease and other dementias	99	Smoking	1	Number	2003	223445.974388201	326025.031187825	136621.003060823
3	YLDs (Years Lived with Disability)	1	Global	2	Female	22	All ages	543	Alzheimer's disease and other dementias	99	Smoking	1	Number	2003	108289.562450471	162592.028786555	64489.8004674414
3	YLDs (Years Lived with Disability)	1	Global	3	Both	22	All ages	543	Alzheimer's disease and other dementias	99	Smoking	1	Number	2003	331735.536838671	489102.412605327	201147.300723305
3	YLDs (Years Lived with Disability)	1	Global	1	Male	22	All ages	543	Alzheimer's disease and other dementias	99	Smoking	3	Rate	2003	7.00576153	10.22195019	4.28351493
3	YLDs (Years Lived with	1	Global	2	Fema	22	All ages	543	Alzheimer's disease and	99	Smok	3	Rate	2003	3.441413	5.167132	2.049468

	Disability)				le				other dementias		ing				911	057	955
3	YLDs (Years Lived with Disability)	1	Global	3	Both	22	All ages	543	Alzheimer's disease and other dementias	99	Smoking	3	Rate	2003	5.235626484	7.719274122	3.174613564
3	YLDs (Years Lived with Disability)	1	Global	1	Male	22	All ages	543	Alzheimer's disease and other dementias	99	Smoking	1	Number	2004	228027.734973684	332485.579375982	139332.380226828
3	YLDs (Years Lived with Disability)	1	Global	2	Female	22	All ages	543	Alzheimer's disease and other dementias	99	Smoking	1	Number	2004	109365.437991899	163889.050916565	65138.3299866103
3	YLDs (Years Lived with Disability)	1	Global	3	Both	22	All ages	543	Alzheimer's disease and other dementias	99	Smoking	1	Number	2004	337393.172965582	496764.014139953	204054.117835396
3	YLDs (Years Lived with Disability)	1	Global	1	Male	22	All ages	543	Alzheimer's disease and other dementias	99	Smoking	3	Rate	2004	7.059746461	10.29376489	4.31373525
3	YLDs (Years Lived	1	Global	2	Fem	22	All ag	543	Alzheimer's diseas	99	Smo	3	Rat	200	3.4301	5.1401	2.0429

	with Disability)				ale		es		e and other dementias		king		e	4	0275	63974	77827
3	YLDs (Years Lived with Disability)	1	Global	3	Both	22	All ages	543	Alzheimer's disease and other dementias	99	Smoking	3	Rate	2004	5.256678523	7.739720106	3.179219335
3	YLDs (Years Lived with Disability)	1	Global	1	Male	22	All ages	543	Alzheimer's disease and other dementias	99	Smoking	1	Number	2005	232865.826798971	340929.717453835	142097.569456133
3	YLDs (Years Lived with Disability)	1	Global	2	Female	22	All ages	543	Alzheimer's disease and other dementias	99	Smoking	1	Number	2005	110727.106481936	165911.930878202	65760.9406934172
3	YLDs (Years Lived with Disability)	1	Global	3	Both	22	All ages	543	Alzheimer's disease and other dementias	99	Smoking	1	Number	2005	343592.933280907	505569.305873717	208207.083001819
3	YLDs (Years Lived with Disability)	1	Global	1	Male	22	All ages	543	Alzheimer's disease and other dementias	99	Smoking	3	Rate	2005	7.118503353	10.42192137	4.343797622
3	YLDs (Years	1	Glob	2	Fe	22	Al l	54	Alzheimer's	99	Sm	3	Ra	20	3.42	5.13	2.03

	Lived with Disability)		al		male		ages	3	disease and other dementias		oking		te	05	6945608	4886849	5266468
3	YLDs (Years Lived with Disability)	1	Global	3	Both	22	All ages	543	Alzheimer's disease and other dementias	99	Smoking	3	Rate	2005	5.284136448	7.775180854	3.20202929
3	YLDs (Years Lived with Disability)	1	Global	1	Male	22	All ages	543	Alzheimer's disease and other dementias	99	Smoking	1	Number	2006	238710.576807876	348856.205875847	145880.033635459
3	YLDs (Years Lived with Disability)	1	Global	2	Female	22	All ages	543	Alzheimer's disease and other dementias	99	Smoking	1	Number	2006	112219.293584156	168010.806626314	66758.1839578322
3	YLDs (Years Lived with Disability)	1	Global	3	Both	22	All ages	543	Alzheimer's disease and other dementias	99	Smoking	1	Number	2006	350929.870392033	516457.059242398	212300.105638412
3	YLDs (Years Lived with Disability)	1	Global	1	Male	22	All ages	543	Alzheimer's disease and other dementias	99	Smoking	3	Rate	2006	7.203447516	10.52725607	4.402147487
3	YLDs	1	Gl	2	F	2	Al	5	Alzhei	9	S	3	R	2	3.	5.	2.

	(Years Lived with Disability)		obal		emale	2	l ages	43	mer's disease and other dementias	9	moking		ate	006	426667036	130286195	038491431
3	YLDs (Years Lived with Disability)	1	Global	3	Both	22	All ages	543	Alzheimer's disease and other dementias	99	Smoking	3	Rate	2006	5.326222492	7.838504035	3.222175406
3	YLDs (Years Lived with Disability)	1	Global	1	Male	22	All ages	543	Alzheimer's disease and other dementias	99	Smoking	1	Number	2007	245600.257427623	358951.462003401	150061.726772368
3	YLDs (Years Lived with Disability)	1	Global	2	Female	22	All ages	543	Alzheimer's disease and other dementias	99	Smoking	1	Number	2007	113729.210135209	170117.903294109	67739.0767965699
3	YLDs (Years Lived with Disability)	1	Global	3	Both	22	All ages	543	Alzheimer's disease and other dementias	99	Smoking	1	Number	2007	359329.467562831	528979.002365314	218444.664997314
3	YLDs (Years Lived with Disability)	1	Global	1	Male	22	All ages	543	Alzheimer's disease and other dementias	99	Smoking	3	Rate	2007	7.31466196	10.69057757	4.469257548

3	YLDs (Years Lived with Disability)	1	Global	2	Female	22	All ages	543	Alzheimer's disease and other dementias	99	Smoking	3	Rate	2007	3.425845098	5.124431836	2.040492358
3	YLDs (Years Lived with Disability)	1	Global	3	Both	22	All ages	543	Alzheimer's disease and other dementias	99	Smoking	3	Rate	2007	5.381290198	7.934078952	3.271410337
3	YLDs (Years Lived with Disability)	1	Global	1	Male	22	All ages	543	Alzheimer's disease and other dementias	99	Smoking	1	Number	2008	252721.95256408	368856.392197489	154749.647929904
3	YLDs (Years Lived with Disability)	1	Global	2	Female	22	All ages	543	Alzheimer's disease and other dementias	99	Smoking	1	Number	2008	115208.160514008	172719.294575625	68752.9846589581
3	YLDs (Years Lived with Disability)	1	Global	3	Both	22	All ages	543	Alzheimer's disease and other dementias	99	Smoking	1	Number	2008	367930.113078088	541047.581640984	223761.823062059
3	YLDs (Years Lived with Disability)	1	Global	1	Male	22	All ages	543	Alzheimer's disease and other dement	99	Smokin	3	Rate	2008	7.426745876	10.83959134	4.547631489

									ias		g						
3	YLDs (Years Lived with Disability)	1	Global	2	Female	22	All ages	543	Alzheimer's disease and other dementias	99	Smoking	3	Rate	2008	3.423030471	5.131784117	2.042768155
3	YLDs (Years Lived with Disability)	1	Global	3	Both	22	All ages	543	Alzheimer's disease and other dementias	99	Smoking	3	Rate	2008	5.435886037	7.99356424	3.305909809
3	YLDs (Years Lived with Disability)	1	Global	1	Male	22	All ages	543	Alzheimer's disease and other dementias	99	Smoking	1	Number	2009	260334.862992314	379943.48648427	159298.630783083
3	YLDs (Years Lived with Disability)	1	Global	2	Female	22	All ages	543	Alzheimer's disease and other dementias	99	Smoking	1	Number	2009	116782.003558241	174631.873076518	69753.5566690412
3	YLDs (Years Lived with Disability)	1	Global	3	Both	22	All ages	543	Alzheimer's disease and other dementias	99	Smoking	1	Number	2009	377116.866550555	554487.0151	229455.664966624
3	YLDs (Years Lived with Disabi	1	Global	1	Male	22	All ages	543	Alzheimer's disease and other	99	Smoki	3	Rate	2009	7.54876371	11.0169785	4.61908063

	lity)								dementias		ng				4	6	3
3	YLDs (Years Lived with Disability)	1	Global	2	Female	22	All ages	543	Alzheimer's disease and other dementias	99	Smoking	3	Rate	2009	3.422330264	5.117637359	2.044148077
3	YLDs (Years Lived with Disability)	1	Global	3	Both	22	All ages	543	Alzheimer's disease and other dementias	99	Smoking	3	Rate	2009	5.496479497	8.081649962	3.344317027
3	YLDs (Years Lived with Disability)	1	Global	1	Male	22	All ages	543	Alzheimer's disease and other dementias	99	Smoking	1	Number	2010	267790.679329265	390297.910855857	163590.44647601
3	YLDs (Years Lived with Disability)	1	Global	2	Female	22	All ages	543	Alzheimer's disease and other dementias	99	Smoking	1	Number	2010	118553.16951933	177515.20988553	70444.0605280757
3	YLDs (Years Lived with Disability)	1	Global	3	Both	22	All ages	543	Alzheimer's disease and other dementias	99	Smoking	1	Number	2010	386343.8488595	568009.31727177	234731.072412633
3	YLDs (Years Lived with	1	Global	1	Male	22	All ages	543	Alzheimer's disease and	99	Smok	3	Rate	2010	7.667187	11.17472	4.683802

	Disability)								other dementias		ing				242	486	242
3	YLDs (Years Lived with Disability)	1	Global	2	Female	22	All ages	543	Alzheimer's disease and other dementias	99	Smoking	3	Rate	2010	3.428303274	5.133358963	2.037091074
3	YLDs (Years Lived with Disability)	1	Global	3	Both	22	All ages	543	Alzheimer's disease and other dementias	99	Smoking	3	Rate	2010	5.558299696	8.171907033	3.377058163
3	YLDs (Years Lived with Disability)	1	Global	1	Male	22	All ages	543	Alzheimer's disease and other dementias	99	Smoking	1	Number	2011	275794.274292163	402550.999731329	168421.181418576
3	YLDs (Years Lived with Disability)	1	Global	2	Female	22	All ages	543	Alzheimer's disease and other dementias	99	Smoking	1	Number	2011	120347.042607549	180228.186230548	71563.2677770307
3	YLDs (Years Lived with Disability)	1	Global	3	Both	22	All ages	543	Alzheimer's disease and other dementias	99	Smoking	1	Number	2011	396141.316899712	582277.194760176	240783.281473655
3	YLDs (Years Lived	1	Global	1	Mal	22	All ag	543	Alzheimer's diseas	99	Smo	3	Rat	201	7.8013	11.386	4.7641

	with Disability)				e		es		e and other dementias		king		e	1	56237	907	0773
3	YLDs (Years Lived with Disability)	1	Global	2	Female	22	All ages	543	Alzheimer's disease and other dementias	99	Smoking	3	Rate	2011	3.43590014	5.145502846	2.043126581
3	YLDs (Years Lived with Disability)	1	Global	3	Both	22	All ages	543	Alzheimer's disease and other dementias	99	Smoking	3	Rate	2011	5.628730829	8.273516186	3.421264639
3	YLDs (Years Lived with Disability)	1	Global	1	Male	22	All ages	543	Alzheimer's disease and other dementias	99	Smoking	1	Number	2012	284807.617283586	414600.65266272	175061.740741378
3	YLDs (Years Lived with Disability)	1	Global	2	Female	22	All ages	543	Alzheimer's disease and other dementias	99	Smoking	1	Number	2012	122198.316300195	183362.755739554	72857.6159801291
3	YLDs (Years Lived with Disability)	1	Global	3	Both	22	All ages	543	Alzheimer's disease and other dementias	99	Smoking	1	Number	2012	407005.933583781	597770.535209811	248240.919376968
3	YLDs (Years	1	Glob	1	Ma	22	All	54	Alzheimer's	99	Sm	3	Ra	20	7.95	11.5	4.89

	Lived with Disability)		al		le		ages	3	disease and other dementias		oking		te	12	8480426	8533332	1812411
3	YLDs (Years Lived with Disability)	1	Global	2	Female	22	All ages	543	Alzheimer's disease and other dementias	99	Smoking	3	Rate	2012	3.444473319	5.168550099	2.053678986
3	YLDs (Years Lived with Disability)	1	Global	3	Both	22	All ages	543	Alzheimer's disease and other dementias	99	Smoking	3	Rate	2012	5.711296566	8.388194184	3.483432042
3	YLDs (Years Lived with Disability)	1	Global	1	Male	22	All ages	543	Alzheimer's disease and other dementias	99	Smoking	1	Number	2013	294084.985451841	429073.702471589	180897.42
3	YLDs (Years Lived with Disability)	1	Global	2	Female	22	All ages	543	Alzheimer's disease and other dementias	99	Smoking	1	Number	2013	123992.961776064	186426.761430667	73732.7424095866
3	YLDs (Years Lived with Disability)	1	Global	3	Both	22	All ages	543	Alzheimer's disease and other dementias	99	Smoking	1	Number	2013	418077.947227905	614676.626507164	254717.087031457
3	YLDs	1	Gl	1	M	2	Al	5	Alzhei	9	S	3	R	2	8.	11	4.

	(Years Lived with Disability)		obal		ale	2	l ages	43	mer's disease and other dementias	9	moking		ate	013	116974499	.84276816	992909592
3	YLDs (Years Lived with Disability)	1	Global	2	Female	22	All ages	543	Alzheimer's disease and other dementias	99	Smoking	3	Rate	2013	3.450832182	5.188419236	2.050246477
3	YLDs (Years Lived with Disability)	1	Global	3	Both	22	All ages	543	Alzheimer's disease and other dementias	99	Smoking	3	Rate	2013	5.793587765	8.517988108	3.529786273
3	YLDs (Years Lived with Disability)	1	Global	1	Male	22	All ages	543	Alzheimer's disease and other dementias	99	Smoking	1	Number	2014	303639.998846311	442624.635937217	186389.188992699
3	YLDs (Years Lived with Disability)	1	Global	2	Female	22	All ages	543	Alzheimer's disease and other dementias	99	Smoking	1	Number	2014	125880.92277762	189605.799646445	74741.6852653222
3	YLDs (Years Lived with Disability)	1	Global	3	Both	22	All ages	543	Alzheimer's disease and other dementias	99	Smoking	1	Number	2014	429520.92162393	631367.983574078	261635.615913513

3	YLDs (Years Lived with Disability)	1	Global	1	Male	22	All ages	543	Alzheimer's disease and other dementias	99	Smoking	3	Rate	2014	8.278732569	12.06814321	5.081893872
3	YLDs (Years Lived with Disability)	1	Global	2	Female	22	All ages	543	Alzheimer's disease and other dementias	99	Smoking	3	Rate	2014	3.459775614	5.211222698	2.054238675
3	YLDs (Years Lived with Disability)	1	Global	3	Both	22	All ages	543	Alzheimer's disease and other dementias	99	Smoking	3	Rate	2014	5.878916329	8.641626895	3.58104534
3	YLDs (Years Lived with Disability)	1	Global	1	Male	22	All ages	543	Alzheimer's disease and other dementias	99	Smoking	1	Number	2015	312399.2675136 59	454321.8543480 07	191877.9582819 16
3	YLDs (Years Lived with Disability)	1	Global	2	Female	22	All ages	543	Alzheimer's disease and other dementias	99	Smoking	1	Number	2015	127862.0087614 52	192632.0461396 5	75775.21329524 74
3	YLDs (Years Lived with Disability)	1	Global	3	Both	22	All ages	543	Alzheimer's disease and other dementias	99	Smoking	1	Number	2015	440261.2762751	648456.4699188	268047.5617436

															11	31	92
3	YLDs (Years Lived with Disability)	1	Global	1	Male	22	All ages	543	Alzheimer's disease and other dementias	99	Smoking	3	Rate	2015	8.41415964	12.236701558	5.168039558
3	YLDs (Years Lived with Disability)	1	Global	2	Female	22	All ages	543	Alzheimer's disease and other dementias	99	Smoking	3	Rate	2015	3.471086505	5.229407095	2.057079525
3	YLDs (Years Lived with Disability)	1	Global	3	Both	22	All ages	543	Alzheimer's disease and other dementias	99	Smoking	3	Rate	2015	5.952363531	8.767177244	3.624021955
3	YLDs (Years Lived with Disability)	1	Global	1	Male	22	All ages	543	Alzheimer's disease and other dementias	99	Smoking	1	Number	2016	320867.606419718	466091.615912894	195866.316334621
3	YLDs (Years Lived with Disability)	1	Global	2	Female	22	All ages	543	Alzheimer's disease and other dementias	99	Smoking	1	Number	2016	129948.748796925	195237.529065035	77297.1521902816
3	YLDs (Years Lived with Disability)	1	Global	3	Both	22	All ages	543	Alzheimer's disease and other dement	99	Smokin	1	Number	2016	450816.35521	664358.53454	273411.49932

									ias		g				6642	8353	0547
3	YLDs (Years Lived with Disability)	1	Global	1	Male	22	All ages	543	Alzheimer's disease and other dementias	99	Smoking	3	Rate	2016	8.538280684	12.402688722	5.219981818
3	YLDs (Years Lived with Disability)	1	Global	2	Female	22	All ages	543	Alzheimer's disease and other dementias	99	Smoking	3	Rate	2016	3.485166795	5.236182417	2.073074737
3	YLDs (Years Lived with Disability)	1	Global	3	Both	22	All ages	543	Alzheimer's disease and other dementias	99	Smoking	3	Rate	2016	6.021633737	8.873954369	3.652005721
3	YLDs (Years Lived with Disability)	1	Global	1	Male	22	All ages	543	Alzheimer's disease and other dementias	99	Smoking	1	Number	2017	329071.779274624	478996.121818289	201577.01779221
3	YLDs (Years Lived with Disability)	1	Global	2	Female	22	All ages	543	Alzheimer's disease and other dementias	99	Smoking	1	Number	2017	132053.172930479	198245.299231677	78461.691799129
3	YLDs (Years Lived with Disabi	1	Global	3	Both	22	All ages	543	Alzheimer's disease and other	99	Smoki	1	Numbe	2017	461124.952	679354.023	280228.089

	lity)								dementias		ng		r		205104	5	217477
3	YLDs (Years Lived with Disability)	1	Global	1	Male	22	All ages	543	Alzheimer's disease and other dementias	99	Smoking	3	Rate	2017	8.653800778	12.596452422	5.300993471
3	YLDs (Years Lived with Disability)	1	Global	2	Female	22	All ages	543	Alzheimer's disease and other dementias	99	Smoking	3	Rate	2017	3.500018772	5.254415728	2.07959709
3	YLDs (Years Lived with Disability)	1	Global	3	Both	22	All ages	543	Alzheimer's disease and other dementias	99	Smoking	3	Rate	2017	6.087012354	8.967713229	3.699109825
3	YLDs (Years Lived with Disability)	1	Global	1	Male	22	All ages	543	Alzheimer's disease and other dementias	99	Smoking	1	Number	2018	337191.208080062	492317.412762593	206438.028794088
3	YLDs (Years Lived with Disability)	1	Global	2	Female	22	All ages	543	Alzheimer's disease and other dementias	99	Smoking	1	Number	2018	134159.617762382	202310.395587457	79461.0581572402
3	YLDs (Years Lived with	1	Global	3	Both	22	All ages	543	Alzheimer's disease and	99	Smok	1	Numb	2018	471350.8	696266.6	285892.5

	Disability)								other dementias		ing		er		25842444	75334867	77029542
3	YLDs (Years Lived with Disability)	1	Global	1	Male	22	All ages	543	Alzheimer's disease and other dementias	99	Smoking	3	Rate	2018	8.76726059	12.80067495	5.367565793
3	YLDs (Years Lived with Disability)	1	Global	2	Female	22	All ages	543	Alzheimer's disease and other dementias	99	Smoking	3	Rate	2018	3.515713157	5.301634959	2.082312788
3	YLDs (Years Lived with Disability)	1	Global	3	Both	22	All ages	543	Alzheimer's disease and other dementias	99	Smoking	3	Rate	2018	6.151777074	9.087238498	3.731291651
3	YLDs (Years Lived with Disability)	1	Global	1	Male	22	All ages	543	Alzheimer's disease and other dementias	99	Smoking	1	Number	2019	346119.394117253	506959.795068534	211186.379445179
3	YLDs (Years Lived with Disability)	1	Global	2	Female	22	All ages	543	Alzheimer's disease and other dementias	99	Smoking	1	Number	2019	136383.423178174	205677.637646283	80639.9399255122
3	YLDs (Years Lived	1	Global	3	Bot	22	All ag	543	Alzheimer's diseas	99	Smo	1	Num	201	482502	713812	292747

	with Disability)				h		es		e and other dementias		king		ber	9	.817295427	.746233609	.887691553
3	YLDs (Years Lived with Disability)	1	Global	1	Male	22	All ages	543	Alzheimer's disease and other dementias	99	Smoking	3	Rate	2019	8.902796661	13.039893331	5.432083338
3	YLDs (Years Lived with Disability)	1	Global	2	Female	22	All ages	543	Alzheimer's disease and other dementias	99	Smoking	3	Rate	2019	3.535476682	5.33179528	2.090434604
3	YLDs (Years Lived with Disability)	1	Global	3	Both	22	All ages	543	Alzheimer's disease and other dementias	99	Smoking	3	Rate	2019	6.229597869	9.216042277	3.779670402
3	YLDs (Years Lived with Disability)	1	Global	1	Male	22	All ages	543	Alzheimer's disease and other dementias	99	Smoking	1	Number	2020	354849.613794671	520518.9347	216876.277030884
3	YLDs (Years Lived with Disability)	1	Global	2	Female	22	All ages	543	Alzheimer's disease and other dementias	99	Smoking	1	Number	2020	138431.117655318	208697.148568049	81813.0720739686
3	YLDs (Years	1	Glob	3	Bo	22	Al l	54	Alzhei mer's	99	Sm	1	Nu	20	4932	7277	3000

	Lived with Disability)		al		th		ages	3	disease and other dementias		oking		mber	20	80.731449989	34.884620703	47.494466936
3	YLDs (Years Lived with Disability)	1	Global	1	Male	22	All ages	543	Alzheimer’s disease and other dementias	99	Smoking	3	Rate	2020	9.0386404034	13.258527772	5.5242181812
3	YLDs (Years Lived with Disability)	1	Global	2	Female	22	All ages	543	Alzheimer’s disease and other dementias	99	Smoking	3	Rate	2020	3.552753741	5.356090363	2.099684686
3	YLDs (Years Lived with Disability)	1	Global	3	Both	22	All ages	543	Alzheimer’s disease and other dementias	99	Smoking	3	Rate	2020	6.30603167	9.303260673	3.835765076
3	YLDs (Years Lived with Disability)	1	Global	1	Male	22	All ages	543	Alzheimer’s disease and other dementias	99	Smoking	1	Number	2021	372839.779505881	545265.057554627	228453.350027475
3	YLDs (Years Lived with Disability)	1	Global	2	Female	22	All ages	543	Alzheimer’s disease and other dementias	99	Smoking	1	Number	2021	143042.405627636	215145.634379117	84581.6587929883
3	YLDs	1	Gl	3	B	2	Al	5	Alzhei	9	S	1	N	2	51	75	31

	(Years Lived with Disability)		obal		oth	2	l ages	43	mer's disease and other dementias	9	moking		umber	021	5882.185133517	9417.349430448	3106.130063068
3	YLDs (Years Lived with Disability)	1	Global	1	Male	22	All ages	543	Alzheimer's disease and other dementias	99	Smoking	3	Rate	2021	9.416593061	13.771435599	5.769910693
3	YLDs (Years Lived with Disability)	1	Global	2	Female	22	All ages	543	Alzheimer's disease and other dementias	99	Smoking	3	Rate	2021	3.637939804	5.471712136	2.151131211
3	YLDs (Years Lived with Disability)	1	Global	3	Both	22	All ages	543	Alzheimer's disease and other dementias	99	Smoking	3	Rate	2021	6.537309451	9.623410846	3.96771147
3	YLDs (Years Lived with Disability)	1	Global	1	Male	27	Age-standardized	543	Alzheimer's disease and other dementias	99	Smoking	3	Rate	1990	11.912003222	17.487528733	7.346941499
3	YLDs (Years Lived with Disability)	1	Global	2	Female	27	Age-standardized	543	Alzheimer's disease and other dementias	99	Smoking	3	Rate	1990	4.507061055	6.803061671	2.713614941

3	YLDs (Years Lived with Disability)	1	Global	3	Both	27	Age-standardized	543	Alzheimer's disease and other dementias	99	Smoking	3	Rate	1990	7.571718937	11.21947526	4.638164698
3	YLDs (Years Lived with Disability)	1	Global	1	Male	27	Age-standardized	543	Alzheimer's disease and other dementias	99	Smoking	3	Rate	1991	11.87757394	17.43749633	7.322347627
3	YLDs (Years Lived with Disability)	1	Global	2	Female	27	Age-standardized	543	Alzheimer's disease and other dementias	99	Smoking	3	Rate	1991	4.502822984	6.795838606	2.700913286
3	YLDs (Years Lived with Disability)	1	Global	3	Both	27	Age-standardized	543	Alzheimer's disease and other dementias	99	Smoking	3	Rate	1991	7.564045296	11.20831641	4.622088134
3	YLDs (Years Lived with Disability)	1	Global	1	Male	27	Age-standardized	543	Alzheimer's disease and other dementias	99	Smoking	3	Rate	1992	11.82699898	17.36321974	7.295997276
3	YLDs (Years Lived with	1	Global	2	Fema	27	Age-stan	543	Alzheimer's diseas e and	99	Smok	3	Rate	1992	4.49601 8	6.77073 5	2.69172 9

	Disability)				le		dardized		other dementias		ing				521	84	616
3	YLDs (Years Lived with Disability)	1	Global	3	Both	27	Age-standardized	543	Alzheimer's disease and other dementias	99	Smoking	3	Rate	1992	7.548696973	11.179028444	4.600494193
3	YLDs (Years Lived with Disability)	1	Global	1	Male	27	Age-standardized	543	Alzheimer's disease and other dementias	99	Smoking	3	Rate	1993	11.764116644	17.275936633	7.256865617
3	YLDs (Years Lived with Disability)	1	Global	2	Female	27	Age-standardized	543	Alzheimer's disease and other dementias	99	Smoking	3	Rate	1993	4.484135984	6.745477544	2.688089623
3	YLDs (Years Lived with Disability)	1	Global	3	Both	27	Age-standardized	543	Alzheimer's disease and other dementias	99	Smoking	3	Rate	1993	7.524948594	11.151296658	4.583848335
3	YLDs (Years Lived with Disability)	1	Global	1	Male	27	Age-standardized	543	Alzheimer's disease and other dementias	99	Smoking	3	Rate	1994	11.683156636	17.203419899	7.199400542

3	YLDs (Years Lived with Disability)	1	Global	2	Female	27	Age-standardized	543	Alzheimer's disease and other dementias	99	Smoking	3	Rate	1994	4.46373823 9	6.70865460 7	2.68772499 4
3	YLDs (Years Lived with Disability)	1	Global	3	Both	27	Age-standardized	543	Alzheimer's disease and other dementias	99	Smoking	3	Rate	1994	7.48754836 4	11.0946907 7	4.56448571 8
3	YLDs (Years Lived with Disability)	1	Global	1	Male	27	Age-standardized	543	Alzheimer's disease and other dementias	99	Smoking	3	Rate	1995	11.5851849 2	17.0567361 7	7.15221485
3	YLDs (Years Lived with Disability)	1	Global	2	Female	27	Age-standardized	543	Alzheimer's disease and other dementias	99	Smoking	3	Rate	1995	4.43954981 8	6.66386417 2	2.67592810 9
3	YLDs (Years Lived with Disability)	1	Global	3	Both	27	Age-standardized	543	Alzheimer's disease and other dementias	99	Smoking	3	Rate	1995	7.44059962 3	11.0270582 6	4.52931229 5
3	YLDs (Years Lived with	1	Global	1	Male	27	Age-st an	543	Alzheimer's diseas e and	99	Smok	3	Rate	1996	11.46102	16.83530	7.066894

	Disability)						dardized		other dementias		ing				882	178	818
3	YLDs (Years Lived with Disability)	1	Global	2	Female	27	Age-standardized	543	Alzheimer's disease and other dementias	99	Smoking	3	Rate	1996	4.402694205	6.590699795	2.645498845
3	YLDs (Years Lived with Disability)	1	Global	3	Both	27	Age-standardized	543	Alzheimer's disease and other dementias	99	Smoking	3	Rate	1996	7.374169781	10.9059241	4.480689163
3	YLDs (Years Lived with Disability)	1	Global	1	Male	27	Age-standardized	543	Alzheimer's disease and other dementias	99	Smoking	3	Rate	1997	11.31055271	16.63617023	6.972269329
3	YLDs (Years Lived with Disability)	1	Global	2	Female	27	Age-standardized	543	Alzheimer's disease and other dementias	99	Smoking	3	Rate	1997	4.353879771	6.523428951	2.609167158
3	YLDs (Years Lived with Disability)	1	Global	3	Both	27	Age-standardized	543	Alzheimer's disease and other dementias	99	Smoking	3	Rate	1997	7.288872518	10.76406815	4.419932765

3	YLDs (Years Lived with Disability)	1	Global	1	Male	27	Age-standardized	543	Alzheimer's disease and other dementias	99	Smoking	3	Rate	1998	11.146674432	16.360475573	6.879518914
3	YLDs (Years Lived with Disability)	1	Global	2	Female	27	Age-standardized	543	Alzheimer's disease and other dementias	99	Smoking	3	Rate	1998	4.294835931	6.443873204	2.568246894
3	YLDs (Years Lived with Disability)	1	Global	3	Both	27	Age-standardized	543	Alzheimer's disease and other dementias	99	Smoking	3	Rate	1998	7.190702027	10.629179973	4.36765043
3	YLDs (Years Lived with Disability)	1	Global	1	Male	27	Age-standardized	543	Alzheimer's disease and other dementias	99	Smoking	3	Rate	1999	10.995631112	16.164401186	6.77541903
3	YLDs (Years Lived with Disability)	1	Global	2	Female	27	Age-standardized	543	Alzheimer's disease and other dementias	99	Smoking	3	Rate	1999	4.228463235	6.330397328	2.523728941
3	YLDs (Years Lived with	1	Global	3	Both	27	Age-stan	543	Alzheimer's disease and	99	Smok	3	Rate	1999	7.09403 3	10.49289	4.304941

	Disability)						dardized		other dementias		ing				145	798	23
3	YLDs (Years Lived with Disability)	1	Global	1	Male	27	Age-standardized	543	Alzheimer's disease and other dementias	99	Smoking	3	Rate	2000	10.863900033	16.008471158	6.675111225
3	YLDs (Years Lived with Disability)	1	Global	2	Female	27	Age-standardized	543	Alzheimer's disease and other dementias	99	Smoking	3	Rate	2000	4.165231626	6.237811867	2.481636561
3	YLDs (Years Lived with Disability)	1	Global	3	Both	27	Age-standardized	543	Alzheimer's disease and other dementias	99	Smoking	3	Rate	2000	7.007844647	10.406359199	4.253139994
3	YLDs (Years Lived with Disability)	1	Global	1	Male	27	Age-standardized	543	Alzheimer's disease and other dementias	99	Smoking	3	Rate	2001	10.75652548	15.843639399	6.61371449
3	YLDs (Years Lived with Disability)	1	Global	2	Female	27	Age-standardized	543	Alzheimer's disease and other dementias	99	Smoking	3	Rate	2001	4.099845407	6.162752124	2.445693279

3	YLDs (Years Lived with Disability)	1	Global	3	Both	27	Age-standardized	543	Alzheimer's disease and other dementias	99	Smoking	3	Rate	2001	6.931064934	10.287303314	4.213049766
3	YLDs (Years Lived with Disability)	1	Global	1	Male	27	Age-standardized	543	Alzheimer's disease and other dementias	99	Smoking	3	Rate	2002	10.65382136	15.66137033	6.531742571
3	YLDs (Years Lived with Disability)	1	Global	2	Female	27	Age-standardized	543	Alzheimer's disease and other dementias	99	Smoking	3	Rate	2002	4.030926318	6.048973886	2.405742718
3	YLDs (Years Lived with Disability)	1	Global	3	Both	27	Age-standardized	543	Alzheimer's disease and other dementias	99	Smoking	3	Rate	2002	6.854175439	10.18361345	4.164600829
3	YLDs (Years Lived with Disability)	1	Global	1	Male	27	Age-standardized	543	Alzheimer's disease and other dementias	99	Smoking	3	Rate	2003	10.55525854	15.56173196	6.48279846
3	YLDs (Years Lived with	1	Global	2	Fema	27	Age-stan	543	Alzheimer's disease and	99	Smok	3	Rate	2003	3.961794	5.945149	2.359426

	Disability)				le		dardized		other dementias		ing				42	409	415
3	YLDs (Years Lived with Disability)	1	Global	3	Both	27	Age-standardized	543	Alzheimer's disease and other dementias	99	Smoking	3	Rate	2003	6.778634784	10.063555162	4.121754796
3	YLDs (Years Lived with Disability)	1	Global	1	Male	27	Age-standardized	543	Alzheimer's disease and other dementias	99	Smoking	3	Rate	2004	10.457773233	15.391634421	6.411846148
3	YLDs (Years Lived with Disability)	1	Global	2	Female	27	Age-standardized	543	Alzheimer's disease and other dementias	99	Smoking	3	Rate	2004	3.892997447	5.829787928	2.316798163
3	YLDs (Years Lived with Disability)	1	Global	3	Both	27	Age-standardized	543	Alzheimer's disease and other dementias	99	Smoking	3	Rate	2004	6.702927721	9.961449079	4.079361997
3	YLDs (Years Lived with Disability)	1	Global	1	Male	27	Age-standardized	543	Alzheimer's disease and other dementias	99	Smoking	3	Rate	2005	10.363469901	15.243153349	6.360716473

3	YLDs (Years Lived with Disability)	1	Global	2	Female	27	Age-standardized	543	Alzheimer's disease and other dementias	99	Smoking	3	Rate	2005	3.831920927	5.753626217	2.275500464
3	YLDs (Years Lived with Disability)	1	Global	3	Both	27	Age-standardized	543	Alzheimer's disease and other dementias	99	Smoking	3	Rate	2005	6.633485531	9.837303817	4.041940632
3	YLDs (Years Lived with Disability)	1	Global	1	Male	27	Age-standardized	543	Alzheimer's disease and other dementias	99	Smoking	3	Rate	2006	10.286786627	15.14551 71	6.300384606
3	YLDs (Years Lived with Disability)	1	Global	2	Female	27	Age-standardized	543	Alzheimer's disease and other dementias	99	Smoking	3	Rate	2006	3.769815342	5.656015374	2.241948957
3	YLDs (Years Lived with Disability)	1	Global	3	Both	27	Age-standardized	543	Alzheimer's disease and other dementias	99	Smoking	3	Rate	2006	6.572521991	9.739678391	3.99417335
3	YLDs (Years Lived with	1	Global	1	Male	27	Age-stan	543	Alzheimer's disease and	99	Smok	3	Rate	2007	10.23420	15.06270	6.285409

	Disability)						dardized		other dementias		ing				865	441	007
3	YLDs (Years Lived with Disability)	1	Global	2	Female	27	Age-standardized	543	Alzheimer's disease and other dementias	99	Smoking	3	Rate	2007	3.706214903	5.559136491	2.210529981
3	YLDs (Years Lived with Disability)	1	Global	3	Both	27	Age-standardized	543	Alzheimer's disease and other dementias	99	Smoking	3	Rate	2007	6.521954539	9.668715936	3.980606475
3	YLDs (Years Lived with Disability)	1	Global	1	Male	27	Age-standardized	543	Alzheimer's disease and other dementias	99	Smoking	3	Rate	2008	10.187080557	14.979155528	6.250027591
3	YLDs (Years Lived with Disability)	1	Global	2	Female	27	Age-standardized	543	Alzheimer's disease and other dementias	99	Smoking	3	Rate	2008	3.643291707	5.470339718	2.180483062
3	YLDs (Years Lived with Disability)	1	Global	3	Both	27	Age-standardized	543	Alzheimer's disease and other dementias	99	Smoking	3	Rate	2008	6.473897644	9.57891744	3.952161406

3	YLDs (Years Lived with Disability)	1	Global	1	Male	27	Age-standardized	543	Alzheimer's disease and other dementias	99	Smoking	3	Rate	2009	10.14868862	14.93709688	6.215679833
3	YLDs (Years Lived with Disability)	1	Global	2	Female	27	Age-standardized	543	Alzheimer's disease and other dementias	99	Smoking	3	Rate	2009	3.582121883	5.371542929	2.147126631
3	YLDs (Years Lived with Disability)	1	Global	3	Both	27	Age-standardized	543	Alzheimer's disease and other dementias	99	Smoking	3	Rate	2009	6.430413184	9.51267053	3.918539374
3	YLDs (Years Lived with Disability)	1	Global	1	Male	27	Age-standardized	543	Alzheimer's disease and other dementias	99	Smoking	3	Rate	2010	10.10627311	14.86810998	6.191351453
3	YLDs (Years Lived with Disability)	1	Global	2	Female	27	Age-standardized	543	Alzheimer's disease and other dementias	99	Smoking	3	Rate	2010	3.526998724	5.283373365	2.099709767
3	YLDs (Years Lived with	1	Global	3	Both	27	Age-st an	543	Alzheimer's diseas e and	99	Smok	3	Rate	2010	6.38734 0	9.442688	3.886024

	Disability)						dardized		other dementias		ing				632	006	215
3	YLDs (Years Lived with Disability)	1	Global	1	Male	27	Age-standardized	543	Alzheimer's disease and other dementias	99	Smoking	3	Rate	2011	10.07298845	14.79230671	6.171002311
3	YLDs (Years Lived with Disability)	1	Global	2	Female	27	Age-standardized	543	Alzheimer's disease and other dementias	99	Smoking	3	Rate	2011	3.470505623	5.20826848	2.069510955
3	YLDs (Years Lived with Disability)	1	Global	3	Both	27	Age-standardized	543	Alzheimer's disease and other dementias	99	Smoking	3	Rate	2011	6.346706669	9.368378224	3.864734731
3	YLDs (Years Lived with Disability)	1	Global	1	Male	27	Age-standardized	543	Alzheimer's disease and other dementias	99	Smoking	3	Rate	2012	10.06051629	14.79200359	6.181096843
3	YLDs (Years Lived with Disability)	1	Global	2	Female	27	Age-standardized	543	Alzheimer's disease and other dementias	99	Smoking	3	Rate	2012	3.415185854	5.128109047	2.039564812

3	YLDs (Years Lived with Disability)	1	Global	3	Both	27	Age-standardized	543	Alzheimer's disease and other dementias	99	Smoking	3	Rate	2012	6.316240234	9.332662314	3.862950966
3	YLDs (Years Lived with Disability)	1	Global	1	Male	27	Age-standardized	543	Alzheimer's disease and other dementias	99	Smoking	3	Rate	2013	10.05403786	14.77365204	6.187404052
3	YLDs (Years Lived with Disability)	1	Global	2	Female	27	Age-standardized	543	Alzheimer's disease and other dementias	99	Smoking	3	Rate	2013	3.3605673	5.058388544	2.002705598
3	YLDs (Years Lived with Disability)	1	Global	3	Both	27	Age-standardized	543	Alzheimer's disease and other dementias	99	Smoking	3	Rate	2013	6.288216784	9.286986606	3.833266642
3	YLDs (Years Lived with Disability)	1	Global	1	Male	27	Age-standardized	543	Alzheimer's disease and other dementias	99	Smoking	3	Rate	2014	10.05293726	14.79134205	6.178508895
3	YLDs (Years Lived with	1	Global	2	Fema	27	Age-stan	543	Alzheimer's diseas e and	99	Smok	3	Rate	2014	3.309056	4.980887	1.965013

	Disability)				le		dardized		other dementias		ing				252	265	041
3	YLDs (Years Lived with Disability)	1	Global	3	Both	27	Age-standardized	543	Alzheimer's disease and other dementias	99	Smoking	3	Rate	2014	6.263152351	9.262489538	3.817296008
3	YLDs (Years Lived with Disability)	1	Global	1	Male	27	Age-standardized	543	Alzheimer's disease and other dementias	99	Smoking	3	Rate	2015	10.0274757	14.77084193	6.171516883
3	YLDs (Years Lived with Disability)	1	Global	2	Female	27	Age-standardized	543	Alzheimer's disease and other dementias	99	Smoking	3	Rate	2015	3.262531271	4.91089861	1.936107865
3	YLDs (Years Lived with Disability)	1	Global	3	Both	27	Age-standardized	543	Alzheimer's disease and other dementias	99	Smoking	3	Rate	2015	6.22940084	9.237624747	3.795986721
3	YLDs (Years Lived with Disability)	1	Global	1	Male	27	Age-standardized	543	Alzheimer's disease and other dementias	99	Smoking	3	Rate	2016	9.976359784	14.72154305	6.10392983

3	YLDs (Years Lived with Disability)	1	Global	2	Female	27	Age-standardized	543	Alzheimer's disease and other dementias	99	Smoking	3	Rate	2016	3.215265055	4.832940034	1.91014892
3	YLDs (Years Lived with Disability)	1	Global	3	Both	27	Age-standardized	543	Alzheimer's disease and other dementias	99	Smoking	3	Rate	2016	6.184569661	9.174071454	3.753629787
3	YLDs (Years Lived with Disability)	1	Global	1	Male	27	Age-standardized	543	Alzheimer's disease and other dementias	99	Smoking	3	Rate	2017	9.905956441	14.59079386	6.075419532
3	YLDs (Years Lived with Disability)	1	Global	2	Female	27	Age-standardized	543	Alzheimer's disease and other dementias	99	Smoking	3	Rate	2017	3.167423876	4.761133542	1.879895667
3	YLDs (Years Lived with Disability)	1	Global	3	Both	27	Age-standardized	543	Alzheimer's disease and other dementias	99	Smoking	3	Rate	2017	6.131439066	9.088033832	3.727913544
3	YLDs (Years Lived with	1	Global	1	Male	27	Age-stan	543	Alzheimer's diseas e and	99	Smok	3	Rate	2018	9.829979	14.49934	6.023384

	Disability)						dardized		other dementias		ing				04	086	276
3	YLDs (Years Lived with Disability)	1	Global	2	Female	27	Age-standardized	543	Alzheimer's disease and other dementias	99	Smoking	3	Rate	2018	3.120485143	4.710312048	1.847208006
3	YLDs (Years Lived with Disability)	1	Global	3	Both	27	Age-standardized	543	Alzheimer's disease and other dementias	99	Smoking	3	Rate	2018	6.076855777	9.024167181	3.695162518
3	YLDs (Years Lived with Disability)	1	Global	1	Male	27	Age-standardized	543	Alzheimer's disease and other dementias	99	Smoking	3	Rate	2019	9.768996511	14.4310111	5.985789091
3	YLDs (Years Lived with Disability)	1	Global	2	Female	27	Age-standardized	543	Alzheimer's disease and other dementias	99	Smoking	3	Rate	2019	3.074926456	4.638798128	1.817395429
3	YLDs (Years Lived with Disability)	1	Global	3	Both	27	Age-standardized	543	Alzheimer's disease and other dementias	99	Smoking	3	Rate	2019	6.029500339	8.960541907	3.667807595

3	YLDs (Years Lived with Disability)	1	Global	1	Male	27	Age-standardized	543	Alzheimer's disease and other dementias	99	Smoking	3	Rate	2020	9.738808087	14.440055511	5.974669738
3	YLDs (Years Lived with Disability)	1	Global	2	Female	27	Age-standardized	543	Alzheimer's disease and other dementias	99	Smoking	3	Rate	2020	3.034431355	4.566934433	1.793894102
3	YLDs (Years Lived with Disability)	1	Global	3	Both	27	Age-standardized	543	Alzheimer's disease and other dementias	99	Smoking	3	Rate	2020	5.994738671	8.887457182	3.650051391
3	YLDs (Years Lived with Disability)	1	Global	1	Male	27	Age-standardized	543	Alzheimer's disease and other dementias	99	Smoking	3	Rate	2021	9.988922231	14.7980456	6.163652704
3	YLDs (Years Lived with Disability)	1	Global	2	Female	27	Age-standardized	543	Alzheimer's disease and other dementias	99	Smoking	3	Rate	2021	3.055892084	4.587233865	1.808099743
3	YLDs (Years Lived with	1	Global	3	Both	27	Age-stan	543	Alzheimer's diseas e and	99	Smok	3	Rate	2021	6.115306	9.031404	3.719107

	Disability)						dardized		other dementias		ing				893	679	343
4	YLLs (Years of Life Lost)	1	Global	1	Male	22	All ages	543	Alzheimer's disease and other dementias	99	Smoking	1	Number	1990	354868.250129056	1025587.01889737	80668.0274379706
4	YLLs (Years of Life Lost)	1	Global	2	Female	22	All ages	543	Alzheimer's disease and other dementias	99	Smoking	1	Number	1990	173153.25581645	486171.602013895	39948.3410440401
4	YLLs (Years of Life Lost)	1	Global	3	Both	22	All ages	543	Alzheimer's disease and other dementias	99	Smoking	1	Number	1990	528021.505945507	1511230.3461629	122674.099556672
4	YLLs (Years of Life Lost)	1	Global	1	Male	22	All ages	543	Alzheimer's disease and other dementias	99	Smoking	3	Rate	1990	13.21303821	38.18634229	3.003564642
4	YLLs (Years of Life Lost)	1	Global	2	Female	22	All ages	543	Alzheimer's disease and other dementias	99	Smoking	3	Rate	1990	6.539315553	18.36078394	1.508691284
4	YLLs (Years	1	Glob	3	Bo	22	All	54	Alzheimer's	99	Sm	3	Ra	19	9.89	28.3	2.30

	of Life Lost)		al		th		ages	3	disease and other dementias		oking		te	90	986476	34027784	00142264
4	YLLs (Years of Life Lost)	1	Global	1	Male	22	All ages	543	Alzheimer's disease and other dementias	99	Smoking	1	Number	1991	362534.8506840 98	1058943.5557473	82312.6091049241
4	YLLs (Years of Life Lost)	1	Global	2	Female	22	All ages	543	Alzheimer's disease and other dementias	99	Smoking	1	Number	1991	177538.7740492 11	500701.0491757 63	42055.2014106782
4	YLLs (Years of Life Lost)	1	Global	3	Both	22	All ages	543	Alzheimer's disease and other dementias	99	Smoking	1	Number	1991	540073.6247333 09	1548504.27170874	124875.5250069 54
4	YLLs (Years of Life Lost)	1	Global	1	Male	22	All ages	543	Alzheimer's disease and other dementias	99	Smoking	3	Rate	1991	13.28662317	38.80946605	3.016693752
4	YLLs (Years of Life Lost)	1	Global	2	Female	22	All ages	543	Alzheimer's disease and other dementias	99	Smoking	3	Rate	1991	6.60500357	18.62766168	1.564586423
4	YLLs	1	Gl	3	B	2	Al	5	Alzhei	9	S	3	R	1	9.	28	2.

	(Years of Life Lost)		obal		oth	2	l ages	43	mer's disease and other dementias	9	moking		ate	991	970871153	.58857731	305459317
4	YLLs (Years of Life Lost)	1	Global	1	Male	22	All ages	543	Alzheimer's disease and other dementias	99	Smoking	1	Number	1992	369362.309906562	1073374.43061529	84386.2958053892
4	YLLs (Years of Life Lost)	1	Global	2	Female	22	All ages	543	Alzheimer's disease and other dementias	99	Smoking	1	Number	1992	181593.303502216	513989.365160238	42901.8201546411
4	YLLs (Years of Life Lost)	1	Global	3	Both	22	All ages	543	Alzheimer's disease and other dementias	99	Smoking	1	Number	1992	550955.613408778	1579160.44221039	129801.296305164
4	YLLs (Years of Life Lost)	1	Global	1	Male	22	All ages	543	Alzheimer's disease and other dementias	99	Smoking	3	Rate	1992	13.33369814	38.74799963	3.046281023
4	YLLs (Years of Life Lost)	1	Global	2	Female	22	All ages	543	Alzheimer's disease and other dementias	99	Smoking	3	Rate	1992	6.658925571	18.84770452	1.573185915

4	YLLs (Years of Life Lost)	1	Global	3	Both	22	All ages	543	Alzheimer's disease and other dementias	99	Smoking	3	Rate	1992	10.02246318	28.7265925	2.361222359
4	YLLs (Years of Life Lost)	1	Global	1	Male	22	All ages	543	Alzheimer's disease and other dementias	99	Smoking	1	Number	1993	376447.558993669	1094348.45579696	84970.8444957099
4	YLLs (Years of Life Lost)	1	Global	2	Female	22	All ages	543	Alzheimer's disease and other dementias	99	Smoking	1	Number	1993	185758.983095878	519575.861393245	43010.3942343044
4	YLLs (Years of Life Lost)	1	Global	3	Both	22	All ages	543	Alzheimer's disease and other dementias	99	Smoking	1	Number	1993	562206.542089547	1613924.3171902	129910.121393019
4	YLLs (Years of Life Lost)	1	Global	1	Male	22	All ages	543	Alzheimer's disease and other dementias	99	Smoking	3	Rate	1993	13.39571294	38.94188558	3.023648351
4	YLLs (Years of Life Lost)	1	Global	2	Female	22	All ages	543	Alzheimer's disease and other dement	99	Smokin	3	Rate	1993	6.717597961	18.78941028	1.555383927

									ias		g						
4	YLLs (Years of Life Lost)	1	Global	3	Both	22	All ages	543	Alzheimer’s disease and other dementias	99	Smoking	3	Rate	1993	10.083575399	28.946883396	2.330030701
4	YLLs (Years of Life Lost)	1	Global	1	Male	22	All ages	543	Alzheimer’s disease and other dementias	99	Smoking	1	Number	1994	382411.715252826	1105542.94241502	88128.3377979985
4	YLLs (Years of Life Lost)	1	Global	2	Female	22	All ages	543	Alzheimer’s disease and other dementias	99	Smoking	1	Number	1994	189539.514267503	533312.695963875	44243.7934178101
4	YLLs (Years of Life Lost)	1	Global	3	Both	22	All ages	543	Alzheimer’s disease and other dementias	99	Smoking	1	Number	1994	571951.229520329	1634438.55323659	134056.647219264
4	YLLs (Years of Life Lost)	1	Global	1	Male	22	All ages	543	Alzheimer’s disease and other dementias	99	Smoking	3	Rate	1994	13.42616261	38.81470868	3.094113875
4	YLLs (Years of Life Lost)	1	Global	2	Femal	22	All ages	543	Alzheimer’s disease and other	99	Smoki	3	Rate	1994	6.76395049	19.0319189	1.57889414

					e				dementias		ng				4	5	
4	YLLs (Years of Life Lost)	1	Global	3	Both	22	All ages	543	Alzheimer's disease and other dementias	99	Smoking	3	Rate	1994	10.12220808	28.92576548	2.372491233
4	YLLs (Years of Life Lost)	1	Global	1	Male	22	All ages	543	Alzheimer's disease and other dementias	99	Smoking	1	Number	1995	388502.027519121	1132206.02041881	86934.0096889058
4	YLLs (Years of Life Lost)	1	Global	2	Female	22	All ages	543	Alzheimer's disease and other dementias	99	Smoking	1	Number	1995	193246.357580793	534897.799000578	45089.920118686
4	YLLs (Years of Life Lost)	1	Global	3	Both	22	All ages	543	Alzheimer's disease and other dementias	99	Smoking	1	Number	1995	581748.385099915	1673274.85833363	133687.496453992
4	YLLs (Years of Life Lost)	1	Global	1	Male	22	All ages	543	Alzheimer's disease and other dementias	99	Smoking	3	Rate	1995	13.46432313	39.23888841	3.012873844
4	YLLs (Years of Life	1	Global	2	Fema	22	All ages	543	Alzheimer's diseas e and	99	Smok	3	Rate	1995	6.807701	18.84343	1.588432

	Lost)				le				other dementias		ing				919	29	194
4	YLLs (Years of Life Lost)	1	Global	3	Both	22	All ages	543	Alzheimer's disease and other dementias	99	Smoking	3	Rate	1995	10.16321037	29.23230186	2.33553575
4	YLLs (Years of Life Lost)	1	Global	1	Male	22	All ages	543	Alzheimer's disease and other dementias	99	Smoking	1	Number	1996	394158.743347333	1145762.46953958	89712.8913854666
4	YLLs (Years of Life Lost)	1	Global	2	Female	22	All ages	543	Alzheimer's disease and other dementias	99	Smoking	1	Number	1996	196196.595177363	553924.626070386	46046.4473647575
4	YLLs (Years of Life Lost)	1	Global	3	Both	22	All ages	543	Alzheimer's disease and other dementias	99	Smoking	1	Number	1996	590355.338524696	1700923.26958335	138500.324872458
4	YLLs (Years of Life Lost)	1	Global	1	Male	22	All ages	543	Alzheimer's disease and other dementias	99	Smoking	3	Rate	1996	13.48572395	39.20104942	3.069431564
4	YLLs (Years of	1	Global	2	Fem	22	All ag	543	Alzheimer's diseas	99	Smo	3	Rat	199	6.8227	19.262	1.6012

	Life Lost)				ale		es		e and other dementias		king		e	6	83887	86239	76306
4	YLLs (Years of Life Lost)	1	Global	3	Both	22	All ages	543	Alzheimer's disease and other dementias	99	Smoking	3	Rate	1996	10.181358966	29.334384299	2.388597903
4	YLLs (Years of Life Lost)	1	Global	1	Male	22	All ages	543	Alzheimer's disease and other dementias	99	Smoking	1	Number	1997	398542.822989665	1175020.7913656	93718.6254396227
4	YLLs (Years of Life Lost)	1	Global	2	Female	22	All ages	543	Alzheimer's disease and other dementias	99	Smoking	1	Number	1997	198189.947469281	550865.209560767	46552.1260696247
4	YLLs (Years of Life Lost)	1	Global	3	Both	22	All ages	543	Alzheimer's disease and other dementias	99	Smoking	1	Number	1997	596732.770458946	1729034.47887663	140847.467741027
4	YLLs (Years of Life Lost)	1	Global	1	Male	22	All ages	543	Alzheimer's disease and other dementias	99	Smoking	3	Rate	1997	13.463848489	39.695352099	3.166066389
4	YLLs (Years	1	Glob	2	Fe	22	Al l	54	Alzheimer's	99	Sm	3	Ra	19	6.80	18.9	1.59

	of Life Lost)		al		male		ages	3	disease and other dementias		oking		te	97	3684453	1071222 2	8093044
4	YLLs (Years of Life Lost)	1	Global	3	Both	22	All ages	543	Alzheimer's disease and other dementias	99	Smoking	3	Rate	1997	10.16048035	29.44001356	2.398188938
4	YLLs (Years of Life Lost)	1	Global	1	Male	22	All ages	543	Alzheimer's disease and other dementias	99	Smoking	1	Number	1998	402795.134421744	1167267.97221621	94221.945076736
4	YLLs (Years of Life Lost)	1	Global	2	Female	22	All ages	543	Alzheimer's disease and other dementias	99	Smoking	1	Number	1998	200058.136559824	555981.509267794	47620.3349799558
4	YLLs (Years of Life Lost)	1	Global	3	Both	22	All ages	543	Alzheimer's disease and other dementias	99	Smoking	1	Number	1998	602853.270981569	1727663.45495727	141872.020195591
4	YLLs (Years of Life Lost)	1	Global	1	Male	22	All ages	543	Alzheimer's disease and other dementias	99	Smoking	3	Rate	1998	13.43836635	38.94330714	3.143506233
4	YLLs	1	Gl	2	F	2	Al	5	Alzhei	9	S	3	R	1	6.	18	1.

	(Years of Life Lost)		obal		emale	2	l ages	43	mer's disease and other dementias	9	moking		ate	998	780413047	.84344393	613958553
4	YLLs (Years of Life Lost)	1	Global	3	Both	22	All ages	543	Alzheimer's disease and other dementias	99	Smoking	3	Rate	1998	10.135595539	29.046699941	2.385252703
4	YLLs (Years of Life Lost)	1	Global	1	Male	22	All ages	543	Alzheimer's disease and other dementias	99	Smoking	1	Number	1999	407564.909558947	1190982.16830838	94730.0590603323
4	YLLs (Years of Life Lost)	1	Global	2	Female	22	All ages	543	Alzheimer's disease and other dementias	99	Smoking	1	Number	1999	201984.589889595	565752.766768349	48297.9553438577
4	YLLs (Years of Life Lost)	1	Global	3	Both	22	All ages	543	Alzheimer's disease and other dementias	99	Smoking	1	Number	1999	609549.499448542	1759477.34022068	143387.106426777
4	YLLs (Years of Life Lost)	1	Global	1	Male	22	All ages	543	Alzheimer's disease and other dementias	99	Smoking	3	Rate	1999	13.430625598	39.246843307	3.121672063

4	YLLs (Years of Life Lost)	1	Global	2	Female	22	All ages	543	Alzheimer's disease and other dementias	99	Smoking	3	Rate	1999	6.759202197	18.93232224	1.616240358
4	YLLs (Years of Life Lost)	1	Global	3	Both	22	All ages	543	Alzheimer's disease and other dementias	99	Smoking	3	Rate	1999	10.12055861	29.21320346	2.38070512
4	YLLs (Years of Life Lost)	1	Global	1	Male	22	All ages	543	Alzheimer's disease and other dementias	99	Smoking	1	Number	2000	414497.587380641	1215060.05049469	96317.8961051661
4	YLLs (Years of Life Lost)	1	Global	2	Female	22	All ages	543	Alzheimer's disease and other dementias	99	Smoking	1	Number	2000	204125.636248112	571326.137527568	48717.0511898569
4	YLLs (Years of Life Lost)	1	Global	3	Both	22	All ages	543	Alzheimer's disease and other dementias	99	Smoking	1	Number	2000	618623.223628753	1781128.96347499	145608.519690805
4	YLLs (Years of Life Lost)	1	Global	1	Male	22	All ages	543	Alzheimer's disease and other dement	99	Smokin	3	Rate	2000	13.49192976	39.55030224	3.135155255

									ias		g						
4	YLLs (Years of Life Lost)	1	Global	2	Female	22	All ages	543	Alzheimer's disease and other dementias	99	Smoking	3	Rate	2000	6.744412127	18.876898857	1.609635501
4	YLLs (Years of Life Lost)	1	Global	3	Both	22	All ages	543	Alzheimer's disease and other dementias	99	Smoking	3	Rate	2000	10.143396244	29.204685744	2.387503177
4	YLLs (Years of Life Lost)	1	Global	1	Male	22	All ages	543	Alzheimer's disease and other dementias	99	Smoking	1	Number	2001	421957.878241643	1210924.80929446	97910.6134463309
4	YLLs (Years of Life Lost)	1	Global	2	Female	22	All ages	543	Alzheimer's disease and other dementias	99	Smoking	1	Number	2001	206439.174401325	579392.874801764	49305.8704903115
4	YLLs (Years of Life Lost)	1	Global	3	Both	22	All ages	543	Alzheimer's disease and other dementias	99	Smoking	1	Number	2001	628397.052642968	1784519.90252927	147545.915790333
4	YLLs (Years of Life Lost)	1	Global	1	Male	22	All ages	543	Alzheimer's disease and other	99	Smoki	3	Rate	2001	13.5656726	38.9304487	3.14776283

									dementias		ng				2		8
4	YLLs (Years of Life Lost)	1	Global	2	Female	22	All ages	543	Alzheimer's disease and other dementias	99	Smoking	3	Rate	2001	6.733790111	18.89907777	1.60829641
4	YLLs (Years of Life Lost)	1	Global	3	Both	22	All ages	543	Alzheimer's disease and other dementias	99	Smoking	3	Rate	2001	10.17448848	28.89347924	2.388942174
4	YLLs (Years of Life Lost)	1	Global	1	Male	22	All ages	543	Alzheimer's disease and other dementias	99	Smoking	1	Number	2002	431241.837278545	1268815.16263568	101948.162595611
4	YLLs (Years of Life Lost)	1	Global	2	Female	22	All ages	543	Alzheimer's disease and other dementias	99	Smoking	1	Number	2002	208671.972296761	583329.937750547	50152.6420443703
4	YLLs (Years of Life Lost)	1	Global	3	Both	22	All ages	543	Alzheimer's disease and other dementias	99	Smoking	1	Number	2002	639913.809575306	1849831.85555954	150403.137583641
4	YLLs (Years of Life	1	Global	1	Male	22	All ages	543	Alzheimer's disease and	99	Smok	3	Rate	2002	13.69187	40.28473	3.236842

	Lost)								other dementias		ing				907	647	516
4	YLLs (Years of Life Lost)	1	Global	2	Female	22	All ages	543	Alzheimer's disease and other dementias	99	Smoking	3	Rate	2002	6.718866893	18.782187796	1.614826
4	YLLs (Years of Life Lost)	1	Global	3	Both	22	All ages	543	Alzheimer's disease and other dementias	99	Smoking	3	Rate	2002	10.22981677	29.57185897	2.404380895
4	YLLs (Years of Life Lost)	1	Global	1	Male	22	All ages	543	Alzheimer's disease and other dementias	99	Smoking	1	Number	2003	439642.279030179	1262866.58329104	102782.686872491
4	YLLs (Years of Life Lost)	1	Global	2	Female	22	All ages	543	Alzheimer's disease and other dementias	99	Smoking	1	Number	2003	210783.764735957	592622.256786188	50960.8983912519
4	YLLs (Years of Life Lost)	1	Global	3	Both	22	All ages	543	Alzheimer's disease and other dementias	99	Smoking	1	Number	2003	650426.043766137	1854038.01259653	154144.384639125
4	YLLs (Years of	1	Global	1	Mal	22	All ag	543	Alzheimer's diseas	99	Smo	3	Rat	20	13.784	39.594	3.2225

	Life Lost)				e		es		e and other dementias		king		e	3	22222	9945	73132
4	YLLs (Years of Life Lost)	1	Global	2	Female	22	All ages	543	Alzheimer's disease and other dementias	99	Smoking	3	Rate	2003	6.698652794	18.833379999	1.619523993
4	YLLs (Years of Life Lost)	1	Global	3	Both	22	All ages	543	Alzheimer's disease and other dementias	99	Smoking	3	Rate	2003	10.26536938	29.26141291	2.432788571
4	YLLs (Years of Life Lost)	1	Global	1	Male	22	All ages	543	Alzheimer's disease and other dementias	99	Smoking	1	Number	2004	448525.131862606	1286948.19449314	104891.862960522
4	YLLs (Years of Life Lost)	1	Global	2	Female	22	All ages	543	Alzheimer's disease and other dementias	99	Smoking	1	Number	2004	212491.12821054	594946.448514911	51059.8961528333
4	YLLs (Years of Life Lost)	1	Global	3	Both	22	All ages	543	Alzheimer's disease and other dementias	99	Smoking	1	Number	2004	661016.260073147	1878256.01449931	156046.611671514
4	YLLs (Years	1	Glob	1	Ma	22	Al	54	Alzheimer's	99	Sm	3	Ra	20	13.8	39.8	3.24

	of Life Lost)		al		le		ages	3	disease and other dementias		oking		te	04	863534	4396005	7455659
4	YLLs (Years of Life Lost)	1	Global	2	Female	22	All ages	543	Alzheimer's disease and other dementias	99	Smoking	3	Rate	2004	6.66450404	18.65971085	1.601426314
4	YLLs (Years of Life Lost)	1	Global	3	Both	22	All ages	543	Alzheimer's disease and other dementias	99	Smoking	3	Rate	2004	10.29881532	29.26374581	2.431249171
4	YLLs (Years of Life Lost)	1	Global	1	Male	22	All ages	543	Alzheimer's disease and other dementias	99	Smoking	1	Number	2005	458650.59200909	1313139.77365144	105978.22020364 9
4	YLLs (Years of Life Lost)	1	Global	2	Female	22	All ages	543	Alzheimer's disease and other dementias	99	Smoking	1	Number	2005	215599.193454094	607596.110051746	51579.2318827507
4	YLLs (Years of Life Lost)	1	Global	3	Both	22	All ages	543	Alzheimer's disease and other dementias	99	Smoking	1	Number	2005	674249.785455003	1911297.93591526	158163.427219473
4	YLLs	1	Gl	1	M	2	Al	5	Alzhei	9	S	3	R	2	14	40	3.

	(Years of Life Lost)		obal		ale	2	l ages	43	mer's disease and other dementias	9	moking		ate	005	.02054489	.14152703	239660908
4	YLLs (Years of Life Lost)	1	Global	2	Female	22	All ages	543	Alzheimer's disease and other dementias	99	Smoking	3	Rate	2005	6.672681448	18.804777949	1.596350052
4	YLLs (Years of Life Lost)	1	Global	3	Both	22	All ages	543	Alzheimer's disease and other dementias	99	Smoking	3	Rate	2005	10.36932813	29.39396626	2.432404889
4	YLLs (Years of Life Lost)	1	Global	1	Male	22	All ages	543	Alzheimer's disease and other dementias	99	Smoking	1	Number	2006	469532.977704955	1346767.53652839	110490.391988813
4	YLLs (Years of Life Lost)	1	Global	2	Female	22	All ages	543	Alzheimer's disease and other dementias	99	Smoking	1	Number	2006	218318.356936231	611704.474359793	52361.6811293229
4	YLLs (Years of Life Lost)	1	Global	3	Both	22	All ages	543	Alzheimer's disease and other dementias	99	Smoking	1	Number	2006	687851.334641185	1946760.682	162782.833012126

4	YLLs (Years of Life Lost)	1	Global	1	Male	22	All ages	543	Alzheimer's disease and other dementias	99	Smoking	3	Rate	2006	14.16885756	40.64071812	3.334212293
4	YLLs (Years of Life Lost)	1	Global	2	Female	22	All ages	543	Alzheimer's disease and other dementias	99	Smoking	3	Rate	2006	6.666450065	18.67867361	1.598887686
4	YLLs (Years of Life Lost)	1	Global	3	Both	22	All ages	543	Alzheimer's disease and other dementias	99	Smoking	3	Rate	2006	10.43983302	29.54687362	2.470629204
4	YLLs (Years of Life Lost)	1	Global	1	Male	22	All ages	543	Alzheimer's disease and other dementias	99	Smoking	1	Number	2007	483662.250181641	1394154.91325329	113910.535183317
4	YLLs (Years of Life Lost)	1	Global	2	Female	22	All ages	543	Alzheimer's disease and other dementias	99	Smoking	1	Number	2007	221402.594345088	616906.032678565	53710.8232536543
4	YLLs (Years of Life Lost)	1	Global	3	Both	22	All ages	543	Alzheimer's disease and other dementias	99	Smoking	1	Number	2007	705064.8445267	2010118.546429	167664.8874

															29	45	
4	YLLs (Years of Life Lost)	1	Global	1	Male	22	All ages	543	Alzheimer's disease and other dementias	99	Smoking	3	Rate	2007	14.40481333	41.52182907	3.392574044
4	YLLs (Years of Life Lost)	1	Global	2	Female	22	All ages	543	Alzheimer's disease and other dementias	99	Smoking	3	Rate	2007	6.669271611	18.58295248	1.617921731
4	YLLs (Years of Life Lost)	1	Global	3	Both	22	All ages	543	Alzheimer's disease and other dementias	99	Smoking	3	Rate	2007	10.55899635	30.10337923	2.510936331
4	YLLs (Years of Life Lost)	1	Global	1	Male	22	All ages	543	Alzheimer's disease and other dementias	99	Smoking	1	Number	2008	498569.76812489	1449098.85274737	116237.849859066
4	YLLs (Years of Life Lost)	1	Global	2	Female	22	All ages	543	Alzheimer's disease and other dementias	99	Smoking	1	Number	2008	224781.361418153	614236.281582911	53994.406417121
4	YLLs (Years of Life Lost)	1	Global	3	Both	22	All ages	543	Alzheimer's disease and other dement	99	Smokin	1	Number	2008	723351.13823	2070756.5595	171857.39209

									ias		g				0642	1065	763
4	YLLs (Years of Life Lost)	1	Global	1	Male	22	All ages	543	Alzheimer's disease and other dementias	99	Smoking	3	Rate	2008	14.65148158	42.58470156	3.415884387
4	YLLs (Years of Life Lost)	1	Global	2	Female	22	All ages	543	Alzheimer's disease and other dementias	99	Smoking	3	Rate	2008	6.678636704	18.25000502	1.604265684
4	YLLs (Years of Life Lost)	1	Global	3	Both	22	All ages	543	Alzheimer's disease and other dementias	99	Smoking	3	Rate	2008	10.68695987	30.59384451	2.539061536
4	YLLs (Years of Life Lost)	1	Global	1	Male	22	All ages	543	Alzheimer's disease and other dementias	99	Smoking	1	Number	2009	514699.898660801	1463046.78198537	120108.171479055
4	YLLs (Years of Life Lost)	1	Global	2	Female	22	All ages	543	Alzheimer's disease and other dementias	99	Smoking	1	Number	2009	228073.276940588	628972.242888559	55218.3006525966
4	YLLs (Years of Life Lost)	1	Global	3	Both	22	All ages	543	Alzheimer's disease and other	99	Smoki	1	Numbe	2009	742773.175	2099616.14	175792.716

									dementias		ng		r		601389	495558	312425
4	YLLs (Years of Life Lost)	1	Global	1	Male	22	All ages	543	Alzheimer's disease and other dementias	99	Smoking	3	Rate	2009	14.92442416	42.42303291	3.48269992
4	YLLs (Years of Life Lost)	1	Global	2	Female	22	All ages	543	Alzheimer's disease and other dementias	99	Smoking	3	Rate	2009	6.683753097	18.43221281	1.618188211
4	YLLs (Years of Life Lost)	1	Global	3	Both	22	All ages	543	Alzheimer's disease and other dementias	99	Smoking	3	Rate	2009	10.82592133	30.60191181	2.562179384
4	YLLs (Years of Life Lost)	1	Global	1	Male	22	All ages	543	Alzheimer's disease and other dementias	99	Smoking	1	Number	2010	530314.5962	1517138.81775184	125301.5926921558
4	YLLs (Years of Life Lost)	1	Global	2	Female	22	All ages	543	Alzheimer's disease and other dementias	99	Smoking	1	Number	2010	232171.9075366664	638367.0332913335	56044.2724937728
4	YLLs (Years of Life	1	Global	3	Both	22	All ages	543	Alzheimer's diseas e and	99	Smok	1	Numb	2010	762486.5	2169141.	181760.4

	Lost)								other dementias		ing		er		03691764	8589822	73661224
4	YLLs (Years of Life Lost)	1	Global	1	Male	22	All ages	543	Alzheimer's disease and other dementias	99	Smoking	3	Rate	2010	15.18358038	43.43761111	3.587543731
4	YLLs (Years of Life Lost)	1	Global	2	Female	22	All ages	543	Alzheimer's disease and other dementias	99	Smoking	3	Rate	2010	6.713913378	18.46020481	1.620680102
4	YLLs (Years of Life Lost)	1	Global	3	Both	22	All ages	543	Alzheimer's disease and other dementias	99	Smoking	3	Rate	2010	10.96983559	31.20727966	2.61497417
4	YLLs (Years of Life Lost)	1	Global	1	Male	22	All ages	543	Alzheimer's disease and other dementias	99	Smoking	1	Number	2011	546369.083273788	1599243.40727007	129456.592278832
4	YLLs (Years of Life Lost)	1	Global	2	Female	22	All ages	543	Alzheimer's disease and other dementias	99	Smoking	1	Number	2011	236080.833900419	656865.398975586	56498.1149656496
4	YLLs (Years of	1	Global	3	Bot	22	All ag	543	Alzheimer's diseas	99	Smo	1	Num	201	782449	226588	186402

	Life Lost)				h		es		e and other dementias		king		ber	1	.917174207	5.4369531	.866392828
4	YLLs (Years of Life Lost)	1	Global	1	Male	22	All ages	543	Alzheimer's disease and other dementias	99	Smoking	3	Rate	2011	15.45507015	45.23758719	3.66192154
4	YLLs (Years of Life Lost)	1	Global	2	Female	22	All ages	543	Alzheimer's disease and other dementias	99	Smoking	3	Rate	2011	6.740092258	18.75346387	1.613017461
4	YLLs (Years of Life Lost)	1	Global	3	Both	22	All ages	543	Alzheimer's disease and other dementias	99	Smoking	3	Rate	2011	11.11774961	32.19573084	2.648578969
4	YLLs (Years of Life Lost)	1	Global	1	Male	22	All ages	543	Alzheimer's disease and other dementias	99	Smoking	1	Number	2012	563360.43607441	1649013.86868367	131986.582571387
4	YLLs (Years of Life Lost)	1	Global	2	Female	22	All ages	543	Alzheimer's disease and other dementias	99	Smoking	1	Number	2012	239791.154146577	654440.994147775	57817.7734587541
4	YLLs (Years	1	Glob	3	Bo	22	Al l	54	Alzhei mer's	99	Sm	1	Nu	20	8031	2308	1877

	of Life Lost)		al		th		ages	3	disease and other dementias		oking		mber	12	51.590220988	152.39767296	84.691289714
4	YLLs (Years of Life Lost)	1	Global	1	Male	22	All ages	543	Alzheimer's disease and other dementias	99	Smoking	3	Rate	2012	15.7421808	46.07898034	3.688147964
4	YLLs (Years of Life Lost)	1	Global	2	Female	22	All ages	543	Alzheimer's disease and other dementias	99	Smoking	3	Rate	2012	6.759129401	18.447099985	1.629742406
4	YLLs (Years of Life Lost)	1	Global	3	Both	22	All ages	543	Alzheimer's disease and other dementias	99	Smoking	3	Rate	2012	11.27019667	32.38906801	2.63508213
4	YLLs (Years of Life Lost)	1	Global	1	Male	22	All ages	543	Alzheimer's disease and other dementias	99	Smoking	1	Number	2013	580720.53864597	1703447.80335778	136643.50651323
4	YLLs (Years of Life Lost)	1	Global	2	Female	22	All ages	543	Alzheimer's disease and other dementias	99	Smoking	1	Number	2013	243227.357792876	670117.920786384	57711.1507929198
4	YLLs	1	Gl	3	B	2	Al	5	Alzhei	9	S	1	N	2	82	23	19

	(Years of Life Lost)		obal		oth	2	l ages	43	mer's disease and other dementias	9	moking		umber	013	3947.896438846	86148.17361457	5346.753975014
4	YLLs (Years of Life Lost)	1	Global	1	Male	22	All ages	543	Alzheimer's disease and other dementias	99	Smoking	3	Rate	2013	16.02833887	47.01648525	3.771467136
4	YLLs (Years of Life Lost)	1	Global	2	Female	22	All ages	543	Alzheimer's disease and other dementias	99	Smoking	3	Rate	2013	6.769229333	18.64996572	1.60615162
4	YLLs (Years of Life Lost)	1	Global	3	Both	22	All ages	543	Alzheimer's disease and other dementias	99	Smoking	3	Rate	2013	11.41800108	33.06646274	2.707051571
4	YLLs (Years of Life Lost)	1	Global	1	Male	22	All ages	543	Alzheimer's disease and other dementias	99	Smoking	1	Number	2014	598853.275184662	1723156.80223935	141898.147522518
4	YLLs (Years of Life Lost)	1	Global	2	Female	22	All ages	543	Alzheimer's disease and other dementias	99	Smoking	1	Number	2014	247091.788708928	690615.280646683	58299.3427776339

4	YLLs (Years of Life Lost)	1	Global	3	Both	22	All ages	543	Alzheimer's disease and other dementias	99	Smoking	1	Number	2014	845945.0638935 9	2399935.1477851 5	199558.44013812 2
4	YLLs (Years of Life Lost)	1	Global	1	Male	22	All ages	543	Alzheimer's disease and other dementias	99	Smoking	3	Rate	2014	16.3277108 8	46.9818021 1	3.8688473 84
4	YLLs (Years of Life Lost)	1	Global	2	Female	22	All ages	543	Alzheimer's disease and other dementias	99	Smoking	3	Rate	2014	6.791197	18.9812233 2	1.6023289 31
4	YLLs (Years of Life Lost)	1	Global	3	Both	22	All ages	543	Alzheimer's disease and other dementias	99	Smoking	3	Rate	2014	11.5785751	32.8482670 3	2.7313858 61
4	YLLs (Years of Life Lost)	1	Global	1	Male	22	All ages	543	Alzheimer's disease and other dementias	99	Smoking	1	Number	2015	617393.2263535 9	1738413.0334262 5	145003.6519898 36
4	YLLs (Years of Life Lost)	1	Global	2	Female	22	All ages	543	Alzheimer's disease and other dementias	99	Smoking	1	Number	2015	251606.4029367	704421.7016557	59220.25585814

4	YLLs (Years of Life Lost)	1	Global	3	Both	22	All ages	543	Alzheimer's disease and other dementias	99	Smoking	1	Number	2015	89868999.629572148	122455872.0710494	94205361.428657442
4	YLLs (Years of Life Lost)	1	Global	1	Male	22	All ages	543	Alzheimer's disease and other dementias	99	Smoking	3	Rate	2015	16.62886476	46.82240423	3.905527327
4	YLLs (Years of Life Lost)	1	Global	2	Female	22	All ages	543	Alzheimer's disease and other dementias	99	Smoking	3	Rate	2015	6.830391594	19.12302713	1.607659953
4	YLLs (Years of Life Lost)	1	Global	3	Both	22	All ages	543	Alzheimer's disease and other dementias	99	Smoking	3	Rate	2015	11.74893633	33.20356375	2.776501011
4	YLLs (Years of Life Lost)	1	Global	1	Male	22	All ages	543	Alzheimer's disease and other dementias	99	Smoking	1	Number	2016	635587.966564491	1815053.64783443	151796.728151721
4	YLLs (Years of Life Lost)	1	Global	2	Female	22	All ages	543	Alzheimer's disease and other dement	99	Smokin	1	Number	2016	256506.90314	713955.80934	60700.282440

									ias		g				06	59	56
4	YLLs (Years of Life Lost)	1	Global	3	Both	22	All ages	543	Alzheimer's disease and other dementias	99	Smoking	1	Number	2016	88892094.869705179	152535465.38872226	79210242.907917458
4	YLLs (Years of Life Lost)	1	Global	1	Male	22	All ages	543	Alzheimer's disease and other dementias	99	Smoking	3	Rate	2016	16.91298327	48.2985418	4.039307945
4	YLLs (Years of Life Lost)	1	Global	2	Female	22	All ages	543	Alzheimer's disease and other dementias	99	Smoking	3	Rate	2016	6.879399378	19.14797259	1.62795418
4	YLLs (Years of Life Lost)	1	Global	3	Both	22	All ages	543	Alzheimer's disease and other dementias	99	Smoking	3	Rate	2016	11.91586885	33.86665933	2.808251681
4	YLLs (Years of Life Lost)	1	Global	1	Male	22	All ages	543	Alzheimer's disease and other dementias	99	Smoking	1	Number	2017	655343.091558643	1884726.08058334	158329.743930258
4	YLLs (Years of Life Lost)	1	Global	2	Femal	22	All ages	543	Alzheimer's disease and other	99	Smoki	1	Numbe	2017	260919.135	730584.888	61994.3234

					e				dementias		ng		r		943451	580568	998125
4	YLLs (Years of Life Lost)	1	Global	3	Both	22	All ages	543	Alzheimer's disease and other dementias	99	Smoking	1	Number	2017	916262.227502093	2613344.58859202	222656.595386433
4	YLLs (Years of Life Lost)	1	Global	1	Male	22	All ages	543	Alzheimer's disease and other dementias	99	Smoking	3	Rate	2017	17.233955597	49.563788366	4.1636936
4	YLLs (Years of Life Lost)	1	Global	2	Female	22	All ages	543	Alzheimer's disease and other dementias	99	Smoking	3	Rate	2017	6.915561766	19.36387266	1.643135801
4	YLLs (Years of Life Lost)	1	Global	3	Both	22	All ages	543	Alzheimer's disease and other dementias	99	Smoking	3	Rate	2017	12.094985266	34.497072266	2.939145757
4	YLLs (Years of Life Lost)	1	Global	1	Male	22	All ages	543	Alzheimer's disease and other dementias	99	Smoking	1	Number	2018	673244.835596819	1975556.08098413	158766.130047821
4	YLLs (Years of Life	1	Global	2	Fema	22	All ages	543	Alzheimer's disease and	99	Smok	1	Numb	2018	265360.9	736787.4	63079.15

	Lost)				le				other dementias		ing		er		12069571	54950867	96232884
4	YLLs (Years of Life Lost)	1	Global	3	Both	22	All ages	543	Alzheimer's disease and other dementias	99	Smoking	1	Number	2018	938605.74766639	2702110.62535519	217791.142429239
4	YLLs (Years of Life Lost)	1	Global	1	Male	22	All ages	543	Alzheimer's disease and other dementias	99	Smoking	3	Rate	2018	17.50494311	51.36615237	4.128055541
4	YLLs (Years of Life Lost)	1	Global	2	Female	22	All ages	543	Alzheimer's disease and other dementias	99	Smoking	3	Rate	2018	6.953902117	19.30784682	1.653017765
4	YLLs (Years of Life Lost)	1	Global	3	Both	22	All ages	543	Alzheimer's disease and other dementias	99	Smoking	3	Rate	2018	12.25009696	35.26626301	2.84247419
4	YLLs (Years of Life Lost)	1	Global	1	Male	22	All ages	543	Alzheimer's disease and other dementias	99	Smoking	1	Number	2019	694280.59028703 31	1964632.21584536	165219.536533612
4	YLLs (Years of	1	Global	2	Fem	22	All ag	543	Alzheimer's diseas	99	Smo	1	Num	201	269691	739070	64660.

	Life Lost)				ale		es		e and other dementias		king		ber	9	.017968223	.452628769	4108774653
4	YLLs (Years of Life Lost)	1	Global	3	Both	22	All ages	543	Alzheimer's disease and other dementias	99	Smoking	1	Number	2019	963971.608255254	2688764.24293951	229472.499255719
4	YLLs (Years of Life Lost)	1	Global	1	Male	22	All ages	543	Alzheimer's disease and other dementias	99	Smoking	3	Rate	2019	17.85811205	50.53377947	4.249735679
4	YLLs (Years of Life Lost)	1	Global	2	Female	22	All ages	543	Alzheimer's disease and other dementias	99	Smoking	3	Rate	2019	6.991218458	19.15897322	1.676196195
4	YLLs (Years of Life Lost)	1	Global	3	Both	22	All ages	543	Alzheimer's disease and other dementias	99	Smoking	3	Rate	2019	12.44584542	34.7146574	2.962721338
4	YLLs (Years of Life Lost)	1	Global	1	Male	22	All ages	543	Alzheimer's disease and other dementias	99	Smoking	1	Number	2020	709912.054004164	2007529.39297301	164841.552728874
4	YLLs (Years	1	Glob	2	Fe	22	All	54	Alzheimer's	99	Sm	1	Nu	20	2734	7534	6497

	of Life Lost)		al		male		ages	3	disease and other dementias		oking		mber	20	75.598033197	42.87153043	7.4662949519
4	YLLs (Years of Life Lost)	1	Global	3	Both	22	All ages	543	Alzheimer's disease and other dementias	99	Smoking	1	Number	2020	983387.652037361	2744293.96958298	228214.487442023
4	YLLs (Years of Life Lost)	1	Global	1	Male	22	All ages	543	Alzheimer's disease and other dementias	99	Smoking	3	Rate	2020	18.08270174	51.13528506	4.198802677
4	YLLs (Years of Life Lost)	1	Global	2	Female	22	All ages	543	Alzheimer's disease and other dementias	99	Smoking	3	Rate	2020	7.018591415	19.33667101	1.667608702
4	YLLs (Years of Life Lost)	1	Global	3	Both	22	All ages	543	Alzheimer's disease and other dementias	99	Smoking	3	Rate	2020	12.57148979	35.08266912	2.917461992
4	YLLs (Years of Life Lost)	1	Global	1	Male	22	All ages	543	Alzheimer's disease and other dementias	99	Smoking	1	Number	2021	737183.096350506	2115306.99851727	177427.403210802
4	YLLs	1	Gl	2	F	2	Al	5	Alzhei	9	S	1	N	2	28	77	65

	(Years of Life Lost)		obal		emale	2	l ages	43	mer's disease and other dementias	9	moking		umber	021	0148.256219806	1699.056687086	583.2474553306
4	YLLs (Years of Life Lost)	1	Global	3	Both	22	All ages	543	Alzheimer's disease and other dementias	99	Smoking	1	Number	2021	1017331.35257031	2876261.38953396	241831.61577932
4	YLLs (Years of Life Lost)	1	Global	1	Male	22	All ages	543	Alzheimer's disease and other dementias	99	Smoking	3	Rate	2021	18.61859601	53.4250536	4.481178634
4	YLLs (Years of Life Lost)	1	Global	2	Female	22	All ages	543	Alzheimer's disease and other dementias	99	Smoking	3	Rate	2021	7.124897598	19.62631083	1.667952278
4	YLLs (Years of Life Lost)	1	Global	3	Both	22	All ages	543	Alzheimer's disease and other dementias	99	Smoking	3	Rate	2021	12.89172229	36.44826533	3.064513862
4	YLLs (Years of Life Lost)	1	Global	1	Male	27	Age-standardized	543	Alzheimer's disease and other dementias	99	Smoking	3	Rate	1990	25.53594223	71.79177996	5.817074955

4	YLLs (Years of Life Lost)	1	Global	2	Female	27	Age-standardized	543	Alzheimer's disease and other dementias	99	Smoking	3	Rate	1990	9.14521793	25.61974115	2.123397542
4	YLLs (Years of Life Lost)	1	Global	3	Both	27	Age-standardized	543	Alzheimer's disease and other dementias	99	Smoking	3	Rate	1990	15.75625967	44.13263242	3.615148889
4	YLLs (Years of Life Lost)	1	Global	1	Male	27	Age-standardized	543	Alzheimer's disease and other dementias	99	Smoking	3	Rate	1991	25.35337662	72.1668298	5.813906903
4	YLLs (Years of Life Lost)	1	Global	2	Female	27	Age-standardized	543	Alzheimer's disease and other dementias	99	Smoking	3	Rate	1991	9.114785541	25.33952726	2.142116409
4	YLLs (Years of Life Lost)	1	Global	3	Both	27	Age-standardized	543	Alzheimer's disease and other dementias	99	Smoking	3	Rate	1991	15.68087159	44.25802339	3.657911406
4	YLLs (Years of Life	1	Global	1	Male	27	Age-stan	543	Alzheimer's disease and	99	Smok	3	Rate	1992	25.12504	70.52354	5.731642

	Lost)						dardized		other dementias		ing				314	627	394
4	YLLs (Years of Life Lost)	1	Global	2	Female	27	Age-standardized	543	Alzheimer's disease and other dementias	99	Smoking	3	Rate	1992	9.072828341	25.15888529	2.117362505
4	YLLs (Years of Life Lost)	1	Global	3	Both	27	Age-standardized	543	Alzheimer's disease and other dementias	99	Smoking	3	Rate	1992	15.58178155	43.7811185	3.645137493
4	YLLs (Years of Life Lost)	1	Global	1	Male	27	Age-standardized	543	Alzheimer's disease and other dementias	99	Smoking	3	Rate	1993	24.92544217	71.33174165	5.604953693
4	YLLs (Years of Life Lost)	1	Global	2	Female	27	Age-standardized	543	Alzheimer's disease and other dementias	99	Smoking	3	Rate	1993	9.038387867	24.94883574	2.080761025
4	YLLs (Years of Life Lost)	1	Global	3	Both	27	Age-standardized	543	Alzheimer's disease and other dementias	99	Smoking	3	Rate	1993	15.49904921	44.202044	3.53272808

4	YLLs (Years of Life Lost)	1	Global	1	Male	27	Age-standardized	543	Alzheimer's disease and other dementias	99	Smoking	3	Rate	1994	24.67112643	70.6789714	5.675134746
4	YLLs (Years of Life Lost)	1	Global	2	Female	27	Age-standardized	543	Alzheimer's disease and other dementias	99	Smoking	3	Rate	1994	8.985738115	24.9232642	2.089323541
4	YLLs (Years of Life Lost)	1	Global	3	Both	27	Age-standardized	543	Alzheimer's disease and other dementias	99	Smoking	3	Rate	1994	15.37965835	43.79966261	3.566482608
4	YLLs (Years of Life Lost)	1	Global	1	Male	27	Age-standardized	543	Alzheimer's disease and other dementias	99	Smoking	3	Rate	1995	24.43334068	69.29867386	5.547923509
4	YLLs (Years of Life Lost)	1	Global	2	Female	27	Age-standardized	543	Alzheimer's disease and other dementias	99	Smoking	3	Rate	1995	8.936840027	24.88536041	2.069592913
4	YLLs (Years of Life Lost)	1	Global	3	Both	27	Age-standan	543	Alzheimer's diseas e and	99	Smok	3	Rate	1995	15.27018	43.42292	3.504318

	Lost)						dardized		other dementias		ing				898	752	445
4	YLLs (Years of Life Lost)	1	Global	1	Male	27	Age-standardized	543	Alzheimer's disease and other dementias	99	Smoking	3	Rate	1996	24.16538753	67.76201685	5.527196237
4	YLLs (Years of Life Lost)	1	Global	2	Female	27	Age-standardized	543	Alzheimer's disease and other dementias	99	Smoking	3	Rate	1996	8.853666763	24.71674037	2.064070222
4	YLLs (Years of Life Lost)	1	Global	3	Both	27	Age-standardized	543	Alzheimer's disease and other dementias	99	Smoking	3	Rate	1996	15.12612694	42.84137204	3.502677186
4	YLLs (Years of Life Lost)	1	Global	1	Male	27	Age-standardized	543	Alzheimer's disease and other dementias	99	Smoking	3	Rate	1997	23.82418486	67.77155256	5.472286748
4	YLLs (Years of Life Lost)	1	Global	2	Female	27	Age-standardized	543	Alzheimer's disease and other dementias	99	Smoking	3	Rate	1997	8.726520776	24.3682826	2.060906123

4	YLLs (Years of Life Lost)	1	Global	3	Both	27	Age-standardized	543	Alzheimer's disease and other dementias	99	Smoking	3	Rate	1997	14.92259771	42.24969975	3.477986286
4	YLLs (Years of Life Lost)	1	Global	1	Male	27	Age-standardized	543	Alzheimer's disease and other dementias	99	Smoking	3	Rate	1998	23.47667883	66.79465094	5.434435632
4	YLLs (Years of Life Lost)	1	Global	2	Female	27	Age-standardized	543	Alzheimer's disease and other dementias	99	Smoking	3	Rate	1998	8.5910905	23.99547633	2.040857092
4	YLLs (Years of Life Lost)	1	Global	3	Both	27	Age-standardized	543	Alzheimer's disease and other dementias	99	Smoking	3	Rate	1998	14.70954847	41.75664504	3.447158626
4	YLLs (Years of Life Lost)	1	Global	1	Male	27	Age-standardized	543	Alzheimer's disease and other dementias	99	Smoking	3	Rate	1999	23.13013426	66.73726141	5.315028834
4	YLLs (Years of Life	1	Global	2	Fema	27	Age-stan	543	Alzheimer's disease and	99	Smok	3	Rate	1999	8.451364	23.63501	2.017594

	Lost)				le		dardized		other dementias		ing				727	051	755
4	YLLs (Years of Life Lost)	1	Global	3	Both	27	Age-standardized	543	Alzheimer’s disease and other dementias	99	Smoking	3	Rate	1999	14.49604011	41.34238379	3.391986322
4	YLLs (Years of Life Lost)	1	Global	1	Male	27	Age-standardized	543	Alzheimer’s disease and other dementias	99	Smoking	3	Rate	2000	22.86872185	64.85778397	5.273771498
4	YLLs (Years of Life Lost)	1	Global	2	Female	27	Age-standardized	543	Alzheimer’s disease and other dementias	99	Smoking	3	Rate	2000	8.313774251	23.17857539	1.983108204
4	YLLs (Years of Life Lost)	1	Global	3	Both	27	Age-standardized	543	Alzheimer’s disease and other dementias	99	Smoking	3	Rate	2000	14.32187849	40.30403527	3.353455331
4	YLLs (Years of Life Lost)	1	Global	1	Male	27	Age-standardized	543	Alzheimer’s disease and other dementias	99	Smoking	3	Rate	2001	22.61678454	64.3099073	5.201321475

4	YLLs (Years of Life Lost)	1	Global	2	Female	27	Age-standardized	543	Alzheimer's disease and other dementias	99	Smoking	3	Rate	2001	8.178496604	22.75942838	1.950684348
4	YLLs (Years of Life Lost)	1	Global	3	Both	27	Age-standardized	543	Alzheimer's disease and other dementias	99	Smoking	3	Rate	2001	14.15067407	39.75688058	3.30752445
4	YLLs (Years of Life Lost)	1	Global	1	Male	27	Age-standardized	543	Alzheimer's disease and other dementias	99	Smoking	3	Rate	2002	22.43265139	63.99994322	5.174279893
4	YLLs (Years of Life Lost)	1	Global	2	Female	27	Age-standardized	543	Alzheimer's disease and other dementias	99	Smoking	3	Rate	2002	8.04379513	22.408777	1.925550799
4	YLLs (Years of Life Lost)	1	Global	3	Both	27	Age-standardized	543	Alzheimer's disease and other dementias	99	Smoking	3	Rate	2002	14.01048566	39.65020292	3.276444651
4	YLLs (Years of Life	1	Global	1	Male	27	Age-stan	543	Alzheimer's disease and	99	Smok	3	Rate	2003	22.21735	63.05183	5.144320

	Lost)						dardized		other dementias		ing				758	062	536
4	YLLs (Years of Life Lost)	1	Global	2	Female	27	Age-standardized	543	Alzheimer's disease and other dementias	99	Smoking	3	Rate	2003	7.907200954	22.03384129	1.902815261
4	YLLs (Years of Life Lost)	1	Global	3	Both	27	Age-standardized	543	Alzheimer's disease and other dementias	99	Smoking	3	Rate	2003	13.85193684	39.02784331	3.264795557
4	YLLs (Years of Life Lost)	1	Global	1	Male	27	Age-standardized	543	Alzheimer's disease and other dementias	99	Smoking	3	Rate	2004	21.98482337	62.52050043	5.023649566
4	YLLs (Years of Life Lost)	1	Global	2	Female	27	Age-standardized	543	Alzheimer's disease and other dementias	99	Smoking	3	Rate	2004	7.7463744	21.73621951	1.864970473
4	YLLs (Years of Life Lost)	1	Global	3	Both	27	Age-standardized	543	Alzheimer's disease and other dementias	99	Smoking	3	Rate	2004	13.67346674	38.45247019	3.201854504

4	YLLs (Years of Life Lost)	1	Global	1	Male	27	Age-standardized	543	Alzheimer's disease and other dementias	99	Smoking	3	Rate	2005	21.786871633	61.576069963	5.060375003
4	YLLs (Years of Life Lost)	1	Global	2	Female	27	Age-standardized	543	Alzheimer's disease and other dementias	99	Smoking	3	Rate	2005	7.630690237	21.453132446	1.835602931
4	YLLs (Years of Life Lost)	1	Global	3	Both	27	Age-standardized	543	Alzheimer's disease and other dementias	99	Smoking	3	Rate	2005	13.538713544	37.898449987	3.175937018
4	YLLs (Years of Life Lost)	1	Global	1	Male	27	Age-standardized	543	Alzheimer's disease and other dementias	99	Smoking	3	Rate	2006	21.573589903	60.776349989	5.002685721
4	YLLs (Years of Life Lost)	1	Global	2	Female	27	Age-standardized	543	Alzheimer's disease and other dementias	99	Smoking	3	Rate	2006	7.491312278	21.023757767	1.793289071
4	YLLs (Years of Life	1	Global	3	Both	27	Age-stan	543	Alzheimer's disease and	99	Smok	3	Rate	2006	13.38452	37.69537	3.149930

	Lost)						dardized		other dementias		ing				04	211	551
4	YLLs (Years of Life Lost)	1	Global	1	Male	27	Age-standardized	543	Alzheimer's disease and other dementias	99	Smoking	3	Rate	2007	21.45772431	60.38725302	5.03210383
4	YLLs (Years of Life Lost)	1	Global	2	Female	27	Age-standardized	543	Alzheimer's disease and other dementias	99	Smoking	3	Rate	2007	7.360347136	20.568063364	1.783205702
4	YLLs (Years of Life Lost)	1	Global	3	Both	27	Age-standardized	543	Alzheimer's disease and other dementias	99	Smoking	3	Rate	2007	13.278490093	37.3087004	3.15393824
4	YLLs (Years of Life Lost)	1	Global	1	Male	27	Age-standardized	543	Alzheimer's disease and other dementias	99	Smoking	3	Rate	2008	21.361639958	60.902645597	4.989254081
4	YLLs (Years of Life Lost)	1	Global	2	Female	27	Age-standardized	543	Alzheimer's disease and other dementias	99	Smoking	3	Rate	2008	7.241214829	19.8314492	1.745751348

4	YLLs (Years of Life Lost)	1	Global	3	Both	27	Age-standardized	543	Alzheimer's disease and other dementias	99	Smoking	3	Rate	2008	13.18822492	37.31569343	3.124766729
4	YLLs (Years of Life Lost)	1	Global	1	Male	27	Age-standardized	543	Alzheimer's disease and other dementias	99	Smoking	3	Rate	2009	21.28038836	60.08496683	4.944177167
4	YLLs (Years of Life Lost)	1	Global	2	Female	27	Age-standardized	543	Alzheimer's disease and other dementias	99	Smoking	3	Rate	2009	7.111838216	19.67399588	1.715646956
4	YLLs (Years of Life Lost)	1	Global	3	Both	27	Age-standardized	543	Alzheimer's disease and other dementias	99	Smoking	3	Rate	2009	13.09804426	36.54242294	3.088939759
4	YLLs (Years of Life Lost)	1	Global	1	Male	27	Age-standardized	543	Alzheimer's disease and other dementias	99	Smoking	3	Rate	2010	21.19093019	59.72839077	5.008256628
4	YLLs (Years of Life	1	Global	2	Fema	27	Age-stan	543	Alzheimer's diseas e and	99	Smok	3	Rate	2010	7.00485 7	19.30007 3	1.68607 4

	Lost)				le		dardized		other dementias		ing				022	107	794
4	YLLs (Years of Life Lost)	1	Global	3	Both	27	Age-standardized	543	Alzheimer's disease and other dementias	99	Smoking	3	Rate	2010	13.01247753	36.51540639	3.094416733
4	YLLs (Years of Life Lost)	1	Global	1	Male	27	Age-standardized	543	Alzheimer's disease and other dementias	99	Smoking	3	Rate	2011	21.08695587	59.76409948	5.002989995
4	YLLs (Years of Life Lost)	1	Global	2	Female	27	Age-standardized	543	Alzheimer's disease and other dementias	99	Smoking	3	Rate	2011	6.888166509	19.18349225	1.649450742
4	YLLs (Years of Life Lost)	1	Global	3	Both	27	Age-standardized	543	Alzheimer's disease and other dementias	99	Smoking	3	Rate	2011	12.91515006	36.78620545	3.070028489
4	YLLs (Years of Life Lost)	1	Global	1	Male	27	Age-standardized	543	Alzheimer's disease and other dementias	99	Smoking	3	Rate	2012	20.98828856	60.36030278	4.833843292

4	YLLs (Years of Life Lost)	1	Global	2	Female	27	Age-standardized	543	Alzheimer's disease and other dementias	99	Smoking	3	Rate	2012	6.765900408	18.43308284	1.627711447
4	YLLs (Years of Life Lost)	1	Global	3	Both	27	Age-standardized	543	Alzheimer's disease and other dementias	99	Smoking	3	Rate	2012	12.81774215	36.39058393	2.994129775
4	YLLs (Years of Life Lost)	1	Global	1	Male	27	Age-standardized	543	Alzheimer's disease and other dementias	99	Smoking	3	Rate	2013	20.91629478	60.85492642	4.8769772
4	YLLs (Years of Life Lost)	1	Global	2	Female	27	Age-standardized	543	Alzheimer's disease and other dementias	99	Smoking	3	Rate	2013	6.641873547	18.30739287	1.577202288
4	YLLs (Years of Life Lost)	1	Global	3	Both	27	Age-standardized	543	Alzheimer's disease and other dementias	99	Smoking	3	Rate	2013	12.72600733	36.69752424	3.009144989
4	YLLs (Years of Life	1	Global	1	Male	27	Age-stan	543	Alzheimer's diseas e and	99	Smok	3	Rate	2014	20.86135	59.63278	4.880208

	Lost)						dardized		other dementias		ing				109	486	222
4	YLLs (Years of Life Lost)	1	Global	2	Female	27	Age-standardized	543	Alzheimer's disease and other dementias	99	Smoking	3	Rate	2014	6.530920528	18.231608833	1.538628201
4	YLLs (Years of Life Lost)	1	Global	3	Both	27	Age-standardized	543	Alzheimer's disease and other dementias	99	Smoking	3	Rate	2014	12.647386866	35.652614447	2.970361334
4	YLLs (Years of Life Lost)	1	Global	1	Male	27	Age-standardized	543	Alzheimer's disease and other dementias	99	Smoking	3	Rate	2015	20.810226799	59.516965562	4.944709951
4	YLLs (Years of Life Lost)	1	Global	2	Female	27	Age-standardized	543	Alzheimer's disease and other dementias	99	Smoking	3	Rate	2015	6.440199103	18.036128899	1.515734365
4	YLLs (Years of Life Lost)	1	Global	3	Both	27	Age-standardized	543	Alzheimer's disease and other dementias	99	Smoking	3	Rate	2015	12.582831444	35.336175551	2.969071663

4	YLLs (Years of Life Lost)	1	Global	1	Male	27	Age-standardized	543	Alzheimer's disease and other dementias	99	Smoking	3	Rate	2016	20.709582275	58.786630068	4.885195163
4	YLLs (Years of Life Lost)	1	Global	2	Female	27	Age-standardized	543	Alzheimer's disease and other dementias	99	Smoking	3	Rate	2016	6.352391868	17.674164459	1.502699723
4	YLLs (Years of Life Lost)	1	Global	3	Both	27	Age-standardized	543	Alzheimer's disease and other dementias	99	Smoking	3	Rate	2016	12.500026657	35.432671921	2.943927634
4	YLLs (Years of Life Lost)	1	Global	1	Male	27	Age-standardized	543	Alzheimer's disease and other dementias	99	Smoking	3	Rate	2017	20.631126555	59.049745551	5.011767274
4	YLLs (Years of Life Lost)	1	Global	2	Female	27	Age-standardized	543	Alzheimer's disease and other dementias	99	Smoking	3	Rate	2017	6.252862682	17.511366888	1.484547355
4	YLLs (Years of Life	1	Global	3	Both	27	Age-stan	543	Alzheimer's disease and	99	Smok	3	Rate	2017	12.42274	35.13400	3.022279

	Lost)						dardized		other dementias		ing				238	012	687
4	YLLs (Years of Life Lost)	1	Global	1	Male	27	Age-standardized	543	Alzheimer's disease and other dementias	99	Smoking	3	Rate	2018	20.48807452	59.2241474	4.795099535
4	YLLs (Years of Life Lost)	1	Global	2	Female	27	Age-standardized	543	Alzheimer's disease and other dementias	99	Smoking	3	Rate	2018	6.15586342	17.09947701	1.463207866
4	YLLs (Years of Life Lost)	1	Global	3	Both	27	Age-standardized	543	Alzheimer's disease and other dementias	99	Smoking	3	Rate	2018	12.31851479	35.12813431	2.846930051
4	YLLs (Years of Life Lost)	1	Global	1	Male	27	Age-standardized	543	Alzheimer's disease and other dementias	99	Smoking	3	Rate	2019	20.41909908	57.71028945	4.839129314
4	YLLs (Years of Life Lost)	1	Global	2	Female	27	Age-standardized	543	Alzheimer's disease and other dementias	99	Smoking	3	Rate	2019	6.055247784	16.61993426	1.450017215

4	YLLs (Years of Life Lost)	1	Global	3	Both	27	Age-standardized	543	Alzheimer's disease and other dementias	99	Smoking	3	Rate	2019	12.244198803	34.143404419	2.912150368
4	YLLs (Years of Life Lost)	1	Global	1	Male	27	Age-standardized	543	Alzheimer's disease and other dementias	99	Smoking	3	Rate	2020	20.286383995	56.610831577	4.687379347
4	YLLs (Years of Life Lost)	1	Global	2	Female	27	Age-standardized	543	Alzheimer's disease and other dementias	99	Smoking	3	Rate	2020	5.961800235	16.436623309	1.417188327
4	YLLs (Years of Life Lost)	1	Global	3	Both	27	Age-standardized	543	Alzheimer's disease and other dementias	99	Smoking	3	Rate	2020	12.134379105	33.463010999	2.805935757
4	YLLs (Years of Life Lost)	1	Global	1	Male	27	Age-standardized	543	Alzheimer's disease and other dementias	99	Smoking	3	Rate	2021	20.575125801	58.195116638	4.901680423
4	YLLs (Years of Life	1	Global	2	Fema	27	Age-stan	543	Alzheimer's diseas e and	99	Smok	3	Rate	2021	5.94999908	16.40613	1.395783

	Lost)				le		dardized		other dementias		ing				016	721	448
4	YLLs (Years of Life Lost)	1	Global	3	Both	27	Age-standardized	543	Alzheimer's disease and other dementias	99	Smoking	3	Rate	2021	12.24278629	34.28057038	2.901140305

Supplementary Table S7 cluster algorithmregion

measure_id	measure_name	location_id	location_name	sex_id	sex_name	age_id	age_name	cause_id	cause_name	rei_id	rei_name	metric_id	metric_name	year	val	upper	lower
1	Deaths	5	East Asia	3	Both	22	All ages	543	Alzheimer's disease and other dementias	99	Smoking	1	Number	1990	8275.34276309539	22997.5934034754	1857.67863535434
1	Deaths	5	East Asia	3	Both	22	All ages	543	Alzheimer's disease and other dementias	99	Smoking	3	Rate	1990	0.679733214	1.889012761	0.152588951
1	Deat	5	East	3	Bo	22	All	54	Alzheimer's	99	Sm	1	Numb	19	8607	2460	2041

	hs		Asia		th		ages	3	disease and other dementias		oking		er	91	.6897115666	3.040082109	.9592188826
1	Deaths	5	East Asia	3	Both	22	All ages	543	Alzheimer's disease and other dementias	99	Smoking	3	Rate	1991	0.697369963	1.993266686	0.165433592
1	Deaths	5	East Asia	3	Both	22	All ages	543	Alzheimer's disease and other dementias	99	Smoking	1	Number	1992	8893.72202179199	24889.6877893316	2056.14664526503
1	Deaths	5	East Asia	3	Both	22	All ages	543	Alzheimer's disease and other dementias	99	Smoking	3	Rate	1992	0.712491126	1.99395502	0.164721388
1	Deaths	5	East Asia	3	Both	22	All ages	543	Alzheimer's disease and other dementias	99	Smoking	1	Number	1993	9196.01560613036	25920.8200100795	2049.07526144974
1	Deaths	5	East Asia	3	Both	22	All ages	543	Alzheimer's disease and other dementias	99	Smoking	3	Rate	1993	0.72996433	2.057551316	0.162652165
1	De	5	Ea	3	B	2	Al	5	Alzheim	9	S	1	Nu	1	94	27	21

	aths		st Asia		oth	2	l ages	43	er’s disease and other dementias	9	moking		mber	994	64.58800069696	326.7896674926	91.68634501805
1	Deaths	5	East Asia	3	Both	22	All ages	543	Alzheimer’s disease and other dementias	99	Smoking	3	Rate	1994	0.745607207	2.152766852	0.172658032
1	Deaths	5	East Asia	3	Both	22	All ages	543	Alzheimer’s disease and other dementias	99	Smoking	1	Number	1995	9729.7593185872	27334.4524070104	2195.19544449585
1	Deaths	5	East Asia	3	Both	22	All ages	543	Alzheimer’s disease and other dementias	99	Smoking	3	Rate	1995	0.761743324	2.140015592	0.171861957
1	Deaths	5	East Asia	3	Both	22	All ages	543	Alzheimer’s disease and other dementias	99	Smoking	1	Number	1996	9988.34791919308	28322.3236862612	2309.9932540227
1	Deaths	5	East Asia	3	Both	22	All ages	543	Alzheimer’s disease and other dementias	99	Smoking	3	Rate	1996	0.777998919	2.206044221	0.179926878

1	Deaths	5	East Asia	3	Both	22	All ages	543	Alzheimer’s disease and other dementias	99	Smoking	1	Number	1997	10175.6779914717	28785.9188325328	2380.73380335762
1	Deaths	5	East Asia	3	Both	22	All ages	543	Alzheimer’s disease and other dementias	99	Smoking	3	Rate	1997	0.789157015	2.232441888	0.184633671
1	Deaths	5	East Asia	3	Both	22	All ages	543	Alzheimer’s disease and other dementias	99	Smoking	1	Number	1998	10344.3532295026	29442.381085726	2402.71430310303
1	Deaths	5	East Asia	3	Both	22	All ages	543	Alzheimer’s disease and other dementias	99	Smoking	3	Rate	1998	0.799110929	2.274451382	0.185611919
1	Deaths	5	East Asia	3	Both	22	All ages	543	Alzheimer’s disease and other dementias	99	Smoking	1	Number	1999	10517.6155412285	29988.1687366725	2435.66330006612
1	Deaths	5	East Asia	3	Both	22	All ages	543	Alzheimer’s disease and other dementi	99	Smokin	3	Rate	1999	0.809395903	2.307776018	0.187439433

									as		g						
1	Deaths	5	East Asia	3	Both	22	All ages	543	Alzheimer's disease and other dementias	99	Smoking	1	Number	2000	10797.2028134795	30702.2266696913	2528.27134592003
1	Deaths	5	East Asia	3	Both	22	All ages	543	Alzheimer's disease and other dementias	99	Smoking	3	Rate	2000	0.827527153	2.35310262	0.193773631
1	Deaths	5	East Asia	3	Both	22	All ages	543	Alzheimer's disease and other dementias	99	Smoking	1	Number	2001	11065.5753759436	31721.1746712114	2628.9563296511
1	Deaths	5	East Asia	3	Both	22	All ages	543	Alzheimer's disease and other dementias	99	Smoking	3	Rate	2001	0.844138639	2.419853312	0.200550224
1	Deaths	5	East Asia	3	Both	22	All ages	543	Alzheimer's disease and other dementias	99	Smoking	1	Number	2002	11392.2993792609	32512.1098948158	2686.24490795127
1	Deaths	5	East Asia	3	Both	22	All ages	543	Alzheimer's disease and other	99	Smoki	3	Rate	2002	0.86460569	2.46746987	0.20386952

									dementias		ng				8	1	4
1	Deaths	5	East Asia	3	Both	22	All ages	543	Alzheimer's disease and other dementias	99	Smoking	1	Number	2003	11702.3468008212	32643.2549764374	2768.70160496818
1	Deaths	5	East Asia	3	Both	22	All ages	543	Alzheimer's disease and other dementias	99	Smoking	3	Rate	2003	0.883345904	2.464060079	0.208994082
1	Deaths	5	East Asia	3	Both	22	All ages	543	Alzheimer's disease and other dementias	99	Smoking	1	Number	2004	12031.3299745272	33728.8191107513	2779.24639472058
1	Deaths	5	East Asia	3	Both	22	All ages	543	Alzheimer's disease and other dementias	99	Smoking	3	Rate	2004	0.90310582	2.531781016	0.208618133
1	Deaths	5	East Asia	3	Both	22	All ages	543	Alzheimer's disease and other dementias	99	Smoking	1	Number	2005	12412.9814907481	35468.7000345994	2866.50450836275
1	Deaths	5	East Asia	3	Both	22	All ages	543	Alzheimer's disease and	99	Smok	3	Rate	2005	0.926231	2.646601	0.213892

									other dementias		ing				312	913	708
1	Deaths	5	East Asia	3	Both	22	All ages	543	Alzheimer's disease and other dementias	99	Smoking	1	Number	2006	12826.5081192463	35741.3367086747	3091.61577681128
1	Deaths	5	East Asia	3	Both	22	All ages	543	Alzheimer's disease and other dementias	99	Smoking	3	Rate	2006	0.950866997	2.64961104	0.229190625
1	Deaths	5	East Asia	3	Both	22	All ages	543	Alzheimer's disease and other dementias	99	Smoking	1	Number	2007	13454.6144143477	38295.1660256287	3144.70089199338
1	Deaths	5	East Asia	3	Both	22	All ages	543	Alzheimer's disease and other dementias	99	Smoking	3	Rate	2007	0.990464839	2.819108322	0.231497951
1	Deaths	5	East Asia	3	Both	22	All ages	543	Alzheimer's disease and other dementias	99	Smoking	1	Number	2008	14187.9619664534	39753.9015527629	3395.81261671981
1	Deaths	5	East As	3	Bot	22	All ag	543	Alzheimer's disease	99	Smo	3	Rate	200	1.0370	2.9058	0.2482

			ia		h		es		and other dementias		king			8	9797	92381	23838
1	Deaths	5	East Asia	3	Both	22	All ages	543	Alzheimer's disease and other dementias	99	Smoking	1	Number	2009	149994.7771072722	414821.204832436	3419.97946089623
1	Deaths	5	East Asia	3	Both	22	All ages	543	Alzheimer's disease and other dementias	99	Smoking	3	Rate	2009	1.08870130909	3.036443965	0.248308867
1	Deaths	5	East Asia	3	Both	22	All ages	543	Alzheimer's disease and other dementias	99	Smoking	1	Number	2010	157330.4259059926	438722.1945359197	3707.25530226539
1	Deaths	5	East Asia	3	Both	22	All ages	543	Alzheimer's disease and other dementias	99	Smoking	3	Rate	2010	1.135368423	3.166545178	0.267577027
1	Deaths	5	East Asia	3	Both	22	All ages	543	Alzheimer's disease and other dementias	99	Smoking	1	Number	2011	164448.7735523503	469975.6187974901	3813.72348534032
1	Deat	5	East	3	Bo	22	All	54	Alzheimer's	99	Sm	3	Rate	20	1.18	3.37	0.27

	hs		Asia		th		ages	3	disease and other dementias		oking			11	1146057	3203889	3854123
1	Deaths	5	East Asia	3	Both	22	All ages	543	Alzheimer's disease and other dementias	99	Smoking	1	Number	2012	17213.9526363545	47846.4726826943	3904.48166163153
1	Deaths	5	East Asia	3	Both	22	All ages	543	Alzheimer's disease and other dementias	99	Smoking	3	Rate	2012	1.229457609	3.417298233	0.278866498
1	Deaths	5	East Asia	3	Both	22	All ages	543	Alzheimer's disease and other dementias	99	Smoking	1	Number	2013	17993.2351070289	53180.7458399497	4197.64046204034
1	Deaths	5	East Asia	3	Both	22	All ages	543	Alzheimer's disease and other dementias	99	Smoking	3	Rate	2013	1.27750452	3.775788111	0.298028933
1	Deaths	5	East Asia	3	Both	22	All ages	543	Alzheimer's disease and other dementias	99	Smoking	1	Number	2014	18865.6254069713	53151.824379939	4288.13486592578
1	De	5	Ea	3	B	2	Al	5	Alzheim	9	S	3	Ra	2	1.	3.	0.

	aths		st Asia		oth	2	l ages	43	er's disease and other dementias	9	moking		te	014	331185097	750467581	302576835
1	Deaths	5	East Asia	3	Both	22	All ages	543	Alzheimer's disease and other dementias	99	Smoking	1	Number	2015	19729.9983727041	57033.2224853683	4626.08946345475
1	Deaths	5	East Asia	3	Both	22	All ages	543	Alzheimer's disease and other dementias	99	Smoking	3	Rate	2015	1.382808865	3.997265693	0.324226966
1	Deaths	5	East Asia	3	Both	22	All ages	543	Alzheimer's disease and other dementias	99	Smoking	1	Number	2016	20529.46273	59116.8648661433	4883.06647318369
1	Deaths	5	East Asia	3	Both	22	All ages	543	Alzheimer's disease and other dementias	99	Smoking	3	Rate	2016	1.428205476	4.112676072	0.339707978
1	Deaths	5	East Asia	3	Both	22	All ages	543	Alzheimer's disease and other dementias	99	Smoking	1	Number	2017	21315.3716275031	63266.7923506329	5147.06503002457

1	Deaths	5	East Asia	3	Both	22	All ages	543	Alzheimer’s disease and other dementias	99	Smoking	3	Rate	2017	1.47217338	4.369601861	0.355488624
1	Deaths	5	East Asia	3	Both	22	All ages	543	Alzheimer’s disease and other dementias	99	Smoking	1	Number	2018	21945.8146608937	63577.7866403207	5047.52821991657
1	Deaths	5	East Asia	3	Both	22	All ages	543	Alzheimer’s disease and other dementias	99	Smoking	3	Rate	2018	1.506229275	4.363598478	0.346432105
1	Deaths	5	East Asia	3	Both	22	All ages	543	Alzheimer’s disease and other dementias	99	Smoking	1	Number	2019	22770.6511867505	62738.1567788145	5197.32013701337
1	Deaths	5	East Asia	3	Both	22	All ages	543	Alzheimer’s disease and other dementias	99	Smoking	3	Rate	2019	1.555064263	4.284544378	0.354937887
1	Deaths	5	East Asia	3	Both	22	All ages	543	Alzheimer’s disease and other dementias	99	Smoking	1	Number	2020	23456.79284025	63878.32846100	5521.653978863

															51	46	92
1	Deaths	5	East Asia	3	Both	22	All ages	543	Alzheimer's disease and other dementias	99	Smoking	3	Rate	2020	1.5962740997	4.3470273995	0.3757578155
1	Deaths	5	East Asia	3	Both	22	All ages	543	Alzheimer's disease and other dementias	99	Smoking	1	Number	2021	25333.4188515316	71868.5402751801	5966.93417361814
1	Deaths	5	East Asia	3	Both	22	All ages	543	Alzheimer's disease and other dementias	99	Smoking	3	Rate	2021	1.72011478	4.879804778	0.405149093
1	Deaths	5	East Asia	3	Both	27	Age-standardized	543	Alzheimer's disease and other dementias	99	Smoking	3	Rate	1990	1.667960921	4.691933274	0.374275605
1	Deaths	5	East Asia	3	Both	27	Age-standardized	543	Alzheimer's disease and other dementias	99	Smoking	3	Rate	1991	1.674713243	4.695897746	0.376792987
1	Deaths	5	East Asia	3	Both	27	Age-standa	543	Alzheimer's disease and other	99	Smoki	3	Rate	1992	1.67118132	4.71458525	0.38796573

							rdized		dementias		ng				4	2	9
1	Deaths	5	East Asia	3	Both	27	Age-standardized	543	Alzheimer's disease and other dementias	99	Smoking	3	Rate	1993	1.668769496	4.700158445	0.366521457
1	Deaths	5	East Asia	3	Both	27	Age-standardized	543	Alzheimer's disease and other dementias	99	Smoking	3	Rate	1994	1.660319052	4.665082148	0.379141956
1	Deaths	5	East Asia	3	Both	27	Age-standardized	543	Alzheimer's disease and other dementias	99	Smoking	3	Rate	1995	1.649792557	4.729227802	0.367418751
1	Deaths	5	East Asia	3	Both	27	Age-standardized	543	Alzheimer's disease and other dementias	99	Smoking	3	Rate	1996	1.636472128	4.755519654	0.37000756
1	Deaths	5	East Asia	3	Both	27	Age-standardized	543	Alzheimer's disease and other dementias	99	Smoking	3	Rate	1997	1.608678579	4.631515035	0.369299597
1	De	5	Ea	3	B	2	Ag	5	Alzheim	9	S	3	Ra	1	1.	4.	0.

	aths		st Asia		oth	7	e-standardized	43	er's disease and other dementias	9	moking		te	998	577258374	50971823	353266931
1	Deaths	5	East Asia	3	Both	27	Age-standardized	543	Alzheimer's disease and other dementias	99	Smoking	3	Rate	1999	1.546208857	4.450040423	0.352971841
1	Deaths	5	East Asia	3	Both	27	Age-standardized	543	Alzheimer's disease and other dementias	99	Smoking	3	Rate	2000	1.52724497	4.451347525	0.352974462
1	Deaths	5	East Asia	3	Both	27	Age-standardized	543	Alzheimer's disease and other dementias	99	Smoking	3	Rate	2001	1.507489106	4.312424644	0.343377676
1	Deaths	5	East Asia	3	Both	27	Age-standardized	543	Alzheimer's disease and other dementias	99	Smoking	3	Rate	2002	1.491890058	4.33315737	0.34046356
1	Deaths	5	East Asia	3	Both	27	Age-standa	543	Alzheimer's disease and other	99	Smoki	3	Rate	2003	1.47584520	4.32391463	0.33688195

							rdized		dementias		ng				9	8	5
1	Deaths	5	East Asia	3	Both	27	Age-standardized	543	Alzheimer's disease and other dementias	99	Smoking	3	Rate	2004	1.458281715	4.130163542	0.328902923
1	Deaths	5	East Asia	3	Both	27	Age-standardized	543	Alzheimer's disease and other dementias	99	Smoking	3	Rate	2005	1.446982481	4.09220441	0.32743935
1	Deaths	5	East Asia	3	Both	27	Age-standardized	543	Alzheimer's disease and other dementias	99	Smoking	3	Rate	2006	1.426790629	3.982162494	0.333092783
1	Deaths	5	East Asia	3	Both	27	Age-standardized	543	Alzheimer's disease and other dementias	99	Smoking	3	Rate	2007	1.421376563	4.044668863	0.325654039
1	Deaths	5	East Asia	3	Both	27	Age-standardized	543	Alzheimer's disease and other dementias	99	Smoking	3	Rate	2008	1.425025379	4.035313703	0.329886678
1	De	5	Ea	3	B	2	Ag	5	Alzheim	9	S	3	Ra	2	1.	4.	0.

	aths		st Asia		oth	7	e-standardized	43	er's disease and other dementias	9	moking		te	009	432224025	082263603	323039732
1	Deaths	5	East Asia	3	Both	27	Age-standardized	543	Alzheimer's disease and other dementias	99	Smoking	3	Rate	2010	1.435525128	3.995757396	0.329397452
1	Deaths	5	East Asia	3	Both	27	Age-standardized	543	Alzheimer's disease and other dementias	99	Smoking	3	Rate	2011	1.432764154	4.028075388	0.323212077
1	Deaths	5	East Asia	3	Both	27	Age-standardized	543	Alzheimer's disease and other dementias	99	Smoking	3	Rate	2012	1.429131458	3.994612444	0.326207622
1	Deaths	5	East Asia	3	Both	27	Age-standardized	543	Alzheimer's disease and other dementias	99	Smoking	3	Rate	2013	1.424190813	4.109156482	0.323202395
1	Deaths	5	East Asia	3	Both	27	Age-standada	543	Alzheimer's disease and other	99	Smoki	3	Rate	2014	1.42023496	4.13409676	0.33088686

							rdized		dementias		ng				3	6	8
1	Deaths	5	East Asia	3	Both	27	Age-standardized	543	Alzheimer's disease and other dementias	99	Smoking	3	Rate	2015	1.415023668	4.119857061	0.329547876
1	Deaths	5	East Asia	3	Both	27	Age-standardized	543	Alzheimer's disease and other dementias	99	Smoking	3	Rate	2016	1.402504471	4.118911836	0.327168364
1	Deaths	5	East Asia	3	Both	27	Age-standardized	543	Alzheimer's disease and other dementias	99	Smoking	3	Rate	2017	1.388300479	4.0536705	0.331969612
1	Deaths	5	East Asia	3	Both	27	Age-standardized	543	Alzheimer's disease and other dementias	99	Smoking	3	Rate	2018	1.365903225	3.823115758	0.312742349
1	Deaths	9	Southeast Asia	3	Both	22	All ages	543	Alzheimer's disease and other dementias	99	Smoking	1	Number	1990	1509.7297560896	4245.19039640798	343.896567377422
1	De	9	So	3	B	2	Al	5	Alzheim	9	S	3	Ra	1	0.	0.	0.

	aths		utheast Asia		oth	2	l ages	43	er's disease and other dementias	9	moking		te	990	324319446	911949833	073875701
1	Deaths	9	Southeast Asia	3	Both	22	All ages	543	Alzheimer's disease and other dementias	99	Smoking	1	Number	1991	1575.18175111176	4378.84342355225	362.865650229924
1	Deaths	9	Southeast Asia	3	Both	22	All ages	543	Alzheimer's disease and other dementias	99	Smoking	3	Rate	1991	0.332581209	0.92454159	0.076614839
1	Deaths	9	Southeast Asia	3	Both	22	All ages	543	Alzheimer's disease and other dementias	99	Smoking	1	Number	1992	1645.77850842501	4595.67951680014	377.691366168409
1	Deaths	9	Southeast Asia	3	Both	22	All ages	543	Alzheimer's disease and other dementias	99	Smoking	3	Rate	1992	0.341661871	0.954058189	0.078408327
1	Deaths	9	Southeast Asia	3	Both	22	All ages	543	Alzheimer's disease and other dementias	99	Smoking	1	Number	1993	1715.26067910667	4834.79716687359	397.667945867214

1	Deaths	9	Southeast Asia	3	Both	2	All ages	543	Alzheimer’s disease and other dementias	99	Smoking	3	Rate	1993	0.350258804	0.987272835	0.081204391
1	Deaths	9	Southeast Asia	3	Both	2	All ages	543	Alzheimer’s disease and other dementias	99	Smoking	1	Number	1994	1783.607773146435	5045.98252662873	415.452768164611
1	Deaths	9	Southeast Asia	3	Both	2	All ages	543	Alzheimer’s disease and other dementias	99	Smoking	3	Rate	1994	0.358417834	1.013995427	0.083485665
1	Deaths	9	Southeast Asia	3	Both	2	All ages	543	Alzheimer’s disease and other dementias	99	Smoking	1	Number	1995	1850.16534579917	5226.51378685359	427.569375295311
1	Deaths	9	Southeast Asia	3	Both	2	All ages	543	Alzheimer’s disease and other dementias	99	Smoking	3	Rate	1995	0.366068243	1.034102559	0.084597612
1	Deaths	9	Southeast Asia	3	Both	2	All ages	543	Alzheimer’s disease and other dementias	99	Smoking	1	Number	1996	1914.539762024	5398.348017337	437.9885346133

															35	92	73
1	Deaths	9	Southeast Asia	3	Both	22	All ages	543	Alzheimer's disease and other dementias	99	Smoking	3	Rate	1996	0.373151793	1.052160568	0.085365794
1	Deaths	9	Southeast Asia	3	Both	22	All ages	543	Alzheimer's disease and other dementias	99	Smoking	1	Number	1997	1977.76783518475	5503.34605718875	458.478906299707
1	Deaths	9	Southeast Asia	3	Both	22	All ages	543	Alzheimer's disease and other dementias	99	Smoking	3	Rate	1997	0.379860203	1.057000784	0.088057803
1	Deaths	9	Southeast Asia	3	Both	22	All ages	543	Alzheimer's disease and other dementias	99	Smoking	1	Number	1998	2045.16320212166	5738.60267189859	467.705304418334
1	Deaths	9	Southeast Asia	3	Both	22	All ages	543	Alzheimer's disease and other dementias	99	Smoking	3	Rate	1998	0.387228492	1.086539429	0.088554703
1	Deaths	9	Southeast As	3	Both	22	All ages	543	Alzheimer's disease and other dementi	99	Smokin	1	Number	1999	2109.0252022	5962.3101657	488.50444627

			ia						as		g				2593	9892	917
1	Deaths	9	Southeast Asia	3	Both	22	All ages	543	Alzheimer’s disease and other dementias	99	Smoking	3	Rate	1999	0.393814884	1.113332588	0.09121765
1	Deaths	9	Southeast Asia	3	Both	22	All ages	543	Alzheimer’s disease and other dementias	99	Smoking	1	Number	2000	2166.42475585571	6083.1820786339	504.827363663057
1	Deaths	9	Southeast Asia	3	Both	22	All ages	543	Alzheimer’s disease and other dementias	99	Smoking	3	Rate	2000	0.399093551	1.120629153	0.092998081
1	Deaths	9	Southeast Asia	3	Both	22	All ages	543	Alzheimer’s disease and other dementias	99	Smoking	1	Number	2001	2217.00946491508	6217.9911303709	511.092940188739
1	Deaths	9	Southeast Asia	3	Both	22	All ages	543	Alzheimer’s disease and other dementias	99	Smoking	3	Rate	2001	0.403017155	1.13033216	0.09290859
1	Deaths	9	Southeast	3	Both	22	All ages	543	Alzheimer’s disease and other	99	Smoki	1	Number	2002	2270.92651	6400.50667	523.982829

			Asia						dementias		ng				566491	386559	378638
1	Deaths	9	Southeast Asia	3	Both	22	All ages	543	Alzheimer’s disease and other dementias	99	Smoking	3	Rate	2002	0.407465576	1.148423836	0.094016677
1	Deaths	9	Southeast Asia	3	Both	22	All ages	543	Alzheimer’s disease and other dementias	99	Smoking	1	Number	2003	2316.20375058768	6517.70154041029	537.963471390284
1	Deaths	9	Southeast Asia	3	Both	22	All ages	543	Alzheimer’s disease and other dementias	99	Smoking	3	Rate	2003	0.410299383	1.154565492	0.095296487
1	Deaths	9	Southeast Asia	3	Both	22	All ages	543	Alzheimer’s disease and other dementias	99	Smoking	1	Number	2004	2357.85157910052	6732.66679970572	533.547487186869
1	Deaths	9	Southeast Asia	3	Both	22	All ages	543	Alzheimer’s disease and other dementias	99	Smoking	3	Rate	2004	0.412500343	1.17786352	0.093342822
1	Deaths	9	Southeas	3	Both	22	All ages	543	Alzheimer’s disease and	99	Smok	1	Number	2005	2400.797	6820.023	554.4458

			tAsia						other dementias		ing				44564566	9776566	72339248
1	Deaths	9	Southeast Asia	3	Both	22	All ages	543	Alzheimer's disease and other dementias	99	Smoking	3	Rate	2005	0.414853684	1.178488456	0.095807297
1	Deaths	9	Southeast Asia	3	Both	22	All ages	543	Alzheimer's disease and other dementias	99	Smoking	1	Number	2006	2444.82096653537	6893.44933047792	565.365981055348
1	Deaths	9	Southeast Asia	3	Both	22	All ages	543	Alzheimer's disease and other dementias	99	Smoking	3	Rate	2006	0.41723673	1.176446168	0.096486187
1	Deaths	9	Southeast Asia	3	Both	22	All ages	543	Alzheimer's disease and other dementias	99	Smoking	1	Number	2007	2489.47850734579	6968.78337975299	576.042800641423
1	Deaths	9	Southeast Asia	3	Both	22	All ages	543	Alzheimer's disease and other dementias	99	Smoking	3	Rate	2007	0.419622992	1.174648315	0.097096963
1	Deaths	9	Southe	3	Bot	22	All ag	543	Alzheimer's disease	99	Smo	1	Number	200	2531.0	7165.5	594.32

			ast Asia		h		es		and other dementias		king			8	7952968462	5437701797	9860320696
1	Deaths	9	Southeast Asia	3	Both	22	All ages	543	Alzheimer's disease and other dementias	99	Smoking	3	Rate	2008	0.420873575	1.191504436	0.098826501
1	Deaths	9	Southeast Asia	3	Both	22	All ages	543	Alzheimer's disease and other dementias	99	Smoking	1	Number	2009	2573.03465633786	7329.53273983449	602.125388433786
1	Deaths	9	Southeast Asia	3	Both	22	All ages	543	Alzheimer's disease and other dementias	99	Smoking	3	Rate	2009	0.421511825	1.200716328	0.098639546
1	Deaths	9	Southeast Asia	3	Both	22	All ages	543	Alzheimer's disease and other dementias	99	Smoking	1	Number	2010	2625.88634220168	7414.96021799461	624.793137562734
1	Deaths	9	Southeast Asia	3	Both	22	All ages	543	Alzheimer's disease and other dementias	99	Smoking	3	Rate	2010	0.424841924	1.199665773	0.101085228
1	Deat	9	Sout	3	Bo	22	All	54	Alzheimer's	99	Sm	1	Numb	20	2681	7615	634.

	hs		heast Asia		th		ages	3	disease and other dementias		oking		er	11	.60568432104	.70693682002	190845362614
1	Deaths	9	Southeast Asia	3	Both	22	All ages	543	Alzheimer's disease and other dementias	99	Smoking	3	Rate	2011	0.429588454	1.220022687	0.101596244
1	Deaths	9	Southeast Asia	3	Both	22	All ages	543	Alzheimer's disease and other dementias	99	Smoking	1	Number	2012	2738.4201481554	7933.89697303528	640.086312748529
1	Deaths	9	Southeast Asia	3	Both	22	All ages	543	Alzheimer's disease and other dementias	99	Smoking	3	Rate	2012	0.433819071	1.25688376	0.101402135
1	Deaths	9	Southeast Asia	3	Both	22	All ages	543	Alzheimer's disease and other dementias	99	Smoking	1	Number	2013	2802.33570305133	7985.78833747329	651.377789484907
1	Deaths	9	Southeast Asia	3	Both	22	All ages	543	Alzheimer's disease and other dementias	99	Smoking	3	Rate	2013	0.438533196	1.249683712	0.101933107
1	De	9	So	3	B	2	Al	5	Alzheim	9	S	1	Nu	2	28	82	67

	aths		utheast Asia		oth	2	l ages	43	er's disease and other dementias	9	moking		mber	014	74.72997886184	07.50472429253	0.767794594996
1	Deaths	9	Southeast Asia	3	Both	22	All ages	543	Alzheimer's disease and other dementias	99	Smoking	3	Rate	2014	0.444419932	1.268842193	0.103697592
1	Deaths	9	Southeast Asia	3	Both	22	All ages	543	Alzheimer's disease and other dementias	99	Smoking	1	Number	2015	2949.95592066278	8310.482579	685.89018121955
1	Deaths	9	Southeast Asia	3	Both	22	All ages	543	Alzheimer's disease and other dementias	99	Smoking	3	Rate	2015	0.450642817	1.269530589	0.10477834
1	Deaths	9	Southeast Asia	3	Both	22	All ages	543	Alzheimer's disease and other dementias	99	Smoking	1	Number	2016	3042.25809114815	8630.99770826083	724.463845521556
1	Deaths	9	Southeast Asia	3	Both	22	All ages	543	Alzheimer's disease and other dementias	99	Smoking	3	Rate	2016	0.459369171	1.303247173	0.10939123

1	Deaths	9	Southeast Asia	3	Both	2	All ages	543	Alzheimer's disease and other dementias	99	Smoking	1	Number	2017	3135.50194991075	9016.2638124238	752.146922535942
1	Deaths	9	Southeast Asia	3	Both	2	All ages	543	Alzheimer's disease and other dementias	99	Smoking	3	Rate	2017	0.468078156	1.345977838	0.112282993
1	Deaths	9	Southeast Asia	3	Both	2	All ages	543	Alzheimer's disease and other dementias	99	Smoking	1	Number	2018	3237.54696784785	9347.89084142854	760.99290442732
1	Deaths	9	Southeast Asia	3	Both	2	All ages	543	Alzheimer's disease and other dementias	99	Smoking	3	Rate	2018	0.479527074	1.380010775	0.112343889
1	Deaths	9	Southeast Asia	3	Both	2	All ages	543	Alzheimer's disease and other dementias	99	Smoking	1	Number	2019	3346.3558487035	9716.63198770186	782.80934965672
1	Deaths	9	Southeast As	3	Both	2	All ages	543	Alzheimer's disease and other dementi	99	Smokin	3	Rate	2019	0.488707947	1.419034759	0.114322913

			ia						as		g						
1	Deaths	9	Southeast Asia	3	Both	22	All ages	543	Alzheimer's disease and other dementias	99	Smoking	1	Number	2020	3455.57880561451	9998.50616934627	796.314250081108
1	Deaths	9	Southeast Asia	3	Both	22	All ages	543	Alzheimer's disease and other dementias	99	Smoking	3	Rate	2020	0.499486418	1.445233438	0.115103193
1	Deaths	9	Southeast Asia	3	Both	22	All ages	543	Alzheimer's disease and other dementias	99	Smoking	1	Number	2021	3541.54873445648	10246.3737919155	832.346173435701
1	Deaths	9	Southeast Asia	3	Both	22	All ages	543	Alzheimer's disease and other dementias	99	Smoking	3	Rate	2021	0.507163796	1.467321283	0.119195266
1	Deaths	9	Southeast Asia	3	Both	27	Age-standardized	543	Alzheimer's disease and other dementias	99	Smoking	3	Rate	1990	0.927574386	2.676590348	0.205965849
1	Deaths	9	Southeast	3	Both	27	Age-standa	543	Alzheimer's disease and other	99	Smoki	3	Rate	1991	0.9391367 5	2.6919329 9	0.2108873 8

			Asia				rdized		dementias		ng				3	4	8
1	Deaths	9	Southeast Asia	3	Both	27	Age-standardized	543	Alzheimer's disease and other dementias	99	Smoking	3	Rate	1992	0.950233212	2.722308107	0.213721809
1	Deaths	9	Southeast Asia	3	Both	27	Age-standardized	543	Alzheimer's disease and other dementias	99	Smoking	3	Rate	1993	0.95926746	2.745840584	0.217194115
1	Deaths	9	Southeast Asia	3	Both	27	Age-standardized	543	Alzheimer's disease and other dementias	99	Smoking	3	Rate	1994	0.966409903	2.735222859	0.220017708
1	Deaths	9	Southeast Asia	3	Both	27	Age-standardized	543	Alzheimer's disease and other dementias	99	Smoking	3	Rate	1995	0.971430106	2.750911334	0.221399091
1	Deaths	9	Southeast Asia	3	Both	27	Age-standardized	543	Alzheimer's disease and other dementias	99	Smoking	3	Rate	1996	0.97536078	2.77399936	0.222377286
1	De	9	So	3	B	2	Ag	5	Alzheim	9	S	3	Ra	1	0.	2.	0.

	aths		utheast Asia		oth	7	e-standardized	43	er's disease and other dementias	9	moking		te	997	975558362	793307059	224027117
1	Deaths	9	Southeast Asia	3	Both	27	Age-standardized	543	Alzheimer's disease and other dementias	99	Smoking	3	Rate	1998	0.9772285578	2.7957196656	0.2202287717
1	Deaths	9	Southeast Asia	3	Both	27	Age-standardized	543	Alzheimer's disease and other dementias	99	Smoking	3	Rate	1999	0.9762313377	2.7604790116	0.22498444
1	Deaths	9	Southeast Asia	3	Both	27	Age-standardized	543	Alzheimer's disease and other dementias	99	Smoking	3	Rate	2000	0.9720619244	2.7523256695	0.2229276222
1	Deaths	9	Southeast Asia	3	Both	27	Age-standardized	543	Alzheimer's disease and other dementias	99	Smoking	3	Rate	2001	0.9641456645	2.77364729	0.2209099555
1	Deaths	9	Southeast	3	Both	27	Age-standa	543	Alzheimer's disease and other	99	Smoki	3	Rate	2002	0.95581919	2.76210119	0.21899890

			Asia				rdized		dementias		ng				2	7	5
1	Deaths	9	Southeast Asia	3	Both	27	Age-standardized	543	Alzheimer’s disease and other dementias	99	Smoking	3	Rate	2003	0.943714972	2.732522522	0.216785158
1	Deaths	9	Southeast Asia	3	Both	27	Age-standardized	543	Alzheimer’s disease and other dementias	99	Smoking	3	Rate	2004	0.930795083	2.675826951	0.20939009
1	Deaths	9	Southeast Asia	3	Both	27	Age-standardized	543	Alzheimer’s disease and other dementias	99	Smoking	3	Rate	2005	0.919312525	2.650118895	0.20770321
1	Deaths	9	Southeast Asia	3	Both	27	Age-standardized	543	Alzheimer’s disease and other dementias	99	Smoking	3	Rate	2006	0.906849106	2.624509567	0.205538164
1	Deaths	9	Southeast Asia	3	Both	27	Age-standardized	543	Alzheimer’s disease and other dementias	99	Smoking	3	Rate	2007	0.893113373	2.545661522	0.203080667
1	De	9	So	3	B	2	Ag	5	Alzheim	9	S	3	Ra	2	0.	2.	0.

	aths		utheast Asia		oth	7	e-standardized	43	er's disease and other dementias	9	moking		te	008	878166663	531721965	204569701
1	Deaths	9	Southeast Asia	3	Both	27	Age-standardized	543	Alzheimer's disease and other dementias	99	Smoking	3	Rate	2009	0.862599871	2.483113812	0.199906092
1	Deaths	9	Southeast Asia	3	Both	27	Age-standardized	543	Alzheimer's disease and other dementias	99	Smoking	3	Rate	2010	0.84981412	2.463346176	0.200253533
1	Deaths	9	Southeast Asia	3	Both	27	Age-standardized	543	Alzheimer's disease and other dementias	99	Smoking	3	Rate	2011	0.836087071	2.434538156	0.189796089
1	Deaths	9	Southeast Asia	3	Both	27	Age-standardized	543	Alzheimer's disease and other dementias	99	Smoking	3	Rate	2012	0.821398772	2.361463241	0.186328669
1	Deaths	9	Southeast Asia	3	Both	27	Age-standa	543	Alzheimer's disease and other	99	Smoki	3	Rate	2013	0.80800838	2.34988352	0.18796672

			Asia				rdized		dementias		ng				8	3	6
1	Deaths	9	Southeast Asia	3	Both	27	Age-standardized	543	Alzheimer's disease and other dementias	99	Smoking	3	Rate	2014	0.796364353	2.308637959	0.183126834
1	Deaths	9	Southeast Asia	3	Both	27	Age-standardized	543	Alzheimer's disease and other dementias	99	Smoking	3	Rate	2015	0.785525749	2.265511432	0.180361178
1	Deaths	9	Southeast Asia	3	Both	27	Age-standardized	543	Alzheimer's disease and other dementias	99	Smoking	3	Rate	2016	0.777206353	2.205488703	0.178375141
1	Deaths	9	Southeast Asia	3	Both	27	Age-standardized	543	Alzheimer's disease and other dementias	99	Smoking	3	Rate	2017	0.768775067	2.210149856	0.179903705
1	Deaths	9	Southeast Asia	3	Both	27	Age-standardized	543	Alzheimer's disease and other dementias	99	Smoking	3	Rate	2018	0.762380881	2.238626571	0.178413284
1	De	5	Ea	3	B	2	Ag	5	Alzheim	9	S	3	Ra	2	1.	3.	0.

	aths		st Asia		oth	7	e-standardized	43	er's disease and other dementias	9	moking		te	019	351988721	760511344	308488474
1	Deaths	5	East Asia	3	Both	27	Age-standardized	543	Alzheimer's disease and other dementias	99	Smoking	3	Rate	2020	1.334182906	3.595123319	0.313323545
1	Deaths	5	East Asia	3	Both	27	Age-standardized	543	Alzheimer's disease and other dementias	99	Smoking	3	Rate	2021	1.380551485	3.868798987	0.321659157
1	Deaths	9	Southeast Asia	3	Both	27	Age-standardized	543	Alzheimer's disease and other dementias	99	Smoking	3	Rate	2019	0.757176096	2.227412473	0.178611437
1	Deaths	9	Southeast Asia	3	Both	27	Age-standardized	543	Alzheimer's disease and other dementias	99	Smoking	3	Rate	2020	0.752360359	2.202982553	0.172890744
1	Deaths	9	Southeast	3	Both	27	Age-standa	543	Alzheimer's disease and other	99	Smoki	3	Rate	2021	0.74675073	2.19361924	0.17051146

			Asia				rdized		dementias		ng				9	5	5
1	Deaths	21	Oceania	3	Both	22	All ages	543	Alzheimer's disease and other dementias	99	Smoking	1	Number	1990	8.80165038945309	25.7290807558099	1.89486611507643
1	Deaths	21	Oceania	3	Both	22	All ages	543	Alzheimer's disease and other dementias	99	Smoking	3	Rate	1990	0.134377415	0.392813531	0.028929485
1	Deaths	21	Oceania	3	Both	22	All ages	543	Alzheimer's disease and other dementias	99	Smoking	1	Number	1991	9.0355860934645	26.3875177534893	1.96399214657844
1	Deaths	21	Oceania	3	Both	22	All ages	543	Alzheimer's disease and other dementias	99	Smoking	3	Rate	1991	0.134785145	0.393626421	0.029297155
1	Deaths	21	Oceania	3	Both	22	All ages	543	Alzheimer's disease and other dementias	99	Smoking	1	Number	1992	9.2706588898417	27.0462146202562	2.02365446063852
1	Deaths	21	Oceani	3	Bot	22	All ag	543	Alzheimer's disease	99	Smo	3	Rate	199	0.1351	0.3942	0.0295

			a		h		es		and other dementias		king			2	44653	70929	00177
1	Deaths	21	Oceania	3	Both	22	All ages	543	Alzheimer's disease and other dementias	99	Smoking	1	Number	1993	9.51945749103839	27.8585336778538	2.10729682832634
1	Deaths	21	Oceania	3	Both	22	All ages	543	Alzheimer's disease and other dementias	99	Smoking	3	Rate	1993	0.135598098	0.396825572	0.030016988
1	Deaths	21	Oceania	3	Both	22	All ages	543	Alzheimer's disease and other dementias	99	Smoking	1	Number	1994	9.75593211886143	28.3524263387031	2.17744358540589
1	Deaths	21	Oceania	3	Both	22	All ages	543	Alzheimer's disease and other dementias	99	Smoking	3	Rate	1994	0.135737215	0.394475827	0.030295427
1	Deaths	21	Oceania	3	Both	22	All ages	543	Alzheimer's disease and other dementias	99	Smoking	1	Number	1995	9.9859958502599	28.8368435497034	2.25896530496411
1	Deat	21	Ocea	3	Bo	22	All	54	Alzheimer's	99	Sm	3	Rate	19	0.13	0.39	0.03

	hs		nia		th		ages	3	disease and other dementias		oking			95	563972	1690669	0683512
1	Deaths	21	Oceania	3	Both	22	All ages	543	Alzheimer's disease and other dementias	99	Smoking	1	Number	1996	10.2272107995934	29.4316764533885	2.31672081190001
1	Deaths	21	Oceania	3	Both	22	All ages	543	Alzheimer's disease and other dementias	99	Smoking	3	Rate	1996	0.135547649	0.390076495	0.030704956
1	Deaths	21	Oceania	3	Both	22	All ages	543	Alzheimer's disease and other dementias	99	Smoking	1	Number	1997	10.4831859533197	30.4681271780255	2.36644232040592
1	Deaths	21	Oceania	3	Both	22	All ages	543	Alzheimer's disease and other dementias	99	Smoking	3	Rate	1997	0.135513158	0.393852799	0.030590326
1	Deaths	21	Oceania	3	Both	22	All ages	543	Alzheimer's disease and other dementias	99	Smoking	1	Number	1998	10.7465518034148	31.0401852959671	2.42756644039595
1	De	21	Oc	3	B	2	Al	5	Alzheim	9	S	3	Ra	1	0.	0.	0.

	aths		eania		oth	2	l ages	43	er's disease and other dementias	9	moking		te	998	135463132	391269759	030600118
1	Deaths	21	Oceania	3	Both	22	All ages	543	Alzheimer's disease and other dementias	99	Smoking	1	Number	1999	11.0123724191777	32.1133499018935	2.49586677732846
1	Deaths	21	Oceania	3	Both	22	All ages	543	Alzheimer's disease and other dementias	99	Smoking	3	Rate	1999	0.135341329	0.394670947	0.030674038
1	Deaths	21	Oceania	3	Both	22	All ages	543	Alzheimer's disease and other dementias	99	Smoking	1	Number	2000	11.2879506306275	33.220292270324	2.54785461068516
1	Deaths	21	Oceania	3	Both	22	All ages	543	Alzheimer's disease and other dementias	99	Smoking	3	Rate	2000	0.135257874	0.398062168	0.030529669
1	Deaths	21	Oceania	3	Both	22	All ages	543	Alzheimer's disease and other dementias	99	Smoking	1	Number	2001	11.4903825490471	33.8567852391114	2.61428490067821

1	Deaths	21	Oceania	3	Both	2	All ages	543	Alzheimer's disease and other dementias	99	Smoking	3	Rate	2001	0.134301796	0.395724604	0.030556264
1	Deaths	21	Oceania	3	Both	2	All ages	543	Alzheimer's disease and other dementias	99	Smoking	1	Number	2002	11.7471842799471	34.8260761110307	2.68348101997219
1	Deaths	21	Oceania	3	Both	2	All ages	543	Alzheimer's disease and other dementias	99	Smoking	3	Rate	2002	0.133993476	0.39724132	0.030608948
1	Deaths	21	Oceania	3	Both	2	All ages	543	Alzheimer's disease and other dementias	99	Smoking	1	Number	2003	11.9769796086889	35.6231452832511	2.74489232763119
1	Deaths	21	Oceania	3	Both	2	All ages	543	Alzheimer's disease and other dementias	99	Smoking	3	Rate	2003	0.133337457	0.396585762	0.030558369
1	Deaths	21	Oceania	3	Both	2	All ages	543	Alzheimer's disease and other dementias	99	Smoking	1	Number	2004	12.18782790697	36.47880014779	2.776000074126

															27	33	87
1	Deaths	21	Oceania	3	Both	22	All ages	543	Alzheimer's disease and other dementias	99	Smoking	3	Rate	2004	0.132415421	0.396326213	0.030160027
1	Deaths	21	Oceania	3	Both	22	All ages	543	Alzheimer's disease and other dementias	99	Smoking	1	Number	2005	12.4451832037443	37.5308707604478	2.82979049981001
1	Deaths	21	Oceania	3	Both	22	All ages	543	Alzheimer's disease and other dementias	99	Smoking	3	Rate	2005	0.131942285	0.397897624	0.030001087
1	Deaths	21	Oceania	3	Both	22	All ages	543	Alzheimer's disease and other dementias	99	Smoking	1	Number	2006	12.6984412950399	38.5624455387006	2.90214166750496
1	Deaths	21	Oceania	3	Both	22	All ages	543	Alzheimer's disease and other dementias	99	Smoking	3	Rate	2006	0.131361163	0.398915709	0.030021693
1	Deaths	21	Oceania	3	Both	22	All ages	543	Alzheimer's disease and other dementi	99	Smokin	1	Number	2007	12.990678798	39.195588891	2.9734127887

									as		g				7149	5417	6776
1	Deaths	21	Oceania	3	Both	22	All ages	543	Alzheimer's disease and other dementias	99	Smoking	3	Rate	2007	0.131118624	0.395612252	0.030011503
1	Deaths	21	Oceania	3	Both	22	All ages	543	Alzheimer's disease and other dementias	99	Smoking	1	Number	2008	13.2422435846942	39.7002600287996	3.02572136281534
1	Deaths	21	Oceania	3	Both	22	All ages	543	Alzheimer's disease and other dementias	99	Smoking	3	Rate	2008	0.130412884	0.390977961	0.029798051
1	Deaths	21	Oceania	3	Both	22	All ages	543	Alzheimer's disease and other dementias	99	Smoking	1	Number	2009	13.5136073696003	40.5800572007218	3.10086242728983
1	Deaths	21	Oceania	3	Both	22	All ages	543	Alzheimer's disease and other dementias	99	Smoking	3	Rate	2009	0.129855903	0.389944473	0.029797025
1	Deaths	21	Oceania	3	Both	22	All ages	543	Alzheimer's disease and other	99	Smoki	1	Number	2010	13.9131977	41.4819346	3.16972889

									dementias		ng				40018	744124	441649
1	Deaths	21	Oceania	3	Both	22	All ages	543	Alzheimer's disease and other dementias	99	Smoking	3	Rate	2010	0.130445487	0.38892074	0.029718318
1	Deaths	21	Oceania	3	Both	22	All ages	543	Alzheimer's disease and other dementias	99	Smoking	1	Number	2011	14.3919278233247	42.9072787586786	3.27430795210391
1	Deaths	21	Oceania	3	Both	22	All ages	543	Alzheimer's disease and other dementias	99	Smoking	3	Rate	2011	0.131646035	0.392482035	0.029950794
1	Deaths	21	Oceania	3	Both	22	All ages	543	Alzheimer's disease and other dementias	99	Smoking	1	Number	2012	14.8685067617122	43.9196005940487	3.44130721148455
1	Deaths	21	Oceania	3	Both	22	All ages	543	Alzheimer's disease and other dementias	99	Smoking	3	Rate	2012	0.132695505	0.391964955	0.030712297
1	Deaths	21	Oceania	3	Both	22	All ages	543	Alzheimer's disease and	99	Smok	1	Number	2013	15.31388	45.11405	3.524738

									other dementias		ing				99675408	71606608	84849629
1	Deaths	21	Oceania	3	Both	22	All ages	543	Alzheimer’s disease and other dementias	99	Smoking	3	Rate	2013	0.133343328	0.392823673	0.030691118
1	Deaths	21	Oceania	3	Both	22	All ages	543	Alzheimer’s disease and other dementias	99	Smoking	1	Number	2014	15.8694932891491	46.3450056208113	3.67295691891048
1	Deaths	21	Oceania	3	Both	22	All ages	543	Alzheimer’s disease and other dementias	99	Smoking	3	Rate	2014	0.134819393	0.393724325	0.031203632
1	Deaths	21	Oceania	3	Both	22	All ages	543	Alzheimer’s disease and other dementias	99	Smoking	1	Number	2015	16.4490825459547	47.9843894772816	3.7615363456959
1	Deaths	21	Oceania	3	Both	22	All ages	543	Alzheimer’s disease and other dementias	99	Smoking	3	Rate	2015	0.136369629	0.397810234	0.031184676
1	Deaths	21	Oceani	3	Bot	22	All ag	543	Alzheimer’s disease	99	Smo	1	Number	201	16.876	49.369	3.8572

			a		h		es		and other dementias		king			6	0722680666	0275518303	3815118005
1	Deaths	21	Oceania	3	Both	22	All ages	543	Alzheimer's disease and other dementias	99	Smoking	3	Rate	2016	0.13655565656	0.39947802	0.031211509
1	Deaths	21	Oceania	3	Both	22	All ages	543	Alzheimer's disease and other dementias	99	Smoking	1	Number	2017	17.5049213817304	51.3431364488953	3.97605158128188
1	Deaths	21	Oceania	3	Both	22	All ages	543	Alzheimer's disease and other dementias	99	Smoking	3	Rate	2017	0.138255552	0.405513027	0.031403238

© 2025 Xu H. et al.
